# Transcriptome Analysis and Systemic RNAi Response in the African Sweetpotato Weevil (*Cylas puncticollis*, Coleoptera, Brentidae)

**DOI:** 10.1371/journal.pone.0115336

**Published:** 2015-01-15

**Authors:** Katterinne Prentice, Ine Pertry, Olivier Christiaens, Lander Bauters, Ana Bailey, Chuck Niblett, Marc Ghislain, Godelieve Gheysen, Guy Smagghe

**Affiliations:** 1 Department of Crop Protection, Faculty of Bioscience Engineering, Ghent University, B-9000 Ghent, Belgium; 2 Department of Molecular Biotechnology, Faculty of Bioscience Engineering, Ghent University, B-9000 Ghent, Belgium; 3 International Potato Center (CIP), Genomics and Biotechnology Program, Nairobi 00603, Kenya; 4 VIB, Institute of Plant Biotechnology Outreach, Technologiepark 3, B-9052 Ghent, Belgium; 5 Ghent University, Department Molecular Biotechnology, Institute of Plant Biotechnology Outreach, Technologiepark 3, B-9052 Ghent, Belgium; 6 Venganza Inc., St. Augustine, FL 32080, United States of America; Kansas State University, UNITED STATES

## Abstract

The African sweetpotato weevil (SPW) *Cylas puncticollis* Boheman is one of the most important constraints of sweetpotato production in Sub-Saharan Africa and yet is largely an uncharacterized insect pest. Here, we report on the transcriptome analysis of SPW generated using an Illumina platform. More than 213 million sequencing reads were obtained and assembled into 89,599 contigs. This assembly was followed by a gene ontology annotation. Subsequently, a transcriptome search showed that the necessary RNAi components relevant to the three major RNAi pathways, were found to be expressed in SPW. To address the functionality of the RNAi mechanism in this species, dsRNA was injected into second instar larvae targeting *laccase2*, a gene which encodes an enzyme involved in the sclerotization of insect exoskeleton. The body of treated insects showed inhibition of sclerotization, leading eventually to death. Quantitative Real Time PCR (qPCR) confirmed this phenotype to be the result of gene silencing. Together, our results provide valuable sequence data on this important insect pest and demonstrate that a functional RNAi pathway with a strong and systemic effect is present in SPW and can further be explored as a new strategy for controlling this important pest.

## Introduction

Sweetpotato *Ipomoea batatas* (L.) Lam. is an important food security crop in Sub-Saharan Africa (SSA), covering around 1.8 million hectares with an estimated production of 11.3 million tons [1]. As this crop is highly adaptable to areas with seasonal rainfalls or long drought periods, it improves consumers’ livelihoods and fulfills their daily food needs particularly for subsistence farmers [2, 3]. Sweetpotato production can be devastated by the infestation of two African sweetpotato weevils (SPW) of which *Cylas puncticollis* Boheman is one [4], resulting in total crop loss especially during periods of pronounced droughts [5]. The primary cause of damage in sweetpotato is the SPW larvae, which tunnel and feed through vines and storage roots. As a result, plants wilt or even die whereas storage roots are reduced in size and number [6]. Furthermore, roots develop a bitter taste due to the presence of terpenoid compounds in response to microbial infection generated by the weevil tunneling. This damage reduces the quality of storage roots for human consumption and causes significant economic losses [7]. Historically, conventional breeding has been applied to develop weevil-resistant plants but the lack of varieties with high level of resistance against SPW [8] together with the complex genetic nature of sweetpotato make it difficult to develop these varieties [3, 9]. In addition, the use of insecticides and diverse techniques of integrated pest management (IPM) have also been ineffective in SSA because of the mode of growth of SPW [10, 11]. Therefore, there is a high need to use other strategies to control SPW which have been proven effective to control other pests for other crops [3].

RNA interference (RNAi) can be a powerful biological tool to achieve sweetpotato resistance against SPW as achieved for other coleopteran pest [12]. This relatively new technique, which triggers gene silencing typically by double-stranded RNA (dsRNA), has become a significant tool to knockdown target genes in plants as well as in insects. To induce an RNAi response in the insect, dsRNA can be delivered into the body through different methods: ingestion, soaking and microinjection. The latter is more frequently used in the laboratory because of the effective delivery of a known dose into the insect, whereas uptake by ingestion or soaking is more appropriate for screening of target genes for future control strategies [13]. After introduction into the cell, dsRNA is recognized as foreign by an RNaseIII nuclease called Dicer and processed into small interfering RNAs (siRNAs). One strand of the siRNA, the “guide strand” is assembled into an RNA-induced silencing complex (RISC) in conjunction with the Argonaute multi-domain protein, which is responsible for target recognition and degradation [14, 15]. At the post-transcriptional level, this complex binds to mRNA complementary to the siRNAs and the mRNA is degraded enzymatically, reducing the amount of mRNA available for protein translation.

In eukaryotes, three main RNAi pathways have been described: microRNAs (miRNAs), small interfering (siRNAs) and Piwi-interacting RNAs (piRNAs) [16], which differ in their biogenesis, type of Argonaute family proteins, mode of target regulation and substrates [17]. The RNAi machinery involved is evolutionarily conserved in most eukaryotic organisms, including insects [18]. In addition, the high sequence specificity of RNAi results in minimal, if any, effects on non-target organisms, including beneficial insects [19]. To date, the potential for RNAi in pest control has been successfully demonstrated for different insect groups [20]. Fourteen essential genes were down-regulated in the coleopteran species *Diabrotica virgifera virgifera* after feeding on an artificial diet containing dsRNA, resulting in very high mortality of the target species [20]. Another Coleopteran insect pest, the red flour beetle *Tribolium castaneum*, also exhibits a very strong RNAi response, including systemic RNAi and a long lasting effect [21, 22]. The evolutionary conservation within eukaryotic organisms and the successful application to control other Coleoptera pests suggest this approach might also be successful against the Coleopteran SPW. However, even within insect groups a high variability of RNAi response has been observed [23]. In fact, RNAi efficacy varies among insect species, genes, mode of dsRNA delivery, dsRNA uptake, spread of silencing signal and life stage [24, 25].

The RNAi response in SPW is uncertain. Therefore, it is necessary to identify the presence of the RNAi machinery in SPW and to determine its functionality. As no substantial gene information was available for *C. puncticollis* prior to this study, we sequenced its transcriptome using an Illumina platform, which has been used in transcriptome analysis of many other species [26–28]. After annotation using reference insect sequence databases, the genes involved in the RNAi machinery were searched for. In addition, the present study aimed to demonstrate the functionality of the RNAi pathway in *C. puncticollis* by applying dsRNA nanoinjection targeting *laccase2*, a gene involved in the insect cuticle tanning [29], which is expected to provide a rapid and clear phenotypic evidence for gene silencing. Effective downregulation of *laccase2* can indicate the potential of *C. puncticollis* to initiate a systemic RNAi response.

## Material and Methods

### Sweetpotato weevil rearing

A SPW colony was maintained in plastic cages at standard laboratory conditions of 27°C, 65% RH under a 16:8 light:dark regime. Insects were kept for feeding and oviposition on sweetpotato storage roots. Fresh storage roots were added every 3 days in order to obtain second instar larvae for nanoinjection. Larvae were removed from the roots at 7–9 days after oviposition.

### cDNA libraries and Illumina sequencing for transcriptome analysis

Total RNA was extracted from second instar larvae of *C. puncticollis* using the RNeasy Mini Kit (Qiagen). The cDNA library preparation and Illumina sequencing were conducted at the North Carolina State University Genomic Sciences Laboratory. The RNA quality and concentration were examined on the Agilent 2100 Bioanalyzer using a RNA Pico Chip. One microgram of total RNA was used following the requirements of TruSeq RNA sample preparation v2 protocol (Illumina). Total RNA was purified using oligo (dT) magnetic beads to isolate poly-A containing mRNA and fragmented into short sequences using divalent cations. The purified mRNA fraction was then used for synthesis of first and second strand cDNA. After the end repair on the double-stranded cDNA, 3’ ends were adenylated and adapters with indexes were ligated for multiplexing. The cDNA library was amplified by PCR and then AmpureXP beads were used for purification. The final library was quantified using Agilent’s Bioanalyzer High Sensitivity DNA Chip prior to clustering on the Illumina cBot. The cDNA libraries were sequenced on the Illumina sequencing platform (HiSeq2000) where each sample was collocated in one lane of a 100bp single-end run.

The Trinity software (http://trinityrnaseq.sourceforge.net/) was used for *de novo* assembly of the raw reads to generate a set of contigs. The software used a Bruijn graph algorithm and a k-mer length of 25. The generated dataset was assembled independently under three different conditions: A full assembly of all reads, an assembly of a reduced representation of the reads, and an assembly following computational normalization of the reads in the dataset via the Trinity In Silico read normalization tool.

### Homology search and gene ontology annotation

The generated contigs were analyzed by searching the non-redundant (nr) insect protein database at the National Center of Biotechnology Information (NCBI) with the BLASTX algorithm (http://www.ncbi.nlm.gov), using a cut-off bitscore >50. For gene ontology (GO) annotation, a second homology search was performed to annotate the generated contigs by searching the Swiss-Prot database with the BLASTX algorithm from NCBI database using a cut-off bitscore >50. The generated gene identifiers were used as input in QuickGo from EBI (http://www.ebi.ac.uk/QuickGO/GAnnotation) and to calculate GO terms.

### Sequence submission

All raw reads have been deposited in the sequence reads archive (SRA) at NCBI, and could be accessed using SRX732288 accession number.

### RNAi-related genes

A list of RNAi-related genes employed by Swevers *et al*. [30] was selected, covering the RNAi core machinery (Table 1), auxiliary factors (Table 2) nucleases, antiviral RNAi and dsRNA uptake (Table 3) (Accession numbers are listed in Tables 1, 2 and 3). Homologous sequences from *T. castaneum* corresponding to these genes were used as a query to search the transcriptome from *C. puncticollis* for the presence of RNAi-related genes using the BLAST tool (http://brcclusterrac.statgen.ncsu.edu/Niblet/). The contigs obtained from the search with bitscore >150 were used for further analysis to verify their identity.

**Table 1:** Overview of identified genes related to the RNAi pathways in *C. puncticollis*.

	**Contig**	**First hit BLASTp**	**Tribolium homologue**	**Comparison to Tribolium**
**miRNA**				
Dcr-1	Cp.comp36004_c0_seq2	hypothetical protein YQE_09128, partial [*Dendroctonus ponderosae*]	EFA11550	E = 0.0; bits = 1971
Ago1	Cp.comp34373_c0_seq6	argonaute1 [*Tribolium castaneum*]	EFA09197	E = 0.0; bits = 1765
Loquacious	Cp.comp35585_c0_seq1	PREDICTED: similar to tar RNA binding protein; [*Tribolium castaneum*]	XP_966668	E = 0.0; bits = 545
Drosha	Cp.comp39990_c0_seq1	PREDICTED: similar to ribonuclease III *[Tribolium castaneum*]	XP_967454	E = 0.0; bits = 1684
Pasha	Cp.comp38940_c0_seq1	hypothetical protein YQE_10523, partial; [*Dendroctonus ponderosae*]	XP_971282	E = 0.0; bits = 786
Exportin-5	Cp.comp39084_c0_seq1	hypothetical protein YQE_01298, partial [*Dendroctonus ponderosae*]	XP_974696	E = 0.0; bits = 1316
**siRNA**				
Dcr2	Cp.comp37119_c0_seq1	hypothetical protein D910_09530, partial [*Dendroctonus ponderosae*]	NP_001107840	E = 0.0; bits = 1012
Ago-2	Cp.comp38067_c0_seq1	hypothetical protein D910_08685 [*Dendroctonus ponderosae*]	EFA04626	E = 0.0; bits = 988
R2D2	Cp.comp37256_c0_seq4	hypothetical protein YQE_06343, partial [*Dendroctonus ponderosae*]	NP_001128425	E = 1e-83; bits = 266
**piRNA**				
AGO3	Cp.comp32215_c0_seq1	hypothetical protein YQE_10018, partial [*Dendroctonus ponderosae*]	EFA02921	E = 0.0; bits = 1003
PIWI	Cp.comp31984_c0_seq1	piwi [*Tribolium castaneum*]	EFA07425	E = 0.0; bits = 989
Aubergine	Cp.comp31984_c0_seq1	piwi [*Tribolium castaneum*]	XP_001811159	E = 0.0; bits = 975
Zucchini	Cp.comp38142_c1_seq5	hypothetical protein TcasGA2_TC010319 [*Tribolium castaneum*]	EFA13216	E = 1e-46; bits = 166
Zucchini	Cp.comp38489_c0_seq8	hypothetical protein YQE_07414, partial [*Dendroctonus ponderosae*]	EEZ99465	E = 1e-50; bits = 176

(FS) frame shift; (RF) reading frame.

**Table 2:** Overview of identified genes associated to RISC complex in *C. puncticollis.* (FS) frame shift; (RF) reading frame.

	**Contig**	**First hit BLASTp**	**Tribolium homologue**	**Comparison to Tribolium**
**RISC**				
Tudor-SN	Cp.comp40322_c1_seq1	hypothetical protein YQE_11841, partial [*Dendroctonus ponderosae*]	XP_974879	E = 0.0; bits = 735
Vasa intronic gene (VIG)	Cp.comp31480_c0_seq2	hypothetical protein D910_11911 [*Dendroctonus ponderosae*]	EFA12812	E = 9e-96; bits = 301
Similar to fragile X mental retardation syndrome related protein 1 (FXMR1)	Cp.comp38219_c0_seq6	hypothetical protein D910_07822, partial [*Dendroctonus ponderosae*]	XP_969396	E = 0.0; bits = 730
p68 RNA helicase	Cp.comp36480_c0_seq1	hypothetical protein YQE_12421, partial [*Dendroctonus ponderosae*]	NP_001164095	E = 0.0; bits = 806
Translin	Cp.comp40752_c0_seq1	hypothetical protein YQE_05829, partial [*Dendroctonus ponderosae*]	EFA07522	E = 4e-105; bits = 313
Similar to translin associated factor X	Cp.comp28110_c0_seq1	hypothetical protein D910_08298 [*Dendroctonus ponderosae*]	XP_975473	E = 3e-107; bits = 333
Armitage	Cp.comp39999_c0_seq2	hypothetical protein D910_08795 [*Dendroctonus ponderosae*]	XP_969071	E = 0.0; bits = 1119
Homeless (spindle-E)	Cp.comp40635_c0_seq2	hypothetical protein YQE_03529, partial [*Dendroctonus ponderosae*]	XP_971741	E = 0.0; bits = 1300
Maelstrom	Cp.comp35977_c0_seq3	hypothetical protein D910_02860, partial [*Dendroctonus ponderosae*]	EFA02892	E = 1e-67; bits = 234
HEN1	Cp.comp39152_c0_seq16	hypothetical protein D910_04572, partial [*Dendroctonus ponderosae*]	EEZ98969	E = 2e-145; bits = 462
RNA helicase Belle	Cp.comp37673_c0_seq4	ATP-dependent RNA helicase belle [*Tribolium castaneum*]	NP_001153721	E = 0.0; bits = 1037
PRP16, mut6 homolog	Cp.comp39484_c0_seq1	hypothetical protein D910_03265 [*Dendroctonus ponderosae*]	XP_969616	E = 0.0; bits = 2094
Gemin3 homolog	Cp.comp40453_c0_seq1	hypothetical protein TcasGA2_TC003675 [Tribolium castaneum]	EFA00789	E = 0.0; bits = 551
Similar to Gawky	Cp.comp40223_c1_seq8	hypothetical protein YQE_12796, partial [*Dendroctonus ponderosae*]	XP_973043	E = 0.0; bits = 1212
Staufen	Cp.comp28896_c0_seq2	hypothetical protein YQE_06727, partial [*Dendroctonus ponderosae*]	EFA11564	E = 0.0; bits = 947
Clp1 homolog (kinase)	Cp.comp34766_c0_seq1	PREDICTED: similar to AGAP007701-PA [*Tribolium castaneum*]	EFA06994	E = 0.0; bits = 673
Elp-1	Cp.comp40424_c1_seq1	hypothetical protein D910_02697 [*Dendroctonus ponderosae*]	XP_970736	E = 3e-137; bits = 335
GLD-1 homolog	Cp.comp39799_c0_seq20	held out wings [*Tribolium castaneum*]	NP_001164152	E = 0.0; bits = 649
ACO-1 homolog	Cp.comp40176_c0_seq1	PREDICTED: similar to aconitase [*Tribolium castaneum*]	XP_972101	E = 0.0; bits = 1544

**Table 3:** Overview of identified genes associated to RNAi in *C. puncticollis.* (FS) frame shift; (RF) reading frame.

	**Contig**	**First hit BLASTp**	**Tribolium homologue**	**Comparison to Tribolium**
**dsRNA uptake**				
Scavenger receptor SR-C-like protein	Cp.comp35050_c0_seq1	PREDICTED: similar to scavenger receptor SR-C-like protein [*Tribolium castaneum*]	XP_001812043	E = 4e-150; bits = 455
Eater	Cp.comp38230_c1_seq1	hypothetical protein D910_03817 [*Dendroctonus ponderosae*]	XP_969372	E = 1e-43; bits = 171
SID1-related C precursor	Cp.comp38247_c0_seq1	hypothetical protein D910_05186 [*Dendroctonus ponderosae]*	NP_001099128	E = 0.0; bits = 963
SID1-related C precursor	Cp.comp38991_c0_seq20	hypothetical protein D910_05186 [*Dendroctonus ponderosae*]	NP_001099128	E = 5e-143; bits = 449
FBX011	Cp.comp39489_c1_seq2	hypothetical protein D910_09724 [*Dendroctonus ponderosae*]	EFA07112	E = 0.0; bits = 1677
CG4966 = orthologous to the Hermansky-Pudlak Syndrome4	Cp.comp40396_c0_seq2	hypothetical protein TcasGA2_TC002372 [*Tribolium castaneum*]	XP_969589	E = 0.0; bits = 632
**Antiviral**				
Ars2	Cp.comp36546_c1_seq2	hypothetical protein YQE_07634, partial [*Dendroctonus ponderosae*]	EFA00685	E = 0.0; bits = 625
CG4572	Cp.comp36338_c0_seq1	PREDICTED: similar to salivary/fat body serine carboxypeptidase [*Tribolium castaneum*]	XP_969249	E = 0.0; bits = 717
Egghead	Cp.comp38318_c0_seq1	PREDICTED: similar to conserved hypothetical protein [*Tribolium castaneum*]	XP_975496	E = 0.0; bits = 731
ninaC	Cp.comp38683_c0_seq2	Neither inactivation nor afterpotential protein C [*Acromyrmex echinatior*]	XP_968286	E = 0.0; bits = 1112
**Nucleases**				
Snipper = Eri1	Cp.comp37539_c0_seq1	Snipper [*Tribolium castaneum*]	NP_001107798	E = 1e-91; bits = 281
Nibbler	Cp.comp36632_c0_seq4	hypothetical protein TcasGA2 TC002596 [*Tribolium castaneum*]	EEZ99816	E = 0.0; 952bits
Sdn1-like	Cp.comp36451_c0_seq1	hypothetical protein D910_06808 [*Dendroctonus ponderosae*]	EFA00159	E = 0.0; bits = 942
dsRNAse	Cp.comp35928_c0_seq3	hypothetical protein YQE_04599, partial [*Dendroctonus ponderosae*]	XP_970494	E = 7e-119; bits = 363
Exosome	Cp.comp37176_c0_seq1	PREDICTED: similar to Rrp6 CG7292-PB [*Tribolium castaneum*]	XP_966807	E = 0.0; bits = 711
Poly(A) polymerase	Cp.comp37990_c0_seq4	hypothetical protein YQE_12311, partial [*Dendroctonus ponderosae*]	EFA00912	E = 0.0; bits = 862

The program ORF Finder from NCBI was used to detect open reading frames. Homologous proteins were searched with the Protein Basic Local Alignment Tool (Protein BLAST) against the non-redundant protein database at NCBI. Upon indication of the presence of frame shifts, sequences were further analyzed with BLASTX against the non-redundant protein database at NCBI.

### dsRNA synthesis and purification

The dsRNAs for *laccase2* (362 bp) and *gfp* (495 bp) were synthesized using the MEGAscript kit (Ambion). The *C. puncticollis* transcriptome was searched for the *laccase2* sequence using the homologous sequence from *T. castaneum* as a query. The fragment was amplified by PCR using cDNA of second-instar *C. puncticollis* larvae as template, prepared with SuperScript First-Strand Synthesis System (Invitrogen). The primers used for the PCR are listed in Table 4. The PCR products were cloned into the pJET1.2/blunt cloning vector (Thermo Scientific). The insertions were confirmed by Sanger sequencing. The dsRNA templates were produced by PCR using DNA plasmids linearized with *Nco*I and primers with a T7 promoter region (TAATACGACTCACTATAGGGAGA) at the 5’ end of each primer (Table 4). The PCR products were purified using the CyclePure E.Z.N.A. kit (Omega Bio-Tek) and immediately used for in vitro transcription using MEGAscript kit (Ambion) according to the manufacturer’s instructions. Nuclease-free water was used for dsRNA elution. The dsRNA synthesis was verified by gel electrophoresis and quantified in a NanoDrop ND-1000 (Thermo Scientific).

**Table 4:** Primers used in PCR for cloning and real-time quantitative PCR.

	**Primer**	**Sequence (5’-3’)**	**Product size (bp)**
Cloning	lac2 -F	GGCACCAACGATTTCTACAC	970
	lac2 -R	TCAACGTGTGTCCTTGAATG	
	dslac2-F	*TAATACGACTCACTATAGGGAGA*TGTACTCCAAACGCAACCAA	362
	dslac2-R	*TAATACGACTCACTATAGGGAGA*ACGATGCTGCCGTAAATACC	
	dsgfp-F	*TAATACGACTCACTATAGGGAGA*TACGGCGTGCAGTGCT	495
	dsgfp-R	*TAATACGACTCACTATAGGGAGA*TGATCGCGCTTCTCG	
qRT-PCR	rpl32-F	TACGTTTCCTCGCAGACACA	205
	rpl32-R	ATCGACAACAGGGTGAGGAG	
	β-actin-F	GAATTGCCTGATGGACAGGT	225
	β-actin-R	CTTCTGCATACGGTCAGCAA	
	lac2-F	ACCACCAAATCTTGACCCCA	102
	lac2-R	AATCTTTCGGCGGCATCTTC	

### Larval injection

Nanoinjection was performed using second-instar larvae of *C. puncticollis*. Larvae were anesthetized with diethyl ether for 5 min and immobilized in an agarose plate at 1.5%. The dsRNA for *laccase2* and *gfp* (control) was injected into the hemocoel at a concentration of 0.2 μg/mg body weight (BW) using a nanoinjector (FemtoJet, Eppendorf) and needles prepared with glass capillary tubes. At least 85 larvae were injected per treatment of which 25 and 60 individuals were used for phenotypic evaluation and real-time quantitative PCR (qPCR), respectively. After injection, larvae were placed into sweetpotato root slices of 1x1 cm in petri dishes and incubated at 27°C and 65% RH. Larvae were evaluated phenotypically every day for 15–20 days.

### Real-time quantitative PCR

Total RNA was extracted from the whole insect body at 1, 3, 5 and 10 days after injection, each time point containing three biological samples of 5 pooled insects. The RNeasy Mini Kit (Qiagen) was used for RNA extraction following the manufacturers’ instructions. After DNaseI treatment (Ambion), RNA was quantified using a NanoDrop ND-1000 (Thermo Scientific) and verified by 1.5% agarose gel electrophoresis. Total RNA (0.9 µg) was reverse transcribed using the SuperScript II kit (Invitrogen) according to manufacturer’s instructions. Real time quantitative PCR was performed in the CFX 96^TM^ real-time system and the CFX manager software (Biorad). The primers used in the analysis (Table 4) were validated with a standard curve based on a serial dilution of cDNA to determine the primer annealing efficiency and a melting curve analysis with temperature range from 60 to 95°C. The reaction included 10 μl of SsoFast^TM^ EvaGreen Supermix (Biorad), 0.4 μl of 10 μM forward primer (Invitrogen), 0.4 μl of 10 μM of reverse primer (Invitrogen), 8.2 μl of nuclease-free water and 1 μl of cDNA, in a total volume of 20 μl. The amplification conditions were 3 min at 95°C followed by 39 cycles of 10 s at 95°C and 30 s at 58°C. The reactions were set-up in 96-well format Microseal PCR plates (Biorad) in triplicates. The endogenous controls, ribosomal protein L32e (rpl32) and β-actin, were used for normalization of the data. Appropriate controls, no-template control and no reverse transcriptase control, were also included in the assay. Relative transcript levels of *laccase2* were normalized to the endogenous reference genes *rpl32* and *β-actin* by the equation ratio 2^-ΔΔCt^ [31]

## Results and Discussion

### Analysis of *Cylas puncticollis* transcriptome

The *C. puncticollis* transcriptome was sequenced to gain insights into the RNAi-related genes and for further exploration of essential genes to be silenced through RNAi technology. Sequencing was performed using an Illumina platform, which generated a total of 213,207,004 reads of 100 bp long, corresponding to an accumulated length of 21,320,700,400 bp. The full dataset was assembled using Trinity software resulting into 89,599 contigs with a mean length of 1,630 bp and an average GC content of 39%.

**Figure 1:**
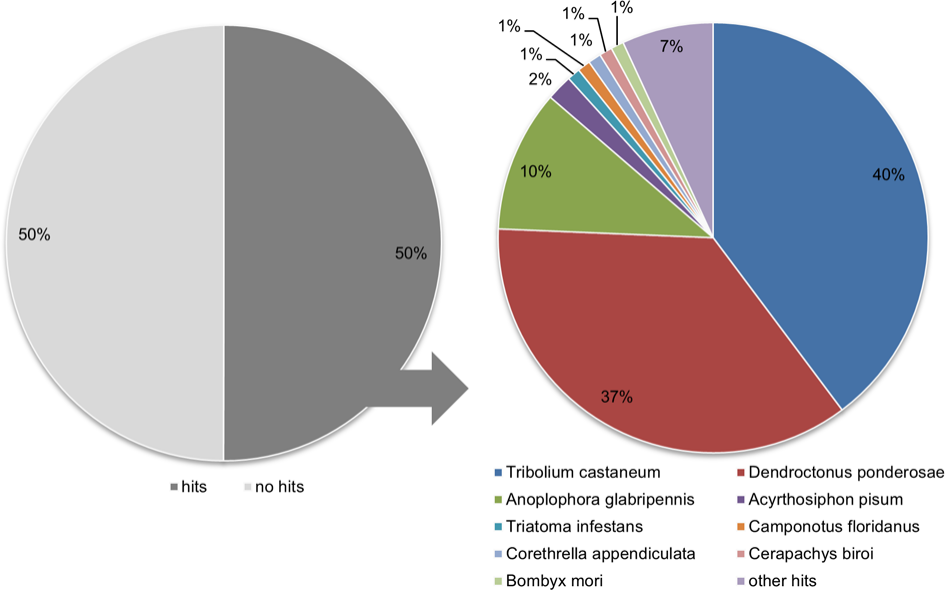
Sequence comparison to other insect genera from the distribution of BLASTX hit (bitscore >50) against the nr protein database of the National Center for Biotechnology Information.

For BLAST annotation, contigs were first searched for similar insect protein sequences using BLASTX against the non-redundant (nr) protein NCBI database, This BLASTX analysis produced 44,824 hits, representing 50.0% of total contigs (Fig. 1). The number of non-significant hits (50.0%) indicates that the *C. puncticollis* transcriptome contains unknown sequences that are not yet described in the insect protein sequences databases. For those sequences with a significant match, 87.68% of the contigs are most similar to sequences from coleopteran species: 40.31% to the red flour beetle *Tribolium castaneum*, which is a worldwide pest of stored food products, 36.51% to the mountain pine beetle *Dendroctonus ponderosae* sequences, which is a serious forest pest [32] and 10.87% to the Asian long-horn beetle *Anoplophora glabripennis* sequences, also found to be destructive of forest trees [33]. The remaining 12.32% of all contigs were more similar to the hemipterans *Acyrthosiphon pisum* (1.78%) and *Triatoma infestans* (0.94%), the hymenopterans *Camponotus floridanus* (0.78%) and *Cerapachys biroi* (0.60%), the dipteran *Corathrella appendiculata* (0.65%), the lepidopteran *Bombyx mori* (0.57%) and others (7.1%).

### Gene ontology classification

**Figure 2:**
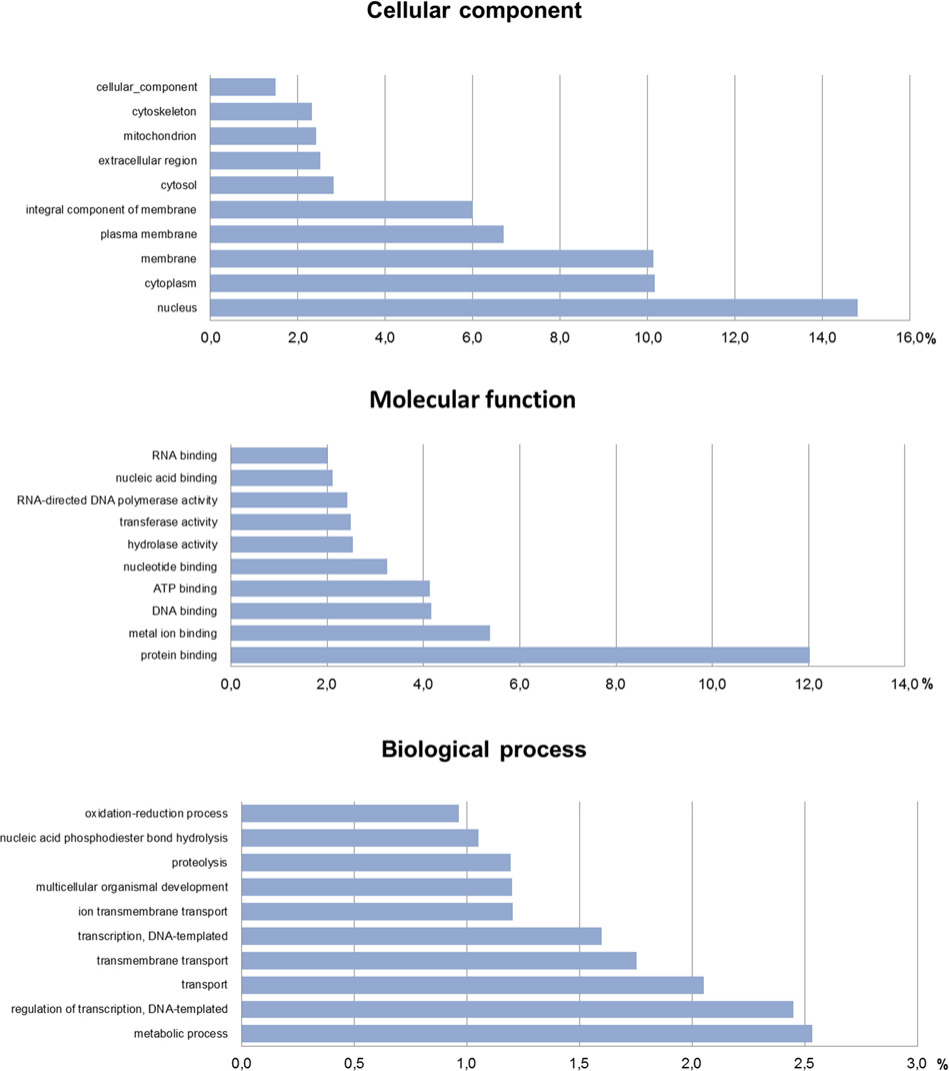
Percentage of *Cylas puncticollis* contigs assigned to a certain gene ontology term as predicted by QuickGO from EBI.

To functionally classify the generated contigs, BLASTX similarity searches were performed against the Swiss-Prot database (bitscore >50), resulting in 36,198 (40.4%) significant hits. The resulting identifiers from this search were used to calculate GO terms, which were grouped into 3 main categories: cellular component, biological process and molecular function. A total of 706,945 predicted GO terms were obtained. The most dominant GO terms within the cellular component were nucleus (29,759; 14.8%), for the biological processes it was metabolic processes (7,607; 2.5%) and for the molecular function it was protein binding (24,774; 12%) (Fig. 2). Similar results were found in the *D. ponderosae* transcriptome, which was the second best hit in the homology search. The most dominant GO term within the biological process was metabolic process as in *C. puncticollis*. However, for the cellular component and molecular function, cell part and binding were the most dominant, respectively [31].

### Identification of RNAi-related genes

To gain insight in the potential of *C. puncticollis* to exhibit an RNAi response, the *C. puncticollis* transcriptome was screened for the presence of the most important genes related to the RNAi machinery. *T. castaneum*, like *C. puncticollis*, belongs to the order of Coleoptera and is more phylogenetically related to *C. puncticollis* than *C. elegans* and *D. melanogaster*. Moreover, the complete genome of *T. castaneum* has also been sequenced and fully annotated [34]. Therefore, homology searches were performed using as reference the *T. castaneum* sequences for the homologous genes listed by [30]. Accordingly, 47 RNAi-related genes from *C. puncticollis* could be annotated. After identification of these contigs, a BLASTp similarity search was performed against the NCBI database to confirm their identity. Sequences of *D. ponderosae* and *T. castaneum* showed closest similarity to *C. puncticollis*.

**Core RNAi machinery.** The core components of the RNAi machinery are proteins that, together with the small RNA fragments, are involved in gene silencing. There are three major pathways studied in eukaryotes: miRNAs, siRNAs and piRNAs [35]. The miRNA and siRNA pathways have an important role in gene regulation by suppressing mRNA translation or inducing mRNA degradation [36]. The difference between the miRNA and siRNA pathways is in their biogenesis, but not in their function. The piRNA pathway has been less characterized and is, in contrast to the two first classes, restricted to germlines [37].

In the miRNA and siRNA pathways, orthologous sequences to the three RNaseIII proteins Drosha, Dicer-1 and Dicer-2, were identified in *C. puncticollis* with a bitscore >150. The main domains of the typical Drosha and Dicer proteins were found to be conserved in *C. puncticollis*. The Dicer domains are: amino-terminal DExH-box helicase domains, PAZ domain, two RNaseIII domains, and carboxi-terminal dsRNA-binding domain (dsRBD). Unlike Dicer, Drosha has no PAZ and amino-terminal DExH-box helicase domain [38]. Three cofactors with conserved domains, Pasha, Loquacious and R2D2, were also identified in *C. puncticollis*. These proteins are required to interact with the RNaseIII genes Drosha, Dicer-1 and Dicer-2, respectively (Table 1, S1 Supporting Information).

Drosha, Dicer1 and Dicer2 are key factors to process dsRNA into small RNAs. Both Dicers were also found in *D. melanogaster*, Dm-Dicer-1 and Dm-Dicer-2, responsible for the miRNA and siRNA pathway, respectively [39]. In *T. castaneum*, Dicer-2 (Tc-Dcr-2) has been found to play an important role in systemic RNAi, while Dicer1 (Tc-Dcr-1) is not involved. In *C. elegans*, a single Dicer was found to govern both pathways [40]. The presence of Dicer-1 and Dicer-2 as well as their cofactors in *C. puncticollis*, suggests that they could have a role in the miRNA and siRNA pathway, respectively.

Another crucial RNAi-related gene is *Argonaute*, which is a component of the RISC complex and is involved in post-transcriptional silencing. A contig containing the main domains (PAZ domain and PIWI domain) usually found in Argonaute (Ago) proteins, is also present in *C. puncticollis* (Table 1, S1 Supporting Information). Five types of Ago were found in *T. castaneum* and *D. melanogaster* and 27 in *C. elegans* [22, 41]. Ago1 and Ago2 are critical in the miRNA and siRNA pathway, respectively [42]. In the present study, we have identified 5 members of the Argonaute protein family: Ago1, Ago2, which belong to Argonaute subfamily and Ago3, Aubergine (Aub) and Piwi, which belong to the Piwi subfamily [43].

Aub and Piwi, as well as Zucchini are proteins involved in the third pathway of piRNA [37]. Searching Aub and Piwi from *T. castaneum* resulted in two protein sequences that matched the same contig in *C. puncticollis*. This result suggests that either Aub or Piwi is present in *C. puncticollis* (Table 1, S1 Supporting Information). For Zucchini, which is an endoribonuclease with a role in piRNA maturation, a 61% of similarity was observed with two analyzed sequences (bitscore >150). Even though the observed similarity for Zucchini was slightly lower than for the other blasted sequences (bitscore >200), the full conserved domain could be identified, suggesting that Zucchini is present in *C. puncticollis*, (Table 1, S1 Supporting Information).

**Auxiliary factors (RISC).** The presence of auxiliary factors to the RNAi machinery was also examined in the *C*. *puncticollis* transcriptome (Table 2, S2 Supporting Information). These included 19 intracellular factors that are associated with (or regulate) the activity of the RISC complex. The protein sequences for Tudor-SN (TSN), Vasa-intronic gene (VIG), fragile X related protein 1 (FXMR1) and p68 RNA helicase, that are present in the holo-RISC complex as found in *D. melanogaster* [44, 45], were identified in *C. puncticollis*, all with conserved main domains.

The two conserved subunits of the C3PO (component 3 promoter of RISC), Translin and Translin-associated factor X, which were characterized to promote RISC activation [46], were also identified in *C. puncticollis*. The nucleases involved in piRNA biogenesis, Armitage (Armi), spindle-E (Spn-E) and Maelstrom, as well as Hen-1 were present in *C. puncticollis* with all conserved domains. Armi, Spn-E and Maelstrom are required for piRNA production and/or stability. Mutation of these genes showed depletion of piRNAs in fly ovaries [47, 48]. Hen-1 is a methyltransferase associated with Piwi proteins in ovaries. This protein methylates small RNAs through a 2’-O-methyl modification at their 3’ ends, playing a critical role in gene silencing suppression. [49, 50].

Full-length fragments were found for the DEAD-box RNA helicases, Belle and PRP16 in *C. puncticollis*. Belle has a function in the endo-siRNA pathway, interacting with Ago2 and endo-siRNA-generating loci and is localized in condensing chromosomes in a *dcr-2-* and *ago2-*dependent manner [51]. PRP16 has an important role in the pre-mRNA splicing [52] with activity in RNAi, and it is a homologous protein to Mut6 in *Chlamydomonas* [53]. For another DEAD-box RNA helicase, Gemin3 homolog [54], a partial fragment is present in *C. puncticollis*, only covering 50% of the full sequence; however the main domains are present.

The proteins Gawky, localized in GW-bodies in *D. melanogaster* and required for miRNA function [55], Staufen (STAU1), a dsRNA-binding protein, and Clp-1, a RNA kinase able to phosphorylate siRNAs, were all present in *C. puncticollis*. Elp-1, a component of the pol II core elongator complex involved in the RNAi silencing, was also identified in *C. puncticollis*. Two fragments covered the full-length sequence of this protein [56]. A full-length sequence is also present for the protein GLD-1 homolog, a KH motif containing RNA-binding protein of the GSG/STAR subfamily, involved in different aspects of germline development. It is known to prevent translation of mRNA into proteins through target mRNA binding [57]. For ACO-1, an RNAi-binding protein involved in translational inhibition, a partial fragment was identified in *C. puncticollis* [58].

**dsRNA uptake.** The proteins sequences for SID1, FBX011, Scavenger receptor SR-C-like protein and Eater were searched in the *C. puncticollis* transcriptome as well (Table 3, S3 Supporting Information). SilC and SilB were found in *C. puncticollis* as a first and second hit, respectively; whereas SilA and SID1 were not. The *sid1* gene in *C. elegans* encodes a multi-transmembrane domain protein, which is essential for uptake of dsRNAs into cells and for the spreading of the RNAi signal in *C. elegans* [59]. Three *sid1*-like genes were found in *T. castaneum* (SilA, SilB and SilC) [60], while in *D. melanogaster* no homologs for Sid proteins were found. Initially, it was thought that the presence or absence of these genes explained the robust and systemic RNAi response in *T. castaneum* and the lack of systemic RNAi in *D. melanogaster*, respectively. However, later research has shown that these Sils in *T. castaneum* are not critical for the systemic RNAi response, as silencing these genes did not affect the systemic RNAi response [58]. Furthermore, other mechanisms, including endocytosis, have been shown to be involved in dsRNA-uptake in certain insects as well [61, 62]. Whether or not these Sils play a role in dsRNA uptake in insects from other orders remains unclear.

FBX011 was found in *C. puncticollis* with a conserved F-box domain and three beta-helices. Scavenger receptors, such as Eater and SR-CI, were found to be important for dsRNA uptake [63]. Scavenger receptors are known to act as receptors for large molecules and/or microbes and play a role in phagocytosis. Eater encodes a Nimrod family protein that contains multiple NIN-type EGF domains. All these protein sequences are present in the *C. puncticollis* transcriptome.

**Antiviral RNAi.** Four protein sequences involved in antiviral RNAi found in *D. melanogaster,* were searched in *C. puncticollis*: Ars2, a regulator of the RISC complex, CG4572, a protein with an unknown function, Egghead (egh), a seven transmembrane-domain glycosyltransferase and ninaC, a protein involved in vesicle transport [64, 65] (Table 3, S4 Supporting Information). In *C. puncticollis*, full-length sequences were identified for CG4572 and ninaC, but only partial fragments for both Ars2 and Egghead proteins.

**Nucleases.** Little is known about the nucleases that interact in RNAi. Six nucleases believed to have RNAi-related activity were found present in the *C. puncticollis* transcriptome: Eri-1 like, Nibbler, Sdn1-like, the homolog of the *B. mori* DNA/RNA non-specific alkaline nuclease, Exosome and Poly(A) polymerase (Table 3, S5 Supporting Information). A full-length sequence of the Eri-1 protein is present in *C. puncticollis*. Eri-1 is an evolutionary conserved protein involved in intracellular siRNA degradation, and of which the SAP/SAF-box domain and DEDDh family exonuclease domain [66] are conserved in *C. puncticollis*. For the small RNA-degrading nuclease Sdn1-like, which has a 3’ to 5’ exonuclease activity, and which can degrade mature miRNAs in plants [67], a full-length sequence with conserved domains was identified. For the nucleases, Nibbler, which processes 3’-ends of miRNAs [68], dsRNAse, a dsRNA-degrading enzyme [69]. Exosome, which has a 3’ to 5’ exonuclease activity [70] and Poly(A) polymerase, which is involved in the mRNA degradation [71], partial sequences with conservation of the main domains also are present in *C. puncticollis*.

In summary, these results revealed the presence of 47 known RNAi-related genes in *C. puncticollis*, which is a first condition for the use of RNAi-based pest control methods for this weevil. Furthermore, our results confirmed the conservation of these RNAi-related genes among coleopteran species as *T. castaneum*, which show a very robust RNAi system [22, 72].

### Silencing of *laccase2* gene

To demonstrate the functionality of the RNAi pathway in *C. puncticollis*, dsRNA targeting *laccase2* was synthetized. This gene is involved in insect cuticle sclerotization and provides a rapid and clear phenotypic evidence for gene silencing in *T. castaneum* [29]. Prior research showed that a high concentration and longer fragments of dsRNA (>300 bp) are critical for an efficient inhibition of *laccase2* expression and longer duration of the RNAi effect [72]. Therefore, we injected a 362 bp-long dsRNA molecule targeting *laccase2* into the hemocoel of second-instar larvae with a final dsRNA concentration of 0.2 µg/mg of body weight. The control group was injected with the same concentration of a 495 bp-long dsRNA molecule targeting the *gfp* gene being absent in *C. puncticollis*.

**Figure 3:**
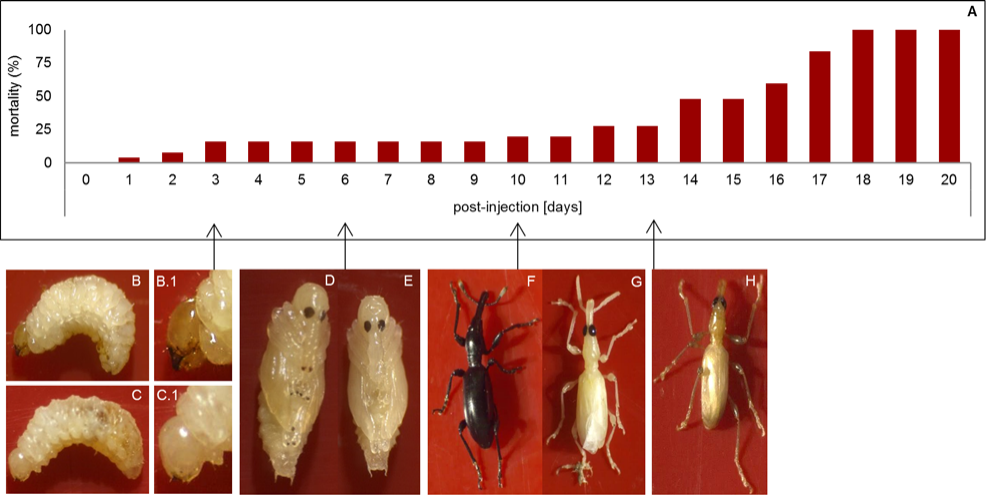
Effect of inhibition of *laccase2* expression after injection with dsRNA in second-instar larvae of *Cylas puncticollis*. (A) Mortality after injection with dsRNA targeting *laccase2* (ds*lac2*) (day 14–20) expressed in percentage. Mortality in larvae injected with dsRNA targeting *gfp* (ds*gfp*) (control) was only 8% (B) Larvae injected with ds*gfp* as a control and (C) treated larvae after 3 days; (D) Pupa development 6 days after injection with ds*gfp* as a control and (E) ds*lac2*; (F) Adult development injected 10 days after injection with ds*gfp* as a control (G) ds*lac2* (H) Surviving individual 13 days after injection with ds*lac2*. Larvae were injected with the dsRNA solution into the hemocoel at a concentration of 0.2 µg/mg body weight. The insects were kept in sweetpotato roots after injection for the duration of the experiment.

**Figure 4:**
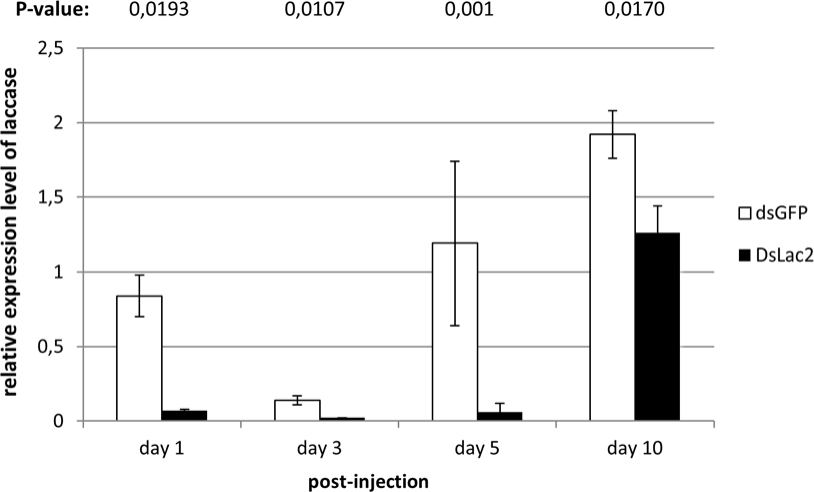
Inhibition of *laccase2* expression in second-instar larvae of *Cylas puncticollis* at 1, 3, 5 and 10 days after injection with dsRNA targeting *laccase2* at 0.2 µg/mg of body weight. Injection with dsRNA targeting *gfp* was used as a control. As internal controls, ribosomal protein L32 and Actin were used. Values are based on two repetitions of three biological samples and expressed as mean ± SEM. Each sample contains 5 pooled insects. The p-values were calculated by unpaired t-test.

Inhibition of *laccase2* expression could be observed phenotypically in 21 of 25 (84%) individuals as early as 3 days following injection. Treated larvae exhibited lack of sclerotization in the head capsule resulting in an untanned cuticle (Fig. 3C.1) compared to the control larvae injected with *gfp* dsRNA (Fig. 3B.1). Injection trauma in the control resulted in 8% of mortality. Suppression of *laccase2* expression can be detected after 24 h when tested by qPCR (Fig. 4). These results demonstrate that *laccase2* mRNA levels were reduced 91.7% compared to the control injected with *gfp* dsRNA (p-value 0.0193) after 24 h. This reduction was also observed at the other two time points, where the expression levels on day 3 and day 5 showed a reduction of 92.9% (p-value 0.0107) and 93% (p-value 0.001), respectively. Interestingly, expression of *laccase2* was found to be variable between different larval stages and even within a certain stage. Possibly, *laccase2* expression only exhibits a peak at a certain time after the molt, given its role in the cuticle tanning. However, further studies should be conducted in order to confirm this. Nevertheless, despite this natural variability, the silencing we observed was strong when compared to the control for each time point. and consistent in all experiments and repetitions.

At 5–6 days post-injection, the pupal stages showed no tanning in cuticular structures as pronotum, prothoracic, mesothoracic and metathoracic legs, elytral and wing sheath and urogomphi (Fig. 3E) whereas an initiation of tanning for these structures was obvious in the control (Fig. 3D). In adults, a complete inhibition of the cuticular sclerotization in the exoskeleton was observed (Fig. 3G) compared to the control (Fig. 3F). Moreover, they exhibited a malformed and soft cuticle with no pigmentation, which complicated their normal mobilization. Additionally, a partial recovery of the cuticle tanning of adults was observed at 13 days post-injection (Fig. 3H). On transcript level, the gradual recovery could already be shown after 10 days, where a smaller difference (34%) in transcript levels between control and treatment could be observed (p-value 0.0170) (Fig. 4). The evaluation of treated insects at 15 to 20 days post-injection showed no survival of adults (Fig. 3A), which could be due to the difficulty of feeding as a result of the malformed and soft cuticle in the mouthparts.

These results clearly demonstrate that an RNAi response is activated in *C. puncticollis* to *laccase2*; furthermore, the lack of cuticle tanning suggests that the RNAi activity is systemic with a persistence of the RNAi signal for at least 10 days. Similar results were also demonstrated by [73], who determined that RNAi in *T. castaneum* is systemic by injecting dsRNA targeting Tc-achaete-scute in larvae. Our results demonstrate that targeting *C. puncticollis* using RNAi as a pest control agent has a clear potential, given the strong and systemic RNAi effect shown here.

## Conclusions

Our data demonstrate that the necessary components of the three major RNAi-related pathways described in insects are present and expressed in *C. puncticollis*. The presence of the core RNAi machinery genes in the transcriptome indicates the potential to initiate an RNAi response in this weevil. Direct injection of dsRNA targeting *laccase2* into the larvae efficiently downregulated gene expression, occurring after 24 h and lasting for at least 10 days after a single injection. This result demonstrated that *C. puncticollis* exhibits a strong and systemic RNAi effect, suggesting the potential of RNAi as a future strategy to control SPW. Furthermore, our research provides valuable sequence data on this important pest insect that will be useful for further research on this economically important weevil.

## Supporting Information

S1 Supporting Information Sequences of *C. puncticollis* core machinery proteins. (DOCX)

S2 Supporting Information Sequences of *C. puncticollis* RISC-associated auxiliary factors. (DOCX)

S3 Supporting Information Sequences of *C. puncticollis* proteins involved in dsRNA uptake. (DOCX)

S4 Supporting Information Sequences of *C. puncticollis* proteins involved in antiviral RNAi. (DOCX)

S5 Supporting Information Sequences of *C. puncticollis* nucleases. (DOCX)
