# Supplementary material for: Transcriptome Analysis and Systemic RNAi Response in the African Sweetpotato Weevil (Cylas puncticollis, Coleoptera, Brentidae)
Source: PLoS One. 2015 Jan 15;10(1):e0115336. doi: 10.1371/journal.pone.0115336 (PMC4295849; doi:10.1371/journal.pone.0115336)

**Supporting information S1:** Sequences of *C. puncticollis* core machinery proteins.

**Dicer-1**

>Cp.comp36004_c0_seq2 len=7412

GTTGAAATGAGTAAACAAATCTGGAAAAAAAAATCAAACACATACCTACGACTAACTTATTAAAAAATATTTAAAGTGATATCACAAGCTTCTGCCACTTATCACATATAAAATACAACTGAATTAGAGTCCCTCTAGTTAAAATAAACTTTGTACTATGCTAATGAATAAAAAACGAGAACAAAACATCAATGAATAAAATAATTGACATAGTAGTGGTTTAGCAATATGCAAAAGGAATGTAAAGAAGATAATTACAACATCCAAGTTGCATATTGTAGAGAGTAACATGTGTAAATTTGTTTGCCTAGCTACGGACACAACAAGATGATCATATAAAATAAGTGTTACACTACCTGCATATAGACTATAGTAATATACACTTTGTGCATTAATGACGAAAGTGGAGCATCTCAGTCTCACAGCAACATTCTATAAGATATATAGAATTGTCGTCGTCTTAGTTGAGTTGCGCCCTGGGTTTGAAGGGCGCGGATAAACCATCCTTGTATTTCTTTTATAGAAGCAGAGTCATCACTTTTCTAATTCACAGCCTTGCTATAAATATTTATTTTTTGCTTCCGGAAAAATTCTGTGTTTAGTTTTGGTTAAAGCTTTCAATAATTTGCTACTTTCAACTTCATAACAGTGGAGAGTCAAATTATTATAAATATATTTAGAGTATATAATACTTTATATTAATTACAATATTTCTTGGTATTTCAATTGCACAAAGTTTTAAATAAGACATCAAATAGTTCAATATACTTTTCATATGTTTCGTGAAATCCGCACTTTTCCACAATAGGAATTTTCCTCAGGCAACACTAATTAAATTTGATGAGATGAGATGTCTGATTTTTCAAACTCAAATATTTCTAGTCAATTTAGGTCATAAACTGAGGAAATTTCTCCAAGAGCTTCAAAATGATGATATGGCTCGTGACTTGATTAAATTTGAGGAAAATTTACTATTGAGGAATGTTCGCATGCGTTTTACTTATTATTTATTTTTATACTCTAGTTAATACTGATTTTTCATTAGAATTCTCTTGCTGAAAACAAGTTAATAAATATTTAATGTAAAAAAACTACTTCGACATTTATATTACAAATTTAAACATATTCGTAAGTTAATATTTTTATTTAATATTGAACTAAGCTACCAAATATTTTAGTATTTAGCTAAAAGTTATATGTGGACATAAACACAAACGTTAGGGGCACAATTGACTAGAAAAAAGAACATCATCAAAGACCAGGCGAGTGAATAAAGTATTCTTGGTTTTTAACATAACTTTTTCAAATAGGAACGCGCCTGGTCGATTAAAAAGTGACTTGAGATCATTATTATGACTTCTAATATTTCGATTTGAAGGGAATTGGATATATGCGAAAAGAGAAATCTCTCTTTTCGCATATTTCTATTCTAATCCCAAATGCAAAAAAAAAAGAAATTTCTGTAATAAAAATTATGTTTCTCGACACTCTTGTTTGACTTGTTTTTGGAATGCACCTTTCAGTTATTAAGTAATCTAAAATGACGGTTGACTTCCATTTGTACAAATATTATAATATGTGCGCTTCTTCACATTTAGCACAATTATGGCACTTTTTTTAATTATCATTTTTATTTCTGAGCAATCCTCTCCTTTTGAGATGTTTTAAAGCACATTTGGCAGCAGTACATTTGGCAATTCTGTAGTTCCTCCCGATACCTTTAAATGTTCCCTTTCCAAATACTTGGACTGTTACTCTCACTCGTCTACCATCTGCTAATTTTTCAGGTCGTCCGAACTTAGCAGTCTCTGGTTCCAATTCGAGAAGCTCTCTAATAGGTGATTTCGGTACTTTGTTAGAGAATTGTTCTATCTCGGTTTTCATCATTCGATGATAAACCTTCCATACAGCGTCCAATGACATTCCTGAATCGAGGTAAATAGCTCCAGCGACTGATTCAAAAACGTCACCTAATGCTTTTGGCACTTCAACATCCTCTACTTCCTCGCATTCAGTATCAACAACGAGATAATGCTCGTCGACAATAGTATGACCACTTTCTTCTTGAAGTCGGACAAATCTTTCAATAACCTCATTTAAGCCGGGCGAAAGATGTTTAAAATATTTATGGAATCCATGTTTTACAGCCAAAGAAGCAAAAATTGTATTGTTAACTAGAGCGGATCTCAAATCAGTTAATGCTCCTGGAGAATGCATTCTAGAATCTTCAAAGAGAGCTTTTGTTATAATAAAATCTAACAAAGCATCTCCTAAAAATTCCAATCTTTGATAGCAGTCTGTTAATCGGTTTGGGGAGTAGGAAGCATGTGTAAAAGCTTGCAACAAATAGGAACGGTCCCTAAATTTATATCCGATATGCTTCTCAAATGTGTCATAACCATCTAATAGCTTCTCTAATTCTTCTTCAGGGTTTGGTATGTTTCTTAGCAGAGGAGATTTTGGAAGATCCAACTCCCCATAACTTCCGTCCTCATTTTTCGGTAGAACCTTAATTCCGAGCCAAGCCATGAACAGCAAAGCACCTCTAGGTCCACATTCAATCAAATAAGCACCGATTAAAGCTTCAACACAATCTGCTATACTTTTGTCAGGTATACTGTGTTGAGTAACCAAATTATAAGGGATGATATTTGGAAGTGAATAAATCCCCCTTTCTCTTACTAAAGTACATACTTCATCAATGGTCATATTTCTAGTGGCAGCCATATCAGCTACTGTCCAACAATTGGCGGGAAACTGTGCATCAATAAGAGCATCTTCGAGTTGTTTCGGAACATAGAAGCAGGGAGGAAGCCAATTATCATGAGGATCGAATTTGGTGGCGACCATATATTCTGCCAAATTTTTAAGTTTGCCCAATTTGTATAGATTTAAATTACTTACATGTTTCGACCTTAAATGGCTTAGTTTACCTTCGTGGATATTTTCGTAAGTTTTGTAAAGATATGTGGTTATTGCATATTTCAGGAACGAATCACCGACCGTTTCTTGCCTTTCCAGGTTAATTCCATCATTAGCATTAGACATTGTTAAAGCTTGTAAAAGAACATTTGGACTTGGACCCGGATGAGTATCTAAATTAGGTTGTTCGTCAAAACTAAAGTTCTGTATATGTTCTGATATGTCATAAACATTAACAAACGGTTCTTGTGGAGCAGAAGATGATTGCTCTATATTAATATCTCCAATCATCAGTGAATCAAAATCGAAATTATCTGGACAAACGGGGGGAATATCATCAGCAGGAGTATTCTGAGATTTTTTCTCAAATTCTTCGTTCTCCTTAATCAAAATTCCACTAGATAAAATGTGTTCCTTATTTCTAAGGCAAGCGTTATTGAATTCATGTCTTAGTCTCTCAGTTTCTTGACTATTGTCATCCACCGTCCAAGCATTCACATCTTCTACAAAATATAACTCATCTTCCTTAGAATCATCATCATCTAGTGCTTCAGCTTGATGATCTCCTGTAAATTCAATACGCAATCTACCCCATTCGTGAGTAGATTCCTCAGAGTCGAAATCGGAATTGTCAGACATATCATCTGAGAAATCCTCATCATAAGAGTTGCCATGTAACCAGCTTGTTGGGGAAGCATATCTTACCAAAGCTGTAGAATCTCCTTGACTCGACTGAGCCATCTCATTAGACCAAGTGCCAACTACCCAGTTGTCACCCCCTTTACCTTCATCATTGCCTTCTTCTTCTTCTGCTTCTGATATATCTGCATCTGAATTTACAGGGTCCTCTTCAGAAGCATCTGACACCAGATTGGTTATTTTTAGCTCCGTTGTTTTTTCTCTTAAGGACTTTAATTTCTCCTCTTGTTTTTTCATCTCTTCATCGCGCGATTTCTTCAAAACGTCAGATAAGCTCCATCCGAAGTTTAAAGGTGTCCATTTGAAATCATCGTCAAGTTTAGTTTTCCCTAGATTCAAGTCGGAAGCAACTGTAGTACGTATCTGGTCAGCTAACAAAAGGGCATTGATTCGGTAAAGTACGCAGGGTAGGGCTACAGCTTTTCTCCAAAGAGAGGCCGAAAAAGGATGAATGGCGCAAAGTTCCGGAACGAGAATTTGCTTCTGTTCTAAGTTTTCTCTTTTGGCTCTTTTTGTTTCTTCGCTACTTGTAGGTAAAGCAACACCCTTTCTGTTTACGTATCTCGGGGTAAGAAAGTTCAGACGGGCCGAAGTGTGATCCACGTCCAACAAGTATTGTTCCAAATTCTGAATTTGAATAGCGTATTTAATGAGATAGTATTGCTCAAATGATTTATATTCTGAGCCAGGAAAAGCGGAAGTTGGATTTAAAAACGAGCAAATTTCTGCCACATAAAAATATTGCGGTTGGTCTTGATTGCGATACCAAGGCATAACCACAGCATCTTTATAATCATCAGTGTTAAATACGTAATCTTTTCTATCCTCGTCTGGTATTACTCTTGGTACAAAGTGTAAATTTTTGTAAATAATGTCTATGAATTCCCAATCCACATCAATGCCATGTTCACTTTGAATTGTCGGTACTATTAGATAATTCTTATCAGATAACTCGGGTTTAAAAAGCATCAAATATTTCTGTAGTCTGAGCACAGTAGTAAAAGTGTAGTTTACAAATTCTTGGATTTTATGAGTCTGCTCCTCATTGACAATGACGTCTTTGGCGCACAGCCGAAGTTCAACTTCAACTTCGCCAGACCTTGTAAATATGGGGAAGGCACTTATCTTTGGTATTTCTCTATTTGTTAATATGCCAAAGCCTTGAGGAGATTCTTCTGGTGGGTAAATTTTTCTACCCCTGGTGTTCTGCTCCTCAGGGAGAGGACACGTTAACGTCATTACGATTTGGTAAAAATAAGTCGGCTGGCCAATAACTGGATGGCAGTGTAACAACGCGTCAGCAACTTTTTTATAATAATATTGTCTTCGTTTCGTAGTACCTGGTCTAACTTCAGAATTTTCATCATATTGTTCATCAAGTGTAATATTATTCCAATCTTCTCCGGCGGGAACGAAATTCTCCTTATTAATAGGTTGCAACTGGTCATCCAATTCGCCGACTTTATGCAAATACTGGCACAACATAAATGCTGCCGCTCTTCTGGACAACAATGAATTTGGCATCAGAGGACTAGTTACAACTTGTTTTACTGGTGAATTAATCGGAAGTCTTATATGGCACACGTAACTGTCACCTACTTTTTCCTCATGCCAAATTGGCGTTAAGCGGGTAAAAGTATCACTAGGAAGCTTCGCGCAGTACTTATTAATAAGTGCTATAGAGTTAAACAAAGTTACACTGGGTGACCCTTCCTCTTTAATTGGCTGATATGGTTTACAACATTTTGAAAAGCTATCAGCTAATTGCTCTTCTGTTTTGTCAGGTTCTAGACTTGAACAGCGCCTTAACAATGTGTTTTCGACTTCGTTATATTCGGCCAAACTTGTAACAAAACTATCAATTTCATCATCGTTGGCAAATAAAATGTAATGTGCCTCAGATGCTCTTGCTCTAGCTTTACACTGAATGTAACTGTGGAATGTTTTGGGTAAATCGAATCTTATGACAAGATTGCACTTTGGTAAATCACAGCCTTGTTCTAGTACCGATGTCGAAATCAAAATGTTACATTCGTGACAACGGAACTTTCGTAATACCTCTTCTTGTATTTTGTGTTCATGTTCAGCTTCTCGTGGCTCCTTTATAATATCTGCCACTTTTTCCACAGAAAATAAAGCAGATATCCACCAAAAGTCGTCGTCACATTGAGATAGAGTACATAAAAGTCCAAATAACGCTTTGGCTTTGTATCGGTTTTCGACGAAAATTAGTGCACAAAGCATGTCTTCAAATTGTGGCCGCTGTTGAAATCTTTTTTTGTCCTTTGCCCTTCGTCTTCTTAAAACTCGAGAGATTAACATCAGGGTCAGTAGATTTTTCCTCTTCGACGGGTTTTGGCTTCTCCCCAGCAGGTTTAAATTGTCGCAAAACGTTAATCAATTTTAATACTTTAGAGGTGGAGTACTGTTTAATTCTTTCCACTTCGTTATTTAGTTTTTCAAATTCGTAATCACATACAGCTCTAATTCGAACTAAAGTGGTTGATACAGTGCACAGTAGAAGATAGTGTCTTTCGTATGGTGTTTTTACTTTTAATTTTTCTATTTTCGAAAGCAAATTCATTGCTGCCCGATCAGCGCCCCATGGACCTAAGTCTTCAAGAATAGACAAATAATCATTTAACAATTCCAAAGGTGTTATTTTTGGATCCGGAATATCTTTATATTCCTCTAAAAACTCATCTTCATAAATTTCACTCGGGTCATATCTATGGTCTTTTATAAATTCAATGCAATTTTGTACAATATCTATTAACTCTTCTTGAAGAGTAAAGGAGTTTAATCTAGAACACTGAATAATGGATTCACGAGGATGGCACGATAAACGAATCAGTGTTACTATTTCGCTGGACGTGTCTACTGTAGCATTAAGTAGTTTTTCTAACCTTTGTAATTCTGCTTCCAATCTCTCAGGTTGTAGTTCTGAGCCTAGAAGTCCAGTTGTTAGTCCTAGAATTCGGGGTTGTTCTTCAGGAGTTAAATCTTGATATTTACTCATGATTTGTTTAATCATATATTGTCTCTTTCCATATAAACAATCATCTATTACAAGCAAGTTATATGATTTTATATCTAATAGAGTATTGCTGTTAATACATATTTCGGCTGTTGTAATGATTACTTGATTGTCACAAAAGTGCTTATTAATATCTCCAGAAGTTTCTGTATCGATACTAACTATAGTTAAATCAGTCAAAAATTTCATGTGGGATGCCATTACAGGAACATTTTGTGGATCTAAAATAAGCAAAGCTTTCTTTTTCAGATTTCGTCGCATTTGCCACGACAGCTCTTGCAGTAATTTAACTACAATAAATGCCTTCGAAGAACTAGTACTGGAACACACTATGGTATTTCGTTTTTTCGCCGAGTCTAGAAGTTCGACCTGATACTCCCTGGGTGTGAATGTATGAGTATATACATTTTCATTTATATAACAAGCCATTTTCAAAGGGGGATGTTAAATTGAGAAGACCGAAATCAGGTTAGAATTTGTTTAAATGTTTTGTAGGTTAGGACATTTTTTATCTCATTTTACAGTACCTTATGCGACTTATCACTAAGTAAAAGTGTTTAATTTCACATGTAATTTTACACAAATCATCTATTTATTTTGTTGTGACCTTTTTTTTAATACGAAATTTGCGGACATTTTTACCACAGAACGAGGCACAGTACAAGATAAACCATAGAAACAGTAATATTTATCATAGCATCTGAAATTGGAGGTGTATGATGGAGCATTTCTGAGCTGCTTTCCAGCCGGCGGT

Protein: RF -2: -7087 -> -5870 (405AA), RF -3: -5943 -> -1618 (1441AA)

Comparison with *Tribolium* Dicer-1 (1835AA)

Range 1: E=2e-123; bits=434

Query 7087 MACYINENVYTHTFTPREYQVELLDSAKKRNTIVCSSTSSSKAFIVVKLLQELSWQMRRN 6908

MACY+NENVYTHTFTPREYQVELLDSAKKRNTIVCSS SS+KAFI +KLLQE S +MR

Sbjct 1 MACYLNENVYTHTFTPREYQVELLDSAKKRNTIVCSSASSAKAFITIKLLQEFSHKMRVP 60

Query 6907 LKKKALLILDPQNVPVMASHMKFLTDLTIVSIDTETSGDINKHFCDNQVIITTAEICI-- 6734

K+AL +LD NVP+M SH+K LTDLT+ SID E + K + VI+TTAE+C+

Sbjct 61 HGKQALFVLDGPNVPIMTSHVKLLTDLTVTSIDKEENPPSLKA---SNVIVTTAEVCVLL 117

Query 6733 NSNTLLDIKSYNLLVIDDCLYGKRQYMIKQIMSKYQDLTPEEQPRIlglttgllGSELQP 6554

+ + SY L+VID CLYG +Q ++++IM++YQ + +PRILGLT GLLGSE+QP

Sbjct 118 CKKNFVHLDSYALIVID-CLYGGQQSLVREIMARYQAIQ-APRPRILGLTAGLLGSEMQP 175

Query 6553 ERLEAELQRLEKLLNATVDTSSEIVTLIRLSCHPRESIIQCSRLNSFTLQEELIDIVQNC 6374

+RLEAELQRLEKLL+++VDTSSEI+TLIRLSC PRE I++C + LQ+++ + +C

Sbjct 176 DRLEAELQRLEKLLSSSVDTSSEILTLIRLSCRPRERIVECFKPIPSPLQDKIKATITSC 235

Query 6373 IEFIKDHRYDPSEIYEDEFLEEYKDIPDPKITPLELLNDYLSILEDLGPWGADRAAMNLL 6194

+F+KDHRYDPSEIY+D+ LEE+K +PDPK PL +D+L IL+DLGPW ADRAA +L

Sbjct 236 QDFLKDHRYDPSEIYDDDLLEEFKQVPDPKEQPLSFFDDFLEILDDLGPWSADRAAYGML 295

Query 6193 SKIEKLKVKTPYERHYLLLCTVSTTLVRIRAVCDYEFEKLNNEVERIKQYSTSKVLKLIN 6014

KIEKLKVK PYERHYLLLC S+ LV IRA+C+ EF+ ++ E++ ++ST KVL+ +

Sbjct 296 IKIEKLKVKVPYERHYLLLCVASSVLVSIRALCELEFQDYTDK-EKVFRFSTPKVLRFLQ 354

Query 6013 VLRQFKPAGEKPK 5975

VL+QFKP G+KP+

Sbjct 355 VLKQFKPTGDKPE 367

Range 2: E=0.0; bits=1971

Query 5901 KKRFQQRPQFEDMLCALIFVENRYKAKALFGLLCTLSQCDDDFWWISALFSVEKVADIIK 5722

++ + R Q ++MLCAL+FV+NRYKA+ALF LLC +S+ D+++WW+S FSV K+AD ++

Sbjct 390 RRPYISRAQSDEMLCALVFVKNRYKAEALFALLCVMSKSDEEYWWVSVSFSVNKIADPVR 449

Query 5721 EPREAEHEHKIQEEVLRKFRCHECNILISTSVLEQGCDLPKCNLVIRFDLPKTFHSYIQC 5542

EPREAE EHK QEEVLRK+R HECNI+I+TS LEQGCDLPKCNLVIRFDLP++FHSYI

Sbjct 450 EPREAESEHKRQEEVLRKYRSHECNIMIATSALEQGCDLPKCNLVIRFDLPQSFHSYIHS 509

Query 5541 KARARASEAHYILFANDDEIDSFVTSLAEYNEVENTLLRRCSSLEPDKTEEQLADSFSKC 5362

KARARA+EAH++L AN++E+ FV +LAEYNEVENTLL+RC SLEPDK EE +AD+ S

Sbjct 510 KARARANEAHFLLLANENEVSDFVENLAEYNEVENTLLKRCYSLEPDKNEELVADASSLQ 569

Query 5361 CKPYQPIKEEGSPSVTLFNSIALINKYCAKLPSDTFTRLTPIWHEEKVGDSYVCHIRLPI 5182

C+PYQP E G+ SV+L N+IAL+N+YCAKLPSDTFTRLTPIWHEEKV + Y+C IRLPI

Sbjct 570 CRPYQPSAEPGANSVSLSNAIALVNRYCAKLPSDTFTRLTPIWHEEKVENGYICSIRLPI 629

Query 5181 NSPVKQVVTSPLMPNSLLSRRAAAFMLCQYLHKVGELDDQLQPINKENFVPAGEDWNNIT 5002

NSPVK+ VTSP M N+LL+RRAAAFM+CQ LHK GELDD LQPI KENF EDWN+

Sbjct 630 NSPVKKTVTSPPMINTLLARRAAAFMICQLLHKAGELDDNLQPIGKENFKVNEEDWNSSA 689

Query 5001 LDEQYDENSEVRPGTTKRRQYYYKKVADALLHCHPVIGQPTYFYQIVMTLTCPLPEEQNT 4822

L+E +EN + RPGTTKRRQYYYKKVADALL CHP+IGQPTYFY+IVM LTCPLPEEQNT

Sbjct 690 LEESDEENLDPRPGTTKRRQYYYKKVADALLDCHPIIGQPTYFYKIVMKLTCPLPEEQNT 749

Query 4821 RGRKIYPPEESPQGFGILTNREIPKISAFPIFTRSGEVEVELRLCAKDVIVNEEQTHKIQ 4642

RGRKIYPPE+SPQGFGILT++EIPKISAFPIFTRSGEV V+L+LC++ +IV E Q KI+

Sbjct 750 RGRKIYPPEDSPQGFGILTSKEIPKISAFPIFTRSGEVSVDLQLCSQ-LIVTENQICKIR 808

Query 4641 EFVNYTFTTVLRLQKYLMLFKPELSDKNYLIVPTIQSEHGIDVDWEFIDIIYKNLHFVPR 4462

EF+NYTFT+VLRLQKYL LF P+ S +YLIVPTI VDW+FID+IY NL +P

Sbjct 809 EFLNYTFTSVLRLQKYLTLFNPDASANSYLIVPTIDGA-TTTVDWDFIDLIYANLTVLPE 867

Query 4461 VIPDEDRKDYVFNTDDYKDAVVMPWYRNQDQPQYFYVAEICSFLNPTSAFPGSEYKSFEQ 4282

+IP+E RK Y F+ + Y+DAVVMPWYRNQDQPQYFYVAEICS LNP S FPGS+Y +FE+

Sbjct 868 IIPEEVRKSYEFDPEKYRDAVVMPWYRNQDQPQYFYVAEICSNLNPASDFPGSDYATFEE 927

Query 4281 YYLIKYAIQIQNLEQYLLDVDHTSARLNFLTPRYVNRKGVALPTSSEETKRAKRENLEQK 4102

YYL KY+IQIQN Q+LLDVDHTSARLNFLTPRYVNRKGVALPTSSE TKRAKRE LEQK

Sbjct 928 YYLRKYSIQIQNKSQHLLDVDHTSARLNFLTPRYVNRKGVALPTSSEATKRAKREKLEQK 987

Query 4101 QILVPELCAIHPFSASLWRKAVALPCVLYRINALLLADQIRTTVASDLNLGKTKLDDDFK 3922

QILVPELCAIHPFSASLWRKAV LPC+LYRINALLLADQIR TVA +LNLGK +LD +FK

Sbjct 988 QILVPELCAIHPFSASLWRKAVCLPCILYRINALLLADQIRRTVALELNLGKIELDSEFK 1047

Query 3921 WTPLNFGWSLSDVLKKSRDEEMKKQEEKLKSLREKTTELKITNLVSDASEEDPVnsdadi 3742

W PLNFGWSL+DVLKKS+DEE KKQE+ + E+ T + +

Sbjct 1048 WPPLNFGWSLADVLKKSKDEEKKKQEKIEPVIE----EIPCTEIAKIEDFD--------- 1094

Query 3741 seaeeeegndegkggDNWVVGTWSNEMAQSSQGDSTALVRYASPTSWLHGNsydedfsdd 3562

+ + +GTWSN+MAQ + +VRYASPTSW+ + D

Sbjct 1095 ---------QDDDEEEMIEIGTWSNDMAQLNSDQEFPVVRYASPTSWMD---LQNTYDDS 1142

Query 3561 msdnsdfdseeSTHEWGRLRIEFTGDHQAEALDDDDSKEDELYFVEDVNAWTVDDNSQET 3382

+SD+ +ES EWG LRIEFTGD+ AEA+DD++ K+D+ V+ N W V+D S+ T

Sbjct 1143 SFSDSDYSGDESESEWGGLRIEFTGDNVAEAVDDENKKDDDFELVDYSNVWKVEDESEIT 1202

Query 3381 ERLRHEFNNACLRNKEHILSSGILIKENEEFEKKS--QNTPADDIPPVCPDNFDFDSLMI 3208

+ LR +F++AC RNK+HILSSGIL+ ++E+F+K S NT D +FDF L I

Sbjct 1203 QTLRKQFHDACARNKDHILSSGILVSKSEQFQKCSDCDNTTTKDSQVANSYDFDFGKLFI 1262

Query 3207 G--------------------DI----------------------NIEQSSS-----APQ 3169

DI N+ Q + APQ

Sbjct 1263 ELDQHKALQIDLAPQDARNEYDISETMTFKFDEQPNLVEHPGPSPNLHQHKALQIDLAPQ 1322

Query 3168 EPFVNVYDISEHIQNFSFDEQPNLDTHPGPSPNVLLQALTMSNANDGINLERQETVGDSF 2989

+ N YDISE + F FDEQPNL HPGPSPNVLLQALTMSNANDGINLER ET+GDSF

Sbjct 1323 DA-RNEYDISETM-TFKFDEQPNLVEHPGPSPNVLLQALTMSNANDGINLERLETIGDSF 1380

Query 2988 LKYAITTYLYKTYENIHEGKLSHLRSKHVSnlnlyklgklknLAEYMVATKFDPHDNWLP 2809

LKYAIT YLY YEN+HEGKLSHLRSK VSNLNLY+LG+ K L EYM+ATKFDPHDNWLP

Sbjct 1381 LKYAITNYLYSKYENVHEGKLSHLRSKQVSNLNLYRLGRRKGLGEYMIATKFDPHDNWLP 1440

Query 2808 PCFYVPKQLEDALIDAQFPANCWTVADMAATRNMTIDEVCTLVRERG-IYSLPNIIPYNL 2632

PCFYVPK+LE+ALIDAQFPANCWTVADMAATR+MT+D++C++VR+RG SL NIIPYNL

Sbjct 1441 PCFYVPKELEEALIDAQFPANCWTVADMAATRDMTLDDICSMVRQRGESLSLSNIIPYNL 1500

Query 2631 VTQHSIPDKSIADCVEALIGAYLIECGPRGALLFMAWLGIKVLPKNEDGSYGELDLPKSP 2452

VTQHSIPDKSIADCVEALIGAYLIECGPRGALLFMAWLGI+VLP+ EDG+YGE++LPKSP

Sbjct 1501 VTQHSIPDKSIADCVEALIGAYLIECGPRGALLFMAWLGIRVLPQLEDGTYGEIELPKSP 1560

Query 2451 LLRNIPNPEEELEKLLDGYDTFEKHIGYKFRDRSYLLQAFTHASYSPNRLTDCYQRLEFL 2272

L ++ P EEL+ LLDGYD FE+HIGYKFRDRSYLLQA THAS+SPN LTDCYQRLEFL

Sbjct 1561 LSNHLTYPREELDMLLDGYDQFERHIGYKFRDRSYLLQALTHASFSPNTLTDCYQRLEFL 1620

Query 2271 GDALLDFIITKALFEDSRMHSPGALTDLRSALVNNTIFASLAVKHGFHKYFKHLSPGLNE 2092

GDA+LD++IT+ L+ED+RMHSPGALTDLRSALVNNTIFASLAV++GFH+YF++LSP LNE

Sbjct 1621 GDAVLDYLITRHLYEDTRMHSPGALTDLRSALVNNTIFASLAVRNGFHRYFRNLSPSLNE 1680

Query 2091 VIERFVRLQEESGHTIVDEHYLvvdteceevedveVPKALGDVFESVAGAIYLDSGMSLD 1912

V+E+FVRLQE+SGHT+VDE YL E EEVEDVEVPKALGDVFESVAGAI+LDSGMSLD

Sbjct 1681 VVEKFVRLQEDSGHTLVDELYL--VVETEEVEDVEVPKALGDVFESVAGAIFLDSGMSLD 1738

Query 1911 AVWKVYHRMMKTEIEQFSNKVPKSPIRELLELEPETAKFGRPEKLADGRRVRVTVQVFGK 1732

AVWKVY+ MMK+EIEQFSNKVPKSPIRELLELEPETAKFG+PEKLADGRRVRVTV+VFGK

Sbjct 1739 AVWKVYYNMMKSEIEQFSNKVPKSPIRELLELEPETAKFGKPEKLADGRRVRVTVEVFGK 1798

Query 1731 GTFKGIGRNYRIAKCTAAKCALKHLKRRGLLR 1636

G FKGIGRNYRIAKCTAAKCALK+LK+RGL++

Sbjct 1799 GVFKGIGRNYRIAKCTAAKCALKNLKKRGLIK 1830

Graphical representation

Cp.comp36004_c0_seq2: -5943 -> -1618 (length 4326)


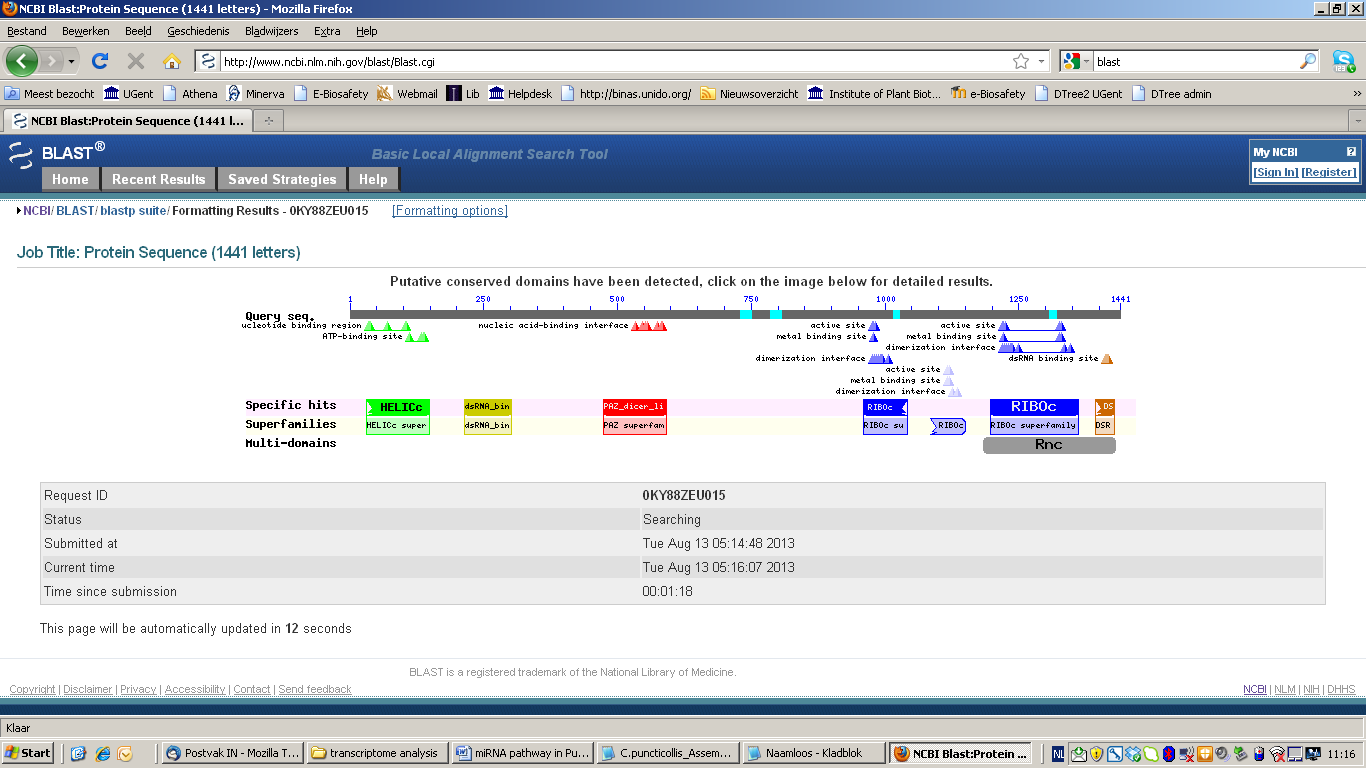


**Dicer-2**

>Cp.comp37119_c0_seq1 len=5891

TTATATAGTTCGCTAGGTTGGAAATAATTTTGCACTAATAGTGTATGGAAGGGGTGTAAAATAAAAGACAACAGTCGCCCTGATTAAAATTACTTCAATGTTGCCAATATTCTACGCAAAATGTTCAATAAATTGACATTTGCGACATATCTTACAGTGCGCCGTCCCGTCGTCAGATGTTCGCGTCCGGTTAAGGGAGGTTCAAGATTTGTTTTAACCAAAAAAAGATGTCCGCGTCTGGTCATTAGAGTGTCCGGTTATAGGAGTCATTTTTACATGTAAATTAATAGGCAAAAGTTTGGTTCCAGAAAAATTGTCCGCTTAATAGAGGTGTCCGCAAGGAGAGGTTTCACTATACTAAAAATATGTTTTGGTTTTTTTTTAGTGAACAATATATAAATAGCTTTATATAAATCAATAATGTAACATTTAACCAATTAAATCAATTTAAATGAAAATAATAAAATCATCTAACCTAATAAACGTACGGCTAACTTGGCTGCAGCTTTTTTGGCTAAAGATTTGTTCGTTCCGAATCCAAAAACTCTCTGGAAGTGCCCATTGAGCATAAATTCCAAGGCAACCATCACTTTATCTTTTCCGACCTCAACGGAATTACCGAATTTAGGGTGAGGATTGGGGTTCCACTCATACAACCTTCTGATCAAATTTTTAGGAACGTTCGCGCTAAAAGTTTCTAGCTCTTTCCACATCAGACGATGAAATATTGACCATACAGTATGTAAATTCTTTCCGCTATCTAAATATATCGCACCAGCAATGGATTCAAACAAATCTCCTAAAACCTTGGGAACGTCAATGTATTCAGCAAGATTTAAACAAGCATCATCATCTACTCCTTCGGTGAGCAAAGTCAACACTTCATCGTCAATTTCGTAATTTTTCGATTCGATGTACTCGGAAAATTTGTCTATTAAGCCTTGCAACTTACTGTTTATCATCAGAAAACACTTGTGGAAATTATATTTGACTGCCAGGCTCGCGAACGTGTTATTATTGACTAGCGCTGATCGCAAATCTGTCAAGTCGCCCGGAGACAGATTGCCGCACGATTCGTATATATGACATGTGATCAGGAAATCCAGGACGGCATCCCCGATAAACTCTAGTTTCTCATACGACTGGGTAATACGATTGGGGGAGTACGACGCATGGGTAAGTGCTTGTAGCAGATATCCACGATTTTTAAAGCTATAACCCAAGATAGCTTCAATTTCTGCCCGCTGAGGGATATGGAAATCAATATCCGCTAGGGATGATTTCTCCTCATTTAGGATCGGATTTTTTGGGTCTTGGGCGAGCAGCTCCATAAAGTTTTCCGATCGAGGAATTATTCCCATCCATTCCATAAATTTAATGCCTCCGCTGATTCCACAGCATTGGAAATAAGCGCCCAGTAAAGCTTCGACACAATCAGCCACCTGTTTATCACCGACAAAATTGCATTTGAAAAGAGAAGCGATTTCGTTAATCAACCCCTCCTCGCTGGGATCTTGTACCGAGCTCTCGTTCATTATTTCATCAATCGTCGCTTGAGACAATTGCCCAGAGGTCTGCTCTTCAATAGGAATATAAACATGGTACAAAGATGACAGGGCAGTCTCGTTGTTTTCGATCCGCTTGGTCACCAAATCGGGGATCTTAAAACCTGGGGGTAACCAATCGGAGATCTGCAAATCGGTGTTTTTCAACAGGCCTCCGATTTGCTTCCGTTCACCAAAGTAGTACAAATTCTTGTTGCTGACCAATCTTCCTTTGAGAGTTGTTGATTGGCCCTCATTGTACTTTGGAAATTTCAAGTAGATGTATATCGACGAATACAATTTCAAAAAAGAATCGCCAAGTGTTTCGAGCCGTTCCATGTTAACGATATCATTTGCTTTGGCCGTGGTCATTGCTTTGTAGATTTCGGACAACTGAGGACCTTCCTGACTTTTTTCTTCCAGAAGCCGTATTGGTTTCAATACATAATCTTTGTGATAAGTTAGAGCTAACTGATTCTTCTTAACACCATTTTCATTTTTTAAAAAGACGTCGGACTCTTTCAAAGGAGTCGATATAAATTTTTCATAATATTCTATGTCCATTACAGTGACGTCGTGTATTTCCCTCTCCAAATCCTTGGGTTCTTCGATGTCTTTCCAAGGGTAATCCCTCTCAAGTGTTTTGATGGCATAATCTCTGTTTAAGTTCATAGCAGGCATTGTGGGAAGCCTATAATCTTCCTTCGGAATGGGCATTAATACGTTTATTTCCGTTTTTTTTCTGCAAATGAGGTACATAATTCAATAAATGCAAGTCCAATTCCAAAGAGGCCTTATTGGGTACGTCTTTCTGTCCCAAACCAGCTTCTGTAGCGATCTGAACTCTCAGGTATTCCAGTCGGAACAAAAATGACAGCCTCGACAATATAGTGGGTAGCAAGCTTGCCTGAATCCATAAAGACGCGGGAAATTCTTGCTTGATGACCAGTTCGGGTATCAAATGAATGTCCAACTCCTCGAACATTTCATCTCGTTTTCTTCTGGAAGCCGCTCCCTTCGGTTTAAAACAATTTAACTTTTTTGACAAACTCTTGACGAACAAGAGAGGCTGGTTCGGATTTACGAGTTGTAGGTTATGTTTGTCGTTGAAGTATTGCTCATAAGTGCCAAACGATTCGTTCGGAAATACGCTTCGGGCTGTTTTTGTAAAGGTCACTTCAGTTACAATGTAAAACCCTGTATTTCTGTACCAAGGCGCCACGATCTTGTTCAAATAAGTGTCCAAATTAACTTCCACATTTAATTTTTCTTCCTGACTGGGCTCCATTACGCTTTTTACAGTCTTGTGCTCCTTTAAGATGGAAAAATCAATGCACCCATGTTCTTTATCGACTGGTACTATCAATATCGACTCAGCGTCCTTTGAATTATCGAAGATTAAAAATTCCCTCAAACAAGTCAATATATCGGAATATACCAACACGTTAAATGCTTTAATATCATCGATATTTTCGGCGGTGAGATTTACCGTAGAGTAATTCTCTTTAAGGGAAATATTTAAAGTACCCAAAGATACGAATACCGGAAAATCGCATATCTCGGGGACACGTTCAGGGGTTATTATCCCATAACACAGGCTCGAGTTGTACATATCATACATGGTAGCCCTGTTTATATTTTCTTCCCTTTTAAATGCTGGTGTTACCTCTATTATGTGCAGGTAAACGGGTTTTGTAGACGTTATCGGTCCTCTAATATATTGTGGAATCACTTTCTTGTGTAGCCTTTTCCGTTTGTTGGTACCTGCGTCAGGTTCCTTCTCCGCCGGGTAATGTTTGAACAAAAAACTGACATCCTCCTCGGATATCCCTGCCTTTTTAGGTAACATATTATCGTCCAATTCTCCGCACTGGTGGAGCAATTTGCATGCCTGTAGCGCTGCAGCTCGCTTGGCAAGCTTAATGCTGGGCATTGGGATGCCCTCAACAATGTCAATTGGGCAAATCGTAGGCAATCTGATGACCACTTTAATAAGTTCCAGACCAGAGGAATCCAAAAATGATTTATAAAAAACTTCCGGAGTGTAAACTGTGTATTTGTCAGAGGGAAGACTAAGGCAATATGAACATAGCAGAGATATTGCAGATGTTGACGTTACTCGCGCCGAATTCGGACCATTGACATAATAAGGTGCGAGACAGTCTTCGTTGTACATTTCCTCTATGTCTCTTTGAGATGGCTCGGAACGTTCTAAATTTTTGCCAATAAGGAACTCATTTAATGTAGATTCGACTTCTTGATACTCCTGGTACCTGGTTTTAAATGTATCCTTGTCTTTATCATCCACGAACATCATGTATAAACTGGATTTATTCCGAGCCCTGCCTTTTGACTGTATGTAAGAACGATAATCTTCAGGATGTTCGAACTTAACGACTAAAGAACACATAGTTATGTCCACACCCTCTTCCAGCACATTGGACGCGACCAATACGTTGATCTCCTTGTTTCCAAAGCTATTCAGGATTTTTCTTGTTTTCTTTCTTATGTATGAACACTCCCTGGTGTCATTGTAAGGGTTGCTGTTATTGCCGACTACAAAATCGGTCTTAATGTGTTCAAATTTCGGAGATGATTTGCTTAAACTATCCAATATGTAATAGACAACCTTTGCTGTAAATCTTCTTTTTGTGAAGATAATAGCGCACAATTCTTCCTTGGACATTGTAGGGTAGTTCTCCAACAGTGTTATCAATTTTTTGACCTTTTCAGAAGAGAATAAAAGAATCTTGTTAAATTCGTCATAGTCTTTCATAGCTTGAGAGAATAATCCCCTGACGTACATGAAAGTCACCCCAACGTAATTCAGTACTTTGTATAAAGTGACATCGTCACAATGCTTCCTTAACCGTTCAATTTGTATAAGATGAGCCAAAAGAGACTTATCCCCTCCGTAGCTCCCTAACGATTCTATTTGGAAAATAATGTCTCTAATTAAGTTTTGCAGTTGTTTGAGTCCCTTGTTTTTGTTTACTGGTTTCAAATCAATCTGAGGTGGGTCGTTGTAAGAAGAAACTATTTTGACACACCCAATTGTGACTATTAATTCGTTCAAAACGCCAATTACTTTATTTTCAATAGGCAACAATGTATGTGTTTTAAATTCGACAATGTTTTCCCATGGATTTGTAGAATAACCAATGACTTGATCTAAGCCATTCACGGTAGCCACTTTGCCATGATAAGTAATTTCCAAGTCCTTCACCATATCCATAACCCTACCTGGTTTACAATTACCGTTCAATAATGTTGCCGTCAACCCAATAATTCTTGGTGGTTCGTTGACGTTTTCCAGTGATTTACAAAGCTGACGCATTGAATGATCGTTTACACCCCTATGGCACTCATCAAAAATCAATAAATTCACTTTGTTCAGGTCTATAAATCCTTGATTAACCAAATTGGCAAGTATCTGGACTGTCATAACAATTACCTGGTACTTATCAAATTCGTTCAACCATTTCTCTTTACTCCATCCGTCCAAATTCATATCTCCACTATAGGAGCCCACTTTAAAAGAAGTTCTTTGTTCGATACACTTTGTATGCTGATCGACGAGTGCTACTGTATTCACCAGGATAAATGACAGTTTGCCATCTTCGCTGTATGGCTTGGATAAGCTCTGACTCAATCTCTTTAGCACCAACACCGCGATGAAGGTCTTTCCGGAACCAGTGGGCAAATAGATTATAGTATTTTTCTCTAAAGCAATTTCCATAAGAGCAACCTGATAATTTCTCGGAGTCATTTCATCCTTTGTCTCCATTGTTTTGCTATTAATTGCACTTGCTTCTTCACTATCTCGTGACATATGGACAGGTCACGTCAACTCTGAAGTTTTGCTATTTCTGTAAATACCCTGTCAAGTCAGGAACAGCCCATACCAACAAAAAACTTGTCCTTTAATATTGATGGACCGCTTTACCAAAGCAGTTATAATCATTTAGGCGATGCACATACCTTTTTTGTAATTAATATAAATAAGTATTATTTTTTTCTACACTTTTATTGAACTAGTCAATTCAAAAAAGTTCCAAAAGTACGATATAAAAATCTGTATTCCGGTTTTAACATAGCCTGCAAGGCGTCACAGACTATTATTCCCTTTATCAATAAGGCAAACTTTGTTTAAACCATGTCAGGTAGAAAGCTCGGCATGGTCCGTTACATACACAGCACCACCAAGTCCGTTATAATCACCATAATAATCATAGACATACTAAAGATTAGTTATAATTGATGTTAAATAAACAAACGTGGACCCCACGTGGACCAATCCTAGGGCCCATAGACAATACCACCAACTAGGGCCTACTTAAATGCTGG

Protein: RF -3: -5376-> -2236 (1046AA); RF -1: -2303 -> -471 (610AA)

Comparison with *Tribolium* Dicer-2 (1623AA)

Range 1: E= 0.0; bits=1012

Query 5331 METKDEMTPRNYQVALMEIALEKNTIIYLPTGSGKTFIAVLVLKRLSQSLSKPYSEDGKL 5152

M+ +DE+ PRNYQV LMEIA+ +NTIIYLPTGSGKTFIA++VLK+L + +PYS+ GK+

Sbjct 1 MDEEDELKPRNYQVNLMEIAIRENTIIYLPTGSGKTFIAIMVLKQLCAPILRPYSDGGKI 60

Query 5151 SFILVNTVALVDQHTKCIEQRTSFKVGSYSGDMNLDGWSKEKWLNEFDKYQVIVMTVQIL 4972

S ILVN+VALVDQH K + +F VG+Y+G+MN+D WS+ +W +F+KYQV++MT QI+

Sbjct 61 SVILVNSVALVDQHGKYVRDHATFSVGTYTGEMNVDFWSEAEWEQQFNKYQVVIMTSQIM 120

Query 4971 ANLVNQGFIDLNKVNLLIFDECHRGVNDHSMRQLCKSLENVNEPPRIIGLTATLLNGNCK 4792

NL+N FIDL KVNL+IFDECH GV D MRQ+ K + + PR++GLTATLLNGNCK

Sbjct 121 VNLINNRFIDLGKVNLMIFDECHHGVEDQPMRQIMKHFHSCTDKPRVLGLTATLLNGNCK 180

Query 4791 PGRVMDMVKDLEITYHGKVATVNGLDQVIGYSTNPWENIVEFKTHTLLPIENKVIGVLNE 4612

+VMD ++ LE+T+H KVATV GLD V+GYSTNP E + L +V+ L +

Sbjct 181 LSKVMDEIRSLEVTFHSKVATVEGLDVVVGYSTNPQELFKVCQPGALSLDAKQVLNNLRQ 240

Query 4611 LIVTIGCVKIVSSYNDPPQID---LKPVNKNKGLKQLQNLIRDIIFQIESLGSYGGDKSL 4441

LI + + I N + LKP+ + LK L+NLI D++ IE LG++GG +

Sbjct 241 LINDLEHINIKDEQNSVNLLQSETLKPLEPSDVLKSLRNLISDLMIHIEMLGAFGGHIAC 300

Query 4440 LAHLIQIERLRKHCDDVTLYKVLNYVGVTFMYVRGLFSQAMKDYDEFNKILLFSSEKVKK 4261

+AH+IQIER++KHC + L+ VLNYV + L + M Y+ KI FSS+KV K

Sbjct 301 VAHMIQIERIKKHCQNHQLFIVLNYVMTIMGTTKLLLEETMAGYEPLEKIRKFSSDKVLK 360

Query 4260 LITLLENYPTMSKEELCAIIFTKRRFTAKVVYYILDSLSKSSPKFEHIKTDFVVGNNSNP 4081

+ +L+ Y T S EELC ++FTKRRFTAKV+++I+D S+ PKF HIK++FVVGN +NP

Sbjct 361 VFEILDEYKTKSDEELCCLVFTKRRFTAKVLHHIIDKASQVDPKFYHIKSNFVVGNKNNP 420

Query 4080 YNDTRECSYIRKKTRKILNSFGNKEINVLVASNVLEEGVDITMCSLVVKFEHPEDYRSYI 3901

YNDTRE YI KK R++LNSF +KEINVLV+SNVLEEGVDI C+LV+KF+ EDYRSYI

Sbjct 421 YNDTRENLYITKKNREVLNSFVSKEINVLVSSNVLEEGVDIPKCTLVIKFDKSEDYRSYI 480

Query 3900 QSKGRARNKSSLYMMFVddkdkDTFKTRYQEYQEVESTLNEFLIGKNLERSEPSQRDIEE 3721

QSKGRAR+ SLY V+ D + +Y ++E+E+ +N+ LIGKN ER P+ +I

Sbjct 481 QSKGRARHIKSLYYTIVETTDVAKYDKKYSAFKEIENLVNDLLIGKNSERDHPNLSEIRN 540

Query 3720 MYNEDCLAPYYVNGPNSARVTSTSAIsllcsyclslpSDKYTVYTPEVFYKSFLDSSGLE 3541

MYNED L PYYVNGPNSA+V TSA++LLC YC +L SDKYT Y PE +Y+ DSS +

Sbjct 541 MYNEDKLEPYYVNGPNSAQVNMTSAVALLCRYCSNLASDKYTTYAPEWYYEE--DSSSAK 598

Query 3540 LIKVVIRLPTICP-IDIVEGIPMPSIKLAKRAAALQACKLLHQCGELDDNMLPKKAGISE 3364

L +VVI LP +CP ID + G M + K AKRAAAL AC LHQCGELD+N+LP K + E

Sbjct 599 L-RVVIFLPVVCPLIDPIVGPYMHNKKDAKRAAALVACIKLHQCGELDNNLLPWKKQLDE 657

Query 3363 EDVSFLFKHYPAEKEPDAGTNKRKRLHKKVIPQYIRGPITSTKPVYLHIIEVTPAFKREE 3184

DVS+LF H+P EKE DAG K+KRLH K I ++ I + +YLH I + P +KR +

Sbjct 658 ADVSYLFTHWPQEKESDAGNKKKKRLHDKEIAPSVKSAIQPDRVLYLHTININPQYKRSD 717

Query 3183 NI-NRATMYDMYNSSLCYGIITPERVPEICDFPVFVSLGTLNISLKENYSTVNLTAENID 3007

++ N T+YD+Y + L +G+++P+ +P++C FP+F S GTL I ++ N V A +

Sbjct 718 DLKNAVTIYDLYKTPLKFGLLSPKPLPDLCKFPLFDSNGTLEIEIRNNVREVEFAANEMK 777

Query 3006 DIKAFNVLVYSDILTCLREFLIFDNSK-DAESILIVPVDKEHG--CIDFSILKEHKTVKS 2836

+++ F+ LV++D+L L+EFLIFDN+ ++E +L+VPV G C+DF +++++K +K+

Sbjct 778 EMREFHFLVFNDLLEILKEFLIFDNTGMNSEMLLVVPVQDRCGDVCVDFRVIRDNKNLKN 837

Query 2835 VMEPSQEEKLNVEVNLDTYLNKIVAPWYRN-TGFYIVTEVTFTKTARSVFPNESFGTYEQ 2659

+EP+ E++N+ V +TYL+KIV+PWYR+ Y+VT+V K+A S FPN + +

Sbjct 838 KLEPAATERINLNVTEETYLHKIVSPWYRSPPKMYVVTKVCPDKSALSRFPNHEYPNFVS 897

Query 2658 YFNDKHnlqlvnpnqplLFVKSLSKKLNCFKPKGAASRRKRDEMFEELDIHLIPELVIKQ 2479

Y+++KH+L +++P+QPLL VK LS++LN FKP+GA +RK+++M+EEL+ +LIPELVIKQ

Sbjct 898 YYSEKHSLSILDPSQPLLLVKGLSERLNAFKPRGAGGKRKKEKMYEELEEYLIPELVIKQ 957

Query 2478 EFPASLWIQASLLPTILSRLSFLFRLEYLRVQIATEAGLGQKDVPNKASLELDLHLLNYV 2299

EFP+ LWIQA LP+ILSRL++L +L+ L+V IA G + + + LEL+LHLL+Y

Sbjct 958 EFPSCLWIQARFLPSILSRLAYLLKLQQLQVDIARGIGAKAEYLKDCPPLELNLHLLHYE 1017

Query 2298 PH 2293

P+

Sbjct 1018 PN 1019

Range 2: E= 0.0; bits=647

Query 2207 NRDYAIKTLERDYPWKDIEEPKDLEREIHDVTVMDIEYYEKFISTPLKESDVFLKNENGV 2028

N+D+A K LE +Y WK IEEPKD+ER I +VTVMDIEYYE FIS ++ LKN++ V

Sbjct 1052 NKDFAAKMLEAEYYWKTIEEPKDIERNI-NVTVMDIEYYETFISHQPSKTGRLLKNDSPV 1110

Query 2027 KKNQL-ALTYHKDYVLKPIRLLEEK-SQEGPQLSEIYKAMTTAKANDIVNMERLETLGDS 1854

K+ + A+TY + K +++L+ + + P L +IY+A+T A+ANDIVN+ERLETLGDS

Sbjct 1111 KQQNVPAITYDCQFEAKQLQILDVQFDNQSPNLCQIYQALTAAEANDIVNLERLETLGDS 1170

Query 1853 FLKLYSSIYIYLKFPKYNEGQSTTLKGRLVSNKNLYYFGERKQIGGLLKNTDLQISDWLP 1674

FLK +S+YI KFP YNEG+STTLKG+LVSNKNLYY G RK +GG+LKN+DL SDW+P

Sbjct 1171 FLKFVASLYIIFKFPTYNEGKSTTLKGKLVSNKNLYYLGVRKNLGGILKNSDLSPSDWVP 1230

Query 1673 PGFKIPDLVTKRIENNETALSSLYHVYIPIEEQTSGQLSQATIDEIMNESSVQDPSEEGL 1494

P F IP ++K I N E ++ SL++ I EEQ SG L++ T+ ++ E P EE

Sbjct 1231 PCFCIPQTISKAIGNKEYSVVSLFNCCISPEEQVSGNLNRKTLSDMTTEEIA--PDEENS 1288

Query 1493 INEIASLFKCNFVGDKQVADCVEALLGAYFQCCGISGGIKFMEWMGIIPRSENFMELLAQ 1314

+ + +VGDK +AD VEALLGAYF GI GGIKFMEW+GI+P SE L+

Sbjct 1289 YGNMCNFLNKQYVGDKSIADSVEALLGAYFLSGGIQGGIKFMEWIGILPLSEQIQRLIET 1348

Query 1313 DPKNPILNEEKSSLADIDFHIPQRAEIEAILGYSFKNRGYLLQALTHASYSPNRITQSYE 1134

+P+LN+ KS+ D+DFH+PQ EIE LGY+F NR +LLQALTH+SYSPNRIT SYE

Sbjct 1349 TQVDPVLNK-KSTKTDVDFHMPQWREIEQRLGYTFTNRAFLLQALTHSSYSPNRITLSYE 1407

Query 1133 KLEFIGDAVLDFLITCHIYESCGNLSPGDLTDLRSALVNNNTFASLAVKYNFHKCFLMIN 954

+LEF+GDAVLDFLITC+I+E CG+L PG +TDLRS+LVNNNTFASL V+ FHK LM+N

Sbjct 1408 RLEFLGDAVLDFLITCYIFEHCGHLEPGQVTDLRSSLVNNNTFASLVVRCGFHKFLLMMN 1467

Query 953 SKLQGLIDKFSEYIESKNYEIDDEVLTLLTEGVDDDACLNLAEYIDVPKVLGDLFESIAG 774

S LQG IDKF++Y+ SKNY IDDEVL LL E +N+AEY+DVPKVLGD+FE++AG

Sbjct 1468 SNLQGHIDKFADYLASKNYVIDDEVLILLEEDE-----MNIAEYVDVPKVLGDIFEALAG 1522

Query 773 AIYLDSGKNLHTVWSIFHRLMWKELETFSANVPKNLIRRLYEWNPNPHPKFGNSVEVGKD 594

AIYLDS K+L TVW +F++++W+E++ FS NVPKN+IRRLYE + P+F ++EVG

Sbjct 1523 AIYLDSNKDLKTVWRVFYKIIWREIDLFSKNVPKNVIRRLYECHTVYPPQFSKALEVGNQ 1582

Query 593 KVMVALEFMLNGHFQRVFGFGTN 525

K MV+L+FM G +RV GFGTN

Sbjct 1583 KTMVSLDFMCEGRKKRVHGFGTN 1605

Graphical representation

Cp.comp37119_c0_seq1: ORF -5376 -> -2236; -2303 -> -471


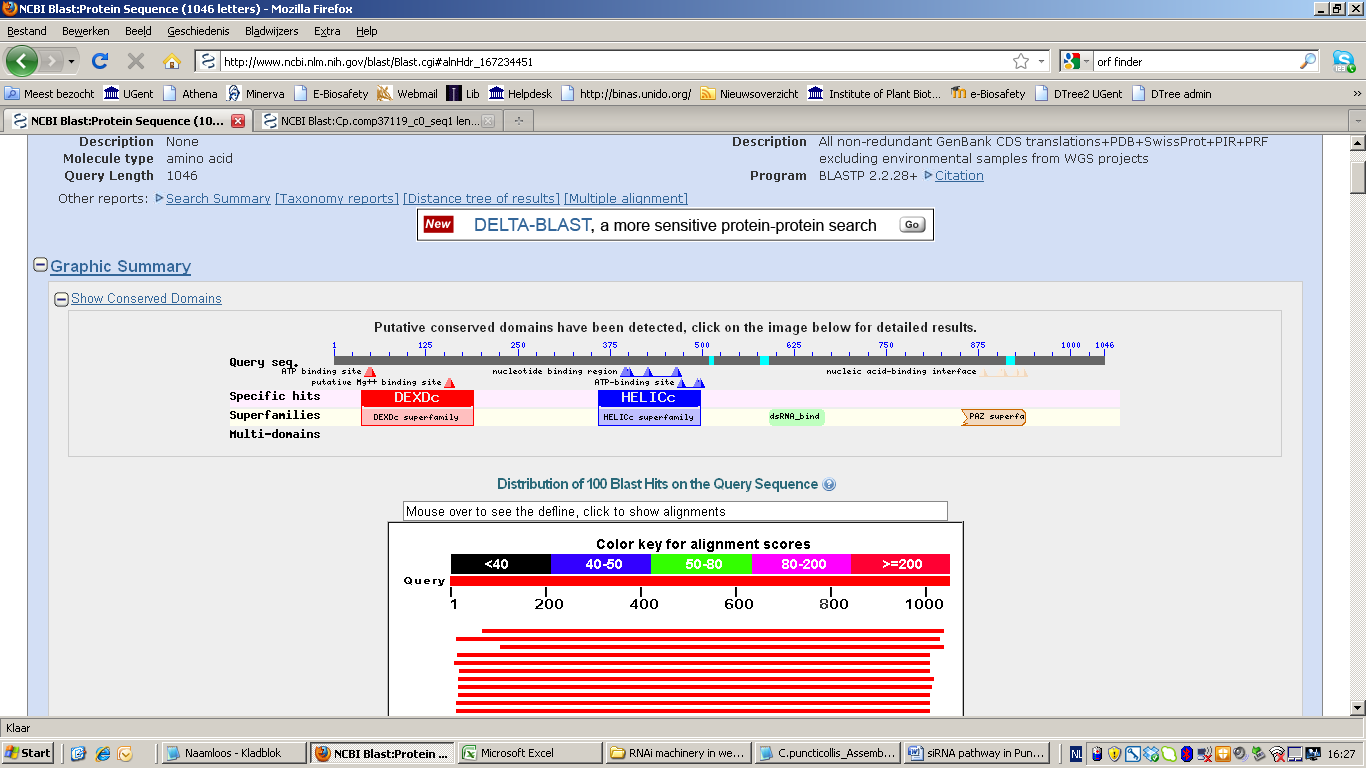


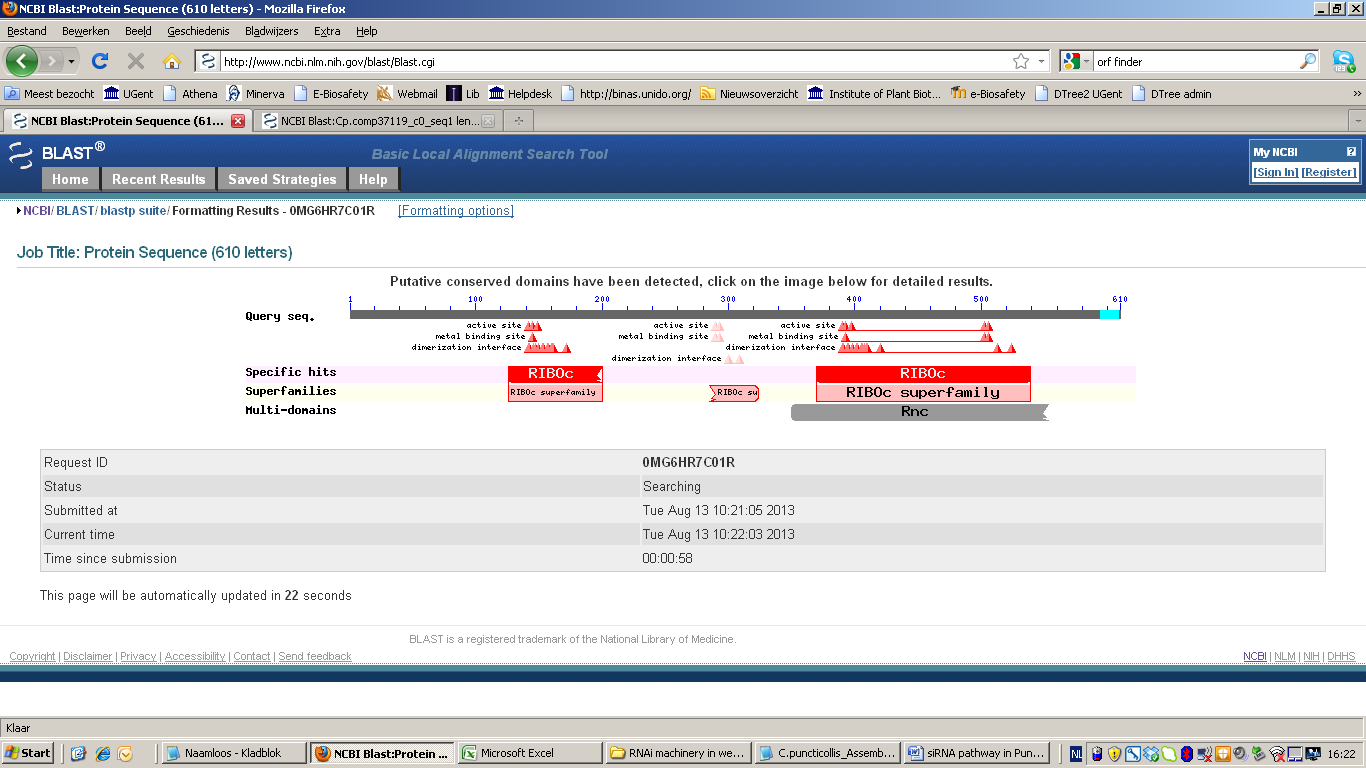


**Drosha**

>Cp.comp39990_c0_seq1 len=4060

GCGACTCGTTCGTCCGCTTTAATATCGTAATTATTTACACAATTTCAATCTTTGTACACTCTCTTCAACATAACCTACAAACAAAAGTGCCAACTTTGTTGTCAACTCGACATTCCAAAGCCTACTGCTTGTCCGCTCACTTGCCGTCGTTATCGCTGCTGCTGTTGCTACTGCTACTGCTACGGCTGCTGCTTCGTCTCGATTTGCTCGTTTTCTTTTTGGGCGTCGACGAATTGCGACTCGACAAACTCCTCGATCTCGATCGGCTCTTGAACCTGTTCCGAGAGTAACTCTCGGGCGATTTCGTCCTCAAACTATCGAAACTGGTGCGCGTTTTCGATCCGCCGTTCTTTTGCTTCTTCATACTTTTGGCGATCACTCGTTTCTGATGGTCCAGTTGCGGGAACAGATGATGCGATATTTCCAAAGCCTTCTTCGCGGCGTTCATTTCCGCTTGTTGTATACTGTGACCCATCGCGCTGGCCAATCGTCTGCCTCGAAAATAAACGGCTACGGTGTACACGCGCGTATTCGTCGGTCCCTTACATTCGATAACTTTATAAACCGGAATGTCCGGTTCGCCTCCGTCCATCGTCCGCAACGTGAGGCAGCACTGTTGCAATTTCGACTTGGGATCGTTCCAGTCTTGGTTCATGATGAAATCCTGCAGTCTCGGGAACAGGGTCACCTGACAAAACACCTCGCAATACTCCATACCTTTGTCGACGTACAGGGCGCCGATGAACGCTTCCAAAAGGTCGGCCCTGTCTTTCGTTTTCAATTCGGGTTTCGGGTTGTTGTAGATCGCGTACTGGGACATGCCCAGGTCGTCGCACACCACCGCCTGCGTCCTGTTGTTGACCAAAGAACTCCTCAACAACGACAAATGACCTTCGTGATGTTCGGGGAAGTATTTGTACAAATATTCCGACGCTATAAGTTGCAAAACGGTATCGCCCAGAAATTCCAACCTCTGATTCGAACCCATCGTGAGATTCGTGTAGCCGACGCTTCGGTCGGTAAAAGCCCTGGCCAACAAACGGATGTGATTAAACTCGACGTTGATGGCTTTCTCGAAACCGGTCAGTTTTTGAAGTATTTCGAATTTCTCGATCCAGTGACGGTCGCCGAACGGTTCCTGCTCCTGTAACGGGTGCGGCGGCAAACTTTTCCACACTTTTAACAAATCCGGTTCGTCTCTGTACAAAGTGTTCGAGAATACTTTGTCGGCTACGTCGATACCGCCGTCCAAGAACAGGGCGCCCATAAGCGCCTCGAAACAATTGGCCATGGCGTGTTTCAGTTCGAGGTCGTGGCACAAGTCCGAGCCGTGAGCGTAAAGCATGAACTGGTCGAGGTTTAGGATTTTCGCCAGCAAAGCCAAGTGCTGGTTTTGGACTATGGCCGCCCGGTAAGTGGCCAGACCGCCTTCCTCGAGGTCGGGAAACGAAAAGAACAGATGGATCGACGACAAAAATTCGACTACGGCGTCGCCTAAAAATTCCAACCGTTCGTTGTGGGTTATGTTCGATTCGGTTTCCTGTTGCCTGCCGAACCTCGACATGATGTTTATAAGCGTGTTTATGCCTCTTTTCCTCGTGTTCATGTAATGTATCCGTCTGTCGCCGTATTCCGGTTGTCTGATACCGCAATTGGTCAAACTGTTCCGGGCGTGGTCGGGATTCGTGCCGAAATTTTGCCTGTACGACGGATGCGTCAAGGCGATTTGCAACAACCCCCTGTTCTTGAACTTGTAATCGATCGACCGTTCGAGCACGTTCAGAGCGTTATGAAACCTCAAATGACAAATCAGTACCGGCATAAGCATCGCGTGTTGCACGATATCGCACATAATACCAGTACGGTAAAAACCCTCGGCCGAAACCGCTATCGTAACGTCACGTTTCATCTTGCCCTGCGTACGTATCTCTTGCAATTTGCCCTCTTTCGCTTCCAACTTGCGTTTGTCTTCGAAAGTCGGTTTGGACATGTTCGCTATAAGATGCCGATACTTGACGTACTCGCGCCACGCTTTCTGATACTCCGGATTGCCGGCGTAACTCAACTGCGGCGGTCTGATACCGAAATGAACGATTTCGGGAAATTTATAATCGCCCTCCTTTTGCAAATCGATATTCCGATCGAGTTGGTCGACTCGTACCGAACTCGGCTTTTTGCCCGGATAAGTGACGACCATACCTTTGATTTCGTCCGCGAAATGTTGCCACTCGTACTGCGACATTTTCACCATCGTTTTCAAATCGTCTTTTTGTATCAACGGTACGGCACAGTTCAACAAATAACGTAACACCACTTCCATACATAAAATTTCTTTACCGTTTTCGTTCAATTCGCGTACGAACCGAGGCATGTAGTGGAACTGGGAGCAGTGCTCGGCGTCTTTCGGCGTCGTCAAATCCAAATCGATCAGTTCGAGCACCTCCCGGAACAAGTACTCGGTGAACAAGTCCAATTCGCGTACGGTAAAATTGTCCGGCACTTTTTCCTCGAGGTACATGATCGTGTATTCGATATTGAACCGTATCACTTTGCAATGGGGCAATTGCATCAGAGGCGAATGGGAGAATATCGAAAAACCTTCGAATATGAACTCGTGCTCGTCGTGTTGGATTATCGTGGGAGCTTTTATCAGGAAATTCGTCGGCGGCGATATAATGATCCTGTAATGGTACAGCCTGTCCGCGTTGTTGCTGTACGGGTCGCATTTCGGTATACGGTTTTCTCCCGGGTATATGCCGTGCCTTATGCCCGACTTTTTCGATTTGAACGAGCAACGGCACAGCGGACCGTCGTTCATTTCGCCCGGGTCGTTGTACCACATTTCGGGATGCAATCTGTCCGGATGTTGCTGTTTCCGTTGCAATTCTTCCATGGAAAAGTTCTCCTCGTCGGTAAGGTTATCCTCGGAACATTCCGAATCGGAACTGCTCGACACTTCGGTCCGATGTTTGCAAACTTTCGCCCTGTTCTTTCTCGGCGGCGGCACGTATTTGCTCTTCGACGCGTTCACTTTCCGAGCCCTCAATACCAACCGTTCGTCGAAATCCGAACACAGCTTGACCAGTTTCTCGGTCGCTTTCGTCACTTTCATGTTGCCCTCGTCTTTCTGGTAAAACAGTTCGGAGGGCGTCGACCTGATCCAAAAACTCTTCTCTCGGTCGATCAGGTCGACCTGTTTCCTGCTGATTTCTTCCAATTTCTGCGAGATTTGCTTTTCCGTATGGCAATAGTTGCGTTTGTAACGGTTGAGGATTTCGTCTCGTTCGTTTTGCACCTGTCGGGCCGCCTCCCGACCTTTGTACCGATCCTTGGACGTCTCCCGACTGTTTTTGCTCGAGTACCTGCTTCGTTCTCTGCTTCTCGGTCTACGATTTTGTCTACTGCGACTCCTGCTCCTGCGACGTCTACTGCTGCTAATATCCCTAGACCGTCTCCTATTGTAATCGGACGGGCTGTAACTTCTCGTTGCTTTTTTCACCGCCCGGTACTCGTCCAACTCTTTTTTATAATCGTGCTCGCTTGAGAATTCCCGGTTAACCCGATACGGATACTGATAATTCGAAACCGATACCTGAACCGCGGACTGCTGATAAGAGTACTCGTAAGTGCTCGAAGAAGGCGGATAATTCGGTGGGGGAATCGTGTACGGGCTCACGACCGGCGGCGGATACGAAGGAACCGGCACTTGGGGCATCTCCTGCTGCCATGACCCGTAATGACCCGAAGGGGTCTGCGCGACGGGGTAATGCATGTAATTTACGTGAGGCGAGCCGGAATTCGGAACGGGGCACTGTTGCCCGTAATACCAGTGATCCCCCATCTTCGATAAAGACCCCGAACAACGAAGAAACTATCGATTTTATAATGATGATTGAGGTTATGTTATCTTGGGTAATTTCTTCTTTTTTTTATCCTAGTGGGCCGAACTTCTTACTCTTAATTTGTAATCGTATTCGTAATTTTAGTATACGGTTCCTTAATCCGTAAACGTATTGTTATTTCTTAAAATTCGATCTCAATTCCGTAACTCTTAACTTAC

Protein: -3841 -> -137 (1234AA)

Comparison with *Tribolium* ribonucleae III (1180AA)

Query 186 ERDEILNRYKRNYCHTEKQISQKLEEISR-KQVDLIDREKSFWIRSTPSELFYQKDEGNM 244

ERD IL+++++NYC T +++S K+ E+++ +++++EK+ W RSTPS+L+Y+KDE N

Sbjct 136 ERDLILSKWRKNYCSTREEVSNKIHELAKVDHEEVLEQEKNIWTRSTPSDLYYRKDESNA 195

Query 245 KVTKATEKLVKLCSDFDERLVLRARKVNASKSKYVPPPRKNRAKVCKHRTEVSSSSDSEC 304

+VT+AT++L +LC F++ LV+RA KVN K KY PPPRKNRA++CKH++E SSSS S

Sbjct 196 RVTRATKRLTQLCDKFNDCLVMRAAKVNKLKPKYEPPPRKNRARLCKHKSEESSSSGS-- 253

Query 305 SEDNLTDEENFSMEELQRKQQHPDRLHPEMWYNDPGEMNDGPLCRCSFKSKKSGIRHGIY 364

SE+ LTDEE+ +MEELQRKQQHPDRLHPEMWYNDPGEMNDGPLCRCS KS+KSGIRHGIY

Sbjct 254 SEEELTDEEDCTMEELQRKQQHPDRLHPEMWYNDPGEMNDGPLCRCSIKSRKSGIRHGIY 313

Query 365 PGENRIPKCDPYSNNADRLYHYRIIISPPTNFLIKAPTIIQHDEHEFIFEGFSIFSHSPL 424

PGE + KC P SNNA+RLYHYRI ISPPTNFLIK PTII +DEHEFIFEGFS+FSH PL

Sbjct 314 PGEKHLEKCVPDSNNAERLYHYRITISPPTNFLIKTPTIIHYDEHEFIFEGFSMFSHFPL 373

Query 425 MQLPHCKVIRFNIEYTIMYLEEKVPDNFTVRELDLFTEYLFREVLELIDLDLTTPKDAEH 484

+LP CKVIRFNIEYTI+Y+EEK+PDNFTVRELDLF +YLFRE+LEL+DLD D +

Sbjct 374 EKLPTCKVIRFNIEYTILYIEEKIPDNFTVRELDLFHDYLFREILELVDLDFKAAGDVDG 433

Query 485 CSQFHYMPRFVRELNENGKEILCMEVVLRYLLNCAVPLIQKDDLKTMVKMSQYEWQHFAD 544

CSQFH+MPRFVREL +NGKEIL M VL+YLL+ +V LI++ DL+ M+KM+QYEWQ +AD

Sbjct 434 CSQFHFMPRFVRELPDNGKEILAMNEVLQYLLDSSVSLIEEKDLEDMIKMTQYEWQSYAD 493

Query 545 EIKGMVVTYPGKKPSSVRVDQLDRNIDLQKEGDYKFPEIVHFGIRPPQLSYAGNPEYQKA 604

EIKGMVVTYPGKKP SVRVDQLDRNIDLQK GDYKFPEIVHFGIRPPQLSYAGNP+YQKA

Sbjct 494 EIKGMVVTYPGKKPCSVRVDQLDRNIDLQKPGDYKFPEIVHFGIRPPQLSYAGNPDYQKA 553

Query 605 WREYVKYRHLIANMSKPTFEDKRKLEAKEGKLQEIRTQGKMKRDVTIAVSAEGFYRTGIM 664

WR+YVK+RHL+ANMSKPTFEDKRKLE+KE KLQE+RTQGKMKRD+T+AVSAEGFYRTGIM

Sbjct 554 WRDYVKFRHLLANMSKPTFEDKRKLESKENKLQEMRTQGKMKRDITVAVSAEGFYRTGIM 613

Query 665 CDIVQHAMLMPVLICHLRFHNALNVLERSIDYKFKNRGLLQIALTHPSYRQNFGTNPDHA 724

CDI+QHAML+PVL+CHLRFH++LN+LE S++YKFKNR LLQ+ALTHPSYR+NFGTNPDHA

Sbjct 614 CDIIQHAMLIPVLVCHLRFHHSLNILEESVNYKFKNRALLQLALTHPSYRENFGTNPDHA 673

Query 725 RNSLTNCGIRQPEYGDRRIHYMNTRKRGINTLINIMSRFGRQQETESNITHNERLEFLGD 784

RNSLTNCGIRQPEYGDRRIHYMNTRKRGINTLINIMSRFG+QQETESNITHNERLEFLGD

Sbjct 674 RNSLTNCGIRQPEYGDRRIHYMNTRKRGINTLINIMSRFGKQQETESNITHNERLEFLGD 733

Query 785 AVVEFLSSIHLFFSFPDLEEGGLATYRAAIVQNQHLALLAKILNLDQFMLYAHGSDLCHD 844

AVVEFLSSIHLF++FPDLEEGGLATYRAAIVQNQHLA+LAK L LDQFMLYAHGSDLCHD

Sbjct 734 AVVEFLSSIHLFYTFPDLEEGGLATYRAAIVQNQHLAVLAKTLKLDQFMLYAHGSDLCHD 793

Query 845 LELKHAMANCFEALMGALFLDGGIDVADKVFSNTLYRDEPDLLKVWKSLPPHPLQEQEPF 904

LEL+HAMANCFEALMGALFLDGGI+V D+VFS TL++ PDLL+VW +LPPHPLQEQEP

Sbjct 794 LELRHAMANCFEALMGALFLDGGINVVDRVFSETLFKVNPDLLEVWMNLPPHPLQEQEPT 853

Query 905 GDRHWIEKFEILQKLTGFEKAINVEFNHIRLLARAFTDRSVGYTNLTMGSNQRLEFLGDT 964

GDR WI KFE+LQ LT FE+++ ++FNHIRLLARAFTDRSVGYTNLT+GSNQRLEFLGDT

Sbjct 854 GDREWIPKFELLQNLTKFEESVGLQFNHIRLLARAFTDRSVGYTNLTLGSNQRLEFLGDT 913

Query 965 VLQLIASEYLYKYFPEHHEGHLSLLRSSLVNNRTQAVVCDDLGMSQYAIYNNPKPELKTK 1024

VLQLIASEYLYKYFPEHHEGHLSLLRSSLVNNRTQAVVCDDLGMS YA+YNNPK ELKTK

Sbjct 914 VLQLIASEYLYKYFPEHHEGHLSLLRSSLVNNRTQAVVCDDLGMSNYAVYNNPKAELKTK 973

Query 1025 DRADLLEAFIGALYVDKGMEYCEVFCQVTLFPRLQDFIMNQDWNDPKSKLQQCCLTLRTM 1084

DRADLLEAFIGALYVD+G+E+CEVFCQVTLFPRLQDFIMNQDWNDPKSKLQQCCLTLRTM

Sbjct 974 DRADLLEAFIGALYVDQGLEFCEVFCQVTLFPRLQDFIMNQDWNDPKSKLQQCCLTLRTM 1033

Query 1085 DGGEPDIPVYKVIEC--KGPTNTRVYTVAVYFRGRRLASAMGHSIQQAEMNAAKKALEIS 1142

DGGEPDIPVYK + C NTRVYTVAVYFRGRRLASAMGHSIQQAEMNAAKKALEIS

Sbjct 1034 DGGEPDIPVYKSVVCFTNSEVNTRVYTVAVYFRGRRLASAMGHSIQQAEMNAAKKALEIS 1093

Query 1143 HHLFPQLDHQKRVIAKSMKKQKNGGSKTRTSFDSL----RTKSPESYSRNRFKSRSRSRS 1198

LFPQLDHQKRVIAKSMKKQKN + S L RT+SPESYSR RF+

Sbjct 1094 QDLFPQLDHQKRVIAKSMKKQKNKKRRRSRSRSGLKFGFRTRSPESYSRRRFQRSRSRSK 1153

Query 1199 LSSRNSSTPKKKTSKSR 1215

++S + S+P+ TSK++

Sbjct 1154 VTSESDSSPRCSTSKAK 1170

Graphical representation


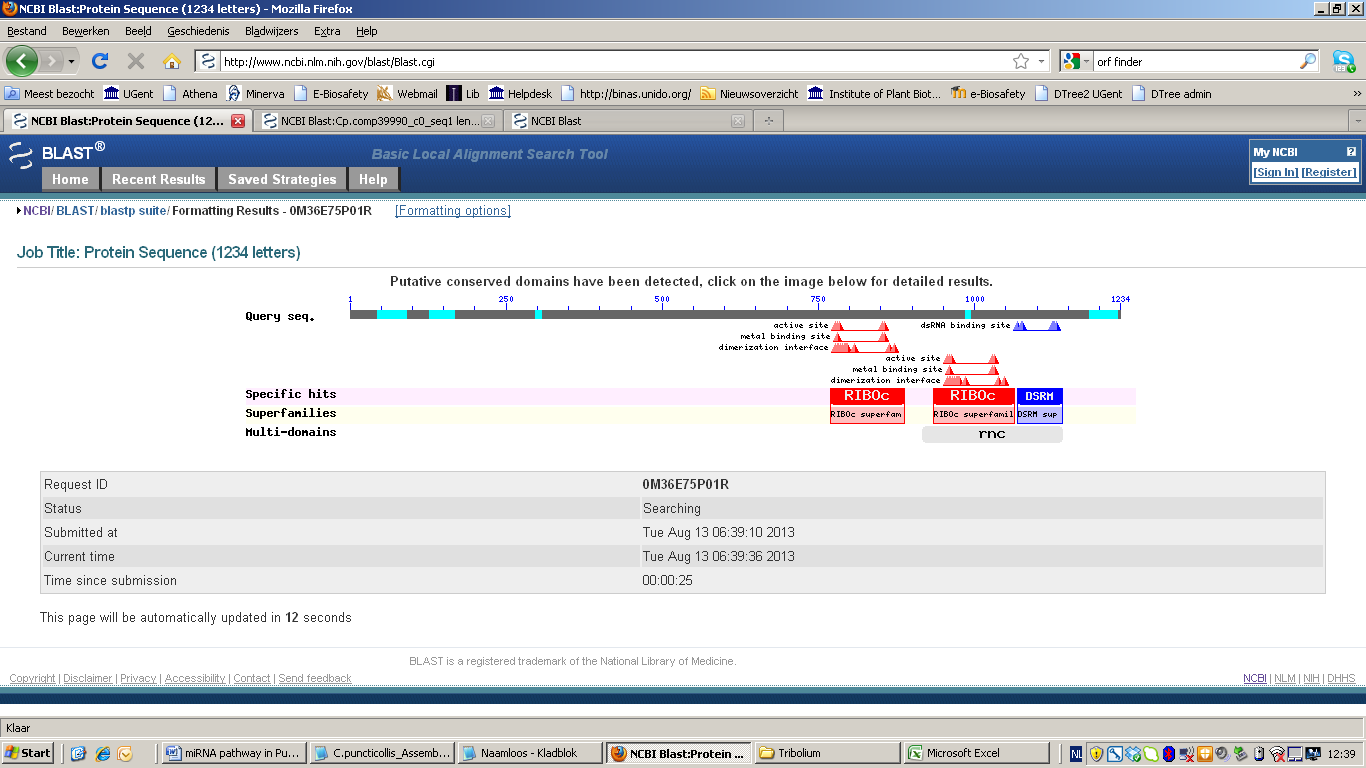


**Loquacious**

>Cp.comp35585_c0_seq1 len=2309

CGCACCCTTTGGCCAGTAGTGTCACGCACGCCCGGCATCCATTTTCGAACAGCGCATGCGATTCCGTTGGTTTCCGATCTTTGGCCTTCGTCGAACGATTTTTGCGAAGCGGGTTTTCATTTCTTCAAGGATTTGCATCTGCCGTTCAATCTCCAATTAATTGATCATTCAATGGAGCCAAACATGGCCATCATACACCCCACCGGCCCAGTGATACAACCATCGGGCGGTCCGATACATCTCAGGCGAGGAAAAAATCCTAAACATTCTATGGCAAGTACAATAACGTTAGCTGAGGAAGCCAAACTTGTCTACTCGCAAGAAATGTCGGGCATAAACAGCAAAACACCGGTGTCAGTTTTGCAAGAACTGTTGAGCCGTAGAGGCTCCACTCCCAAATATGAGTTGGTTCAAATCGAAGGGGCAATACACGAGCCCGTTTTCCGGTACAGGGTGTTTTTGAGCAATGATCTTGTGGCCACCGGCACGGGCAGGTCCAAAAAGGACGCCAAACATGCCGCCGCTAAGAATTTGCTCGATCTTATAGTGGGCAAACAAACGCCCGAGCAAGCAAATCAGACTAACGGAACGCCAGGATCAACCGACATCACGGCGCAGGTGGTATCGCCGTTTGACGACAAAGTGATGGGCAACCCGATCGGGTGGCTGCAGGAAATGTGCATGTCGCGCCGCTGGCCCCCACCACTCTATGAGATGGAGCAGGAAGAGGGGCTGCCGCATGAGCGTCAGTTCACTATCGCCTGTCAGGTGTTGAAGTTCCGCGAGGTCGGCACTGGCAAGTCCAAGAAGCTAGCCAAACGTGTAGCCGCCCATAGAATGTGGCAAGCACTTCAGGACCTGCCCATGGAGGGCACCAATGTACAGACTTTTGAAAGCGATGAAGAGCTTACTGGGAATGCGGTTGACATATCGGTCCGGTATGTGGGCCTTAAAGACAGCAAAATTACCAAACTGAGCACGCAACAGTCGCAGAAAGTATCCCAGTTCCACAAGGGGCTCAAACAATCGGCCGGCAAAAAACTTAATGAGCTTCAGAGTTTGAACATGAAAGAGTTCAACTTTGTTCAGTTCCTGCAAGAAATAGCGTCCGAGCAGAATTTTGAGGTTACCTATGTCGACGTGGAAGAGAAATCTTTGAGCGGACGGTGCCAGTGTCTGGTGCAGCTGTCCACACTGCCAGTGGCGGTGTGTTTCGGGACAGGCAAGACGCCTAAAGACGCTCGCGCCAACGCCGCCCTCAACGCCCTCGAGTACCTGAAGATCATGACCAAGAAGTGAACAGCGACGGCGGCAGAGGCAGGTACGGCAGCGGATACTTCATAATTTTTGTCCCCCCAAAAAATCATATATATATAAAGTCTTTTTTTCTCTGACATTTTCCCTCCGGAAATGCGGCCTGGCGCCTTTATCGCTTTCTTCGTCTTCGGTCGTTTTAGCTGTTTGACTTACGAATTTGTCGAAATTTTTTACTTTTCAGAAGTTTCGAGGTGTTTTAAAAATATTTTAGTTGTTTAATGCCGTTTAAGAGTGAACTTAAACAAGCAAAAATAGTTTTATATATGTTTTTTAAAAAATTGTATTTCTGACATTTTTGTATTTATATTATTTTTTAAAGAATTTGATTAATATATTTTTGATTTTTCTTTTTTTAAATATAAAATGATATTTTTTTCGGCAAAGCGTTGTACAACAAATGGCTCGACTAAAACTAACGGATCACGTGTGTAAAGAGAAGAATAGCGAGTTTTTTACAAGCTATACTTTGCATAGAATTAGTGTTTTTTTTGTGAATAATATACATATATATTTAATTTAGCGTGTTGTGTGACTTTTGCGTAATCGAAACGATGTAACGCATTATAAATAAATATACATTAGTGGCGCGGGTCCACCACAGCGCCGTTGAGCTAACGATATTTAAAGGATGTCCGTTCATTCGTTCGTAACTTTCATAGTAGATAAAATTGTCGTAGGCGTTAGTGTGCATTGTATTTTCGAAATATTAACTATGTATTATTATATAACATATTATAAATGGTGCCCCACGAAGTACGACGTGTAAAGGGAGCATTTATTTAATGCAAAGTATAGAGGTTGTATCCCGTCAATCAATGATTTTTGATTCGATCATAATTAACATATATGTCAATAGATTTAAAAAAAAAAGTGGCGATAAGCAGATCGTCGTGTCGATTTCTTTCCTTTTTAAGATTTTAGAATCTTACCAGTTCTGTTTACTTATATTGATGTGTATGCTAATTACAGTATATTTAAATGGCAAAAAAAAA

Protein: 172 -> 1299 (375AA)

Comparison with *Tribolium* tar RNA binding protein (384AA)

Query 1 MEPNMAIIHPTGPVIQPSGGPIHLRR--GKNPKHSM-ASTITLAEEAKLVYSQEMSGINS 57

M+PNM ++H + + +H RR +N H M A ++L+EEAKL EM+ + +

Sbjct 13 MDPNMTLLHSSSQIHN-----VHPRRKNNRNTLHGMQAERLSLSEEAKL----EMASLPT 63

Query 58 KTPVSVLQELLSRRGSTPKYELVQIEGAIHEPVFRYRVFLSNDLVATGTGRSKKDAKHAA 117

KTPVSVLQELLSRRG+TPKYELVQIEGAIHEP+FRYRVF++NDLVATGTGRSKKDAKHAA

Sbjct 64 KTPVSVLQELLSRRGATPKYELVQIEGAIHEPIFRYRVFINNDLVATGTGRSKKDAKHAA 123

Query 118 AKNLLDLIVGKQTPEQANQTNGTPGSTDITAQVVSPFDDKVMGNPIGWLQEMCMSRRWPP 177

AKNLLD++VGKQ+PEQAN +NGTPG+ DITAQVVSPFDDKVMGNPIGWLQEMCMSRRWPP

Sbjct 124 AKNLLDVLVGKQSPEQANASNGTPGANDITAQVVSPFDDKVMGNPIGWLQEMCMSRRWPP 183

Query 178 PLYEMEQEEGLPHERQFTIACQVLKFREVGTGKSKKLAKRVAAHRMWQALQDLPMEGTNV 237

P YEME EEGLPHERQFTIACQVLKF+EVGTGKSKKLAKR+AAH+MWQALQD+P+EG N+

Sbjct 184 PSYEMEHEEGLPHERQFTIACQVLKFKEVGTGKSKKLAKRMAAHKMWQALQDMPLEGNNL 243

Query 238 -QTFESDEELTGNAVDISVRYVGLKDSKITKLSTQQSQKVSQFHKGLKQSAGKKLNELQS 296

Q ++ DEEL ++ RY GLKDSKI L+ Q +QKVSQFHK LKQS G KL ELQ+

Sbjct 244 PQGYDDDEELAAKMCNLQGRYSGLKDSKIPTLNIQHTQKVSQFHKALKQSNGPKLKELQN 303

Query 297 --LNMKEFNFVQFLQEIASEQNFEVTYVDVEEKSLSGRCQCLVQLSTLPVAVCFGTGKTP 354

LN K+FNF+QFL EIASEQ FEVTYVD+EEK+LSG+ QCLVQLSTLPVAVC+G G TP

Sbjct 304 IVLNSKDFNFIQFLHEIASEQQFEVTYVDIEEKALSGKSQCLVQLSTLPVAVCYGAGATP 363

Query 355 KDARANAALNALEYLKIMTKK 375

K+A++ AALNALEYL+IM+KK

Sbjct 364 KEAQSAAALNALEYLRIMSKK 384

Graphical representation


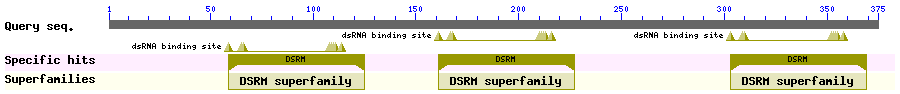


**R2D2**

>Cp.comp37256_c0_seq4 len=5440

GAAAAATGAACCACCGGAGGATCCGGCCGTAGTAACTATGACGTCGTTTAGTTCGTAACATTCGTAAAACCAAACTATACTTCATACCCTATAATTCATCATATTATTCTCCTTGTATTTTGTATATAAAATTCAAAAAACGCAAGTAAAGGACACTACACTACTTAATTTGTATCAAATACAAAATTTTCAGCACAATTTTGCATTTTTACGTTTCTATTTCACGCCCTTAACCAAAGTCTTAACACAGTCACTTTTTTTCTGTTTTATTTTTTCTATGCTGTTTTTATGAATATGTATCAGTATGTCATGAAATTAACCTCAATGATTTTTTTGTTTTTAATGTGTTTTTAAGTTAAGAACGATCATAAATCACTTGTGAAAGATGCAAAACACGAAAACTCCTGTAATGGTATTACAAGAATTAACGGTTAAGAAAGGTTTTGCGCCCCCGGATTATCAGATTGTTAGCTGCAAATCAGGAACCCACGAAAATCGTTTTGATTTTGTCGTGTCCGTTGCTGGGATAGAGGCCGAAGGTAGCGGTTCCTCTAAACAAATAGGTAAACACGAAGCGGCTCACAACGCCCTGATGCAACTCAAGGAGATGGGTATATACGATCCGTCAGAAAATCCCGTAACAGCATTTCAAGCTGCTTTGAAGAATACTGACAGTACTTTTAAGTCTACTGTTAATTGCATTGCAAATTTGCAAAACATTTGTGTGGAGAATAAACTTCCTCCGCCAATATTCAATGAAATTTCTTGTGTTGGTCCACCTCACGCCAGGGAATTTACTTACGAATGTAAAATAGCATCTTTACGCACAGAGGCTAAAGGAAACAGTAAAAAGATGGCTAAACAGTTGGTTGCCAAAGAAATGTTGGAGAAAATCACTGGTGTCCTACCAGAACTCGCTGCACAATTAGAAGAACACCACAACACCCTTACAGATCTAGACTCAAAAGCCTTATTGAGGTACAGCGACTTCCAAGATGTCATTCCAGACAAAAGTATAAAAGTCGACCACATGTCTCATACTTTCAAGAAATTAATGGCACAAAAAAATCTAACATATAAGGATCATTTTGAACATTATTTTGAAGAACCATCGGAAGATAATCTCAAAGAACTTCTTGGTAAACTCGAATTAAGATACGAAATTGATACTTTGCAAACAACGCCATTATTAATAACACTTTGTATTAATACCGACACGAGTCCGTTTACGGTCATTTCCGCTGGATCGACAGAAATGAACGCAAAAGAAGCTCTTCTGAAACAAACTTTTCAGATAATTCAGTGTTTTATGCAAATACCAATTTAAAATGCTAAAATAGATAACAGTTATTAATTTTTGATAGGTAATTGGTGTTGTATTATAACTTACTTTGAAACAAAAGTTTTCATTAACAACCAACCCATTTCCTTATTTTTGGTTCCATCTAAACCAGTTGATTTGAAACAAGATCCTCAAGGGCTTTGGTGGGTCGCGACTCTAGCTTGCTTACTGCAATTTTCAAAAGTAAAGGTAAGGGGAATAAAAAAAAACAACCAGTTGCGTGTAAAAATTTTTATTTTGAATTGACTAGAAATAATTTTTCTGGTAATAAATTGTGCCATAAAAACTCGTTTCAAAACTATGGAGATTACATGATTCAATTAACCACTTCAGATGCAAAGTCGTATTTCTATACATTTGAAGAGATCCTTATCACAAACACACCCATTTTTTTGTATCACAACACAAATACAATCGATAATCAAATATTCGATGGTATTTTTTTAGTAATTTGTAAGTACCCAGTCTTGTACAAGTAATTTATAACTGAATTGCGTTATATACATATTGTGTAAATGTTAAGTCTTCAAAATTACAAAGGTATATGATTTACCATAAAAGAAAAGTATTTGATGCATAAGATCATAAATTTACAAATAAATGATATTTTAATAACGAACTAACAGCTTATGATCCATATCAACCAGGTTGTTCATTACAGTTTGGTTTTACAATCTTTTAGCTATTTCCGTAGACTTTACCTTCGAACACCAAAGTTCTTAAAAAATGCACTACACTATTTTGATTTTATATTTCACATATACATAATATACAAAATGATAAATTTATCTAAACTAAAACCTATGTGATGTTATCAAAACTTCTAAATTAAGTATTAATCTTTGGCACCTTTATAATTAAGGGAAAAAATGAAACTCATTTGTGAATCGAAAACACACACACGTTAAAATGACACTTAACAATTTGAGCCGCATTTTCTGTGCATTTACACCTAGTTAAAAAAAAGTTATTGTCAAGTATAGTAAATAAATAATTAATAAAAAGATTACTTATGATTGGTTTAAATAATTTTGTTTTCTGTCCAAGATACAAAAGTTAAGCAATCGAGATTAATCAAACTATCAATAAATAGTTCTAAAGACTTTTTGCAATATGAAAAGCAAACATTATAAACACCTTTAAAGTATACTTACAATAGTTCCAATAAATAAGATTGTGTCAGGTTAAACATAATTTGTACGCTTACATTTAGAAAATATTCCACAGATATGTTTCAAGATCCTAAAATAAGCAATAGTTAAATAAAAACTAAAAAAAAACTACATATATTTATATGAAATTTCACAGCAATTAGACATACCTATTAGTCTTTCATTTATTTGTCATGTTTAGTTTAATCAGTAATTATGTTTATCGAGCTCTTAGTGCGAAAAAGAACTAGTGCCAGTAATTGTGTAAATAAAGGTATATATAAAAAAAGTTTCATACTATAGAAGTGGAGATAACTTCATAAAAAATGTAACATAACCTATACTCTACACAAAAACTGATGTTACATTTAAAAAGCATAAATATTTGAAAGGGCAATTATAAAAAAAATAAAAGAAATATAAGACATAGAACTAATATGTCAGAATACATAATTTTATCTCAACCTAACGCTAAATTCATCCAAATAAAGCCCATTGTGTAACCAAATGACAAAATTAATTAAATTGAAGATTCTATCGTAATAAGAAATTTTAAACTGATCATTATGACTTTACCAGAATCCTATTGTTTTAAAAACTAAGTTGTATTTAAACAACAGTCGATACATTACTAGTGTGTAAAGGAGACCTCTGTGAATGAAATTGGATTAGTTCCATCACAGTAATGGCTACTTGAACTAAACCTTTGCCTTCCAAAACGTCAGCGGCGCAACAAATGAGATTCTCATCAACTCCAATCTTCCTACATGCTTCTATAAAGTTGTCAACATTTCGTCGACATCTTGCCATCGTCAGTTTCGGAACTGCTGGTGAAGGAACATGGATGCTAGCTACAGACCGCGGCTTAATATGATTAGCCAAATGACATAAAACTACTCCATCTGTTAGAGCCGAAGCTAGATCCTCAGGTAATGTCATTTTTAATCTTGTTTCGATGATATTTCGCAATTGATTTATCAAATCGGTTTCTTCTCGAGCTTTATCAATTTCCCTCCTCATCGTGAATGTTATCTTTTCTGGGGCTATATCCTTATTCCAACTAATATTTCTTCCTGATTTGACACCATTAAAATTTTTCGAAATTAAAGTCGCACTAGTTCCATAGCTACCTCGTGAAGTTTCTTTGTAACTATTGACGTAATTTGAACTACTAGTTCCGGAAGGAGGTACATTATCATGGGACATTACTCCGGCCAATGATTTTATAGGACTCTTCGGTTTAACATATTCCTGTATGTTTAACTTAGTCTCGACAACATTCCCATTAACGTAACGTTTTGGTGAGAAGCTCGTATTCCTAGAGGGAGTTACCTTTTGTACAGGTTTTTTGGTGTTTGCGGTATCATCGAAAATTGTTTTTGGACCTCCAAATGCTGAATTTAATGGTGTCGGATCCGATTTCGATGTGGCCATGGTTTGGCCAATGGTAGAGTGGTGAGTGGGTGTGTTTGGTTCAGTTTTATTACTTTGGTCCTCTGAATCTTTAGTACGGTATATCGAAGGTACGTCGTGTGCTCGTTGTTGTTTTAAGGCTTCTTTATATTCTCTGTAAGTTTGGATCTGTTGAAATAATTTTGGTTTATCTTCGACTCCATCCCCGTTAGAAAAGTTATTCGAACACGGACTGAGACGAACACTGCTAAGATCTTTCACAGCATCCAGCTTATCGTAATCATTGTAAAAGGTACTGTTTTTAGATGGGGGATCTTCGAGATTCTCTGGAGGTGCAGGCGAAATGGTCGAAGGTGTTGATATCGCAGAAACGGTTTCCATTTGATCTGATGATCTCCCCGAAACGGGAGTCGAAGGCCAATTTTTATCATCAACTGCAATTTCTTGTGACCATCGTTTCTCGCAGCAATCACTACTAGTACTATAACCACTATCTACATTGTGTCTTTTATTTTTCAATCTATTGACCAGATGGGGCGGACCTGGGGAACTTGATCCACTTTTCCTCGATTTCCTCCCAAGTGTTCCAGTTCCGCCGCCAGTTTTTCTTTCAATCTTAATCGCTTCCATGTCTAAATATTTGAAGATATGTACCCTACCTCGTTTACACAAACTAGCAGGTGGACTTGTAAGGGGATTTTCGTCTAGTATTAAATCGATAACAGTTGTCATGTTTCGTATCTCTATTGGCAAAGTACTAATTCTATTAGCCCTCAAGTCCAGTCTCACAAGTTTTAGCATAGTAATTTCAACTGGCACGGCCAATAACAGATTACTTCGTAGCACCAGAGACTGAAGGGACTTGAGTTCACCCATACGTGGAGGAAAGTGAGTGATCTGATTACAAGAGGCCTCCAATTCTGTTAACAGCGACATTTTGTACAGCTCATCGGGTAATGACGACAACCTATTGTTCGCAACTAATAAAATCTCTATCGGAAGCTGACATAACTCTCTAGGCAATGCAGTTAATTGATTCCTACTCAAGTCCAAAAAGCTAAGGCATTGCAAATTGATGATAGTGTCCGGAAGTGTCCTGATCGAATTGTGATAGCACTGGAGCCGTTCCAGAAAGTGAAAACCGGTGATAATGTCGGGAAGTTCAGTAAATCGATTTTTTGACAAATCTGCAACAACTGTATCGCTCAAAGTGTATTTTTCCTTCGCTTTTGGAAAATCCTTCAGTTTCCTGTTACTCAATTTCAGTTCACCACTTTGATCGGCTTCTTCCAGTATCCTTTCCAGTTGATTTAGTATGTGTCCGCTTACGTTAGGAACGGACCCTGCCATTGTTCATGGCACGCGAAACGCCGTTTTTTTGTTTCTATCACAAATTTATCAATGAATTTTATAAATAAAAACCAACAAAACTAAATGCACTTCCGCTGTCACATCACTATCACCATAGACTACCACTGTGCTAGCGCATTCAAACATGGAACAAACTTGTAACAAACCACATCCTGTCGTTTTA

Protein RF2: 386 -> 1327 (313AA)

Comparison with *Tribolium* R2D2 (320AA)

Query 2 QNTKTPVMVLQELTVKKGFAPPDYQIVSCKSGTHENRFDFVVSVAGIEAEGSGSSKQIGK 61

QNTKTP MVLQE T+K+GF+PP+Y +V K+GTHEN F + V+VA + G G SKQ+ K

Sbjct 4 QNTKTPAMVLQEFTMKRGFSPPEYILVMSKTGTHENEFHYKVNVANVCGLGFGRSKQVAK 63

Query 62 HEAAHNALMQLKEMGIYDPSENPVTAF--QAALKNTDSTFKSTVNCIANLQNICVENKLP 119

H AA AL L E G+YDPS NPV F Q+ +DS K VN I NL+++C E KLP

Sbjct 64 HNAASKALEILAEQGLYDPSSNPVQEFNAQSHRNESDSPQKPPVNFIGNLKDMCCEFKLP 123

Query 120 PPIFNEISCVGPPHAREFTYECKIASLRTEAKGNSKKMAKQLVAKEMLEKITGVLPELAA 179

P F EIS VGPPH REFTYEC IAS+ T+A N+KK AKQL A+EMLEKI P+LA

Sbjct 124 YPEFKEISDVGPPHCREFTYECCIASITTQATANTKKQAKQLAAREMLEKIRETCPQLAE 183

Query 180 QLEEHHNTLTDLDSKALLRYSDFQ---DVIPDKSIKVDHMSHTFKKLMAQKNLTYKDHFE 236

Q N++ + + +YS+ DV+P++++ ++ S K+ M KN+ ++D F+

Sbjct 184 QFAAESNSILADSHEVIKKYSELSTTLDVMPNRAVLIEDYSTAIKRRMEDKNVCFED-FQ 242

Query 237 HYFEEPSEDNLKELLGKLELRYEIDTLQTTPLLITLCINTDTSPFTVISAGSTEMNAKEA 296

++ ++ L + KL++RY+ID Q +P + DT PFTV++ GS++ NA

Sbjct 243 KQYKLKDKEGLDYIFEKLDIRYQIDLFQESPPVYCALFGLDT-PFTVMAVGSSQENAMAN 301

Query 297 LLKQTFQIIQCFM 309

L+ + +++++ +M

Sbjct 302 LVFEIYRLLEIYM 314

Graphical representation


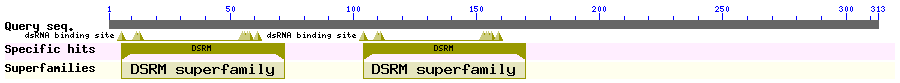


**Pasha**

>Cp.comp38940_c0_seq1 len=2851

TCAACTGGCCGCGAAAGCCTCAATCAATTGATCATTAAATTATTATACTTATACCATCAATATTAACTAGATACTCGCAATCGGCTACATTATTGGACTTAAAACAAATAGTTTATAAGTTACTTAAGGTATCCGTTTTCATACAGGTCAATAATCAAGAATTAAGTGAAATTTTCAACTTTTTCAACTTAAAACACTCCAACCGGTAGATTATTTATACTTATAATACTATAAAATTGCGTGATCCTTTTTATAATTATTCAATATCTACTAAATAATTTGTCAAATTCATCTAAATCCTACAAAAATACGCTAATATTGATTTGGTCAGTTGCAAAACACTATATATATACATCGCATGCGATATTCAGTCAATAAATAAATGAAATGCTTCATATTTTCTTAACGTTAGACGGGTCTAAATTAATTTCACCCTCCGAAGTGTTCACCAACCCTACCGGCCTTACCGTTTTTCTCTTGTCGTTCAATTTTCCCAATTCCATCTTCAATTTATTAAGGATGGCGAAATTCGGTTGATTCACCGCGGCCTTGCTTTGCAACGAAGTAATTTCCTGCTCCTCCATTTTCTTCTCTTTAAAACTTTTGACGGAATGGTTGCCGTACAACCTCAAAAGCGAGCCCCACGACGTTATGCTCGGATGTAGGGCCTGCAAAATCGCTTGCGACGCCCTCTGTTTGCCGTCGCGTTTGTTTTTGCACGCGACCGTCGTCGTATATTTGCCGACGGTCATCGTGAATTCGTTTCCCTGATGCTTGGACGTATTGCCTTGGTAGTGGATTTTAAGGTCGTCCAGACCAAAGTTACGTTGCAAACAGGTGAGGAGAATGTCGTAGGGAGCCGGTTCGGTTGTTTTGGCGCAAAACTCTGCGATACGCGGGTCTTCCACTCGGATTTCGTCGAAAAACGACAGGTCTTGTTCTTTTTCTCGTCTCGTGCGACCTGTTTGATTATCTGTAGTGATCTTTGATTTCATTTCCGGGATGAGTATTTCCAAAGTTGCCCGAGCCGCTTCCGATTTGGCTTGTTTCTTACTAGTACCGTACCCGACACCATATCTCATATCCATTATGATAACGGTTGCCGCGTAAGGTGTAGCAGCGTTTTCAAGTTCTGTAAATTGATAGGTAGGTTGTTTTTTTAAAGCGTGTTGTACGTACTCGTGCAATATGCAAACGTATGACTTCCCGTTAGGGTTCATTATCCATTCTTTTTTTGCATTGGGGTTGTTTTTTTCGGCGCTGTCGATGTCTTTAATAGGAAAAGTTATCAATTTAGTACCGTCGGGTAGTGTGGGTCGTTGCAGTTGATTTTCGTGTTTTCTTCTTTTGGTAAATTTGCGCCTTTCCGACCAAGATTTGAACCTCAAAACGTTTATCGACTGAAATTGGAATATATTTTGGCAATACTCTCTTAGGGCTTCCGCTGTTAAATTTTGAGTTTCTATATTTTCTTGGACGGTTTCTATTCGTGCGTTTGGTAAATTTTCTGGCAGTTTCGTATCGTCCACCCTTTTCAGTTCGACCTGTTCCTTCTTTTCTTCTTCTAGAGCTTTTCTATAGTTCAAACACGGTATTGCGTTTACAGGTATTAAGTGCTTGCGGGCCGAACCGGGCCCCAAAAAATACGGCCTGGCCAAAGTACAAACTCTCGTAGTTTTGTGTAAATACACGGGCATTCCGCTGTTGTGAGTAACTTGTATCCATCCTTCGGGAAGTACGTCGAAATGATTTCGTCCCTTTTCCACCAGCACTATTTTGCTTTTTTCTTCGAAAGGCACTTTCGCATTTTCGTCCGCGTCTAAACCGGCCTCTTCCGCTTTCCTTTTTTTCTTGACCAACGCTTCTTCGAGCATTTTTTCCACCTCTTCGTCGGGAATGTCAGAGTCATATTCGATCGATTCGTCTGATTCCTCGCTGTAATATCGACTTTCGACGTTCGTACTAGAGTTATTTTCATTCAATTGTTCGTACGTTCTGGATAAATTTAAAGGCAATTGCAGTGTATTCCTCGTATCAGATTCAATGCTGGACCCGGCATTCACGACTTGTTTGGATTCACCTATGCAAGTGTCTTTTGGAGTACTCTTTTCCGAACTTTCTGCCCATGCAGCGGTTTTAAACGGGCATTTATTCATTGTTAGGTGCATATCTGAACTATCACTCGCACTCTCCGAGCTGCTCGGGTTATTTTTCAATTGGTGCTTTTTGATACAATTTTTATCGTTAATTGTGTCATCTGTTAAAGAACTATGGCCGTTTAGTGTCTGGCAACCGTTGTAACTTTTAGATCTAATACAAACTATTTCCTCAATCTTAAACGTGTGAGTTTGATCCGTCTTGAAATTCTGATTATCTCTGGGGCAGCTCATAATTTCGGTTTGAGCGTTGGTAAAAGGTTTGGTGTCTATTTCATCAATTCTGTTAGTGCATAAATCCATCATTTCGTCTACCCGGTCGGTTCCCTTTAAATAATTGTCACCATCACTCATCTCTTTCTTCTTTATCGGTGATTACGTTTTCGCACGGTAACAATCAAAAGAATACTTCAACAATATTAAATCAAAAATTGAAATATTCTTTAGAGGGGAACCGAGGAAAACAAGCTACATAGCTCATGTTCCAGGTCATTCCTCTTTTGTCTGTTCTGAAAGAGGCATAACCTCGAACTATTAGAAACGATAAAACATTGATCGTAGCGAAAACACACCGCAGTTACTAGGTGTTTAAAGGGTTCATAAATTTGAATAAGTATTTTTCCAAACACACGGCAACCCCTTCCCAAATGTTACAACAAAAAATCACCCTGCGTTCAGCAGCGCCTGCGCCATCG

Protein: -2510 -> -393 (705AA)

Comparison with *Tribolium* double stranded binding protein (563AA)

Query 194 ESDESIEYDSDIPDEEVEKMLEEALVKKKRKAEEAGLDADENAKVPFEEKSKIVLVEKGR 253

+S++S DSDIPDEE++KMLE+AL KRKA EAGLD + AK PFEEK K+VL+EK +

Sbjct 56 DSEKSFSCDSDIPDEEIDKMLEDALKNNKRKASEAGLD-ESAAKQPFEEKDKVVLIEKSQ 114

Query 254 NHFDVLPEGWIQVTHNSGMPVYLHKTTRVCTLARPYFLGPGSARKHLIPVNAIPCLNYRK 313

NHFDVLPEGWIQVTHNSGMP+YLHK +RVCTL++PYFLGPGS RKH IP++AIPCL+YR+

Sbjct 115 NHFDVLPEGWIQVTHNSGMPLYLHKNSRVCTLSKPYFLGPGSVRKHEIPLSAIPCLSYRR 174

Query 314 ALEEEKKEQVELKRVDDTKLPE---NLPNARIETVQENIETQNLTAEALREYCQNIFQFQ 370

AL+ EK T+ PE +LPNARIETV+ENIE+QNL E +R+Y +FQF+

Sbjct 175 ALDSEK-----------TQTPESSSDLPNARIETVKENIESQNLKPEDVRKYASKLFQFK 223

Query 371 SINVLRFKSWSERRKFTKRRKHENQLQRPTLPDGTKLITFPIKDIDSAEKNNPNAKKEWI 430

+I V+RFKSWSERR+FTK+RKHE QLQRP LP GTKLITFPI+ +++E N NAKKEWI

Sbjct 224 TIKVMRFKSWSERRQFTKKRKHEQQLQRPNLPAGTKLITFPIQPNEASENTNSNAKKEWI 283

Query 431 MNPNGKSYVCILHEYVQHALKKQPTYQFTELENAATPYAATVIIMDMRYGVGYGTSKKQA 490

MNPNGKSYVCILHEYVQHALKKQPTY+FTELENAATPYAATV I DM+YGVGYGTSKKQA

Sbjct 284 MNPNGKSYVCILHEYVQHALKKQPTYKFTELENAATPYAATVSINDMQYGVGYGTSKKQA 343

Query 491 KSEAARATLEILIPEMKSKITTDNQTGRT-RREKEQDLSFFDEIRVEDPRIAEFCAKTTE 549

KSEAARATLEILIPEMKSKITTD +TG + R+++QDLSFFDEIR+EDPR+AEFCAKTTE

Sbjct 344 KSEAARATLEILIPEMKSKITTDAKTGSSASRDQDQDLSFFDEIRIEDPRVAEFCAKTTE 403

Query 550 PAPYDILLTCLQRNFGLDDLKIHYQGNTSKHQGNEFTMTVGKYTTTVACKNKRDGKQRAS 609

P+P+DILLTCLQRNFGL+DL+I YQGNT K++ N+FTMTVGK+T TV CKNKRDGKQRAS

Sbjct 404 PSPHDILLTCLQRNFGLNDLQISYQGNTLKNKKNQFTMTVGKHTATVVCKNKRDGKQRAS 463

Query 610 QAILQALHPSITSWGSLLRLYGNHSVKSFKEKKMEEQEITSLQSKAAVNQPNFAILNKLK 669

QAILQALHP ITSWGSLLRLYGN SVKSFKEKK+EEQEIT LQSKAA+N PNFAIL+KLK

Sbjct 464 QAILQALHPHITSWGSLLRLYGNGSVKSFKEKKLEEQEITLLQSKAAINSPNFAILDKLK 523

Query 670 MELGKLNDKRKTVRPVGLVNTSEGEI--NLDPSNVKKI 705

+EL KL DKR ++P+G+ +E + L SN+K +

Sbjct 524 LELSKLRDKRTQIKPIGVFIPTESDSLPKLSSSNLKNV 561

Graphical representation


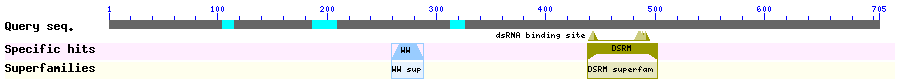


**Exportin-5**

>Cp.comp39084_c0_seq1 len=4465

CGTGACGTCAGTTCGGTCAGAAAATTAGTTAATAATTTTTGCAAAAAAAAACAATAAAATTGACATTTATGTATTATTAAAAAAAGAGACTCTTAACTCCCTCTTCCTTGACCAGATAGTTTTCTGTGGTGTTGGTTTAGATTTTTTTGCGTGCTGCCGGTTGAGGTTTTTAAAATAATCCAATTTTTTTTCAAATTAAACGTGATGGATATTTAAGGCGACGAGGACATATCAGAAAGTTTGTTTAGATGGCTGGCCCAGATGTTGCAGCCTTAGCTGCCGATCTAGCAAGGGCGGTTGAGCTCACTATGAGTTCGGGGGCTTCACAAAGCGACCGCCTAAAAGCATATCAAGCCTGCGAGAGTTTTAAAGAGACATCACCGTTGTGTGCCGAAGCGGGCTTGTATTTGGCTGCAGGCACACAACACTCCCTTATAGCCAGGCATTTCGGGCTTCAGCTAATGGAACATACAGTTAAATATCGGTGGACTCAGATATCGCAACAGGAGAAAATATTCATTAAGGAGAATGCCATGAAACTGTTGGCAGCCGGCGGTATAAGCGATGAGCCACATATGAAAGACGCTCTAAGCCGGGTAGTAGTGGAAATGGTTAAGAGAGAATGGCCTCAGCAATGGCCTGGCCTTTTAGCGGAATTATCTGACGCTTGCTCTTGTGGTGAGACTCAAACCGAATTAGTTTTGTTTGTTTTTTTACGTCTGGTCGAAGATGTAGCCTTATTACAGACATTAGAATCAAATCAAAGACGAAAAGATATTTATCACGCCCTTACGGCCAATATGTCACTGATATTCGAATTTTTCTTGAGATTAATAGAATTACATGTTAGTCAATTGAGAGCTTGCACGGAAAACGAGCCCTCAAAAGCGGCCGCTCACGGTCGTGTTGTACAAGTGGTTTTACTGACGTTGACCGGGTTTGTGGAGTGGGTCAATATGACCCATATAACAGCTCAAAACGGAAGACTGCTGCAGATTTTGTGTTTGTTGGTTAATGATACTGCCTTTCAGTATTCTGCTGCAGAATGTTTGTCTCAGATTGTGAATCGAAAAGGCAAAATTGACGAAAGGAAACCCTTGTTGATGTTACTAAGTGCTGAAACAATGCAGTGTCTTTTGACGGCAGCCAAAAGCACCGCGAATATGTTCAATGAGCAACATTACTTGTTCAAAAAAAAAACTAATACAGGTCTTTGGTGGCTTGACCATTCAGATCTGCACATTGTGGGGCAAAGACCCGTCATTCCAGCCACATAACTTCTCAATGTTCCTGGAGGCCATACTGGCTTACAGTAGCCATTCCAGTTTGTTTTTAGCTCATTTGGCCAATCCTCTGTGGAACAGCATGTTGAAACAAGAACACGTCGCTAGAGATCTCACATTTTTGTCGTTTCTTCCAAAATGGGTGCAGTGTACTGCACCAAAAATAATCAAATTTAATTATCCAGCCGGCAAGAGTCCCACTTTAGGTGAAGACATAAGCGAAACCGTAGCTTACGCGAAAATAGATTTCGACAGTGAAGAGGAGTTCTCGGCTTATTTTTATCGGTGCCGCTCGGACTTTCTCGATTCTTTTAGACAAGCGACTTTGGTAGCCCCGTTGATAACTTTTAATTACGTCGAACAGTGGTTGTTAAAGTGCCTGCAAGTTCCCAATACGACTTTCGGACTCTCGGTGAGCGATCCGGTCTATCAAGAGTGGGAGGCGCTGGCAAACTTCTTGGAGAGCGTGCTAAGCCGGGTGTTGCAAGCCCACGAGAGACCCTCAATTCCGTCAGGATTGCGGTTACTGGAGATGTGCTTAAGGTACCAGCCGGCCGATCCTCTGATAATGTCCACCTTGCTCACGTGCATTTCCGCTTTGTTTGTTTTTCTGAGTATGTCTACTGGACAGATGGCGCCAGCTGCAAATTCTATTGCCGTGTCTGGAGCAGCGCTATTACCCCAAGTGCTTGAAAAAATATTTTCGACCCTAGTCTACAACCCCAACAATCAGTCGAAAGAAGTTAGATCACGACCATTAAAAAACGTCCGGCGACATGCCGCTTCTTTAATGGTCAAAATCGGCAATAAATATCCATTGCTGTTGTTACCAGTATTCGATCAAATCAGAACGACAGTCGAGAATTTGTCAAAGCCTGACGGACCAGCGCAACTGAGTACTCTCGAAAAAGTGACGTTGCGGGAAGCCCTGCTGCTGATTTCCAATCATTTCGCCGACTACGAAAGGCAAAGCGCGTTTGTGGGCGAAGTGCTGAGAGAAGCCAGCGCCCAGTTCATCTCTATTGTTAATTCTGGTGCTTTGAAGGGGGCGGCTTCGTTTATATCCTTTGTTGGGTTGGATAAGCCTTCAGTGCCCGCTAATACCAAAGATATTTGCGGACACAATAGAAGCAATTTGGTGTTTTGCGTTAACTTGGTTTTGGGGGCCATTAAACGTTGCTCGTGGCCCGACGACCCCGAACGAGCCACTAGAGGGGGATTTGTGGTGTCGTTGACGGAATCCGGGAATCCCGTGTGTCGAAATCCCGCAGCACCTCACGTTATCCCTTTACTGCCTCATATGTTGTCACTTATTTGCATTTTTAACGAACTATTCACCAATGAAGCTCAAAATTTGATCCACCAGAGTTATAAGGGCTGTCTGGCAATGCCCGAGACTGAAAAGTCAAATTTACTTGGCCTTATTGGACACTCATCAGATAACGGGGACAATTTAATCATTCAGAGTCCCCTGGAGAGGATGCAGAGGTTTCTCACTTCTCTGCACGAGAGTTTTTACCATTTGATCGGATCAATGGGACCTTCTCTCGGCCGAGACTTGTACAATATTCCTGATTTGGCACTGGCCGTTATTAATAGTGTCTTGGCCTATTTACAGTATGTGCCGGATTATAGAATTAGGCCAATAATACGGGTGTTCCTTAAGTCTTTTATATTCGCTTGCCCGGTGCCGTATTACGAGATCGTTATACTTCCAGTGCTCGGACATATTACACCTATAATATTTAATCGTCTACACTCAAACTGGCAACGGGTAATCGAGTTTCGTAACAGAGAAGTACAAGAGGACAACGCCGACACACAGGAGGTGCTCGAGGATATACTAACGCGGGCCCTGACCAGGGAGTATCTCGACCTTCTCAAGGTGGCTCTAGTTGGAGGAAGCCTCACTCCCGAATCGACTTCAGAAACCATGGAAACCGAGGAGCTCAGTAACTCTCCAACTCCGCCGCCCGTTAGATCTAATCTCACGTCTGAGGTGATTTCTGATCTAGGCTTGATCCTCCTGAGGAACACCAAGACCTGTCAGCCTATAGTGTTGGCAGTGCTCGGAGCTTTATCTTGGATTGACAGCAATGCATCTCTGAAAGCAACTTTACTGACAGGACCCATTGTTCGGCAGCTATCGCAAGATAATTCGTTGAATGGGGAGATGGCGACCCACATCATGGCCGCTATTTTAAATGCTTTAATGTTGCACGGCCAGCACGAGGCGAATCAGGGCTCGCTACTGACCCTAGGTGCGCAGATTTATGAGCTTCTGCGACCGTCATTCGTGGAGGTGTTGGGCGTGATGCAGCAAATTCCGGGCGTGAACCCAGTCGATCTCCAAAAGCTCGACGAGAGGATATCGGGCAGCACCAGTAAGGGAAATAAAGTCGAGAAGGTCAAGAAGGATCTATTTAAGAAAATAACAGCTAACCTTATCGGCAGAAGCGTGGGTCAGCTGTTCAAGAAGGAGGTAAAAATACAGGACTTGCCGCAGCTTGTGATACCCAAAAAGGCCGAAAAATCCGAAGTAATTGGAGACCTAAGGAACATTTTTAGTTAGTACTTTTATTACTTATTATTTTTTGTATCGTATTGTAATTGAACGGTACATTCCTTTGTGCTCTTCAATCATGACTGGAAACTCTAATAAATGACGCCCCTCGGGTGAATAAGACTTTAGATATTAATAATACAACGTATCGTGATTTTTGTAAAAATACTATCAACTTTATTTATTTTGTGTTGGAATAAAATTGTCCATGCATAGTTCTGGTTTACTGACCTCCGAAATAAGAACATAATAATTGGAAACTAACCGAAAAAAAGAACCAAATTTGATTGGTTTGAAAGTACAATTAAGGCTGATTCTCAGTAGACACTCCGTCTCTGTTCCATAACCATTACATATCTGTTAAAGTGTGCATACGTACAGTACTTTTCAGCTAATAATATTTTATTTAAAAGAAACGGAGATGAAACGTATAGTGAGGTTCAACCTTTATTGATCAGTTTCGACAGATTTCGGTCCAGATCTATGTTCTGAGGGATTATCGAAATTGATTCTTAATGACTAAGAGGGTACTAATGTGCATTTGTGTACATATTTCTTTTTTGTTAGCTTTTATTTCGTTATTATGTTTTACTATTAAATTGAATTTTTTTCCAAAAAA

Protein: RF3 249 -> 1277 (342AA), RF1 1171 -> 3855 (894AA)

Comparison with *Tribolium* chromosome region maintenance protein 5/exportin (1204AA)

Range 1: E=9e-143; bits=472

Query 249 MAGPdvaalaadlaravELTMSSGASQSDRLKAYQACESFKETSPLCAEAGLYLAAGTQH 428

MAGPDVAALAADLARAVELTMS+GASQ+DRLKAY ACESFKETSPLCAEAGLYLAAGTQH

Sbjct 1 MAGPDVAALAADLARAVELTMSTGASQTDRLKAYNACESFKETSPLCAEAGLYLAAGTQH 60

Query 429 SLIARHFGLQLMEHTVKYRWTQISQQEKIFIKENAMKLLAAGGISDEPHMKDALSRVVVE 608

SLI+RHFGLQLMEHTVKYRWTQISQQEKIFIKENAMKLLAAGGISDEPHMKDALSRV+VE

Sbjct 61 SLISRHFGLQLMEHTVKYRWTQISQQEKIFIKENAMKLLAAGGISDEPHMKDALSRVIVE 120

Query 609 MVKREWPQQWPGLLAELSDACSCGETQTelvlfvflrlvedvALLQTLESNQRRKDIYHA 788

MVKREWPQQWPGLL+ELS+ACSCGE QTELVL VFLRLVEDVALLQTLESNQRRKDIYHA

Sbjct 121 MVKREWPQQWPGLLSELSEACSCGEIQTELVLLVFLRLVEDVALLQTLESNQRRKDIYHA 180

Query 789 LTANMSlifefflrlielHVSQLRACTE-NEPSKAAAHGRVVQVVLLTLTGFVEWVNMTH 965

LTANM++IF+FFLRLIELHV+Q R C E N K+ AHGRVVQVVLLTLTGFVEWV+M+H

Sbjct 181 LTANMAVIFDFFLRLIELHVNQFRICGETNNTPKSTAHGRVVQVVLLTLTGFVEWVSMSH 240

Query 966 ITAQNGRLLQILCLLVNDTAFQYSAAECLSQIVNRKGKIDERKPLLMLLSAETMQCLLTA 1145

I AQNGRLL ILCLL+ND AFQY AAECLSQIVNRKGK+DERKPLL+L + E +QCL++A

Sbjct 241 IMAQNGRLLHILCLLLNDLAFQYPAAECLSQIVNRKGKVDERKPLLLLFNDEPIQCLVSA 300

Query 1146 AKSTANMFNEQHYLFKKK 1199

+K+ + +EQHYLFKKK

Sbjct 301 SKNPGAILDEQHYLFKKK 318

Range 2: E=0.0; bits=1316

Query 1192 KKKLIQVFGGLTIQICTLWGKDPSFQPHNFSMFLEAIlaysshsslflahlaNPLWNSML 1371

KKKL+QV GGLT Q+ LWGKD +P+NFS FLEAILA+SSH SL L+H+ANPLWNSML

Sbjct 316 KKKLVQVLGGLTTQLVVLWGKDSISRPNNFSAFLEAILAFSSHQSLTLSHMANPLWNSML 375

Query 1372 KQEHVARDLTFLSFLPKWVQCTAPKIIKFNYPAGKSPTLGEDISETVAYAKIDFDSEEEF 1551

K EH++RD FLS++P+WVQCTAPKI+KFNYPA K D AYAKIDFDSEEEF

Sbjct 376 KHEHISRDPVFLSYIPQWVQCTAPKIVKFNYPASK--VQNTDTGGAAAYAKIDFDSEEEF 433

Query 1552 SAYFYRCRSDFLDSFRQATLVAPLITFNYVEQWLLKCLQVPNTTFGLSVSDPVYQEWEAL 1731

S YFYRCRSDFLDSFRQAT+VAPL+TFNYVEQWL+KCLQVPN T GL +SDP++ EWEAL

Sbjct 434 STYFYRCRSDFLDSFRQATVVAPLVTFNYVEQWLMKCLQVPNVTSGLVLSDPLFHEWEAL 493

Query 1732 ANFLESVLSRVLQAHERPSIPSGLRLLEMCLRYQPADPLIMSTLLTCISALFVFLSMSTG 1911

+ FLES+LSRVLQA ERPSI SGLRLL++CL YQP DPLI+STLLTCISALFVFLSMSTG

Sbjct 494 STFLESILSRVLQAQERPSIASGLRLLQLCLVYQPVDPLILSTLLTCISALFVFLSMSTG 553

Query 1912 QMAPAANSIAVSGAALLPQVLEKIFSTLVYNP-NNQSKEVRSRPLKNVRRHAASLMVKIG 2088

QMAP ANS+A SGAALLPQVL+KIFSTLVY P + QSK+ RSR +KNVRRHAASLMVKIG

Sbjct 554 QMAPTANSVAASGAALLPQVLDKIFSTLVYAPPDEQSKDTRSRAVKNVRRHAASLMVKIG 613

Query 2089 NKYPLLLLPVFDQIRTTVENLSKPDGPAQLSTLEKVTLREALLLISNHFADYERQSAFVG 2268

NKYPLLLLPVFDQIR TVENLS+ D A LSTLEKVTL+EALLLISNHF DY+RQS FV

Sbjct 614 NKYPLLLLPVFDQIRATVENLSRSDSVAGLSTLEKVTLQEALLLISNHFCDYDRQSNFVR 673

Query 2269 EVLREASAQFISIVNSGALKGAASFISFVGLDKPSVPANTKDICGHNRSNLVFCVNLVLG 2448

EVL EA+AQ+ IV SGA + A+ FISFVGLD P V + + GHNRS++VFC+NL+LG

Sbjct 674 EVLAEANAQWRLIVASGAFESASKFISFVGLDTPPVAPHADNPHGHNRSSIVFCINLLLG 733

Query 2449 AIKRCSWPDDPERATRGGFVVSLTESGNPVCRNPAAPHVIPLLPHMLSLICIFNELFTNE 2628

AIKRCSWP+DPERATRGGFVV+LTESGNPVCRNPAAPHV+PLLP +LSLI +FNELFT E

Sbjct 734 AIKRCSWPEDPERATRGGFVVALTESGNPVCRNPAAPHVVPLLPDILSLIRVFNELFTCE 793

Query 2629 AQNLIHQSYKGCLAMPETEKSNLLGLIGHS-SDNGDNLIIQSPLERMQRFLTSLHESFYH 2805

AQNLIH+SYKGCL M ETEKSNLLGLIGHS D G+ +QSP+ERMQRFL LHES YH

Sbjct 794 AQNLIHESYKGCLGMLETEKSNLLGLIGHSVGDLGELQAVQSPMERMQRFLFGLHESCYH 853

Query 2806 LIGSMGPSLGRDLYNIPDLALAVINSVLAYLQYVPDYRIRPIIRVFLKSFIFACPVPYYE 2985

+IGSMGPSLGRDLY +PD+ LA+INSVLA LQ +PDYR+RPIIRVFLK FI++CP P+YE

Sbjct 854 MIGSMGPSLGRDLYTLPDIGLAIINSVLACLQCIPDYRMRPIIRVFLKPFIYSCPTPFYE 913

Query 2986 IVILPVLGHITPIIFNRLHSNWQRVIEFRNREVQEDNADTQEVLEDILTRALTREYLDLL 3165

V+LP++ HI P++ +RLH+ W +V EFRNRE QEDNADTQEVLEDILTRALTREYLD+L

Sbjct 914 AVLLPIVAHIAPLMLSRLHAKWLQVNEFRNREGQEDNADTQEVLEDILTRALTREYLDVL 973

Query 3166 KVALVGGslt--pestsetmeteelsnsPTPPPVRSNLTSEVISDLGLILLRNTKTCQPI 3339

KVALVGG LT + + E + + PPP RSN+T+EVISDLGL+LLR+ KTCQ I

Sbjct 974 KVALVGGGLTPETNTENMETEDLSMDSPTPPPPTRSNMTTEVISDLGLVLLRSEKTCQSI 1033

Query 3340 VLAVLGALSWIDSNASLKATLLTGPIVRQLSQDNSLNGEMATHIMAAILNALMLHGQHEA 3519

VLAVLGALSWIDSNASLKAT LTGPIVRQL D+SLNGEMA HIMA++LNALMLHGQHEA

Sbjct 1034 VLAVLGALSWIDSNASLKATFLTGPIVRQLVSDSSLNGEMAAHIMASVLNALMLHGQHEA 1093

Query 3520 NQGSLLTLGAQIYELLRPSFVEVLGVMQQIPGVNPVDLQKLDERISGSTSKGNkvekvkk 3699

NQGSLLTLGAQ+YE+LRP+F+EVLGVMQQIPGVNPVDLQKLDERISGSTSKGNKVEKVKK

Sbjct 1094 NQGSLLTLGAQMYEMLRPTFLEVLGVMQQIPGVNPVDLQKLDERISGSTSKGNKVEKVKK 1153

Query 3700 dlfkkITANLIGRSVGQLFKKEVKIQDLPQLVIPKKAEKSEVIGDLRNI 3846

DLF+KIT NLIGRS+GQLFKKEVKI DLP L KK + EV DL+N+

Sbjct 1154 DLFRKITGNLIGRSMGQLFKKEVKIHDLPSLAFSKKPQPKEVTPDLQNV 1202

Graphical representation

No putative conserved domains have been detected.

**Ago-1**

>Cp.comp34373_c0_seq6 len=5036

GGGGACACGGCAACGCCGTTTTGAACATTGTGCATTTCGTAAACGTCACTAGTAAAATTTTCCATTAAAATTGTCCTGTGCGTTCGATAGTTTCACCCACGTACCTACATATAGCGTAAAATATAGATTTTTACCTTCCTCGGGGTTCGCGACGCAAAAACGCCCACTTTCCGTTTTTGTACAAAAATCCAATTATTTCTCGGAAACCACGCGAAAATATCGTGGGACACTGGCAACGTCGCCGGAACAGCGGAGCACAACAATTGCAAATGGCCGACGTGGTTCTCAGAGACCATAGACGATTTGTTGAATTGAAGTAGTGTGTGGTGTATGTGTGCTTGTGTGACTAATTTATTTATTTAAACATGTACCCCGGAACTTCTGGACCAGGAACGTCAGGCGCAGGCCCCATGGGCCCAACGACGACAGCGGTGGCCTTACCGGGCACTGCAGCCAGTACCAGTTTGACAACGGTATCGCCCCCGGCGGAGCCGCCGATGTTCCAATGCCCACGAAGGCCCAACTTGGGCCGTGAGGGCCGCCCTATCGGCCTTAAGGCCAATCATTTCCAAATATCGATGCCACGTGGCTTTGTCCACCATTACGATATCAACATTCAGCCCGACAAGTGTCCGAGAAAGGTCAACAGAGAAATCATCGAAACAATGGTTAAATCGTATGGGAAGATATTTGGCAACCTTAAGCCCGTCTTCGACGGTCGCAACAACCTCTACACTCGCGATCCTCTTCCTATTGGCAATTCCCGGGAGGAACTGGAGGTTACTCTACCTGGTGAAGGCAAGGACAGGTTGTTCCGAGTCAGCATCAAGTGGGTGGCCCAAGTGTCCCTGTACGGGCTGGAAGAGGCACTCGAGGGTAGAACCCGACAAATTCCATACGAAGCCATACTGGCTTTGGACGTTGTGATGCGTCATTTGCCTTCTATGAGCTACACACCTGTCGGGAGAAGTTTTTTCAGTAGTCCCGAGGGCTATTACCATCCATTGGGTGGCGGCAGGGAAGTGTGGTTTGGTTTTCATCAGTCGGTACGTCCTAGTCAGTGGAAAATGATGCTCAATATTGACGTATCTGCGACGGCATTTTACAAAGCGCAACCTGTGATAGAATTTATGTGTGAAGTTTTGGATATTCGGGACATTAATGAGCAACGCAAACCCCTGACCGACAGTCAGCGGGTCAAGTTTACGAAAGAAATCAAAGGACTAAAGATTGAGATAACGCATTGCGGGGCGATGAGGAGGAAATACAGGGTGTGCAACGTCACGAGAAGACCAGCACAGATGCAATCATTTCCATTGCAGTTAGAAAACGGACAAACCGTCGAATGCACGGTGGCGAAATATTTCTTGGACAAATACAAGATGAAGTTACGGTACCCTCATTTGCCATGTCTGCAAGTGGGGCAGGAACACAAGCACACTTACTTACCATTAGAGGTCTGCAATATTGTCGCCGGACAACGGTGTATCAAAAAACTGACCGATATGCAGACTTCCACTATGATTAAAGCTACAGCCAGGTCAGCTCCGGATCGGGAACGAGAAATAAACAACCTCGTCCGGAGGGCAGACTTCAACAACGACGAATACGTACAGGAGTTTGGACTGACCATTTCCAACAACATGATGGAGGTCCGGGGCAGGGTCCTGCCGCCACCCAAGCTCCAGTACGGCGGCAGAGTGGCGTCCCTCAGCGGACAGAGCAAACAGCAAGCTTGTCCCAATCAGGGGGTGTGGGACATGCGCGGCAAGCAGTTTTTCACGGGCGTCGAGATAAGGGTGTGGGCGATAGCGTGTTTTGCGCCCCAGAGGACCGTACGAGAAGACGCCTTGAGAAACTTTACGCAACAACTGCAGAAGATATCCAACGACGCGGGGATGCCCATCATTGGGCAGCCATGTTTCTGCAAATACGCCACCGGGCCGGACCAAGTGGAACCGATGTTCCGCTACCTAAAGACAACATTCCAGTCCTTGCAGTTAGTTGTTGTCGTTTTGCCCGGCAAAACTCCAGTATACGCCGAAGTAAAACGCGTCGGAGACACGGTATTAGGGATGGCCACCCAATGTGTTCAAGCGAAAAACGTAAACAAGACATCGCCCCAGACTTTGTCCAATCTGTGCCTGAAAATAAACGTGAAACTGGGCGGCATCAACAGTATTCTAGTGCCATCGATCAGACCAAAGATATTTAACGAGCCCGTCATATTCTTGGGAGCGGACGTCACTCATCCTCCGGCCGGCGACAACAAAAAACCGTCGATAGCGGCAGTGGTAGGCTCGATGGACGCTCATCCGTCCCGGTACGCGGCCACCGTCAGGGTACAGCAACACCGTCAGGAAATCATCCAGGAGCTCAGCTCTATGGTCAGGGAACTGCTGATCATGTTTTACAAATCGACCGGCGGCTACAAACCGCACCGTATCATTTTGTATCGGGACGGAGTGTCCGAGGGCCAATTCCTTCAACTGTTGCAGCATGAATTGACCGCCATCCGCGAAGCTTGCATCAAACTTGAGGCGGACTACAAGCCGGGCATTACTTTCATTGTGGTGCAGAAGAGGCACCACACTAGATTGTTTTGCGCCGATAAGAAGGAACAAAGTGGAAAGAGCGGTAACATACCGGCCGGTACGACGGTCGATGTGGGTATTACCCACCCGACCGAGTTTGATTTTTACCTGTGCAGCCATCAAGGTATTCAGGGCACGTCGAGACCATCCCATTACCACGTGTTGTGGGACGATTCGCATTTGGACTCTGACGAATTACAATGTTTGACATATCAACTTTGCCACACATACGTTCGGTGTACCCGATCGGTTTCTATTCCGGCTCCCGCTTATTACGCCCATTTGGTGGCGTTCCGAGCCAGGTACCATCTCGTTGAGAAGGAACACGACAGTGGAGAAGGTTCCCACCAGTCTGGTTCGTCGGAAGACCGAACGCCCGGGGCAATGGCGAGGGCCATCACAGTCCACGCCGACACCAAGAAGGTTATGTATTTCGCTTAGGGTTCGCGGTTAATCGGGATGAGCTTCGAAAAGTACCACAGGTAGAACTGCGGACGTCTGTCCGAGAACAAGCGGAGTTTTTCGTTATTCTGATCGCGGATGTTGCACTTTTTTAAAATTATTATTTTAAGGAAGTGCAGCGGTATTTTGTTGCCAACTAAATTTATAACGAGCACGATAGCTTTTACACTATCGAGTAGATAAAAATGTCGATTTTGACAAACTGTATATCTTTTTAAGGTGATGGCCGCTAATCGTTAAGATTGCTGTCGATATATCCAAAAAAATATATAGACGGCAATCTGTTCATCGGTTAAATTGCACTAAAAAACTTCTCCAGGGATCGTATATTGGCGGCGTTTTCCAACCTTTCACATTTATTTATTAATGTTAAAAATGAGGTAGGTTATTATTTGTTGCAATCGTGCCAGAAATTGAAAAACTACTTTGATACACTTTAGTACCTTTACGTCTTTCTCTATTCCATGTAATCGAGCATTGTACCGAGCTTTAATGAGTTTTTTTAAGTTAATTTTCTAGCCTTAAGGTAATTTGTGTCAAAATATTTGATAGTAGCATTGCAGCCAAAAAAACACACATAGACACACACATACAGAGTTATTGTTTAAAAGACTATACACATTCATATCTTATTATTATGACCGATAATGCTGAATTAAAAGGGCTCACATTTTTTTTCTTACATACAAATAGAGTTATTTTTTAAACGGAAGACTAATACACATGACCGATAATGCTAAATTAAAAGAGCTAACATTTTTCTTAAGCCTAGACAATAAATTTCAATTGATAGGTTACAGGCCGAGGCATTCCTTACAAAGTGGGGATTATATTATTTTTTTATCATATTATGGTATTATATTTTGAGTGTTAATAACCTGTAAGCTTTTTTTTACCGTTGTCGGCGGCGGTTAGACCTAAGTTAGTTTAGTTTCAGAATAGAGCACTTGTCCATTGTTATTTTTTAACAAATGACGTTTACACACTCATATTTACCAAACATTTCGTCGCAAACTAGATTAGGATTTACTAAATGTAAAATTTATATATATTGTTCTCTTTGGGTGATGTGTCGTTATTTAGCGTTAAGTGACGTTCCACACATTCACCGACGACCAAAATGATCTCGAATTTACACTCCTCCCACCAAACTGTTCGTTCAGGTAGGTAAATGTGTGGAACCGACGTAGGTAGCCAAAATGTACTTGAAAGCCGGGAATATTCTATGGAAGTACAAAAATATATATATATTTTTTTCCTATTGTATCTTTAATGTGGTTGGTACATTTGAACTACTGATTACTAATATTACATTATTTGTCCTCTTCAGTTGAATTTCAGGGTTAAAAGTTTCGAGTGTTGCGCACATTGGTCGACGGTAGTTCAAATGTACAACGCCTCAATATTTTTCTGCTCGGGGAGTAGAAAATCGTTGACGCTCGTGCATCACTTCAAAGGTGTCAGTCTTGCAGCGGTTAGTAGTAGGTATAGTTTATAATTTGTATCCTGCCCACAATGCAATCGACCCGTATTGTCATATCACTTGTTTTTAAAATAATATTTTTGCTGTTATTGCAGGCGCAGAGTAAAAAAAATTGGGGGATTCCATCTTTTTAAACCATTCGGTTTCCTTTGGTTAATTAGAAGAGTAATTGGTTGGAGACGGTTCGAAACATTTTTCTGTGCCTCGGTACTAACAGTCTCCCAAATATTAAATATTATGTAAGCTACCTTAAAATTGGCATGTTTCTGTGATAATAATTTCTGGTCCTCCGAAAACAATTATTCCCTATATTATTAAGCCTTTTATGAGTCTAGTAGTTTCCCTTTATTTTTTTTACTTTGTATCCATTTTGTTGTCACTTCGAATTTTTTTGTATATGTAGTATTGCCAATGAATATTTTATTTAAGATTTTTTTTATTAGAGCTTTTTGTCAGGTATTTTTATTGGCCGAATAAATTATTGATTCAAAAAAAAA

Protein: 366 -> 3035 (889AA)

Comparison with *Tribolium* PREDICTED: Argonaute-1 (912AA)

Query 17 GPTTTAVALPGTAASTSLTTVSPPAEPPMFQCPRRPNLGREGRPIGLKANHFQISMPRGF 76

G +TAVA+ G A ST+L TV P +PP+FQCPRRPNLGREGRPIGLKANHFQ++MPRGF

Sbjct 44 GTASTAVAVVG-ATSTALATVPPTTDPPVFQCPRRPNLGREGRPIGLKANHFQVTMPRGF 102

Query 77 VHHYDINIQPDKCPRKVNREIIETMVKSYGKIFGNLKPVFDGRNNLYTRDPLPIGNSREE 136

VHHYD++IQPDKCPRKVNREIIETMV +YGKIFGNLKPVFDGRNNLYTRDPLPIGNSREE

Sbjct 103 VHHYDVSIQPDKCPRKVNREIIETMVHAYGKIFGNLKPVFDGRNNLYTRDPLPIGNSREE 162

Query 137 LEVTLPGEGKDRLFRVSIKWVAQVSLYGLEEALEGRTRQIPYEAILALDVVMRHLPSMSY 196

LEVTLPGEGKDRLFRV+IKWVAQVSLYGLEEALEGRTRQIPYEAILALDVVMRHLPSMSY

Sbjct 163 LEVTLPGEGKDRLFRVTIKWVAQVSLYGLEEALEGRTRQIPYEAILALDVVMRHLPSMSY 222

Query 197 TPVGRSFFSSPEGYYHPLGGGREVWFGFHQSVRPSQWKMMLNIDVSATAFYKAQPVIEFM 256

TPVGRSFFSSPEGYYHPLGGGREVWFGFHQSVRPSQWKMMLNIDVSATAFYKAQPVIEFM

Sbjct 223 TPVGRSFFSSPEGYYHPLGGGREVWFGFHQSVRPSQWKMMLNIDVSATAFYKAQPVIEFM 282

Query 257 CEVLDIRDINEQRKPLTDSQRVKFTKEIKGLKIEITHCGAMRRKYRVCNVTRRPAQMQSF 316

CEVLDIRDINEQRKPLTDSQRVKFTKEIKGLKIEITHCG MRRKYRVCNVTRRPAQMQSF

Sbjct 283 CEVLDIRDINEQRKPLTDSQRVKFTKEIKGLKIEITHCGTMRRKYRVCNVTRRPAQMQSF 342

Query 317 PLQLENGQTVECTVAKYFLDKYKMKLRYPHLPCLQVGQEHKHTYLPLEVCNIVAGQRCIK 376

PLQL+NGQTVECTVAKYFLDKYKMKLRYPHLPCLQVGQEHKHTYLPLEVCNIVAGQRCIK

Sbjct 343 PLQLDNGQTVECTVAKYFLDKYKMKLRYPHLPCLQVGQEHKHTYLPLEVCNIVAGQRCIK 402

Query 377 KLTDMQTSTMIKATARSAPDREREINNLVRRADFNNDEYVQEFGLTISNNMMEVRGRVLP 436

KLTDMQTSTMIKATARSAPDREREINNLVRRADFNND YVQEFGLTISNNMMEVRGRVLP

Sbjct 403 KLTDMQTSTMIKATARSAPDREREINNLVRRADFNNDPYVQEFGLTISNNMMEVRGRVLP 462

Query 437 PPKLQYGGRVASLSGQSKQQACPNQGVWDMRGKQFFTGVEIRVWAIACFAPQRTVREDAL 496

PPKLQYGGRVASLSGQ QA PNQGVWDMRGKQFFTGVEIRVWAIACFAPQRTVREDAL

Sbjct 463 PPKLQYGGRVASLSGQ---QAMPNQGVWDMRGKQFFTGVEIRVWAIACFAPQRTVREDAL 519

Query 497 RNFTQQLQKISNDAGMPIIGQPCFCKYATGPDQVEPMFRYLKTTFQSLQLVVVVLPGKTP 556

RNFTQQLQKISNDAGMPIIGQPCFCKYATGPDQVEPMFRYLK+TFQSLQLVVVVLPGKTP

Sbjct 520 RNFTQQLQKISNDAGMPIIGQPCFCKYATGPDQVEPMFRYLKSTFQSLQLVVVVLPGKTP 579

Query 557 VYAEVKRVGDTVLGMATQCVQAKNVNKTSPQTLSNLCLKINVKLGGINSILVPSIRPKIF 616

VYAEVKRVGDTVLGMATQCVQAKNVNKTSPQTLSNLCLKINVKLGGINSILVPSIRPKIF

Sbjct 580 VYAEVKRVGDTVLGMATQCVQAKNVNKTSPQTLSNLCLKINVKLGGINSILVPSIRPKIF 639

Query 617 NEPVIFLGADVTHPPAGDNKKPSIAAVVGSMDAHPSRYAATVRVQQHRQEIIQELSSMVR 676

NEPVIFLGADVTHPPAGDNKKPSIAAVVGSMDAHPSRYAATVRVQQHRQEIIQELSSMVR

Sbjct 640 NEPVIFLGADVTHPPAGDNKKPSIAAVVGSMDAHPSRYAATVRVQQHRQEIIQELSSMVR 699

Query 677 ELLIMFYKSTGGYKPHRIILYRDGVSEGQFLQLLQHELTAIREACIKLEADYKPGITFIV 736

ELLIMFYKSTGGYKPHRIILYRDGVSEGQFLQLLQHELTAIREACIKLE+DYKPGITFIV

Sbjct 700 ELLIMFYKSTGGYKPHRIILYRDGVSEGQFLQLLQHELTAIREACIKLESDYKPGITFIV 759

Query 737 VQKRHHTRLFCADKKEQSGKSGNIPAGTTVDVGITHPTEFDFYLCSHQGIQGTSRPSHYH 796

VQKRHHTRLFCADKKEQSGKSGNIPAGTTVDVGITHPTEFDFYLCSHQGIQGTSRPSHYH

Sbjct 760 VQKRHHTRLFCADKKEQSGKSGNIPAGTTVDVGITHPTEFDFYLCSHQGIQGTSRPSHYH 819

Query 797 VLWDDSHLDSDELQCLTYQLCHTYVRCTRSVSIPAPAYYAHLVAFRARYHLVEKEHDSGE 856

VLWDDSHLDSDELQCLTYQLCHTYVRCTRSVSIPAPAYYAHLVAFRARYHLVEKEHDSGE

Sbjct 820 VLWDDSHLDSDELQCLTYQLCHTYVRCTRSVSIPAPAYYAHLVAFRARYHLVEKEHDSGE 879

Query 857 GSHQSGSSEDRTPGAMARAITVHADTKKVMYFA 889

GSHQSGSSEDRTPGAMARAITVHADTKKVMYFA

Sbjct 880 GSHQSGSSEDRTPGAMARAITVHADTKKVMYFA 912

Graphical representation


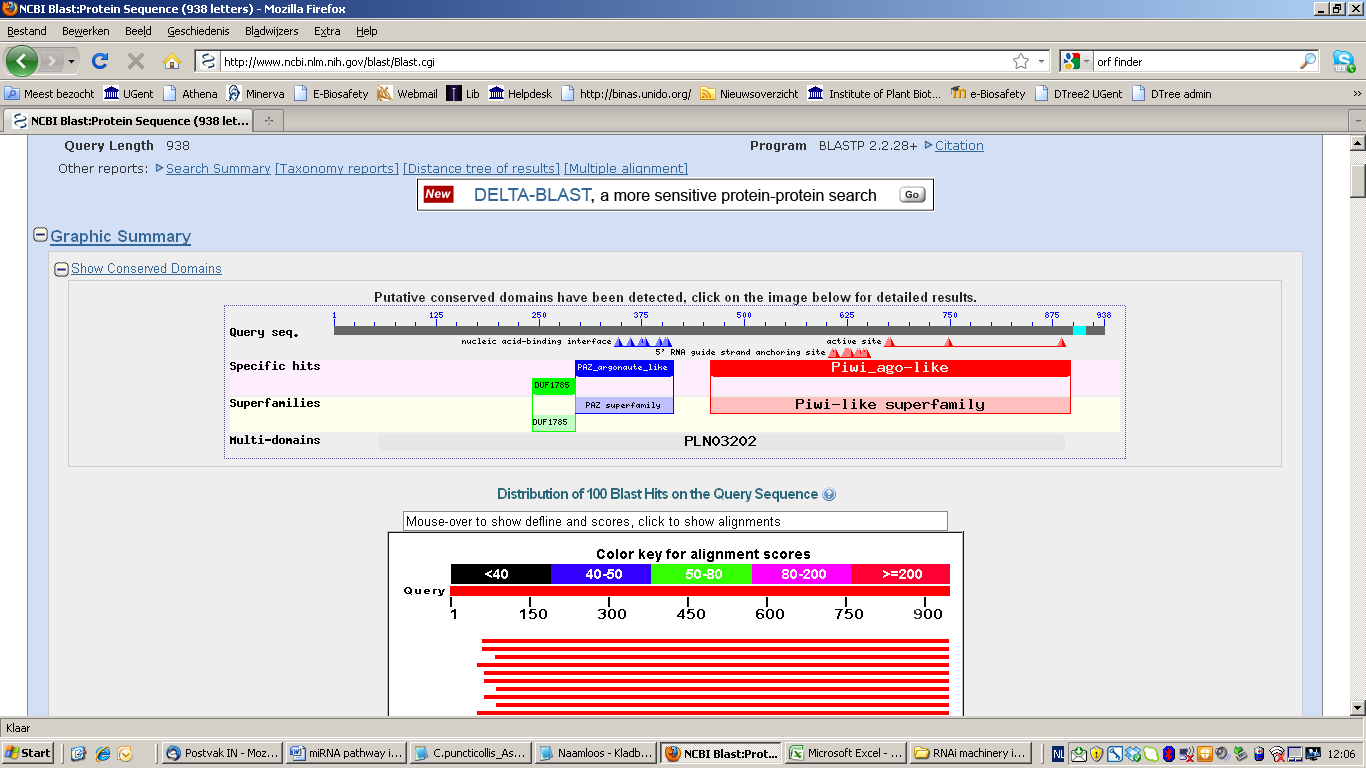


**Ago-2**

>Cp.comp38067_c0_seq1 len=3069

ATCCAGGTCATAGTAGTTCAAACGTACCATCACTTACACCCATATTTATTATTTGGCTATAGTGCAATTTGTCAAGTTTGTGAAGGCACGTGTCAAACCGTATTTGTTCCGCTATATATTTTTTATTTACTAACCAGACGTTATTTTAATTAAAAATTTTTGGAATTTTTAATTCTTTTATTAAACACAATATATTGTATTAAATAGAATTTAATACAAAGTTATTTCTATTCTTAAATCATCATTATTTGCATAATTTCGCGCCGTAAAACAATTATCTAATCCCAAATAAAAAACCAATTCCGTCAACATTAGGTCAACATTAATATAAAAAAAGCAAATCAAGCATACAAAAGAGACACAATTTAAACGAAAAACATTGGCAAATCCTTCCGTATCTCGTCTCGGATCTGGAAACGTTGAAATTCCCTCTGCAAAGCCCTCATGTCCAGGTTATCATTTTCGATGTATACCTTAGCCCGGGCCGCTGCCAAATGGGCGTAATAAGTTGGGGCGGGATAACTAACCGATCGGTTGCACCTGCTGAACATGTGGCACAGATAATAAGCGAGCTGCTCGACGGCGTCGTTGTCCAGGAGGTTGTCGTCCCACAGGGTACAGTACTTGGTCGGTTTCGCGACCCCCTGGATGCTGGCGTGCGACACCAGGTAAAAGTCTTGCATGAAGGGGTGAGTTATGTCGGTATCCACGCAAGTACCGGCGGGCACGTTGCAATTCTTGTCCTCCGAGTCGCGAGGGTTGGTCGGAAACAATCTGGTGTGATGCCGCTTTTGAACTACCACGAACGTTATCTTCGGTTCGTAACCGTCCTGCTGGAGCGTTTTACAAGCGGCCCGGATGGCCCTTATCTCCGAATCCCTTACTTGCTGGAACTGACCCTCCGATACGCCGTCCCTGAAGAAAACGATCGCTTCCGGTTTGCACTTGGTCTGCGCGTAGAAGAATTTGAGTTGCTCGACGACGATCGCCTGCAAATCTTCGATTATCTCCAATCTGGGCGACTGCAGTCGCCAACAAATGTTATATTGGAACGCTTTCGGGTCGTGCGAGGCCGTAACGGCCGCAACGCTCGGTATGTTTTGAGCGTCCGGACCCGGATGCGTAACGTCCGCTCCCATAATCATCACCGGTCTTTTCATGATCGGCGGCCGATCGGCAATCGTGTGATTTATACCGTTCAATTTGCTGTTGACTTTCAGTAGGATGTTTAGGGCGGTTTGGGCGTTCATTTTGCGGGCGACCGTTGTCGATTTGACGCACTGCGTCAGACAACCGACGTTTATTTCGGCCGCCCGTTTCACGTACGAGTATTGCGGTCCGCTGTCGGGCACCACGACGAATATCACGTCCAGTTGTTTCTCTTTTTGTACGTTAAAGTATTGGGATATCGTTTGAAAATCTTGTCTCGGGCCGATGGTCGTGAACGGTTCTTGGGCGGGCGTTTCGAATATCATCCCGACATCTCTGGCTGCCCTGTGGAACATATCGGCCATTCTCTTCAAATCGTCGGGCCTGGGACCGTACCGGCTGACGACGCTCGCGATCGTCCACTTCTTAATAACCGCCCCCTTCAGGAACACGTCGTTCCTCCACACCCCCTTAGAGACGCGCACGTTCGAGCGATTGTACTGGATCTGGGGCGGTTGCAAAACTCTCGCGTCCAACTTCTGAAACTCGTTACCGACGGAAAATCCAAACTCCCTGACGCACGGGCTCTGATTATAACTGGCCTGTTGCACTCCCGTCATGATTTTCTGTTTCCGGACGTCGGTAGAGGTCGCCGCTTGCCGAATCATATTACTGGTTTGAATATCGGTCATTTTCCGATTCAAAGCTTGTCCTTCCACTATGGTACAAAGTTCCAAGGGCAGTAAGATCTTGTCGGTTCGTTGCACGCTGCCCACCCAAAGGGTAGGCAAATCGGGGTACCGTAATGCGTAGTTTTTCACCGATCTGAAGTATTCCGCGACGGTCATGTTGCGATTTTCGTGCTGGAACCGTGCTTGGCTGGCCGGTTGACCTAAACCGTTGACGCGGTAACCACGTCTGGTACCCGGGTGACCGGGTATTTCGTAATTCACCCGTAGAGTTTTGATAAAGTGGCTGAGAATCTCCTGTTGTTGCGGATTGAGGTATTGGAGGTCGTTACGACGAAATTCTTGTCTAGGACGACTCAACAGATCGACTAAGGTGTCGATAACGTTTTGGGACTTTGGGAACGCTTTGTGGGCGACGTCTACGTTGAGCAGGGGCGTCCAGCCCCTGATCGCGGACTGGTAGAAGCCGTGGTACATTTCCATACCTTGTCCCAAGTCGATGATCCTCCCTTCGGGCTTGACGAAAAACGACCTGCCTACCGGCAAACAAGTAGTAACCGGTGCACTTCTCAATATAATATCAACCGCTTGTAGAGCCAACTGCGGCGTCGCCGGCGAACTGTGCAAATTCCTCAACGGCGTCAAATCGATCGTATTCGCCCACTTAACCTCGATTTTGAACCGTTTCTCGCGTCCCTCCTCGTCGACGATCGTCACCTCTTCCTCCAAAACGTCGTAAAGGTTCCCACTTAAAAGGAAGGACGTAAACATGTTCTTGTTACCGTCGTAAGCCGGATATTTGTTCGGGAATCTCGTCTGCCGGAACTTTTCGACGACCGCCCTCAAGAATTTCTTGGGCCTGTCCGGCACTATGTTGACGTCGTAATGGTAGACCTCTTTCAAAGTGCCCAAGGACAACTTAAGATGGTTCGATTCGACTTTGATGCGTCTACCTTGAGTGCCCGGTTTGATCTGGACTTTCGACACTAATCCGGAATCGTCGGCGAGCGTTTTCGGCTTCGACACTTGCATTTCACTGACTCTTTTGGTTAAATCTTCCGTAGACGGAGAAGATTTCGTTTGGGGCGCCGGAACTTTAGCCTGAGGCTGTGGCTGAGGCTGAGACGAGGAAACTTGAGGCTGAGGCTGAGAACCGGGCTGGGCCCACGTTTGGTGCCCTCCACGGCCTCGACCTCCACCTCTTTCCTGACCACCCATGCCTCCTCTTTGA

Protein RF -2 : -3056 -> -366 (896AA)

Comparison with *Tribolium* Argonaute 2b (904AA)

Query 39 QAKVPAPQTKSSPSTEDLTKRVSEMQVSKPKTLADDSGLVSKVQIKPGTQGRRIKVESNH 98

++ VP P+ S P E P L + + PGT+GRRI++ESNH

Sbjct 59 KSPVPHPEPSSPPRQE-----------PAPPLSGGGDCLSGALVVTPGTKGRRIQIESNH 107

Query 99 LKLSLGTLKEVYHYDVNIVPDRPKKFLRAVVEKFRQTRFPNKYPAYDGNKNMFTSFLLSG 158

L L+LG L E YHYDV I PD PK LR V+ F + +P +PA+DG KN+++ L

Sbjct 108 LSLNLGKLTEAYHYDVAITPDTPKCLLRDVMNLFGRKHYPQNHPAFDGRKNLYSPKKLP- 166

Query 159 NLYDVLEEEVTIVDEEGREKRFKIEVKWANTIDLTPLRNLHSSPATPQLALQAVDIILRS 218

+ + + V+ E R+K FK+EVK A T+DLTPL ++ + +PQ ALQ +DI+LR+

Sbjct 167 -FPNDTKSDTIEVEGENRKKEFKVEVKLARTVDLTPLHDIMRTTQSPQDALQCLDIVLRN 225

Query 219 APVTTCLPVGRSFFVKP-EGRIIDLGQGMEMYHGFYQSAIRGWTPLLNVDVAHKAFPKSQ 277

AP C+ GR FF P +G+II LG GME+Y+GFYQSAIRGW LLNVDVAHKAFPK+

Sbjct 226 APSNACIIAGRCFFTPPRDGQIIPLGDGMELYYGFYQSAIRGWKALLNVDVAHKAFPKAS 285

Query 278 NVIDTLVDLLSRPRQEFRRNDL-QYLNPQQQEILSHFIKTLRVNYEIPGHPGTRRGYRVN 336

NV+D + ++ S R R +L Q L Q FIK L+V YEIP ++R +RVN

Sbjct 286 NVLDIVCEIGSDFRTTMTRANLSQPLREFVQRDFEKFIKQLKVKYEIPNQSSSKRIHRVN 345

Query 337 GLGQPASQARFQHENRNMTVAE-YFRSVKNYALRYPDLPTLWVGSVQRTDKILLPLELCT 395

GLG+P SQA+F+ ++ MT E Y++ VK L+YP LPTLWVGS +R KILLPLE CT

Sbjct 346 GLGEPPSQAKFKLDDGRMTTVERYYQEVKRCKLQYPHLPTLWVGSRERESKILLPLEFCT 405

Query 396 IVEGQALNRKMTDIQTSNMIRQAATSTDVRKQKIMTGVQQASYNQSPCVREFGFSVGNEF 455

+V GQA+NRKM + QTS MIR+AATSTDVRK KIM ++ A+YN PC+REFGFSV N F

Sbjct 406 VVGGQAINRKMNENQTSAMIRKAATSTDVRKDKIMQTLRTANYNNDPCIREFGFSVSNNF 465

Query 456 QKLDARVLQPPQIQY-NRSNVRVSKGVWRNDV--FLKGAVIKKWTIASVVSRYGPRPDDL 512

+KLDARVL PP + Y + + ++ SKGVWR D FL GA I KWTIAS +RY R D

Sbjct 466 EKLDARVLNPPSLLYADNAQIKPSKGVWRADRNRFLVGATINKWTIASG-TRYPSR--DA 522

Query 513 KRMADMFHRAARDVGMIFETPAQEPFTTIGPRQDFQTISQYFNVQKEKQLDVIFVVVPDS 572

++ADM R A GM + A P T IG RQ + YF + ++ D+I VVVP+S

Sbjct 523 DKLADMIFRMASSNGMQITSKAT-PSTHIGGRQGLRDFIDYF--KGKQDYDLIIVVVPNS 579

Query 573 GPQYSYVKRAAEINVGCLTQCVKSTTVARKMNAQTALNILLKVNSKLNGINHTIA--DRP 630

GPQYS+VK+AAE+NVGCLTQC+K T+ R +N QT NILLK+NSK+NG NH ++ RP

Sbjct 580 GPQYSFVKQAAELNVGCLTQCIKERTIGR-LNPQTVGNILLKINSKMNGTNHRLSPNSRP 638

Query 631 PIMKRPVMIMGADVTHPGPDAQNIPSVAAVTASHDPKAFQYNICWRLQSPRLEIIEDLQA 690

IMKRP MIMGADVTHP PDA++IPSVAAVTASHDP AFQYNICWRLQ P++EIIEDL

Sbjct 639 LIMKRPCMIMGADVTHPSPDARDIPSVAAVTASHDPNAFQYNICWRLQPPKVEIIEDLCN 698

Query 691 IVVEQLKFFYAQTKCKPEAIVFFRDGVSEGQFQQVRDSEIRAIRAACKTLQQDGYEPKIT 750

I VEQLKFFY +T KPE+IVFFRDGVSEGQF+QV+ +EI AI+ ACK LQ+D YEPKIT

Sbjct 699 ITVEQLKFFYQKTGFKPESIVFFRDGVSEGQFKQVQRAEIAAIQKACKMLQKDDYEPKIT 758

Query 751 FVVVQKRHHTRLFPTNPRDSEDKNCNVPAGTCVDTDITHPFMQDFYLVSHASIQGVAKPT 810

F+VVQKRHHTRLFPTNPRDSEDKN NVPAGTCVDT IT+P MQDFYLVSHASIQGVAKPT

Sbjct 759 FLVVQKRHHTRLFPTNPRDSEDKNNNVPAGTCVDTHITNPRMQDFYLVSHASIQGVAKPT 818

Query 811 KYCTLWDDNLLDNDAVEQLAYYLCHMFSRCNRSVSYPAPTYYAHLAAARAKVYIENDNLD 870

KYCTLWDDN ++ND +E+L Y+LCHMF+RCNRSVSYPAPTYYAHLAAARAKVYIEND LD

Sbjct 819 KYCTLWDDNNMNNDDIEELTYHLCHMFTRCNRSVSYPAPTYYAHLAAARAKVYIENDKLD 878

Query 871 MRALQREFQRFQIRDEIRKDLPMFFV 896

M L+R ++ QI+++I K PMFFV

Sbjct 879 MSQLKRHQEKCQIQEKIVKGKPMFFV 904

Graphical representation


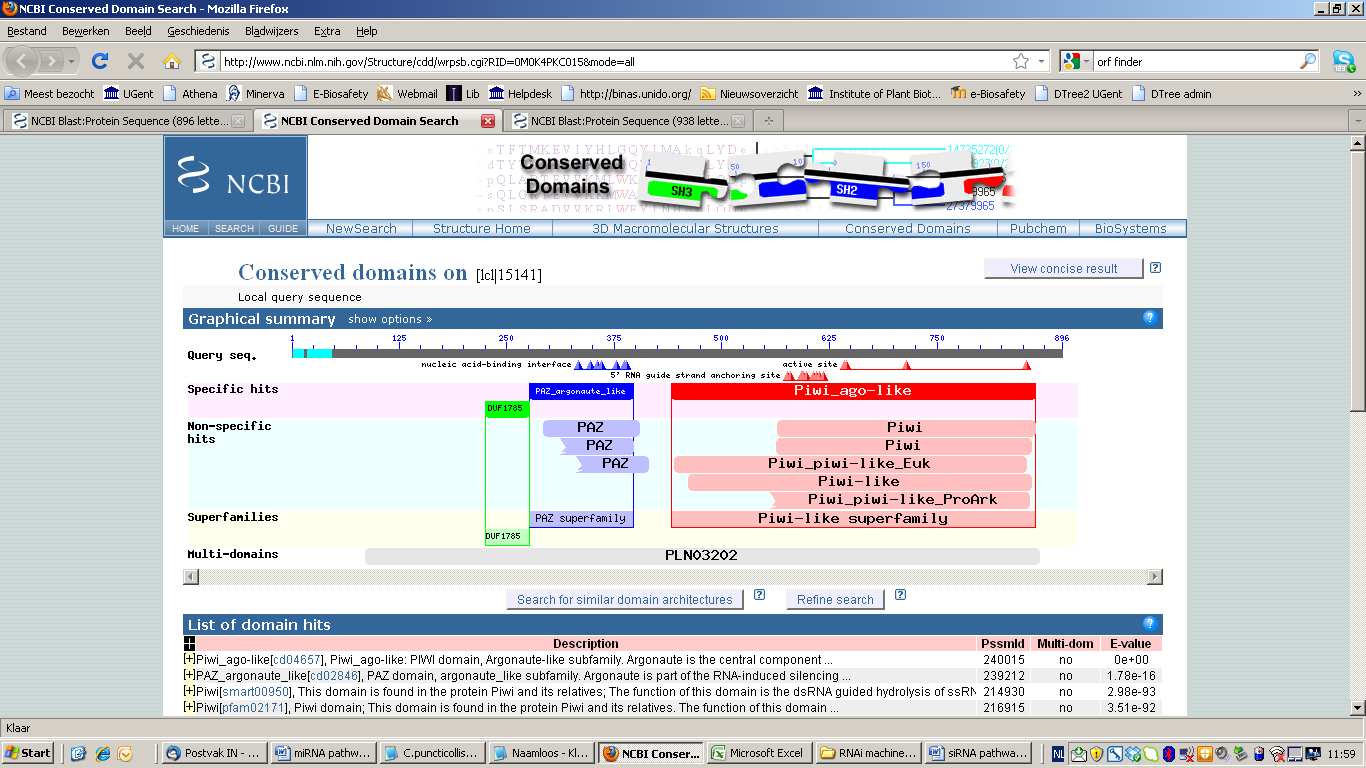


**AGO-3**

>Cp.comp32215_c0_seq1 len=3307

TTTTTTTTTTTCCGTATAAAAAAATATTTATTTAATCTAATAATTATGTCAATGCCACAGAAATTAATAATCGCACAAACATTTCCAACAAAACAGTCATCGATTCAACGTAATATAAAACATGGACATCTTTATTAACAGCCAAAGTGTGCAAGTAATGGTCGAATAAAGAAACGGAGTCGGTAGAAACGTAAACAAAGAAATCAATACTGCTGAGATCCTACTCAAAACTTAAAAACTACATAATCAGTGAAAGTGAAAATATAACCCATTGTTTTCTTAAAAAATAAAGGTTTGTGTTCGATTCACGCTTACAAGAACCATAATTTATCTGCCAGTTCGGCGCTTGGCTTCGTCTTAACGTGCTGGCCAACCATGGCGGCCAACTTATGCGCGTACTGGCAGGGGGCGGGCACCCTTATAGTACCAGGCCAGTTGTAATACAAATGACACAATTTGTAACATAGTCTTTGCACATGATCCGGTTTGAGCTTGCACGTATCGTGCAGCACTATATAATGGGTAGGATTGACGGTTCCTTGTCTGACGCTTTGGGGGATCATGAAGAAATCGTACAGGTATTTTCTCGTGACAGTATGGTCCAAAATTGTTCCCGACGGCGGATTTTCTACTTGGCCATTTCTGCCAAAACCAAACACCCTTGTATTGATCCGTTTCTGAACTACCACGAAACAGATTGTAGTCTGTAGTTTGAACTCTCTAATGACACTCTCGAACTGTTCCACTTCGTACCTCCGGCAATGCTCTAATTGACCGTCGCCCACCCCATCTCTAATTATAACCACTTTGGCGGGGAATTTTCCGGTACTATCTTTGTACTGCTCAAGCGATTTCGCAAACGCCATCTTAAAAAAATCGCTCAGCTCCTTGGTTTGGAACAGAGCGCTAGAGTACCACCTCGAAATACTGTCGTTAAGACTGGTCACGAAACCGCACACCGAATTGGGCGGCGAGCCGTGATAAACGTCGATGCCGCATATCATCCAACCGGTAAATGGAAACCGTAACGTCCACAGACTGCCTCCGAGCTTGCAATTGATTTGAAGTGCAATTTTCAATATTATCGAACGCACTTTGTCCGGCTTCGATAATGTCCGGGAGTTGATCACTTGGGACGCAACTGGCAATTGAGCCGAGCACAGTTTTTTTATCACGGCGTACCGGTCGCTGCGCATCGACGGACAAATAAACACGGCAACCTGAATGGATCTGTCGATTTTGTCCTTCACAAACGTGACATAGGTTTGAGTCTGATCATTTGGAAGTCTTTCCATCTGCGGTTTACTAATGGCAAAGCCGAGGGTCGTTGCCAACCTAAGCATATGTTGCACGAATTCGTTGGCATACTTTGTGTCTTGCTGTGTGTAAAAAACTATCCACCTATGAAGATCGACAGGTGCCACAACCGCATTATTGCCCAATTCTCTGTTCCAGTCGGCAGAAGGCCCTGTGGTTATTTCTATAGAATTTCCGAATCGTATTTTCTCATGTTCCAATTGTCGTCCTTGCAAATCGATATTGCCTTCAGCCACAGAAAGGCCCCACTGGGACAGCACCTGCTGCGCCTTTTCGCTACTCCTTACGTTATCAAGGTACACCCGCAGCGCCTGCATTCTCTGGTGGGGGGTTACTCGGGTATATTGGGCCACATCTTTCATAACCCTAAAATCGTTCCTCATTGTATCAGTCAGACCTGTCAGAAACGACAGTTCGGGCACGAGGCAGATCATCCTATCTTCCTTTTCCGCTTTGCCCGACACTTTGACGCTCCTCCGGTGCAAAAGCAGCGGCTGGTTAACGTCGTTTATGTTTATGTTGTACTGCTGCTTGTAATACTCAACAAATGTGATTGTGTTTCCGTCCCGCGTGGGGAACGTATCACTCGGCGTCATATCCCACAGAATATCGTCGATAATGTAAGTTTTGTTGTTGTACCGAGTAAAAACGCAAGACCCAATGATATTTTTCATGGCGTTTTCCTTAAAAGTTCTTGGATCAGAGGCGCATCGAATCTCGGTCAGCAATTCGTAAGCATTTTGAGTGCGCAACACTCGATGCTGGGTGTCCAAACACAACATCAGACCACCTTCGAGTTCGTCAACCGTTACCGCAAAACCCGGGTATACCTCCAACTTGTGCTGCGGAATTAAATGTTTATGAGCAGGATTGAAGTAATTACGTCCCATTTGAGTATACAGCAATATGTGCATTATTCTCTTAAACAAAATGTTGTACAAATGCAGGCACTCTCTATCACCAAACCTCTTCTGGCGTTTGTAAATAATTGTAACGGAAACTTCCTGATCACCTCCCGGTAAGGTGCCCTTATAAACTTTCTGCTCCTCTTTAATCTTTTGCGGGAGGTATAAGCAATGTCCTCCATCGAAAACTTTAACCGATTCCATATCTCGCATCATCTGGTTGACCATTTTGATCCGTTCATTTTTAGCGTCCAACTCTGGATTGAATCTCACTTCGTACTCGAACACGCCTCTGCCTTTTTCTACGTCAAGGCGGATATAATTGGCCGAAAGCTTTAGGGGCGATCCCGACTCTCCTTTGTATAGTGCTGGCACCTTTTCCTCTTCAGATATGCTCACTTGTTGTAGTTGTTTTGAAACTTCCAGATCCGGATCGGCTTTTGGCTTTTCGCTGCTCCTTTCTGATGTTGGTTCAGGCTTTGGGGCTGGGGCCGATACCGTACTGGATGACTGCCCAATTAATCCCACCTTTTCGGTCTGCAATCTGTATAAAAGCGCGGCCCGTCCTCTCGGTTTTGGTGGATCCTCGGCTTGGGGAATCTGCGCCTTTAGCTGCTCGAGTTTCGCCTTTAGGGCGGCAGCGCGACCGCGGGGCGCTGGTGCCCCGCCATCTGCCATTGCGTGGTTTTTATATCAAACAGATCAAAAAAATCTCTTTACTTAAACCCGCATTACTTTAAAAAAACCTTGCACGGAAAAAAGTGTGGCAAGAATCGAAGCACATGGATCGGATCATAGAACAATAAAAACATATACACATGTGCATATTTCCGATGGATAAAAGCTGAAAATCTTAGGAAACGCAAGAATAAACTTTCTTTTCCTTAAGTAGGTTACTGTAGGTTACAACAACCATAGATAAAAATTTTAAAAAAAACAGTCTGATAAATGTCGCACATGTGTCGCGGAGATATTCGATACGCCAAAAATTTGAACAAAATTATTCACAGAACCAATATCAATAAATTATAATGCTTGACGAACACAATATATTATTGTTTAAAAAAAAAACGACAAGTGAACCA

Protein: -2900 -> -312 (862AA)

Comparison with *Tribolium* argonaute-3 (853AA)

Query 7 PAPRGRAAALKAKLEQLKAQIPQA------EDPPKPRGRAALLYRLQTEKVGLIGQSSST 60

PAP+GR A L+ + +A+ A + PPK RGRA LL ++Q K G S

Sbjct 5 PAPKGRGALLEMLKKHKEARAGGAGEPVEEQAPPKTRGRAMLLQKIQEAKERKAGGDSGQ 64

Query 61 VSAPAPKPEPTSERSSEKPKADPDLEVSKQLQQVSISEEEKVPALYKGESGSPLKLSANY 120

+S P P P+ R V+K L +V+I+ E Y+GESG+P+K +ANY

Sbjct 65 LSTPGPSTVPSETRRGVS-------GVTKALGEVAITASETCS--YRGESGTPIKATANY 115

Query 121 IRLDVEKGRGVFEYEVRFNPELDAKNERIKMVNQMMRDMESVKVFDGGHCLYLPQKIKEE 180

I L+VEK RGVFEYEVRF P++DAK+ RIK+VNQ + ++ + KV+DG CLYLP

Sbjct 116 ILLNVEKDRGVFEYEVRFQPDIDAKSNRIKLVNQALGELSTTKVYDGDVCLYLPCLAFSP 175

Query 181 QKVYKGTLPGGDQEVSVTIIYKRQKRFGDRECLHLYNILFKRIMHILLYTQMGRNYFNPA 240

++ ++ +P + V+ T+IYKR+++ ECLHLYN+LFKRIMHILLY +MGRNYF+P

Sbjct 176 RQEFESVIPNTETPVTTTLIYKRKRKLS--ECLHLYNVLFKRIMHILLYQRMGRNYFSPD 233

Query 241 HKHLIPQHKLEVYPGFAVTVDELEGGLMLCLDTQHRVLRTQNAYELLTEIRCASDPRTFK 300

HK+L+PQHKLEV PGF V VDE+EGGLM+CLDTQHRV+R+Q YEL EIR A++PR F+

Sbjct 234 HKYLVPQHKLEVLPGFCVHVDEMEGGLMVCLDTQHRVIRSQTVYELFHEIR-ATNPRNFR 292

Query 301 ENAMKNIIGSCVFTRYNNKTYIIDDILWDMTPSDTFPTRDGNTITFVEYYKQQYNININD 360

E KN+IG+CV T+YNN+TYIIDDI W+M P DTF R F++YY++ YNI I D

Sbjct 293 EEVTKNVIGACVLTKYNNRTYIIDDIAWNMNPKDTFEDRSKGPSCFIDYYREHYNIRIED 352

Query 361 VNQPLLLHRRSVKVSGKAEKEDRMICLVPELSFLTGLTDTMRNDFRVMKDVAQYTRVTPH 420

V+QPLL+ R+ VK S + E RMICL+PEL +LTGLTD MRNDF+VMKDVA +TR+TP+

Sbjct 353 VDQPLLITRQ-VKQSPDGKIE-RMICLIPELCYLTGLTDAMRNDFKVMKDVAAFTRITPN 410

Query 421 QRMQALRVYLDNVRSSEKAQQVLSQWGLSVAEGNIDLQGRQLEHEKIRFGNSIEITTGPS 480

QRM ALR YLD VR SEKA+QVLS WGLS+A+ +D++ R L E I FG GP

Sbjct 411 QRMLALRTYLDRVRQSEKAKQVLSGWGLSLADDTVDVKARVLPQEAIYFG-------GPD 463

Query 481 A---------DWNRELGNNAVVAPVDLHRWIVFYTQQDTKYANEFVQHMLRLATTLGFAI 531

A DWN+ + +N + PV++ W ++YT++D KYA F Q ++RL +G I

Sbjct 464 AEAHKYTGGTDWNKAISDNKLTGPVNITNWQLYYTRRDQKYAANFAQTIVRLGKGMGCVI 523

Query 532 SKPQMERLPNDQTQTYVTFVKDKIDRSIQVAVFICPSMRSDRYAVIKKLCSAQLPVASQV 591

P+ L +D+T+TY+T ++D + + QVAVFICP++R+DRY++IKK+C +PVASQV

Sbjct 524 QDPRHIVLDDDRTETYMTAIRDNVANT-QVAVFICPTLRADRYSIIKKMCCVNIPVASQV 582

Query 592 INSRTLSKPDKVRSIILKIALQINCKLGGSLWTLRFPFTGWMICGIDVYHGSPPNSVCGF 651

I S+TLS P KVR+II KIA+QI CKLGG+LW+++ P +GWM+CGIDVYHG+ SVCGF

Sbjct 583 ILSKTLSNPQKVRTIIHKIAMQITCKLGGTLWSVKIPVSGWMVCGIDVYHGANNQSVCGF 642

Query 652 VTSLNDSISRWYSSALFQTKELSDFFKMAFAKSLEQYKDSTGKFPAKVVIIRDGVGDGQL 711

V S+N S+++++S A+FQ E+ D+FKM F + L+ KD G FP+KV++ RDGVGDGQL

Sbjct 643 VASINGSMTKYFSKAMFQDGEIGDYFKMPFRQMLQAAKDREGAFPSKVIVFRDGVGDGQL 702

Query 712 EHCRRYEVEQFESVIREFKLQTTICFVVVQKRINTRVFGFGRNGQVENPPSGTILDHTVT 771

EHCR+YE+ Q + VI+E ++TTI FVVVQKRINTR+F ENPPSGT++D+ VT

Sbjct 703 EHCRKYEITQLQEVIKELNIETTITFVVVQKRINTRIFRTVNETNFENPPSGTVVDNMVT 762

Query 772 RKYLYDFFMIPQSVRQGTVNPTHYIVLHDTCKLKPDHVQRLCYKLCHLYYNWPGTIRVPA 831

R+ YDFF++PQSVRQGTVNPTHY+VL D +KPDH+QRL YKLCHLYYNW GTIRVPA

Sbjct 763 RRQFYDFFLVPQSVRQGTVNPTHYVVLVDEGNIKPDHLQRLAYKLCHLYYNWSGTIRVPA 822

Query 832 PCQYAHKLAAMVGQHVKTKPSAELADKLWFL 862

PC YAHKLAA+VGQ++K PS +L DKL++L

Sbjct 823 PCLYAHKLAAIVGQYIKKTPSTQLDDKLFYL 853

Graphical representation


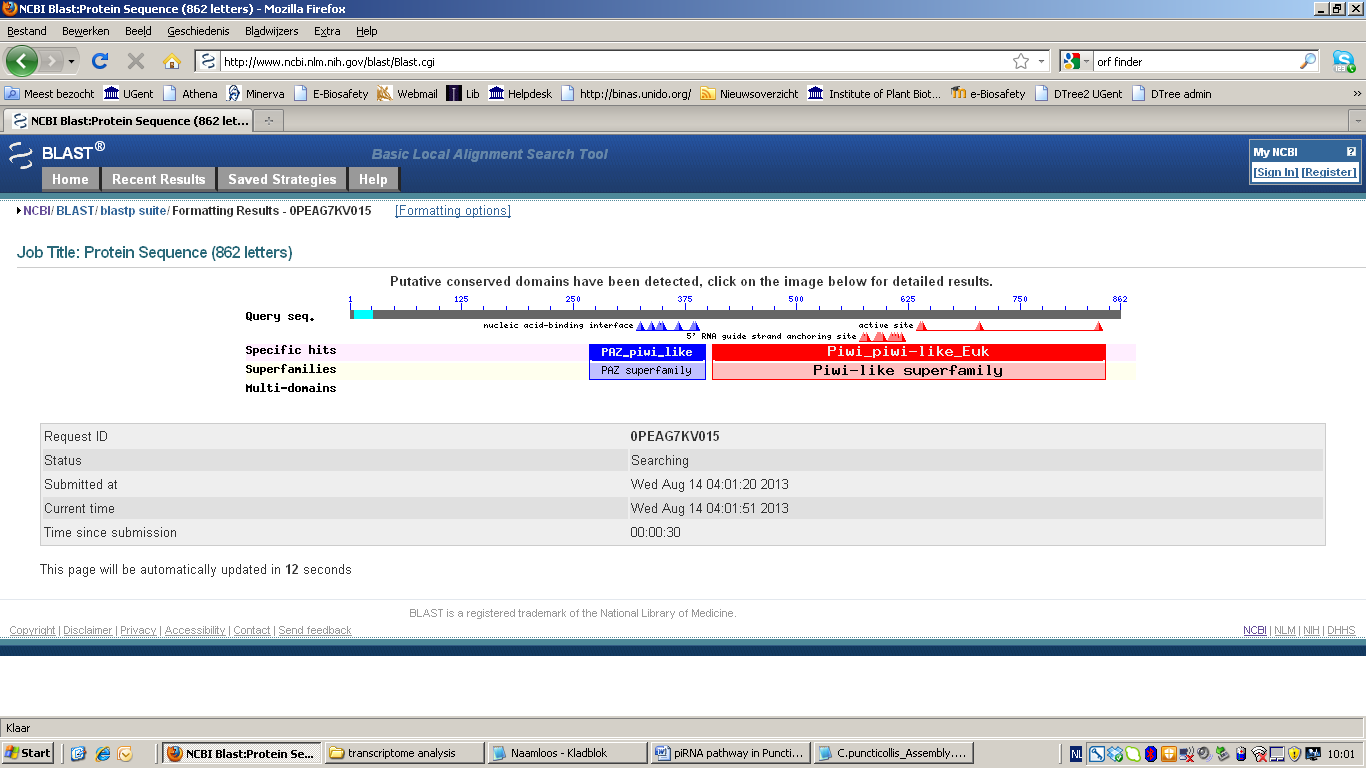


**PIWI**

>Cp.comp31984_c0_seq1 len=3170

TTTTTTTTTTCAAACTTTTATTGAAAATGCTTTAAAAAAATAACATATATCATTTCAACAGCTCAGGTCAACTATACTTAAAAATAGACATGGTCATTTCAGCTAATTTGCCGATATAGCATGAGAAAAAAAAGTCTTATCAATTCAGTAGTCGTGAAACAGAAGAAAAACCTAAATAGTAAATACTTATATCAAAGTCAGATCAAAATAAATGTGGTGAATAAAGACTGACCAACAGTAAACTGAAACAAAACTCTTATAAAGATAAAAAAAAACTTAACATACTGGAAAAATTAAAGATAATATAAGGTGTTTTTCAAGGCCGGATTTGCCGATCTGTGCAAGCTTTGGGCAGTAAGGAATGCAAGTTTATGGGCGTACTGACACGGAGCCGGTACTCGGACGGTACCGGACCAATTGTAATACATATGGCACAATTTAAAAGTAAATCTCTGCAATTTGTTTGCGTCGATACCCAAGGAATCTTCAATCACATTATAACTTGTCGGCGCTACTGTACCTTGACGAACGCACTGAGAAACTATGTAAAAATCATATCTTTCGGGCCAAGTGATGACATCGTCAACGACGGTGCCGGGAGGTGGATTGTAGTCATTCCTTGGGCCTACACCCTGGGCACGGAAAATCTTTGTATTGATTCTCTTTGATACAATTATAAATGCCATTTTAAGCTGTTGAGTTTGGTATATTTCGGCATTCAGTTTCTTCTTGAGCTGTTCGACCTCGTGTTCCTTGACGTATTGGATTTGGCCGTCGCCAACACCGTCTCTGAATATTACTATACGTTCTGGAATAACCTTATTTCTTTCTTGATAATTTTTGCATGCGAGCAACATGAAAGTCGCAATATTGCTACTCAATTCCTGCTCTTGCTCGTGCTCAATTGTCAAACTAAAATATTGACTGCACGCGGTGTCCATGGAGGCGACCATACCGGCAAAACTTTTTCCTCTATTGGCAGTGTCTCGACATACGTCATAACCAACAACCATGGTAGCTTTGGGAAGGGCGGTTCCCCAAGGAGCCCCGCCCATCTTGCAGTTCATTTGAATTGCAACTTTGGTCGCAATCGACATCACACCTTTATTTTGTAGAGTACGTCCGACAACTACCTGGCATGGCATTGCCCGGTCGACGTAACACTTCTTTTTGACGGCATTGTAACGGTCCGCCGAATTGTTCGGTAACACGACCAGTACCATTCTAGGTTGTAAACTGGTAATGACGTGTTCGAGGATGTCCAAATATGACTGGGGACGGTCATCTTGCATTTCCAAGATTTTCGGGTTACCGAGATTAAAGTGCATACCTCTGCCCGCCTTCACTAGACAATTTATAAATTCGTTTGTGCCTTGTCTGAACCGGCCCGGACAAATGACCGCCAATTTATCCGCTTGGGCGATCGTGTACATCGGTGCCGACCGTAACTGTTTGGTGAAATCCGCTTCGGGACCGCCCGTGATCATAGCGCCATTTCTTAACAACAAGGATTCCTCGGGCAATACTCGCCCTTCGAGCTCGATGATATCGCGGGCGACGTCCAAGTCCCATCGTCTTATTTCGTTCATCGCTTCGGGACAATTTTGTAGTCTTTGGCTGAACTCCCTCAGTTTTTGCATACGATTTTGCGGATTTACGCGGGTATATTCGCCCAACGCTTTCATCAAATGGAAATTCTCTCTTTGCTTATCGGTTAAGCCGGTCATTATACAAGTCGACGGAACGAGTATGATAGTCTCGGGCATACCCAACCTAACTTCTCTCATGCTAGCTCTCGAGATGAGCATAGGCTGATCCATGGTGTTTACGGATACTCTCCATCGATTCTGCAAGAACTGGGCATAGCTAATTTGAGATCCATCTTTCATCTGGAAAGTAGACGCCGGCGTTTGCTTAAAATCGACGTCGTCGATACGATAAGTCTTGTTGTTATAATATGTCAACACGACGCTGCCGATAATTCTCGAACGAAATTCGTTTTGAGGGTTCCTACCTTGTAAACATTCTAGGAACAAATCGTAAACGTTATCCGTACGGACGGTTTTAAAGGCTAAATCGGTGTTCATCATTATATCTTTTTCGTACTGTCTAATCGACGTGAAAAAACCTGGCCACAGCTCTAAATTATGTTCCGGCACAGAAATCTTCAGCTTCGGATCATAATAATTCCTATTCATCAATTGTAACTGCATAGATTCCAAACATTTCCTCATGATTATATTGAAAAGTTGCAAGTAATGATAATCGCCCCACTTCACTTCTCCCACTAAACGAATGGTGACGGTAGTGTGAGTTCCGTCGTCGGTATCGACGACGAATTGAAGCGGCTCTGGCTGAATTCGATTGGGGGTGTAAAGGACGGTTCCGTCGAATAAGTAACCGGACACGTTTTTGTTTTTCATACCCGTTCTTACCAGGTATTTGCGTGTCGATGTACTGTCGAGATCGGGAGAAAAATCGACGCGGTACTGATTTAAACCCCAGTGACCTTTCGGTATGAATCGGAAATAGTTCGCTCTGAGCCTGATGGCGGTCCCGTCAAAGCCTTTTTTAGATTGAATATGGCTGGGCCTAGTGCTGATAATTTCATTGCGGTTCGTTCTGCCCCTTACGCCTCCATTGCCGCCGCCGCGGCGTTCTAATCCCGAATCGCCTGCCTGTGACACTTGTCTCGGTTCGGTCGTAATTGATTTGTCATCGCCCCCACCTTGTCTGGAACCACGTCCCACAGTTTGCGATTGAATAGCTTCGGGTGCCGGAGGTCTGGCGGCCCATTGCTGCTGTTGGGGTGGTTGGGAGGGCTGAGCTCTGCCGGCCCAAGCGCCGGGCGGAGGTCCTTGCTGTTGCGATGGACCTCCGCCCGGTCTCCCGGCCCCCCAAGCTCCTTGGGGTGCCTGTCCTTGTGGGGGACCCGGTCTCTGATGTTGCTGCTGACCCCCAGCTTGTTGCTGTGCGCCACGGGCACGACCTCGAGCTCTCCCTTTGCCCCTTGGTTCCATCCTTCCGCCGATAAAAAATTTCACAAAAGAAAGTTGTTTTTCTACACACTTTCTGAGGCCCGCATGCAACTGTCCCAAATGCGAAGTAAAAATGGCCAGAGAGCTACGAACTAACTATAGGTCTCTGAAATGGCTTGCAATTTAGTGAAG

Protein: -3020 -> -294 (908AA)

Comparison with *Tribolium* piwi (889AA)

Query 54 QGPPPGAWAGRAQP--SQPPQQQQWAARPPAPEAIQSQTVGRGSRQ----GGGDDKSITT 107

+G P AW AQP S P+ Q +A P+ + Q GRG RQ G + I

Sbjct 42 RGLPQSAWVRPAQPAGSAWPRPQMGSA----PQQQKPQVAGRGERQEFDQGAVPRRVIAG 97

Query 108 EPRQVSQAGDSGLERRGGGNGGVRGRTNRNEIISTRPSHIQSKKGFDGTAIRLRANYFRF 167

E Q +Q G G R GGG VRGR R EI+ TRP +++SKKG GT I L ANY

Sbjct 98 EGDQGNQEGSQGAAR-GGGASSVRGRVVRKEILYTRPQNLKSKKGTIGTPINLIANYLPL 156

Query 168 IPKGHWGLNQYRVDFSPDLDSTSTRKYLVRTGMKNKNVSGYLFDGTVLYTPNRIQPEPLQ 227

I +G W L QYRVD +PD+D+T+ RK LVR +K+ GYLFDGTVLYT RI + +

Sbjct 157 IKQGKWCLYQYRVDMAPDVDNTNKRKELVRVAVKDLLKGGYLFDGTVLYTTQRINNDSVD 216

Query 228 FVVDTDDGTHTTVTIRLVGEVKWGDYHYLQLFNIIMRKCLESMQLQLMNRNYYDPKLKIS 287

VD + G + +TIRLVG++ WGD HY+QLFNII+RKCL+ M LQ + RNY+ P KI

Sbjct 217 LFVD-NSGENVRITIRLVGDLAWGDMHYIQLFNIIIRKCLKLMGLQQVGRNYFMPDNKIV 275

Query 288 VPEHNLELWPGFFTSIRQYEKDIMMNTDLAFKTVRTDNVYDLFLECLQGRNPQNEFRSRI 347

+ EH ++LWPG+FTS+RQ+EKDI++N DL FK +RTD VYD LEC QG N + EF+S+I

Sbjct 276 ISEHKIQLWPGYFTSMRQHEKDILLNVDLQFKFMRTDTVYDNLLEC-QGANARKEFQSKI 334

Query 348 IGSVVLTYYNNKTYRIDDVDFKQTPASTFQMKDGSQISYAQFLQNRWRVSVNTMDQPMLI 407

IGSVVLT+YNNKTY+IDDVDF TPA TF++KDGS+ ++ + + ++ V + DQPMLI

Sbjct 335 IGSVVLTHYNNKTYKIDDVDFNSTPAHTFKLKDGSETTFKDYFKKKYNVDIRVKDQPMLI 394

Query 408 SRASMREVRLGMPETIILVPSTCIMTGLTDKQRENFHLMKALGEYTRVNPQNRMQKLREF 467

SR+ RE+R+G+PET+ LVP C+MTGLTD+QRENF+LMK L +TR+ + R++KL EF

Sbjct 395 SRSKPREIRVGVPETVYLVPELCLMTGLTDRQRENFNLMKMLATHTRIGVEGRIKKLMEF 454

Query 468 SQRLQNCPEAMNEIRRWDLDVARDIIELEGRVLPEESLLLRNGAMITGGPEADFTKQLRS 527

SQ+L N P+ +NEIRRW LDV ++ +GRVLP+E+++ N A + GP+AD+TK+LRS

Sbjct 455 SQKLHNKPDVVNEIRRWGLDVGNSLVRFQGRVLPQETVVGGNDAKYSAGPQADWTKELRS 514

Query 528 APMYTIAQADKLAVICPGRFRQGTNEFINCLVKAGRGMHFNLGNPKILEMQDDRPQSYLD 587

PM + + ++LAV+C R + T +FI L K GM ++LGNPKI ++QDDR SY++

Sbjct 515 RPMLYMPKMERLAVVCSHRNKSATQDFIQLLAKTAGGMRWSLGNPKIFDIQDDRSGSYIE 574

Query 588 ILEHVITSLQPRMVLVVLPNNSADRYNAVKKKCYVDRAMPCQVVVGRTLQNKGVMSIATK 647

+E +I QP M+LV+LPNNS +RY+A+KKKCYVDR +P Q+ V R L +KGVMSIATK

Sbjct 575 QIEKIINMNQPTMILVILPNNSTERYSAIKKKCYVDRGIPTQMFVARNLTSKGVMSIATK 634

Query 648 VAIQMNCKMGGAPWGTALP-KATMVVGYDVCRDTANRGKSFAGMVASMDTACSQYFSLTI 706

VAIQMNCK+GGAPW +P MVVGYDVCRDT N+ KSFAG+V S+D S+++++

Sbjct 635 VAIQMNCKIGGAPWCVPIPLSGLMVVGYDVCRDTVNKKKSFAGIVGSLDKNISRFYNICC 694

Query 707 EHEQEQELSSNIATFMLLACKNYQERNKVIPERIVIFRDGVGDGQIQYVKEHEVEQLKKK 766

EH+ E+ELS N A ++L CK Y+E+N PERI+I+RDGVG+GQ+ +V EHEV +K+K

Sbjct 695 EHKMEEELSDNFAAAVVLLCKQYKEQNGHYPERILIYRDGVGEGQLPFVVEHEVANIKRK 754

Query 767 LNAEIYQTQQLKMAFIIVSKRINTKIFRAQGVGPRNDYNPPPGTVVDDVITWPERYDFYI 826

L EIY ++KMAF++VSKRINT+IF + NPPPGTVVDDVIT PERYDFYI

Sbjct 755 LQEEIYINGEVKMAFVVVSKRINTRIFTEKD-------NPPPGTVVDDVITLPERYDFYI 807

Query 827 VSQCVRQGTVAPTSYNVIEDSLGIDANKLQRFTFKLCHMYYNWSGTVRVPAPCQYAHKLA 886

VSQCVRQGTVAPTSYNVIEDS+G+ KLQ T+KL HMYYNWSGTVRVPAPCQYAHKLA

Sbjct 808 VSQCVRQGTVAPTSYNVIEDSMGLPPEKLQYLTYKLTHMYYNWSGTVRVPAPCQYAHKLA 867

Query 887 FLTAQSLHRSANPALKNTLYYL 908

F+ +Q +HR A+ L N LYYL

Sbjct 868 FMVSQYIHRPAHHDLDNVLYYL 889

Graphical representation


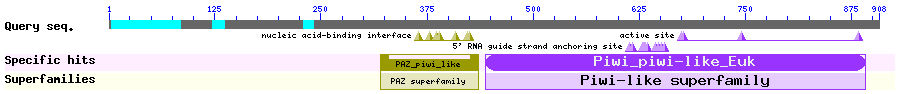


**Aubergine**

>Cp.comp31984_c0_seq1 len=3170

TTTTTTTTTTCAAACTTTTATTGAAAATGCTTTAAAAAAATAACATATATCATTTCAACAGCTCAGGTCAACTATACTTAAAAATAGACATGGTCATTTCAGCTAATTTGCCGATATAGCATGAGAAAAAAAAGTCTTATCAATTCAGTAGTCGTGAAACAGAAGAAAAACCTAAATAGTAAATACTTATATCAAAGTCAGATCAAAATAAATGTGGTGAATAAAGACTGACCAACAGTAAACTGAAACAAAACTCTTATAAAGATAAAAAAAAACTTAACATACTGGAAAAATTAAAGATAATATAAGGTGTTTTTCAAGGCCGGATTTGCCGATCTGTGCAAGCTTTGGGCAGTAAGGAATGCAAGTTTATGGGCGTACTGACACGGAGCCGGTACTCGGACGGTACCGGACCAATTGTAATACATATGGCACAATTTAAAAGTAAATCTCTGCAATTTGTTTGCGTCGATACCCAAGGAATCTTCAATCACATTATAACTTGTCGGCGCTACTGTACCTTGACGAACGCACTGAGAAACTATGTAAAAATCATATCTTTCGGGCCAAGTGATGACATCGTCAACGACGGTGCCGGGAGGTGGATTGTAGTCATTCCTTGGGCCTACACCCTGGGCACGGAAAATCTTTGTATTGATTCTCTTTGATACAATTATAAATGCCATTTTAAGCTGTTGAGTTTGGTATATTTCGGCATTCAGTTTCTTCTTGAGCTGTTCGACCTCGTGTTCCTTGACGTATTGGATTTGGCCGTCGCCAACACCGTCTCTGAATATTACTATACGTTCTGGAATAACCTTATTTCTTTCTTGATAATTTTTGCATGCGAGCAACATGAAAGTCGCAATATTGCTACTCAATTCCTGCTCTTGCTCGTGCTCAATTGTCAAACTAAAATATTGACTGCACGCGGTGTCCATGGAGGCGACCATACCGGCAAAACTTTTTCCTCTATTGGCAGTGTCTCGACATACGTCATAACCAACAACCATGGTAGCTTTGGGAAGGGCGGTTCCCCAAGGAGCCCCGCCCATCTTGCAGTTCATTTGAATTGCAACTTTGGTCGCAATCGACATCACACCTTTATTTTGTAGAGTACGTCCGACAACTACCTGGCATGGCATTGCCCGGTCGACGTAACACTTCTTTTTGACGGCATTGTAACGGTCCGCCGAATTGTTCGGTAACACGACCAGTACCATTCTAGGTTGTAAACTGGTAATGACGTGTTCGAGGATGTCCAAATATGACTGGGGACGGTCATCTTGCATTTCCAAGATTTTCGGGTTACCGAGATTAAAGTGCATACCTCTGCCCGCCTTCACTAGACAATTTATAAATTCGTTTGTGCCTTGTCTGAACCGGCCCGGACAAATGACCGCCAATTTATCCGCTTGGGCGATCGTGTACATCGGTGCCGACCGTAACTGTTTGGTGAAATCCGCTTCGGGACCGCCCGTGATCATAGCGCCATTTCTTAACAACAAGGATTCCTCGGGCAATACTCGCCCTTCGAGCTCGATGATATCGCGGGCGACGTCCAAGTCCCATCGTCTTATTTCGTTCATCGCTTCGGGACAATTTTGTAGTCTTTGGCTGAACTCCCTCAGTTTTTGCATACGATTTTGCGGATTTACGCGGGTATATTCGCCCAACGCTTTCATCAAATGGAAATTCTCTCTTTGCTTATCGGTTAAGCCGGTCATTATACAAGTCGACGGAACGAGTATGATAGTCTCGGGCATACCCAACCTAACTTCTCTCATGCTAGCTCTCGAGATGAGCATAGGCTGATCCATGGTGTTTACGGATACTCTCCATCGATTCTGCAAGAACTGGGCATAGCTAATTTGAGATCCATCTTTCATCTGGAAAGTAGACGCCGGCGTTTGCTTAAAATCGACGTCGTCGATACGATAAGTCTTGTTGTTATAATATGTCAACACGACGCTGCCGATAATTCTCGAACGAAATTCGTTTTGAGGGTTCCTACCTTGTAAACATTCTAGGAACAAATCGTAAACGTTATCCGTACGGACGGTTTTAAAGGCTAAATCGGTGTTCATCATTATATCTTTTTCGTACTGTCTAATCGACGTGAAAAAACCTGGCCACAGCTCTAAATTATGTTCCGGCACAGAAATCTTCAGCTTCGGATCATAATAATTCCTATTCATCAATTGTAACTGCATAGATTCCAAACATTTCCTCATGATTATATTGAAAAGTTGCAAGTAATGATAATCGCCCCACTTCACTTCTCCCACTAAACGAATGGTGACGGTAGTGTGAGTTCCGTCGTCGGTATCGACGACGAATTGAAGCGGCTCTGGCTGAATTCGATTGGGGGTGTAAAGGACGGTTCCGTCGAATAAGTAACCGGACACGTTTTTGTTTTTCATACCCGTTCTTACCAGGTATTTGCGTGTCGATGTACTGTCGAGATCGGGAGAAAAATCGACGCGGTACTGATTTAAACCCCAGTGACCTTTCGGTATGAATCGGAAATAGTTCGCTCTGAGCCTGATGGCGGTCCCGTCAAAGCCTTTTTTAGATTGAATATGGCTGGGCCTAGTGCTGATAATTTCATTGCGGTTCGTTCTGCCCCTTACGCCTCCATTGCCGCCGCCGCGGCGTTCTAATCCCGAATCGCCTGCCTGTGACACTTGTCTCGGTTCGGTCGTAATTGATTTGTCATCGCCCCCACCTTGTCTGGAACCACGTCCCACAGTTTGCGATTGAATAGCTTCGGGTGCCGGAGGTCTGGCGGCCCATTGCTGCTGTTGGGGTGGTTGGGAGGGCTGAGCTCTGCCGGCCCAAGCGCCGGGCGGAGGTCCTTGCTGTTGCGATGGACCTCCGCCCGGTCTCCCGGCCCCCCAAGCTCCTTGGGGTGCCTGTCCTTGTGGGGGACCCGGTCTCTGATGTTGCTGCTGACCCCCAGCTTGTTGCTGTGCGCCACGGGCACGACCTCGAGCTCTCCCTTTGCCCCTTGGTTCCATCCTTCCGCCGATAAAAAATTTCACAAAAGAAAGTTGTTTTTCTACACACTTTCTGAGGCCCGCATGCAACTGTCCCAAATGCGAAGTAAAAATGGCCAGAGAGCTACGAACTAACTATAGGTCTCTGAAATGGCTTGCAATTTAGTGAAG

Protein: -3020 -> -294 (908AA)

Comparison with *Tribolium* Aubergine (901AA)

Query 64 RAQPSQPPQQQQWAARPPAPE-AIQSQTVGRGSRQGGGDDKSITTEPRQVSQAGDSGLER 122

R Q P+ W A P +Q + GR S + ++ T P ++ GD G +

Sbjct 62 RVQHGHGPK---WGALPSNRSLKLQDEERGRNSTRALCPEEIYITYP-IIAGEGDQGNQE 117

Query 123 ------RGGGNGGVRGRTNRNEIISTRPSHIQSKKGFDGTAIRLRANYFRFIPKGHWGLN 176

RGGG VRGR R EI+ TRP +++SKKG GT I L ANY I +G W L

Sbjct 118 GSQGAARGGGASSVRGRVVRKEILYTRPQNLKSKKGTIGTPINLIANYLPLIKQGKWCLY 177

Query 177 QYRVDFSPDLDSTSTRKYLVRTGMKNKNVSGYLFDGTVLYTPNRIQPEPLQFVVDTDDGT 236

QYRVD +PD+D+T+ RK LVR +K+ GYLFDGTVLYT RI + + VD + G

Sbjct 178 QYRVDMAPDVDNTNKRKELVRVAVKDLLKGGYLFDGTVLYTTQRINNDSVDLFVD-NSGE 236

Query 237 HTTVTIRLVGEVKWGDYHYLQLFNIIMRKCLESMQLQLMNRNYYDPKLKISVPEHNLELW 296

+ +TIRLVG++ WGD HY+QLFNII+RKCL+ M LQ + RNY+ P KI + EH ++LW

Sbjct 237 NVRITIRLVGDLAWGDMHYIQLFNIIIRKCLKLMGLQQVGRNYFMPDNKIVISEHKIQLW 296

Query 297 PGFFTSIRQYEKDIMMNTDLAFKTVRTDNVYDLFLECLQGRNPQNEFRSRIIGSVVLTYY 356

PG+FTS+RQ+EKDI++N DL FK +RTD VYD LEC QG N + EF+S+IIGSVVLT+Y

Sbjct 297 PGYFTSMRQHEKDILLNVDLQFKFMRTDTVYDNLLEC-QGANARKEFQSKIIGSVVLTHY 355

Query 357 NNKTYRIDDVDFKQTPASTFQMKDGSQISYAQFLQNRWRVSVNTMDQPMLISRASMREVR 416

NNKTY+IDDVDF TPA TF++KDGS+ ++ + + ++ V + DQPMLISR+ RE+R

Sbjct 356 NNKTYKIDDVDFNSTPAHTFKLKDGSETTFKDYFKKKYNVDIRVKDQPMLISRSKPREIR 415

Query 417 LGMPETIILVPSTCIMTGLTDKQRENFHLMKALGEYTRVNPQNRMQKLREFSQRLQNCPE 476

+G+PET+ LVP C+MTGLTD+QRENF+LMK L +TR+ + R++KL EFSQ+L N P+

Sbjct 416 VGVPETVYLVPELCLMTGLTDRQRENFNLMKMLATHTRIGVEGRIKKLMEFSQKLHNKPD 475

Query 477 AMNEIRRWDLDVARDIIELEGRVLPEESLLLRNGAMITGGPEADFTKQLRSAPMYTIAQA 536

+NEIRRW LDV ++ +GRVLP+E+++ N A + GP+AD+TK+LRS PM + +

Sbjct 476 VVNEIRRWGLDVGNSLVRFQGRVLPQETVVGGNDAKYSAGPQADWTKELRSRPMLYMPKM 535

Query 537 DKLAVICPGRFRQGTNEFINCLVKAGRGMHFNLGNPKILEMQDDRPQSYLDILEHVITSL 596

++LAV+C R + T +FI L K GM ++LGNPKI ++QDDR SY++ +E +I

Sbjct 536 ERLAVVCSHRNKSATQDFIQLLAKTAGGMRWSLGNPKIFDIQDDRSGSYIEQIEKIINMN 595

Query 597 QPRMVLVVLPNNSADRYNAVKKKCYVDRAMPCQVVVGRTLQNKGVMSIATKVAIQMNCKM 656

QP M+LV+LPNNS +RY+A+KKKCYVDR +P Q+ V R L +KGVMSIATKVAIQMNCK+

Sbjct 596 QPTMILVILPNNSTERYSAIKKKCYVDRGIPTQMFVARNLTSKGVMSIATKVAIQMNCKI 655

Query 657 GGAPWGTALP-KATMVVGYDVCRDTANRGKSFAGMVASMDTACSQYFSLTIEHEQEQELS 715

GGAPW +P MVVGYDVCRDT N+ KSFAG+V S+D S+++++ EH+ E+ELS

Sbjct 656 GGAPWCVPIPLSGLMVVGYDVCRDTVNKKKSFAGIVGSLDKNISRFYNICCEHKMEEELS 715

Query 716 SNIATFMLLACKNYQERNKVIPERIVIFRDGVGDGQIQYVKEHEVEQLKKKLNAEIYQTQ 775

N A ++L CK Y+E+N PERI+I+RDGVG+GQ+ +V EHEV +K+KL EIY

Sbjct 716 DNFAAAVVLLCKQYKEQNGHYPERILIYRDGVGEGQLPFVVEHEVANIKRKLQEEIYING 775

Query 776 QLKMAFIIVSKRINTKIFRAQGVGPRNDYNPPPGTVVDDVITWPERYDFYIVSQCVRQGT 835

++KMAF++VSKRINT+IF + NPPPGTVVDDVIT PERYDFYIVSQCVRQGT

Sbjct 776 EVKMAFVVVSKRINTRIFTEKD-------NPPPGTVVDDVITLPERYDFYIVSQCVRQGT 828

Query 836 VAPTSYNVIEDSLGIDANKLQRFTFKLCHMYYNWSGTVRVPAPCQYAHKLAFLTAQSLHR 895

VAPTSYNVIEDS+G+ KLQ T+KL HMYYNWSGTVRVPAPCQYAHKLAF+ +Q +HR

Sbjct 829 VAPTSYNVIEDSMGLPPEKLQYLTYKLTHMYYNWSGTVRVPAPCQYAHKLAFMVSQYIHR 888

Query 896 SANPALKNTLYYL 908

A+ L N LYYL

Sbjct 889 PAHHDLDNVLYYL 901

Graphical representation


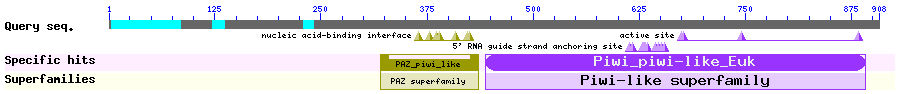


**Zucchini**

>Cp.comp38142_c1_seq5 len=2838

GTATTGTCTATGGTTAAGTTGTGTCTTATGTGCTAGAGTTGCAACATTTGTCAAAGTCAGCACCTGTAGAAAAAGTTCAGTTTTTATTTCGTTTCAAACCCTAAAATGTTTAATGGGAACAAAAATGGTTAAATTGTAGTTAAATTCAATTAAAATCACTTTCATAATTGAAGACCCCGTGACGCAAAGATTAGCAGTATCTCTGGTATTAAATTCGAAACTCAAGGTGTAACTAATTTGGCCCAATAACAACCGGAGCCGAATGAATAAACAATTCAACGAAAACAACTTGTAAATTTTGGTTTTCACTTTATAAATGCAAGATAAAAATTCGAAAATATGTATTTTTTAAACAACTTTCAACAATCATTGTTTAAAAAAAATGATTTCCTGTCTTTACAGCCACAACTTTTAATGTTCATATAAAAAATGAAAACTCATTAACAAATTTATAGCTTTATTTAGCAAACTTGAAGACGATTTAAAGATTTAAAAAAAAACAAAATAAATTTGGTACATCTGAATTACATTCATAAATTTATAAATTATTAATACTCTTGATCCATCGAGATATTAAAAGGTGTAAAAGTAATCTCATTTTAACTTAAGATCTAATCTGGTATAAATATGAAATTTTACAAAATTTACATGAGTTGTGCATCCATTAATACCGTTTTATTTATTAAACCTTCGTTATCCATTTTAACATTTTCCCAACATTCTTCGAAATGATTTTTAAATGCGTTGACTAGGTAAGGATTGGAGCTGAAAACAACGGATTCCGAGTTGTTCATAACACTACTGGCGGTCAAGTTCATGGAACCGGTACACAAATATCCTCCTTTATCACCGTTCGCGTCTTTGATCATAAATTTATGGTGCATTATGGAATCTATAGCCGACGTTGGGGCTACGAAAAATTGATAGTCGACACCTTGTTTCATTAATTCTCTAATATCTTCGCTTGAGCTTTCGCAATGGTGAAAATTACTGATGATCCTAACGTCTACACCTCGTCTTCTAGCTTTGGCAAGTTCGGAATAAATAACTTTTACATTTATCAACATAAAAGCAATTTGGAGTGATTCTTTGGCGGTTTTTATAAAGTAGGTAAATGGTTCGTATAAGTCTTCAACGACATTGATACTTTTAATTTTCAGTTTTCTGTATTCAGGATATCCCGATAAACCTCTGTTGGGATCGTATGTGACGACGCAATTGTGCCTTTTATAAAAAAGGATTCTTTCATTATAATCTTTGCGTAACTTCTGCCAAAGCTGAAAAACCGACTTCCAACCGTAAAACGGTATAACTAGGCAAGTTACGGACGAAATAAACAAGATCTTATTTTTTGATAGATTTTCCCACATATTTTTTATAGGTTAGAGCGATTTTAATGTTAGGTTAGGTCGGCAAGGTCAATGGAAACGTCACTTTCGTATATTGTAAAATAATAAGCGATAAGACGAAAAATATTAAAAACTGGAATTTGGATTGGGGGCCTGAACTACTTCAAAATACACACGCATACAGACATGCGATGACGCAAGATAAAATAATCAACCGAACGTTAGTTTTGTAACCAACAAGCGGCCGCCGCGGAGTCGAAAAATGAGTGAACTGCAGGATAATGGTCGCGAGGCTTTAGTAGGAGTAATAGATGCCAGTACAAGAACTGTAAAATTTTGTGTCTTTATTAGTCAGCATATAAAAGAAATAGCAGAATACGCTATAGATTTGGAACCGATAACTCCACAGGAAGGTTGGTCCGAACAAGATCCTTTAAAGATATTGGCGGCTGTTAAACAATGTATGCAAAAAGTTATAACTTCTTTAGGACCCAAAGCTAGGAATATTGTTACAATTGGTATTACAAATCAGAGAGAAACCACAATATTATGGGATAAGACTACAGGAAAGCCTCTCTATAATGCCATAGTGTGGAATGACATCCGAACAGATTCGACCGTAGACGTAATATTAGCTAAAATTCCGGATAACAACAAGAACCATTTTAAACACATTTGCGGTCTTCCAGTTTCACCATATTTTAGCGCTTTTAAATTAAAGTGGCTTATGCATTACGTACCTGGCGTTAAAAAGGCTATTAGGGAAAAAAAGTGCCTTTTCGGGACAGTTGACACTTGGTTGTTGTGGAATTTGACCGGCGGTATTAACAACGGTAAACACTTTACCGATGTTACCAACGCCTCAAGGACATTTCTTATGAACATCGAGACACTACATTGGGATCCGTTGCTCTTAAATTCTTTTAAAATACCTCACGATATTTTGCCGGAAATTAAGAGTAGTTCAGAAATATACGGAAGGGTTTCGAAAGGGTGGCCTTTGGAAAATGTGGTTATATCAGGCATTTTAGGCAATCAGCAATCGGCCTTAATAGGTCAAAGCTGTTTCAAAGAAGGTCAGGCGAAGAACACTTACAGAAGCGGTTGTTTTTTGCTTTACAATACGGGTACAACGAGGGTGCAATCGTCGCACGGTTTAGTAACTACGGTGGCGTATAAATTCGGAGATGCTCCCACCATTTACGCCTTAGAAGGAAGTGTGGCAGTGGCGGGCGCAGCCATGAATTGGTTACGGGACAACATGGGTTTCGTCAGAAACGTTTCCGAAGATACCGAATCACTGGCGAAGGAAGTGTTCAGTACCGGTGACGTTTACTTCGTACCGGCATTCAAAGGGTTATACGCCCCTTATTGGCGGAAAGACGCAAGAGGGTGTAATAATTTGTGGTTTGACAGCGTTTTCGACGAAACAGCACATTATTAGGGCGGCTTTAGAAGCGGTATGTTTTCAGACGAGGGATATTCTGGA

Protein: -1370 -> -645 (241AA)

Comparison with *Tribolium* hypothetical protein TcasGA2_TC010319 (236AA)

| **Score** | **Expect** |  | **Identities** | **Positives** | **Gaps** |
| --- | --- | --- | --- | --- | --- |
| 166 bits(419) | 9e-47 |  | 86/222(39%) | 136/222(61%) | 5/222(2%) |

Query 19 LVIP-FYGWKSVFQLWQKLRKDYNERILFYKRHNCVVTYDPNRGLSGYPEYRKLKIKSIN 77

L +P Y +K ++ ++ L++ Y + LFY+RHNCVV Y ++G+P K+ I + N

Sbjct 14 LFVPCVYMFKKLYSTYKSLKQQYEDEELFYQRHNCVVMYSKTH-ITGWPPEYKMSISTKN 72

Query 78 VVEDLYEPFTYFIKTAKESLQIAFMLINVKVIYSELAKARRRGVDVRIISNFHHCESSSE 137

+ + +P+ YFI T+K S+ +A M ++K + L A RGV VR+I N+ ++ ++

Sbjct 73 L-DKFLDPYLYFINTSKHSIDLAVMTFSLKPLMEALQSALTRGVKVRLIVNYLSVKNQAK 131

Query 138 DIRELMKQGVDYQFFVAPTSAIDSIMHHKFMIKDANGDKGGYLCTGSMNLTA-SSVMNNS 196

ELMK G+ F++ T+++ +IMH K+ +KD +G K G+L GSMNLT S+++NN

Sbjct 132 QYNELMKSGIKVAFYIEKTTSLSNIMHCKYSVKDYDGSK-GFLFMGSMNLTGMSTILNNY 190

Query 197 ESVVFSSNPYLVNAFKNHFEECWENVKMDNEGLINKTVLMDA 238

E V F+SN YLV F F++ W + DNE NKTVL D+

Sbjct 191 EDVTFTSNIYLVETFHKSFQDSWNMIMEDNENFYNKTVLADS 232

Graphical representation


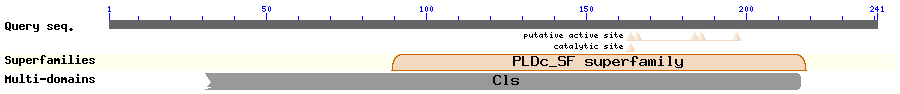

Supplement: S1 Supporting Information — (DOCX) [file pone.0115336.s001.docx]
