# Supplementary material for: Transcriptome Analysis and Systemic RNAi Response in the African Sweetpotato Weevil (Cylas puncticollis, Coleoptera, Brentidae)
Source: PLoS One. 2015 Jan 15;10(1):e0115336. doi: 10.1371/journal.pone.0115336 (PMC4295849; doi:10.1371/journal.pone.0115336)

**Supporting Information S2:** Sequences of *C. puncticollis* RISC-associated auxiliary factors.

**Tudor-SN**

>Cp.comp40322_c1_seq1 len=2395

GGAGACTTTCCTCGAGCCGATCCTACGATATTACATTCACTTTTTTTTTTTTTTAATAAACAATTTCATTTACTTTTTAAAACAGAGCCACTCCCCTATGTAAAAAGGTATACTTGCGTGTAATACATAAATAGAAAATTTATTGCAAAGAAAAAACAAAGAGTAAAGTTAATAATGTACAAAGGTTTTCCTAACAAAAATTTAATTTAAAATTTTAATGAAAAAACTCTCCGACATTTGCTGTTGCAGAACTTAAGACTCCTTAAGCTTTGATTCAGTCTGGAAGTTTGCGAAATAAGCTTCATGAAATAAACGAACACAAAACAAAAAAATGCAATAAAAATTGGTCATTTTATTATTATTTTGTCAACTGTCTTATATCTAAATGTCATAATGGATGAAATAAGAGATGATGGTTCGACTCGACTCGGACATCCCTTCCCGACCCCTGTAAATTAGTACATTAAGGTACGTGCTCTGGCTTTTGTTTGCTTCGTGGCCGGTCGATTGCTAATTGCCGAGCCCGAATTCCTTGGCGTCATCCTCTCTGATGTCGCCGTACTGCCAAATATTCAAGTGCTCTTTCTTCGCTTGATCCTGGGCCTGTTTATAGTCTTCGAGCAGCTTATTCTGCCTCCTGGATTTGATATTGTCCACCAGGAGAAGTCCTTCGGCGATCAAAGCCTGGAAAATGTCCGACGCGCCCGACGTATCTTTATGCAAAGAAGCTGCTGGTGGCGAGCCTTGGTTTCTGTACTCCAAGTTCAAATACAGTTTGCTAACGTTGGTATCTTCCCTCAGATATTTAAGCGCCAATTCTCTGTCGTCTTCGTCTTTCGGCAAAGAAACGTACGGCATGACGTATTCGGTGGCGAAAGGCTTATCGGCAGAATAGGCGGCGGGCAAACTGGCCAATCTTGTAGTGGGCAGCACTTCCCGATTACCGTAATCGATATAATGTACCGTGGCTTTGCCGTTTTGTACCTTTTCGACTTTCACCCTGTACCATTCGTTGTCGAGGGTAAATTGGGCAGCGCACACGTCACCCTTTCTAGGGTTGAAAGCTCCAGGCAATGGTGGATTTGCCTCGAACTCTTGCCTCAGTTTGGCACACAACGAATCCAGTTTGGGGCCGTCCGCGAACCTCTGTACGAAGAAACTACCTTCCGGTGTTATCTCGGTGACGACCACTTCATCGAAGTTTACTTTCCTCTCGACGTTCACTTTTTCTTCTTCGACCTTCTCTTTCACTTCCTCCTCGACGTAATTCTTCCACCGTCTCAACTTTTGCGATTTGGCGCTCTCCTCGGCCTGTTTAAGCTGCGGGGCATAAGCCGACTTTTCTCCGGTAAAATGTACGCTCGCGAATCCTTCTTTGACGAGAGCTACCGACAAATTAACGTTATCGATCCACAACCAACCGATAAAGTTGCCGGCCTTGTCGTGAGTGTCCACTTGAATCGACACTTCGCGCTGCAGACAGCGTTCTTTCGAAAACTGTAGAGCCTCGTCTCCGAAAGCTTCGCCCTCGGATGCCGGAAGGGTTCCAGTCGCTGGTCTGCTGGCTCTAGGACAATTGATACCCCCCAGCAAAAACGTGCACAGACTGTTGGACTTTGGAATATACAGCCTGAATCGAGATCCGCTAGCCACGAATTCTACGATGGCATCTATCCTTTGGGCCCTTTGGAACGTGGCGAGCTCTAATTTTGCCCTGGCAGCGTCAATTTCTGTGACTCTCAATGGAGCACTGTCTTTCTTAGAATGTAGACCCAATTGGGACTTTTCGGCCTTCGATTCTGCTTTGACCAACTCGTCGTAACGACAGCTCCTCTGATCGTCGTCCTGTCTGTATCGCACAACGGTCGCCAAACCTTTCGAGACTAACGCTTCAGCCACATTTTTGTTATTAGACAGCACAGTGGCGCAAACCTTCTCCGGAAACCCGTCCCTTGCCTCTTGTACGTAGTCGATAATCACTTGGACTTTTTTTGCCTATCAATTTCTTCCTCAGGTATTCTCTAGCTTCAAACATCCACGGAATGTCGTAAAGGGGCCTGAAACCTTTCGGTCTCGGCAGTGGTTTCCCTTCGTCGTCGTTGGCCTTGCCCGCTTCTCTCGGCGGTCTTATACTTGCCAGGAATATTTTCTTCAAAGTGCCGTTGGCCAGTTTGACTTGCAAAGCGTCCCCGTTTACAACTTCGACGACGGTCGCGGTGAACTCTTTCTCTTTGCCCGTTATCTGAGGTACGGTCGATTGCCAACCTCTCCATAGTCTTTTTTGTTCTTTCTTTGCCTGACGTTCGGCTGCCCTTAGCTTTTCTACAACGTCAGATGGCAGAGGGGCTAAGGACCAGTCCACGCATTTAGCGAATCCCTCTCGGACGAGGTATTC

Protein RF -1&-2: 2395 -> 517 (626AA)

Comparison with *Tribolium* PREDICTED: similar to ebna2 binding protein P100 (900AA)

Range 1: E=0.0; bits=201

Query 2395 EYLVREGFAKCVDWSLAPLPSDVVEKLRAAERQAKKEQKRLWRGWQSTVPQITGKEKEFT 2216

E L++EGFA CVDWS+A + S V E LRAAE++AK + R+W+ WQS PQ+TGKEKEF+

Sbjct 275 EALLKEGFAHCVDWSIAFMKSGV-EGLRAAEKKAKMARLRIWKDWQSNAPQVTGKEKEFS 333

Query 2215 ATVVEVVNGDALQVKLANGTLKKIFLASIRPPREAGKANDDEGKPLPRPKGFRPLYDIPW 2036

ATV EV+NGDAL VKL NG KKIFL+SIRPP+E G+ D++GK PRPKGFRPLYDIPW

Sbjct 334 ATVAEVINGDALSVKLNNGQYKKIFLSSIRPPKEPGRVADEDGKTAPRPKGFRPLYDIPW 393

Query 2035 MFEAREYLRKKLIGKK 1988

MFEAREYLRKKLIGKK

Sbjct 394 MFEAREYLRKKLIGKK 409

Range 2: E= 0.0; bits= 735

Query 1995 AKKVQVIIDYVQEARDGFPEKVCATVLSNNKNVAEALVSKGLATVVRYRQDDDQRSCRYD 1816

KKV V+IDY+QEARDG+PEKVCATV KNVAEALV+KGLA+VV+YR DDDQRS +YD

Sbjct 407 GKKVHVVIDYIQEARDGYPEKVCATVTVGGKNVAEALVAKGLASVVKYRPDDDQRSSKYD 466

Query 1815 ELVKAESKAEKSQLGLHSKKDSAPLRVTEIDAARAKLELATFQRAQRIDAIVEFVASGSR 1636

+L+ AESKA KS +G+H+KKD RVTEIDAARAKLEL++FQRAQRIDA+VEFVASG+R

Sbjct 467 DLLAAESKAMKSGIGIHNKKDVPIHRVTEIDAARAKLELSSFQRAQRIDAVVEFVASGTR 526

Query 1635 FRLYIPKSNSLCTFLLGGINCPRASRPATGTLPASEGEAFGDEALQFSKERCLQREVSIQ 1456

R++IPKSNSLCTFLLGGINCPRASR AT PA EGE FGDEALQF+KE+CLQREVSIQ

Sbjct 527 LRVFIPKSNSLCTFLLGGINCPRASRQATNAQPAVEGEPFGDEALQFTKEKCLQREVSIQ 586

Query 1455 VDTHDKAGNFIGWLWIDNVNLSVALVKEGFASVHFTGEKSAYAPQLKQAEESAKSQKLRR 1276

VDTHDKAGNFIGWLWIDNVNLSVALVKEGFASVH TGEKS YA LK+AE+SAK +LR

Sbjct 587 VDTHDKAGNFIGWLWIDNVNLSVALVKEGFASVHRTGEKSQYAALLKEAEDSAKQHRLRI 646

Query 1275 WknyveeevkekveeekvnverkvNFDEVVVTEITPEGSFFVQRFADGPKLDSLCAKLRQ 1096

WKNY EE+ + EEEK NVERKV+++EVVVTE+TPEGSFFVQ ++GPK ++L AKLRQ

Sbjct 647 WKNYEEEKEEPHAEEEKPNVERKVSYEEVVVTEVTPEGSFFVQTISEGPKAEALNAKLRQ 706

Query 1095 EFEANPPLPGAFNPRKGDVCAAQFTLDNEWYRVKVEKVQNGKATVHYIDYGNREVLPTTR 916

EF+ANPPLPGA+ P++GD+CAA++T+D+EWYRVKVEKVQ GKA+VHYIDYGNRE LP+TR

Sbjct 707 EFQANPPLPGAYTPKRGDICAAKYTVDDEWYRVKVEKVQGGKASVHYIDYGNRETLPSTR 766

Query 915 LASLPAAYSADKPFATEYVMPYVSLPKDEDDRELALKYLREDTNVSKLYLNLEYRNQGSP 736

LASLPAAY+ +KP+ATEY++PYV+LPKDE+ +ALKYLREDT VSKL LN+EYR QG P

Sbjct 767 LASLPAAYAGEKPYATEYILPYVTLPKDEEYAAMALKYLREDTAVSKLLLNVEYRVQGGP 826

Query 735 PAASLHKDTSGASDIFQALIAEGLLLVDNIKSRRQNKLLEDYKQAQDQAKKEHLNIWQYG 556

AASLH D + DI + LI EGLLLV+N K RRQNKLL YK+AQ+ AK+ H NIW+YG

Sbjct 827 SAASLHTDNTAEGDIIKNLITEGLLLVENRKERRQNKLLGAYKEAQEVAKRNHSNIWEYG 886

Query 555 DIREDDAKEFGLG 517

DI EDDAKEFGLG

Sbjct 887 DITEDDAKEFGLG 899

Graphical representation


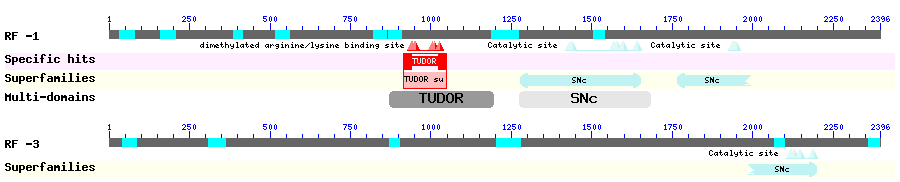


**VIG (Vasa Intronic Gene)**

>Cp.comp31480_c0_seq2 len=2837

AGCTTCAAACCTTATCATCAGCCGAGTGTAGTCAACATCCTCACCACTAATAAGTGCAAGATGATCTTCTGCGGAGCCGGTGCAATTTTGTTCTTAGTCAGCGCCTCTTTGGCGTCCAACATTGCAAAGAGAGGCGTATTTGACAGCGGTTATGGATATAATTCTTTCGACAGTTACGGACCGTCAAGCTCTGGTTTTTCGAGTCCCAGCATAAGCTTCAAACCTTATCATCAGCCAGTATCTCCAGCCGAAATTGCATCGGCAATCCAAGCCGCCAAGGAAGCCTCGTCTGCTGTCATGGCCGCTCAGCGAAGGGTCCAGGAAGCGAAGGAAGACGTTTTACATCAACAACACGTAGCCAGTCAAAAACAGGCCGACGCAGCACTCGCACTACAGAAAACTGAAGCAGCCGCCGCGGTTCAACAACAAGAAGCTAAATCGGCAGCAACGGCTTTAGTGAGAGCCCAACAAAGGTTAGCTCAAGCCAAAGCCGAAGTTTCCGAAGCCCAGCGTATGGCCGCCGCTAAAGAAGCACAGGCCGCCGCCATTATTCAAAAGAGTGCTGCGGCTGCTGCACATGAAATTCAAAAAACAGATGACGCCGCTCGAAAAATTCAATCAATTCAATCTTCGGGTAGTTCGGCTATTCGGCAAGCGGCCGCAGCCAAACAAGGCTCTTTGACCGCTACGTCCGCAGCTGTCGCGGCTGCAGCTTCGTCCGGCGACCACGCGGGTGGTTCGGACGGTTCCGCCGGTCCTTGGTACGGTCAGAAGGTTATCCATTTGCAGCCATGGGGTTAAACCGCATGTACTGATGGCCTTGAGACGACTCATTGTTTATTAATTATTATGGTTTTTTGATTTATAAATACCCTGACTTGTTTTAGTATTAGGATTTGTTTATTGTTATATATCTTGTACAAATATAATGACATGTTGACGTTAAAAAAAAAAAAAAGAAAAAAAAACAATTAAAGACCATATTATGAGCAATCATGTATCAAACTGTCAACAATGTGTACAGTGACTAGTAACCGTTTCCTCAATTAGGGCACACAGTCTAAAATGATACATGTAGAAACCTAGATTAAGATTTCTAAAAATTTTATTTAATTATGGCTGAAAATATTTTTTAAAATTACTTTCGATCCATTCACAATACATGATTAAGTTTGCCGACACCAGATTTTTGCAGTAATTAATTAAAACCATAACAATAACTTCGATTATAGCGATTTAATAAGATGCTCAAAAAAAAAACACTCAAAAAGTCTCAGAAAAATAAGGTGTCGGCTTAAAAAGTTTCGGTAGGTAAATTTACCGAATATTTTTCATGTTCATCCCAATGTAACAAAGTAGTTAAACGTATTATACAAATTAATTTTCATGTGACGGGTTGTGACCATCGAAACATAAGGCAGCAAAAAGCTACGTGTGATTATAAACATCTCTTTAAACAAGTGATGGAAAGTCGCGTTCGTCGTCGACTTTTGGAGCCCTGGGAGTACCTTTTTCGTCGCCCTGTTCTTCTCTAAACCGTGGTCTTTCGCCTCCATCTCTGGGCGGACCTCCGCCACGTTGACTACCTCCTCGGCCTCCTCTGACTCCAGATCTTTGTCCTCTTCCACGACCACCGCCAGGACGCCTATTGTCGTTAAACTGAATGTCGATTGAGTGACAAAGTTTTTGTCGGCCAGCTCGTTGGGGATACTCTGAAGCGTCAAACTCTTCTTCTTCCGATTCTTCTTCTTTTTCTTTTTTCTTAAGCTCAAACATCTTTTTCCATTGGCTCGGGTCTTCTCCTTCTCCAGCTTTCCGTATATTATATTGTGGCTTAGCACGTCCTGCGCGTTGGGCCTTCCATTCGTCCAAAGTCAATTCTTTAGGCTCTTCCTCAGCTGGAACAGCTTCCACTTCTGGTTGCTCTTCCTTCTTTTCCACTTCGATAGGTTCAATTTTTTCAGTTTCTCCCCAGCTCTGATCAGCTTCACTAGGCCTCTCCGTTTCAGCTTCAATAATGTCTTTGTGAGAACCCCAGTTGTGAGCTCCAGCGCCGTCTCGTTTTTCTATAGGCTTAACTCCTGTCTTGTCAGATCCACTTTGCCTGTCAAATTCTCGTTTACCTCTTCTATCGTCAAAAGTTTTTCTAGGGCCGCGGGGTCCACCTGGGCGATTACCAATTGCTCTGCCTTCTCGTCGTTCACCTCGTTCTCCTCTATCCCGATTGCGATTTTCAAAATTCTCAGAATTGTTTTCACGCCTCGGTCTATTTTCACGATCACGATCACGGTTTTCAGCAGGTCCATTGTAAGGTCGATCCTCGCGGTTTCTCCTATTATTCCTCTCTTCACGATTTTCATTATTAAATTTGGTAAAAGTCCTCTCCGGTTTTCCATCAACAGCAGCTCTTTGATTCGGTTTATTGTCTTCACGTTTATTTTCTTGAGCCTTATTAATAGTCGTTTCCTTGATTGGTTTCTTCTGGACATTTGCCGCCGGTTTCGCCTTGGGTTGTTCGTTTTTACCTTTGTTTTCCTTTTCCGCTACTTTGGCTTTTTTCTTGAGCTCTTTCTCTTGCTCTTTTATGGATAGCGTTTCCAACGGATCCGATTCATCATCCAAAAAAAGTGCGTACCTGTTGGCTACGCCAATCCCGTACGAATTTTCCATGATGGTCGAAACGGCAAAAAGTCTTCGCAACTTTGCGATGTTGGAAGATTTATTTTTTGGCTACTTCAGCCCTCTGTTATTCAAGCACTCATTCAATGCAATATGGCCGGTCGCACGAGGATGGCATTGCTTCGCGCATTTGATTGACAGTTTTACCTCCATACGCG

Protein RF -3: -2670->-1453 (405AA)

Comparison with *Tribolium* hypothetical protein TcasGA2_TC001877 (346AA)

Query 82 DNKPN-QRAAVDGKPERTFTKFNNENREERNNRRNREDRPYNGPAENRDRDRENRPRREN 140

D KPN QR+ VDGKPER F KFNNENREERNNRRNRE+R +NGP ENR+R+ R REN

Sbjct 23 DAKPNHQRSNVDGKPERNFNKFNNENREERNNRRNREERTFNGPTENREREERPR--REN 80

Query 141 NSENFENRNRDRGERGERREGRAIGNRPGGPRGPRKTFDDRRGKREFDRQSGSDKTGVKP 200

N ENFENRNR+RGERG GRA+GN+P GPRGPR+ FDDRRGKREFDRQSGSDKTGVKP

Sbjct 81 NGENFENRNRERGERG----GRALGNKPAGPRGPRRNFDDRRGKREFDRQSGSDKTGVKP 136

Query 201 IEKRDGAGAHNWGSHKDIIEAETERPSEADQSWGETEKIEPIEVEKKEEQPEVEAVPAEE 260

I+KRDGAGAHNWGSHKD+IE E ++P++ADQSW E ++ E + ++ P EE

Sbjct 137 IDKRDGAGAHNWGSHKDVIE-EADKPNDADQSWSENDRPE-TNAAPETKEETENETPVEE 194

Query 261 EPKELTLDEWKAQRAGRAKPQYNIRKAGEGEDPSQWKKMFELKKKEKEEESEEEEFDASE 320

EPKELTLDEWKAQRAGRAKPQ+NIRKAGEG DPSQWKKM+EL+KKE E ESE+EE+DA+E

Sbjct 195 EPKELTLDEWKAQRAGRAKPQFNIRKAGEGVDPSQWKKMYELRKKENESESEDEEYDAAE 254

Query 321 YPQRAGRQKLCHSIDIQFNDNRRPGGGRGRGQRSGVRG----GRGGSQRGGGPPRDGGER 376

YPQR GRQK IDIQFND RR G + G G GER

Sbjct 255 YPQRVGRQKHVLDIDIQFNDTRRGAGRGRGQRSGPRGNRPTQRGGTGTGTGTGAAPAGER 314

Query 377 P--RFREEQGDEKG-TPRAPKVDDERDFPSL 404

P R+R+EQ DE+ RAPKVDDERDFPSL

Sbjct 315 PERRYRDEQADERTENRRAPKVDDERDFPSL 345

Graphical representation


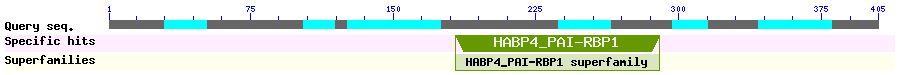


**Similar to fragile X mental retardation syndrome-related protein 1**

>Cp.comp38219_c0_seq6 len=4251

TCGGTCGGTTTTACGCATTTTCTAAAAATTACACAAGCTAAAGTTCGACACACGCAAGACAATCACAGTAGACTTTGAAAAGCTTGACAAAAAAACCTTTGATTATCTAAAAGTCACTTGTCACAATTGTGCCAGAATTTGCCTCGAAGATGGTGTTTATGAACGACGGCATTGCGACTCGCTCTCGGCGGCTCAAGGCTGTTTCCTCACCCAAGAATCAAACCAACATGGAGGACCTGGCTGTAGAAGTTGTCGGCGAAAATGGGGCTCTTTATAAGGGCTCCATCCTGGACGTACACGATGACGGCGTTCTCGTTCACTTCGAAGACGAGTGGCAACCGGACTCGAAATTTCCCTTCGAGCAGGTCAGGCTACCCCCCAACCCGGACCAAAAAGTCGAGTTCACGGAAAATATGGAGGTGGAGGTGTTCTCCAGGTCGAACAACCAGGAGGGCTACGGCTGGTGGAAGGCGAGGATTAAGATGATGAAGGGCGACTTTTACGTGCTCGAGTACTTGGGCTGGGACAGCACCTACACGGAAATCGTCAGCATCGACCGTCTCCGAATGAAAAACAGCAACCCGCCGATCGAGCGCGGCATGTTCCACAAGTTCGAAATCGAAGTTCCCGAAGACGTCAGAGACTACTGCAAAAAGGACACTTACAAGGAGTTTCAGAAGGCGATAGGCGCGGGCCGTATCTTCTACAACCCCGACAAGGGCGTGCTCGTTGTCATATCCAGGTGCGAGTCGAGCCGGAAACGCGCCTCCCTACTGCAAGACATGCACTTCCGCAGCCTGCAGCAGAAAGTCCTTTTGATGAAAAGGACCGAAGAGGCAGCCCGGCAACTGGAGAGCACCAAACTGGCGACCATAGGCGGCTTCTCGGACGAGTTCCACGTCAAAGAGGACCTGATGGGTCTGGCGATCGGCGCCCACGGCGTCAACATCCAACAGGCGCGCAAAGTCGAAGGTATAACCAATATCGAGCTCGAGGAGAACTCGTGCACGTTCAAGATTTACGGGGAGACCGACGAGGCGGTCAAAAAAGCTCGGTCGATGCTCGAGTACAGCGAGGAGTCGCTACAAGTGCCGAGGGCGCTCGTCGGTAAAGTTATAGGCAAAAACGGTCGTATAATCCAAGAGATTGTCGACAAAAGTGGAGTGGTGCGGGTCAAAATCGAGGGAGATAACGAGCCGCAACCGACGTTCCCGAGGGAAGAGGGGCAGGTCCCGTTTGTCTTTGTCGGCACGGTCGAGAGCATATCCAACGCCAAAGTGCTGCTGCAGTACCATTTGGCCCACCTCAAGGAAGTGGAACAACTAAGACAGGAAAAACTCGAGATCGACCAGCAGCTGCGCAGCATCCACCACGGCTCGAACCTCGGTTCGATGCAAAGTCTGTCGATGAGCGGACGACGCAACGACCGCGGCTACAATTCGGACATGGACGGCCGTAGTTCGGGCCGGGGCGGTTCGATGAGGGGTCGCGGGGGCGGCCGCGGCAGGGGCGGCCCCAGGCAGAACGACCGCTACAATTCGGGATCCCGTCACCAGACGCCCGACACCGCCGACGAGCGCGTCCAGGATGGGGGCGGGCCCCGAAGATACGGCGGTTACAGGGGCGGACGCGGCGGCGGCGGCGGCAGGGGTAGAGGAGACGCGCGTAGGGGCGGCGAGGACAGGCGCAGGAACAATGACGACGAAGAAACGGTACTGGACAGTCAAGAAGTGTCCAGCGCGGACAGGGTGAACAATCACTCGGATCAGGCGCCGGCGACCGACAACGGCGTGGACAACCAGCCCCGCGGGGGCGGGCCGAAGAACAGGTACAACAGGGGGCCGCCCCCGCGCCGCGGCGGCGGCGTCGACAAACACCAGCAGCATCAGCAGCAGCAACAGCAGAAGGAGGCTTTAGTCAACGGCACCTCGGCTTAGGACCGGTCATTTCTCCCGAAGTATTAACGCAAGTCGGAGTCTTTATCGTGCGCAGAGGTCGGAAACGGGACTTGAGTTTCTATTTTTTTGTTTTTTTTTTTTTTTAAAAAACGAAATCGTTTATTGAAACGTGATAAATAAAACAAATTGTCAGTAAAAAATCGAAAATAAATAGCTTTCTTAATACTTTAAAGGTGCTTTATTCGTAGCACTCTTTCTCGGTGCGAGGCAGCATGTGGTTGTGGAGGGCGATGAATTTACGGAGTTTGCAGAAGTTGCCGCAGCCTGGTATCTTCAACCTCCTAGGTTTCTCGGACGTATAATCTTGGTAGAAGAATTTGAGTCCTCTAACTCCGTTCACGTTGTGCACCTCTATGACGACGTAACTGCCGTAGGGCGGCACGTGGGGGTAAAACACACCTAGGAATCTCAACAGAACGGCCACGTTGAACTCGTGGGCTGAATAGAGGAATATTTTGGTGTCTTTAAAGTATTCGCCTTTGGCTTTGGTTTTGGTATCGTCTATGATCTTTTTGACGAGGAATCCCGTGCAGTATTTCTTCAATTCCGAATTGTAAGTACTGACGTTGTAATCTTTAATAGCCAGTTGCAGAATGTGGTGGTAGAAGTCTTTGGCCCACTCCGGGTGGTCATATCCGTTTTCTTTCTCAGTCGTCAAGGTGAAGTAAAGGTCGGCAAAGGCTTTGCTGTTCAATTTCTGACCGGTATGCTTCTCCAAGTATTCGATGACGTCCGTGTAATTGTCGTACAGTTGTCTGCCTTCAGTTGAATTCAAAAAAGACCAAAACAGTCTGTTGTACTTCGGACAGTTCTTCAGAGGATCGGCCAGTACGTGGTCTTCATGCACCGGCCAGTAATTGAAAGGTACTGGTTGCCAATCGATACTGTTCTCCCAGACTAGGTCGCCTCTGGGTGGGAACAAAGACGCCAGCACCAGCTGCAGCGACATTTTGGTCCTGTTGTAGTCTGTGCATCTGGCGTCGACCATGTCGAGGGTAAACTCCTTCCCCAAGAAGTCGTAGTACCTCTTCCTTAGCTCTTTTCCTATGTTGTACTCTTTTCGTTTGCCTGCGTTGGTCAATTGCCCATTCCCTATCGGGTAGTACGTGACATTGATGTAGGGGTCGTTCTTGTAAATGACGTGCTTGTCGGGCGTGCGGTCGCCATGTCTGAAGACTACGTGGACGAGTTCGAGGGTGTCCAGGTTGTCGCTGTCGTCCCTGGACAGTGTCCCGCACCGGACACAGTTCAGAACCGATACAAAAATAACGAATGCCGGGGCAAACGTTAATTTGTGGACCATGTTTTGCGTCGAAGACGTTTGGGACGGATAGGCCGTAGACTGACAACCGTATCATATCTGGGGTCAATATTTAAACGGACCGTTATCAACGGGGACGCGATAAGCGGCGTCCACTTGTGGCTCTGCCAGATAAGAATCACGCAGCACTACGCAGTATTCGGACCGTGGACGGTTCGTAGAGTGGAGAAAATTACATTTTTGTTGTTGTTGTGTTTATCGTTTTAAAAATTCTATGACGGTCAAATGTCGACTATGTATAATTTGTGGATGAAGCGGGCAGAAACTTGACAGCTGTCAAATGTCAAAGATACAGCGCCATCTTGGGTTTTGTTGAACATGAGATTTTGGTTTCGAAATTGTTGTGTTTCAAACACTTTTGTTTACAAGTTCTTTATTGTTGCCCTTCCGAAATGATTTTTTCATTTTGAAATCAACAATATACGGTTCGTTGACGGCGCAGAGTGGAGACAATTGGAAATCTGTTTTGTTATTGTTTTGTTTATCGTTTTAAAGCTTCTATGTTGGTAAAATGTCAACTAGGACATCATTTTTCTGTGTATAAAGCGGGAAGAAACTTGACAGTTGTCAAATGTCAAAGAGACAGCGCCATCTTGGGGTTTATTTGTTTTTGACGGATCTTTCAATGTTGGTTTAGAAATTGTAGTGTTTTAAGCACTTTCTGGAATCAAAATACATGGTTCGTTGCCTAACCGAAATCCAGAGGGCGTCTAGACGACTGACAAGTTTTCGATTCCTGCGCCATTTTGCACACTTAATGCTCCAATTTGTGAGAAATCCATGGGATACGTTTTACTAATTTCTAAAATTAACTTAAAAATATTTGTTCCGGGTTCGGAAATCGGTCGATATAAAAAATGACGACAGACAAACTAATCTTTAGTTAAAAGTTAATTTTATTAAGATTTATTGCAGATTTTTTACAAAAATGTATTTTTGGTAAACACTAAACTCAAAAACACATGAAAATTG

Protein RF 3: 150->1937 (595AA)

Comparison with *Tribolium* PREDICTED: similar to fragile X mental retardation syndrome-related protein 1, putative (660AA)

Query 27 MEDLAVEVVGENGALYKGSILDVHDDGVLVHFEDEWQPDSKFPFEQVRLPPNPDQKVEFT 86

MEDLAVEV GENGALYKG ++DV +D VL+HFEDEWQPDSKFPF QVRLPP PD KVEFT

Sbjct 1 MEDLAVEVCGENGALYKGYVVDVFEDSVLIHFEDEWQPDSKFPFSQVRLPPKPDPKVEFT 60

Query 87 ENMEVEVFSRSNNQEGYGWWKARIKMMKGDFYVLEYLGWDSTYTEIVSIDRLRMKNSNPP 146

ENMEVEV+SR+N+QE YGWWK+RIKMMKGDFYVLEY+GWD+TYTEIVS DRLR+KNSNPP

Sbjct 61 ENMEVEVYSRANHQEAYGWWKSRIKMMKGDFYVLEYVGWDTTYTEIVSDDRLRVKNSNPP 120

Query 147 IERGMFHKFEIEVPEDVRDYCK-KDTYKEFQKAIGAGRIFYNPDKGVLVVISRCESSRKR 205

I+ MF KFEIEVPEDVR+Y K ++ +KEFQ AIGA I Y P+KGVLVVISR ESSR+

Sbjct 121 IDSSMFVKFEIEVPEDVREYAKIENAHKEFQNAIGASLIRYVPEKGVLVVISRNESSRRC 180

Query 206 ASLLQDMHFRSLQQKVLLMKRTEEAARQLESTKLATIGGFSDEFHVKEDLMGLAIGAHGV 265

A L+QDMHFRSL QKVLL+KRTEEAARQLESTKLATIGGFSDEF+V+EDLMGLAIGAHG

Sbjct 181 ARLVQDMHFRSLSQKVLLLKRTEEAARQLESTKLATIGGFSDEFNVREDLMGLAIGAHGA 240

Query 266 NIQQARKVEGITNIELEENSCTFKIYGETDEAVKKARSMLEYSEESLQVPRALVGKVIGK 325

NIQQARKV+GITNIELEENSCTFKIYGETDEAVKKARSMLEYSEESLQVPRALVGKVIGK

Sbjct 241 NIQQARKVDGITNIELEENSCTFKIYGETDEAVKKARSMLEYSEESLQVPRALVGKVIGK 300

Query 326 NGRIIQEIVDKSGVVRVKIEGDNEPQPTFPREEGQVPFVFVGTVESISNAKVLLQYHLAH 385

NGRIIQEIVDKSGVVRVKIEGDNEPQPT PREEGQVPFVFVGTVESISNAKVLL+YHLAH

Sbjct 301 NGRIIQEIVDKSGVVRVKIEGDNEPQPTIPREEGQVPFVFVGTVESISNAKVLLKYHLAH 360

Query 386 LKEVEQLRQEKLEIDQQLRSIHHGSNLGSMQSLSMSGRRNDRGYNSDMD---GRSSGRGG 442

LKEVEQLRQEKLEIDQQLRSI HG+ LGSMQSLSMS RRNDRGYNSDMD G

Sbjct 361 LKEVEQLRQEKLEIDQQLRSI-HGNALGSMQSLSMS-RRNDRGYNSDMDGGGRPGRGSMR 418

Query 443 SMRGRGGGRGRGGPRQNDRYNSGSRHQT 470

GRG G G G RQNDRYNSG+ T

Sbjct 419 GRGGRGRGGGGPGGRQNDRYNSGTSTIT 446

Sbjct 617 MTDGGANQKPRGGGDPKNRSNRPPPARRNN----------EPKPKEALVNGTTA 660

Graphical representation


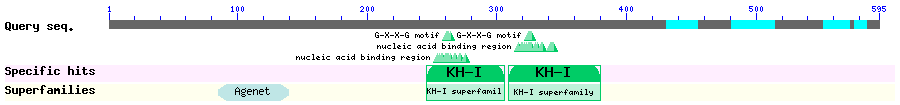


**Drosophila homolog of p68 RNA helicase = ATP dependent RNA helicase p62 (Tribolium)**

>Cp.comp36480_c0_seq1 len=1977

TTTTTTTTTCCCAAATCTCTCGACCCAACGGCTCTTCTGCTGCTTGATATTTCTCTTTAGGTTTGGACATTGAAGAGATCGATCTCTTCGTCCTTAATTAACGGACGCCGAATAATTGAAATTAACCAGAGAAATCTACTTCTATCCGCAGCAATAAACATGGCATTTGAAAGACAGCATGGAGGCAGTTTTAGAAGCCGCGACGGCTTTAGCAATTCCAGAGATAAAGGTTATGGCCAAAAGAGCGGCTACGAAGGCGGTAGCTACAGAAATAGCCGAGCCGGTTTCAACAACCGACTCAGAACTGTAGAATGGAGCAGTAAGCAATTGCGTCCATTTAAAAAAGACTTCTATGTGCCCCATCCATCGATATGTAGTCGTTCAACTTTCGAAGTCGATCAGTTCCGCAGCGCAAAGGAGATAACTGTCGAAGGGGAAGCCCCAAAACCCATCCAAAGTTTCCACGAAGCCAACTTCCCGGATTATGTAATGGACGAAATTGTATCTCAAGGCTATGAATTTCCCACTGCTATCCAAGCCCAAGGATGGCCCATCGCTATGAGCGGAAATGATATGGTGGGGGTCGCTCATACTGGCTCTGGCAAAACTTTGGCATACATTTTGCCGGCTATAGTTCACATTAACAATCAGCCGGAGGTGCAAAGGGGCGACGGTCCAATAGTGTTGGTGCTTGCTCCAACAAGAGAATTAGCTCAACAGATCCAACAAGTCGCTAATGATTTTGGCCGCAGAAGCAAGATACGTAACTCTTGCGTCTTTGGAGGAGCCCCCAAAGGGCCCCAGGCCAGAGACTTGGAAAGGGGCGTGGAAATTTGCATCGCAACCCCCGGTAGATTAATCGATTTCTTGGAAAAGGGCACCACCAACTTAGAAAGGTGCACTTATCTGGTGTTGGACGAAGCCGATCGTATGTTGGATATGGGTTTCGAACCTCAAATTAGGAAAATTATCGGCCAGATAAGACCCGATAAGCAGACTCTGATGTGGTCGGCAACTTGGCCGAAAGAAGTTAAGAAACTGGCCCAAGATTTTATGAATAATCCCATTCAATTAAATGTGGGCTCTTTGCAATTGTCGGCCAATCACAATATTTTGCAAATCGTAGATGTATGTCAAGAACACGAAAAGGAATCGAAGTTAAATAATCTCTTGCAAGAAATCGGCACAAACGGTGAACCGGACGCGAAAATAATCATTTTCGTCGAGACGAAAAAGAAGGTTGAAGGAATCACGAAAACCATCAGGCGATTGGGATGGCCAGCAGTTTGCATGCACGGCGACAAAAGCCAGCAGGAACGCGACTACGTTTTGCGGGAATTTAGGAGTGGAAAATCAACCATCTTGGTCGCTACCGATGTTGCAGCTCGTGGATTAGATGTGGATGGTATAAAATATGTAATAAACTACGATTATCCCAATTCGTCCGAGGATTACATCCATAGAATAGGAAGAACCGGACGATCAGACACTACCGGCACATCATATGCGTTTTTCACTCCGTCCAACATTAGACAGGCCAAAGATCTCGTCTCGGTACTGAAAGAGGCAAACCAGGTTGTCAACCCTAAACTGTCCGATATGGCAAGCAAATCTAGCGGTTTCGGTGGTTTTCAAAAAGGGGGCCGTTGGGGCAATGGCGGTTCATACAGAGGCAGAGAAAACAGCGGACCAAAACACACCAGGTGGGGAGGTGGTTCTGGAGGATACAAAGCCAACAATGGATATGCGAAGAGTTATTAAGAGGCACTTTAGTTCCGAATTTCCATTCAAGTTCAGTATTTTTCTTCACATATTCCACACACTAGGACAATTGAAATTTTCTATTTGGTTAGAAAATGTGAGTTTTATTTATTTCTGTGGCAATCTCTGTCTGTCATGATTGTAAATCTGTTTATAACTGTGATAGAAGTAGATGTCTTCGTTTTGTGTAATTCGACGTTTAATAATAAAGAGTTT

Protein RF 1: 160->1761 (533AA)

Comparison with *Tribolium* ATP-dependent RNA helicase p62 (549AA)

Query 1 MAFERQHGGSFRSRDGFSNSRDKGYGQKSGYEGGSYRNSRAG------------------ 42

M++ +Q+GGS+R R G ++G+ GG+ RN G

Sbjct 1 MSYGKQNGGSYRGR-----------GSENGFGGGASRNGFGGGSRFKNGGGGGGGSRFGG 49

Query 43 -------FNNRLRTVEWSSKQLRPFKKDFYVPHPSICSRSTFEVDQFRSAKEITVEGEAP 95

NRLR W K LRPFKKDFYVPHP++ +RS +EV+Q+R +KEIT++G+AP

Sbjct 50 RSGGGGSPGNRLRKPNWDMKNLRPFKKDFYVPHPAVANRSKYEVEQYRRSKEITIDGDAP 109

Query 96 KPIQSFHEANFPDYVMDEIVSQGYEFPTAIQAQGWPIAMSGNDMVGVAHTGSGKTLAYIL 155

PIQ+F EA FPDYV EI QGY+ PTAIQAQGWPIAMSG D+VG+A TGSGKTLAYIL

Sbjct 110 NPIQNFEEACFPDYVQHEIQKQGYDTPTAIQAQGWPIAMSGKDLVGIAQTGSGKTLAYIL 169

Query 156 PAIVHINNQPEVQRGDGPIVLVLAPTRELAQQIQQVANDFGRRSKIRNSCVFGGAPKGPQ 215

PAIVHINNQP + RGDGPI LVLAPTRELAQQIQQVA+DFG S +RN+C+FGGAPKGPQ

Sbjct 170 PAIVHINNQPSIARGDGPIALVLAPTRELAQQIQQVAHDFGSSSYVRNTCIFGGAPKGPQ 229

Query 216 ARDLERGVEICIATPGRLIDFLEKGTTNLERCTYLVLDEADRMLDMGFEPQIRKIIGQIR 275

ARDLERGVEICIATPGRLIDFLEKGTTNL+RCTYLVLDEADRMLDMGFEPQIRKII QIR

Sbjct 230 ARDLERGVEICIATPGRLIDFLEKGTTNLQRCTYLVLDEADRMLDMGFEPQIRKIIEQIR 289

Query 276 PDKQTLMWSATWPKEVKKLAQDFMNNPIQLNVGSLQLSANHNILQIVDVCQEHEKESKLN 335

PD+QTLMWSATWPKEV+KLAQDF+ N +Q+N+GSLQLSANHNILQIVDVCQEHEKE+KLN

Sbjct 290 PDRQTLMWSATWPKEVRKLAQDFLRNYVQINIGSLQLSANHNILQIVDVCQEHEKETKLN 349

Query 336 NLLQEIGTNGEPDAKIIIFVETKKKVEGITKTIRRLGWPAVCMHGDKSQQERDYVLREFR 395

NLLQEIG NGEP AKIIIFVETKKKVE IT+TIRR GWPAVCMHGDKSQQERD+VLREFR

Sbjct 350 NLLQEIGNNGEPGAKIIIFVETKKKVESITRTIRRYGWPAVCMHGDKSQQERDFVLREFR 409

Query 396 SGKSTILVATDVAARGLDVDGIKYVINYDYPNSSEDYIHRIGRTGRSDTTGTSYAFFTPS 455

+GKS+IL+ATDVAARGLDV+GIKYVINYDYPNSSEDYIHRIGRTGRSDTTGTSYAFFTPS

Sbjct 410 NGKSSILIATDVAARGLDVEGIKYVINYDYPNSSEDYIHRIGRTGRSDTTGTSYAFFTPS 469

Query 456 NIRQAKDLVSVLKEANQVVNPKLSDMASKS----SGFGGFQKGGRWGNGGSYRGRENSGP 511

N RQAKDLVSVLKEANQ +NP+LS+MA++ S G GGRWG GGS+RGRENSGP

Sbjct 470 NFRQAKDLVSVLKEANQAINPRLSEMANRCSSYGSKGGSGGGGGRWGYGGSFRGRENSGP 529

Query 512 K-HTRW--GGGSGGY 523

+ H R+ GG S GY

Sbjct 530 RNHQRFTNGGRSNGY 544

Graphical representation


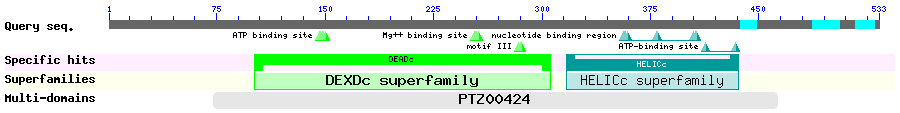


**Translin**

>Cp.comp40752_c0_seq1 len=6047

TAATCAACAAGTAAACAAACGTTGGTACCTTATTTTACCAAAGTAAATTCAAGATTGGTGGTATTGTCTATGATTCAAGAGCTATTTGTAGTTTTTGCCATTGAGTTGCTGCCGCCTTTTTTTTTTGTACAAGTCCGTTTCAATATTTAAAAGTGCAATTTTAAACTAAATAATAATTGTACTAAACTATAATGTCAGAAGCAAATAAAGTTATTACTGACATTTTTGTCCCATTCCAGGAGTACATAAATGCCGAACAGGACGTTCGAGAAGAAATTCGAAATATATTAAAAACCACTGAGAAGTTTTTAAGAGAAATACACACATCTTTACAAATAATCCATTGTGAATCTGATTGTGAACAAGTACATGCAGCTTGTTTAAAAGCAAGAGAACTTTTTGAAGGGGTTCGTAAAGAGTTTGACAATCTGGACAAAATTGTTCCAAATGGACAATATTATAGATATAATGACCATTGGAGATATGCCACACAGCGCCTTTGTTTTCTGGCAGCCCTTGTTGTGTTTTTGGAGCAGGGCATTTTAATTGACAAAATTACAGCAGCTCAGATTTTGGGAGTTCATGAAAAACAAAATATTCATTTGGATCTCGAGGACTATCTCATGGGCATGCTAAACTTAGCTACTGAATTGTCTCGATTTGCGGTAAATTCTGTGACGCTTGGAGATTACAGTAGGCCATTACAAATATCCAGATTCGTAGCTCAGTTGAATGCTGGTTTTCGACTATTAAATTTGAAAAATGATTCGCTTCGTAAGAGATTTGATGCATTAAAGTATGATGTAAAAAAAAATTGAAGAAGTAGTCTACGATTTGTCCATAAGAGGCCTGGTACCGTCCGGTGATGCAAGCACTACCCAAAACGATGGTGAACTAATACAGTAACTTATTAATGATAATAATTTATACTTTTTCTTTCATAAAGCTTAATTAATATGTTACTAGTATTAATGGTAATTGTATGAATTCAAATTAATGTTTAATAATAATTACTGCCAATGCAAATTAAAAATTTACTTTTCCATGTACTAATTATATTGATACTTACATTATACTAGTACCATGCCAGTATGTTTTCCTTAAAGAACTAAGGAATAAAGTGTTCAAATTTGACAACAATGGATACTTGGAATAAAGAAAGAAAAATAAAATTCATTTTAGTAATTTTAATAGAAATAATGATTTTGCACAAAATAGTGGTAGGGGACTGGCAGGCAAAACGGGGTACTTAAAGTTTAAAGTAGGATTTAAAAAAAATCTAAAACTGTGAAAATCTGGAAATAAGGCAATATCATTGCTATAGTACTAACTAATAATAATAATAATACATTTATTGTTTCAAGCCAGTACAATGATGTTAAGTAACAGTATTTATATATGCATTTGGCAAACAATAGGCCTTTAAAAAATGCAGACATCTGGTCCCAGGTCCAGACACTGTTCAGGCCCTAATTTAAATAATAAACATTGTTTTATATAAAAAAACTAAACTAACGCAAATTGAATCTATATATAATTATACACATGTCGTAGAGGACAGAAATAGGAAGAAAGAAAAAGAGTGAGAAAAAGGAGACAAGAAAAGTGATAAATAAAGATTACTGTGAGTTAAAAATTTGTGCTTTTAATTTATTTTTAAATGTCTGAATTGACAAGTCAAAATCCATAAATTCTAAGGTGTTCAAAAGATGTGCGATGTTGTATGAGAACGAACGTTTAAAAATTTCTTTGCGGTGTTGAGGTAGTCTAATAAAAAATCTTTGGCGCAAATTTAGGTTATGTACGTCAGTGCGGAATGTTATTTTGTTATATAAATATGGAGGCGATTTACGTTTTAATTGCCAAAAAAAAAACAAAATAATATAGGCAAAACACTAATTTGACTATTTTCTGAATATTTAATAAATAACTCAACAAAGTATATACAAATTTTAATTATCACAAAGACTAGTAAAAAGATATAACCTCAGTAGCACAACAATATTGTTTGTAACATTACAATTTATTTCGTATGGATACGTACTTATCTGTTAAACCAAGTATATAGACAAGTTTTCTTTTGATATCAAATCCAATCTTTAGATGTTCGTGTATTTTTATGAATATCGGGATAACGGTACTTTTGATAAATAGATATCCTTTAAAATGCTACTGGGCAAGTTGGTTGAACAAATAAACGAATATTATAAATTTTTCAATATGGTGAGTTTAAAACTAAATGGTGCGTGGACAGGGAAAGAAGTGTTGAAAATGAGTGATAAACGTTCCAAACAACCCAGCGCTTCCCGAATGGCGGAAAATCCAAAGGAATCCGAAAAACCAGAAAGTGGCACGGGGGCCCAATGCAGAAACCAAGAGGCGCGCGAGTACATGAAAAAGCAACGCGAGAAACGCTTGCAAGAGTTAAAAGCGAAAAAAAACGAATGCTTAAAGGATGCGGAAATTCGCAAAGAAAAACTTAAAGAATTGCAGAAGAAAAGCAGAGAATTGGTGACAAGAAACGTCGAATTGAAACGATCTCGATCAAAATCTCCTCGTTTAAACAAGGGCAAATTGGAAGGGACAAGGACCAGATCCCTAGATAACATAACTTCAAATTCTGGTCCTAAAGAGGAAATAAAAACGGTGTCAAGTCGTTCTCCGGAACCATTAGTATCGCCGAAGCTCTGCGATCAAAATACTAACCTTTTGACAGTCCCGGATCCATTGCCCCATTTTGGTTTGCGGGACCGTTCCGATAAAATTAATATTTTGTCTAATATTACATTACGATCCGGTAGAAACAGCGCCGAATTAAAGAAAACTGAAGTTCCCATTTGGATGCAAGAAACGGAAGAAACGGATCCCTATAACTTCATTCAACACATCCGAAACCATCCAAAGTGAGGATTTCCGTTTTCCCAAGGAAATCGAAAGCATCCGAAAGAAACTGTTCGATTGTTCGGATTCGTCCAGGCGATCGTCCATTATCACATTTAATCGGGCCAAAATATCGCCCAATAAAGTATGACAGGATGAACTGAAAGTCACTGAGTTTAAGTCTTTATATTCGGATTTTCATCCGTCTCGAACAACTTCGGACAATTCGGAAAAACCTACTGTCGTAACGGATTCGTCCAATCGACCAAGTTTGATCAAGAATATCACCATCAGGTCTCAAAAGCATTCGGAGAAATCACCCCAAAACGAAATTCACTTGAAATTCGAAGCGGAAATCCACCTGTTGAACGATTTCAATCAATCGTTAAAGCAATTTGCGGAAATCGAACAAGCCTTCGAATATTTGAGATCTAAAAACGATTTGACGCCTCCGACGACAAAAAGCGTTCCGTCTTGCAGCCGGTACGACTCCAGTGCGATCTCGTTGATCAATTACAGTCGAGGCTCGGAATTTCCCACCGAAAATAGTATAGTCGTATTAGACGACACTTTATCCGACTTTGATAAGCAAAACAGTCAATCGGTCGTACAATATTCGAGTACGGTCGACGGAGAATCGTCTAATCGGCAGTTCGAAAGTCTGACGGTTAAAAACGTAGCCGGAATATCGCTCAAAATGTTCGATCAGCTGATTAAGGATGAAGACGTACGTTTGGACAATTTAAAAAATATCTTGAAAATACGAGAAAAGACCCTGCTGGACAGGACCAAAGCCGAACTCGCCTGGTTGGAAATACAAAGAAAGCAGCTGAAAGAAACGGGCAAGTTGCAGGAAGCGTCGACGATCAAGAAAAAACAACGCGGCATCCTGCTGAGGCACCAGCAGGAAAAACACGAGATGCAACGGTTAAAACAAATGCAGAAAGAGGCGTCCAGCAAGAGAAAACACATGCTGAGGGAGCACCGGAGTCTGATCAACAAACAATTATACAATGAAAAAATGTTACCGAAACTTAAACCGTCCGGACACAAAGAACGCAGATTATCAGGTCCGATTAAAGTGATCCAGACACATACCAACGATTCCCAAGAAGACGACGTTAATAGAAGTCTGGTAATTAGCAATGATGAGGTGCAGATTTCGAATTTAGACCGCTCCACATCTAAAATCGACGAGTTAGAACCGGCAATTAACATGGATATATCCAATATGAAAAGAACTCTATTGATGCGAGAACGAGCTCTGGAAAAGCGACGGAAAGCGGCCGAGGAGCTCTTAAAATGGCACAAGAAACTTCTGGACGAGGAAAAGGCGATAGTCGCGCTCGAATCGACCGCCAGCAAGATCATTTCCCAAATACCTAGAGACGAAAAAACGAAATTCGGCAAGTTTACCGCGCTACGGAGGAATGTTTCGGAACCGGACGTGACGTGTTTGGGCAGAGACACGGTCTGGCCTATGTCTAGGATTCGTCCCGAGATGACGGCGTCGTCCGGCGCCGTCAACGATGAAATCAGTTCGAATTATAGCGCCGACTTTGAACCGGAATCGGTACCGGAAGACCCGGACGCAGACCGAAGCCGGATATCGTCCATAAACGAGCTCATAGAGAATTTCAGCAAAATCAAAGAGGAAATATCCAATTTAAGCAACTCCCAATCGGAAAAACTATCGGACAATGAAGCCAAAACTAGCGAAATCGTAACTTTAGAAGAAATCAACCAAGAGTCTGAGGAACCGATAGAAAGCGACAACAATGAGGACGAAGACGAGTCACTAACACCAGAACCGGAAAAAGTATCCGTAATAATCGTAGAAGAAGTCGAACACGATAACCCTACGGACGATACGTTTGAACTACCGTCTACTTCGGTCGAACAAACGCCCAAAGAATCTAGCGTAATCGAAGAAGTGGTAAATGAAGAACAATCGATCGTCGAGGAAGACCTTTCCGCTCCTTCAACGAGCCAAGATCCGACCTGCGCGCAGAAAATTTCGACGGATAATAAAACCGACGAGGAAAAAGCCGCGTCGATTTCCAAGACGCAGGAAGATGGTTCGGGTAAAACTGCCGTCGTCGACGTCAAACAGAGGGTTTTTGAAATATTGGCGGAGACGTCGAGGTGTGACGTGAAAAGTCCGCGCATGCAAGATCTGTGCGTCATGACGTACGATTTGACTTCGCCTCCGAATTCGCCCGAACTAGATTCGGCCTCGGAAATAGACAAGAGGGTGTATTTGGGGAGCGTGGCGGAGGAGCTGCTTAAGAAACAGTTGGCCATCGAACAAGAGATCAAATTATTAACGGAACAGCAGGCGATTTATCGCGAGATCCCGAACAAGTCGCCGCCTCCGTACACGCCGCCCACGTCCGCTCGACCGACGCCGACGCCGTCCGTCGTACCCCGTACCAAAGAAGAAATCGAAGAGATCACGAAATATTCGTCGAAAATCATCCACAAAGCGTACCTGAGCGGCAATTTGGCCAACATCGCGATATCCGACAACACCCTTAACCTAATAGCGAAAAACATCGACAAAAATTGTTACAAATACGTGTTCGATTTGTGCAAAGAAATCGCCGTCGATCATTACGGTCGGTTTCGGGAGGAGGCCGGACCGTCGTGGTTTCGCGTTACGAAAAAACCGACGGTCGCGGCGTCGGGACCGTTGGACGAAAACGGTCTGGAGAAATTGTTCAACAAAAAACTGATGGAAATATTCGGGTACGAGAAACGCGACATTAGGGAAAGCACGATCATCAAATGGAGCAGGAAGAAACGAGATCACGTGGACGAACTACTCGTACAAGAGATGCAAGCGGAAGAATCGTCGTGGACCATTTTCGAACGGGACGAGTTGATCGTCAAGAACAGAATAGTGGACGAAATTATGAACGGTTTGTTGAAAGATACGGCCGCGTGTTTGGTGGAAACGATGAGAAAAAAGTACCAAAAGTGATGTTCCGGGTAGGGTGGGCCGGTTTTGTTTTTGTCTGTGATACTGTGTAAGATACGGATCAAAAATAATATTCGTTTAGAAATTACTGTTTTTTTTCTAGTATTTTGTCAGTCGATTTATGTTTTTTAAATGTCTTTTAAGGTTTCATATTTTTGCTAAATAAATTCTTTTGTCAGAAAAAAAA

Protein RF 3: 192->818 (208AA)

Comparison with *Tribolium* translin (226AA)

Query 4 ANKVITDIFVPFQEYINAEQDVREEIRNILKTTEKFLREIHTSLQIIHCESDCEQVHAAC 63

++ ++ +IF PFQE IN EQDVREEIRNI+K EK LREI T+LQIIH + E+ AC

Sbjct 3 SDNILENIFTPFQECINNEQDVREEIRNIMKDIEKPLREIVTTLQIIHRTHNGEE--TAC 60

Query 64 LKARELFEGVRKEFDNLDKIVPNGQYYRYNDHWRYATQRLCFLAALVVFLEQGILIDKIT 123

ARELFE VR ++ LD +VP GQYYRYNDHWR+ATQRLCFLAAL++FLE+G L+DK T

Sbjct 61 FAARELFESVRAGYEKLDGVVPAGQYYRYNDHWRFATQRLCFLAALIIFLEKGFLVDKET 120

Query 124 AAQILGVHEKQNIHLDLEDYLMGMLNLATELSRFAVNSVTLGDYSRPLQISRFVAQLNAG 183

AQILG+HEK +HLDLEDYLMG+LNLATELSRFAVNSVT GDY+RPLQIS+FVA+LNAG

Sbjct 121 TAQILGLHEKSRLHLDLEDYLMGLLNLATELSRFAVNSVTYGDYNRPLQISKFVAELNAG 180

Query 184 FRLLNLKNDSLRKRFDALKYDVKK 207

FRLLNLKNDSLRKRFDALKYDVKK

Sbjct 181 FRLLNLKNDSLRKRFDALKYDVKK 204

Graphical representation


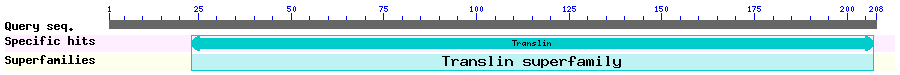


**Similar to translin associated factor X**

>Cp.comp28110_c0_seq1 len=1196

TACTGCTTCTCGCAGAGTCCGTTTACTGTGGTTATTCCATGTTATACACTATGGTTTATCTATGACACTATGCTTTGTTTTGTTTTTTATAGGTTGTTATCTTGGGTTGTGCTTGTGGTTTAATTTAAAATTTCATTTTTTGAGTGGTTATTTTCGGGTTGTTTATAATCAATAAACGTTTTGCTATAGTTTTTACGAATTTTCATATATAATCATGTCCAAATACAAAGGGCATAGCCGTCCAAAGAAAAATAAATTTACTGTTGGGAAGCAAGCGAAAAAACAATTGGAGTTGATCGACACAGATAATCCTGTTATTCAAATGTTTCAAGAATTTACTTTAGAACTAGATGATAAACATGATCGATACGAAAGAGTAGTCAAAATTAGCAGAGATATCACTATCGAGGCAAAAAGGATAATTTTTTTACTTCATAGTACTAACACAGACATAGAATCGAAGAGAGACTCTGTCTTAGAAGAGGCTTTTGAGAGACTTTCCACTTTGTACAAAAACAATTTCAAGAATATTGCAATGGAGCTAAGAGATAAAGACCATTATTTATACCATAAAGCATTTACATGTGGCATGCAAGAATTTATAGAAGCTTTATGTTTCTATCACTATACAAAAAATGGCAATATAACTTCATGGTTGGAATTAAATAAATTTTTCCAGTACAAAGATCAAGATGAAGAAGATCTTAATTTGTTATTCACACAGTATGATTTTATACTAGGCATAGCTGACTTTACTGGAGAACTGATGAGGAAATGCATCAATGTATTGAGTGTTGGAAATGTAAATGAATGCTTTAAACTTTGTAAATTCGTTAGGAATATTCATACTGGTTTTGTAGGATTATCATTTGCTGGTAATAAAGAACTTTCCAAAAAGGCCTACGTCTTACGTCAAAGTCTAGCGAAAATGGAATTGGTCTGCTACAATATTAAAATTAGAGGAAGTGAAATTCCAAATCATATGCTTCTTAGTGTTATAGAATCTAATGAGGTTGATAATGATATAGATGAAGGATTTGCTTTGTAAAATGCTTTTTAGGCATTTTAAAGTTTAATTTTTGTATATTTATTATTTGTGTTGTATAATTTTCTTGAAACCAAATTTAATACTTGTACAATGATCTATTTTTAACAGAATATGTTTAATAAAGACCTAAGGCTAAGAAGAAAAAAAA

Protein RF 2: 215->1048 (277AA)

Comparison with *Tribolium* PREDICTED: similar to translin associated factor x (548AA)

Query 17 VGKQAKKQLELIDTDNPVIQMFQEFTLELDDKHDRYERVVKISRDITIEAKRIIFLLHST 76

+G++ ++ LE ID +N VI+MF F ELD+KHDRYE++VK+SRDITIE KRIIFLLHST

Sbjct 6 IGEKGRQVLENIDENNRVIKMFLGFRKELDEKHDRYEKIVKLSRDITIENKRIIFLLHST 65

Query 77 NTDIESKRDSVLEEAFERLSTLYKNNFKNIAMELRDKDHYLYHKAFTCGMQEFIEALCFY 136

NTDIE KR++VL+EA +RL + NFK IA L+D D Y Y KA+T G+QEFIEAL FY

Sbjct 66 NTDIEGKREAVLDEACKRLKVITDENFKTIASILKDFDSYQYQKAYTSGLQEFIEALVFY 125

Query 137 HYTKNGNITSWLELNKFFQYKDQDEEDLNLLFTQYDFILGIADFTGELMRKCINVLSVGN 196

+ + I SW +NKFFQY +QD E +LLF Q DFILGIADFTGELMR+CIN L VGN

Sbjct 126 QFLHSNKIESWESINKFFQY-EQDGEKFSLLFPQLDFILGIADFTGELMRRCINNLGVGN 184

Query 197 VNECFKLCKFVRNIHTGFVGLSFAGNKELSKKAYVLRQSLAKMELVCYNIKIRGSEIPNH 256

V++CFK C FV++I+TGF+G+ G KE+ +K YVL+QSLAKMELVCYNI+IRGSEIP H

Sbjct 185 VSDCFKTCNFVKDIYTGFLGIINPGAKEMGRKTYVLKQSLAKMELVCYNIQIRGSEIPKH 244

Query 257 MLLSVIESNEVDNDIDEGF 275

ML++VIES++++ + DEG+

Sbjct 245 MLVNVIESSDMNTEEDEGY 263

Graphical representation


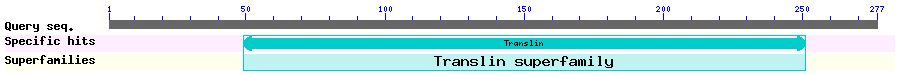


**Armitage**

>Cp.comp39999_c0_seq2 len=3613

GTGAGGTTAGGTTCTCACAATGTTGTTGAGGTTGTAATTTTTGTTTATAACAAGCACAATGTTAAGTTATGTGTTGTCCTTTTTTCGTCGTTCGTCCGAGTCCAACTTAACTCTGGAAGAATGTTGTAAAATTTTGGCAAGCGAAAATACTGAAGATGCTGGCCCCAAAAATGATACTTTAAGCCCTAGCAGCGATGATGACTTAAACGATCGAGATGGATACATATTGTCTACAAAAATAGGGAAAATAACAGGACAAAAAGGGGATAAATATGTAATTGACGACATTTATGAATTTGATATGGATTCTATCGAGTACCCCGTTGGCTCGGAAGTGTCGTATCAAATGATCGTAGACGACGGAAAAATAATAATATATAACGTAAATTTAATCTCAGACGACTGGAACGTGGCTCATACTAAGAAATCTTTGTGGAGTACACGTGTTATGGTTTGCAAAGTGGAAAAAAGAGATAAACGAATATTTTACTTGTCTCCTGGCAATATCAGAATCGATATTGACAAAATTGTCACAGAGTTCATACCTATAGTGGGAGACTGGCTCGAAGTGGATGTTAAACACGAAGTCAATGAATTTGCTTTAGACTTGAGCGGCACGGTGTTAAATATTAATAGAATATCTCCGTTAAGACCGCATATCGAAGAAGCAACGGTAAACTCCTGGAATAACACAACAGGTTCAGGAATTTTAAATCGGAATATATTTTTTAACAAAGATACATTGTCTTATGGTTACATTCCCGTAGTGGGAGACAAAGTAGTAGCGGAAATTATCGAAAGTGACCAGGAAAAGTGTACGTGGAGGGCTTTAAAAGTAATACCTCAGTATTTAAATAAAAAGTTTGATCGACATAACGATTTAATCGCGTATAAGGGTGATGAAATTCACCCTAGTGTTGAAATCGATAATGTTAATTTAAAATTTTTAAGATTAAATGAGTGCCAGCAATTTAATGTTATAATAAGGAACAAATCGAACGAGAAATTAGCTTTAACGGGTGCTGAATTCAGTAATCCGAATAGCCAGTGTAGAATAGTAAAACCCATTTCTGGGGAAGTTGATATTTTGCCACAAGAATCGTACGATTTAGAGTGTGAGTGCAAAGTTCGTAACATGGGTGCTTCGAATGAGTTTTTGTTGGTTTTGTTTAAAGATTTTAAGGTTAGTAAATGGATCGAGATAAATACCGACCCGGGTCAAGCAAAGAACGAGTTTTATCAGAACAAACAGCATAATTTTCAAAATAATTACAATTCTAATAATCAACTAATTCGTGGTCAAAGAAATAGCACTGTAAGATTTAAATCTGTTAAAATACCCGATTATCCAGTACCTAAGAAACTACTGGACCTAATTGTGAAATATCACAATCAAATTGATAAAATTAACCTCATTGAGGAACTCAAAGTAACAAAACGATCGCTTTTTAATAACTTAACCTCGACGAACTACGAAGACAAATTTCATACTCTGTTACACTTAGATGAAATCGCCAATTTAATTTGCATAAGAAATTACGACCAGGACTTGGCATGTTTTATAAGAAATGGCGAATTTTTGATGTTGGAAATAGAAAATTTATCAGAACGAAGGCCATCGATAGTTCTAGGCGACAGAATTATCGCAAGCGATCCTTTAAGAATCGCCAAAGAAGATTACGAAGGTAACGTCTTCAAAGTAGGCGCCCATCACGTTTATTTAAAATTTTCCGCTCTGTTCCACGACAAATACAACGGGGAAGACTATTCGATACGAGTTATACCCGGTCGTTCGACCTACAGGAGGCAACACCATGCGATTTACATGGTGGCCAGAAATTTGGGCCAGGATTGGCTGTTCCCTTCAAAAATTGTGGAAAGGGATGTGCAGGTGCGGTTTACGTATGATCACTATTCTAGAGTTATGCGGAATATTAAAAAGAGATTAAGGCCGAGAGAGTTATATCAAATAGCCGTGGAAGAAAACAGAAGAAAGAGTGAAGCAAATGCGACAGAAGTCGAAGAAACTGGTTCTATTGACGATTCGAGTCTTTTAAAACTGCAATGGTTCAATCAACAATTAAACTGTAAACAAAAGGACGCCGTCGTCAACATCTTAAAAGGAGTGGCGAGGCCGTTGCCTTATATAATTTTTGGACCTCCCGGTACTGGAAAAACCGTAACTGTAATAGAAGCTATACTGCAAATAATAAGACTCATACCGGAATCCCGACTTTTGGTCACAGCTCCTTCGAATAGCGCCGCCGATTTAATAGCTCTCCGATTGATAGATTCGGGAGTACTCAAGCCCGGAAATTTGGTTAGACTGGTGTCCGTCAATTATGCCGTTGGTGACAACATACCCGCCAGATTGGTACCTTACTGTGCGACTGCGGGTTTTGCTAAGGAGGGTACTGCCGAGGCAAATAATGTGTTGGAAAATGGCATGATTTGTGATTGCAGTAGATCTGTCTTGGGTAGGCACAAAATAACAGTCTCGACATGTTCTTCTGCCGGTTCTCTTTATCAGATGGGTTTTCCCAGAGGACATTTTACTCACATCATAGTAGACGAGGCCGGTCAAGCGGCCGAACCTGAAGTCATGATACCGATTTCGTTCTTGGACAAGTGGAACGGTCAGATCATATTAGCAGGGGATCCGATGCAGTTAGGTCCAGTTGTTATATCCAGAATTGCGGAAGAATGCGGACTGAATGAATCTTTTCTTGAAAGATTGACAAATAGATTTCCTTATATAAGGGACGCCGAAGGTTTTCCAGATACCGAGGGTTTCGATCCGAGACTGGTTACGAAACTACTGTACAATTACCGATCGTTGGATGTTATTTTGGAACTGTTTAGCTCAATGTTTTATCATGGGGATCTTATACCTACAATATCAGATAGGGACAGCAAAGAGGCAATGTTTCTGACATCCTTAAGTGAAATCTTGCCAAAAAGAGATGACGGTTCAGTGCCTTCTATTGTTTTTCATGGTGTTAAGGGTGAAAATTATCAAACAAATGATTCGCCATCTTGGTATAACCCGCACGAAGTCGCGCAAGTCTTTTATTATGTTAACGAACTATATAGATTAGGTTGTGAACCGACAAACTTAGGAATCATTACACCATACAGTAAACAGGTGAAAGAGATTAAATCAATCCTGAGAGAAGCAGAATTCGATCTCCCCAAAATAGGCACCGTTGAAGACTTCCAAGGTCAGGAATTTGGCGTTATTATACTGTCTACCGTTCGTTCGTCTCGTGATTATATACCGTCCGATTTGGAGCATAGTTTAGGGTTCGTATCGAGCCCTAGAAGACTGAACGTTGCCATATCTAGGTCTAAGTCTTTACTTATTATCATTGGGAACCCGAATCTGCTTTGTTATGACACCTATTGGAGAACTGTGATAACATATTGCATGGAGAAGGGTGCCTATACCGGTTGTGATTTAAATATGACTTAAAGTTTTCATTGTGATAAAATAATGATGTTTTTGTCATACAATTAGTTATTTTTATATTTTTTCGTATAACAAATACCAAATGTTAGAAAGGCAAAAGAGGATTCACTGTTTTTTTTTAAATAAAAATTTTGAGAAAGCAAA

Protein RF 2: 59->3472 (1137AA)

Comparison with *Tribolium* PREDICTED: similar to armitage CG11513-PA (1150AA)

Query 4 YVLSFFRRSS---ESNLTLEECCKILASENTEDAGPKNDTLSPSSDDDLNDRDG-YILST 59

YVLSFF ++S E+ L L+E ++A E+ P +P ++ + + G Y +

Sbjct 23 YVLSFFWKTSAPTEAPLPLDEAYNVIA--ECEEKLPNQ---TPQNEKEKEEEIGSYEVFR 77

Query 60 KIGKITGQKGDKYVIDDIYEFDMDSIEYPVGSEVSYQMIVD-DGKIIIYNVNLISDDWNV 118

K+GKIT D + IDD+Y+F D VGS++SYQ+I +G+I I +V+++ +DWNV

Sbjct 78 KVGKITQIIDDAFRIDDLYDFKSDIDGLLVGSKISYQLIKSAEGEIKIVDVHVLENDWNV 137

Query 119 AHTKKSLWSTRVMVCKVEKRDKRIFYLSPGNIRIDIDKIVTEFIPIVGDWLEVDVKHEVN 178

T K W R+++CKV KRD R+ LSPG+I ++++ I TEFIPI GDW+E+DVK E++

Sbjct 138 VDTGKKSWHERILICKVVKRDSRVLTLSPGDIIVNLNSICTEFIPIEGDWVELDVKCEID 197

Query 179 EFALDLSGTVLNINRISPLRPHIEEATVNSWNNTTGSGILNRNIFFNKDTLSYGYIPVVG 238

E +DL+G ++ IN++ PLRP ++ V W++ SG++++NIFF+KD+L GYIPV G

Sbjct 198 ENVIDLNGKIIEINKLLPLRPQVKLGHVTQWDSKNRSGVVDKNIFFDKDSLYCGYIPVRG 257

Query 239 DKVVAEIIESDQEKCTWRALKVIPQYLNKKFDRHNDLIAYKGD---EIHPSVEI-DNVNL 294

DKV+ EIIES+Q C+WRALKV+P +L+ F + D + K + + H SV I DN++L

Sbjct 258 DKVIVEIIESNQAACSWRALKVLPDHLH--FKENKDTLITKEETCYDTHDSVSITDNISL 315

Query 295 KFLRLNECQQFNVIIRNKSNEKLALTGAEFSNPNSQCRIVKPISGEVDILPQESYDLECE 354

F +L E + F + I +S E++ L E N QCR+ I + P + E

Sbjct 316 TFTKLGESKLFAMEI--ESREEIQLVCIEIPRVNGQCRLSTTID-HYTLKPARPLTIRVE 372

Query 355 CKVRNMGASNEFLLVLFKDFKVSKWIEINTDPGQAKNEFYQNKQHNFQNNYNSNNQLIRG 414

CK +N+G S E L+ F+ F++ +WI IN + KN ++K + +LI+G

Sbjct 373 CKAKNIGESQELLIFHFETFRIKRWISINVASYRPKN-IRESKDVRKEVVSRDKKELIKG 431

Query 415 QR-NSTVRFKSVKIPDYPVPKKLLDLIVKYHNQIDKINLIEELKVTKRSLFNNLTSTNYE 473

Q+ S RF + +PD+ VP++L +LI NQ + EL+ K L L N+E

Sbjct 432 QKIRSATRFLPLHMPDFAVPERLFNLITANRNQTSYLK--SELERMKPCLATTLNYMNFE 489

Query 474 DKFHTLLHLDEIANLICIRNYDQDLACFIRNGEFLMLEIENLSERRPSIVLGDRIIASDP 533

DKFHTLLHLDEI L+ ++NYDQ+ CF+++GE+L+L+I+NL+ERRPS+++GDR+IA

Sbjct 490 DKFHTLLHLDEIHALLEMQNYDQERVCFVKSGEYLLLQIDNLAERRPSVIMGDRVIARSI 549

Query 534 LRIAKEDYEGNVFKVGAHHVYLKFSALFHDKYNGEDYSIRVIPGRSTYRRQHHAIYMVAR 593

D+EG + K+G +HV++KFS +FHD Y+GEDYSI + R TYRR+H A+ + R

Sbjct 550 KDPNSPDFEGYIHKIGKNHVFIKFSQMFHDSYDGEDYSIYIKASRVTYRRKHFAVNLAVR 609

Query 594 NLGQDWLFPSKIVERDVQVRFTYDHYSRVMRNIKKRLRPRELYQIAVEENRRKSEANATE 653

NLG+DWLFP+KI+E++ Q+ F++D+Y V + R K E

Sbjct 610 NLGKDWLFPTKIIEKEPQLDFSFDNY--VDSLNNESNSSDSSDSKPCHNVRSKKEL---- 663

Query 654 VEETGSIDDSS-----LLKLQWFNQQLNCKQKDAVVNILKGVARPLPYIIFGPPGTGKTV 708

+E+ ++S+ KL+W N+ LN QK+AV NIL GV RPLPYIIFGPPGTGKTV

Sbjct 664 LEKIRKFNESTEKPLPTRKLEWHNKFLNYYQKEAVRNILLGVCRPLPYIIFGPPGTGKTV 723

Query 709 TVIEAILQIIRLIPESRLLVTAPSNSAADLIALRLIDSGVLKPGNLVRLVSVNYAVGDNI 768

TV+E +LQI+RL+P SR+L+ APSNSAADL+ALRLIDSGVLKPG+L+R+V++ A+G +I

Sbjct 724 TVVETVLQILRLMPHSRILLCAPSNSAADLLALRLIDSGVLKPGDLIRMVAIT-AIG-SI 781

Query 769 PARLVPYCATAGFAKEGTAEANNVL-ENGMICDCSRSVLGRHKITVSTCSSAGSLYQMGF 827

P RL P+ ATA KEGT + V+ NG++ CS +VLGRH++T+ TCSSAG LY MGF

Sbjct 782 PPRLAPFTATANTEKEGTETSLPVVGPNGLVLGCSSTVLGRHRLTICTCSSAGLLYSMGF 841

Query 828 PRGHFTHIIVDEAGQAAEPEVMIPISFLDKWNGQIILAGDPMQLGPVVISRIAEECGLNE 887

+GHF+H+IVDEAGQ +EP V+IP++FLD GQ ILAGDPMQLGPV++S IA E GL E

Sbjct 842 SKGHFSHVIVDEAGQTSEPSVLIPLAFLDVSTGQAILAGDPMQLGPVILSHIASEYGLEE 901

Query 888 SFLERLTNRFPYIRDAEGFPDTEGFDPRLVTKLLYNYRSLDVILELFSSMFYHGDLIPTI 947

SFLER+ +RFPY++D+ GFP T G+DPR++TKL+YNYRSL IL+L S +FY+ DLIPTI

Sbjct 902 SFLERMISRFPYMKDSHGFPKTFGYDPRMITKLIYNYRSLPNILKLPSLLFYNDDLIPTI 961

Query 948 SDRDSKEAMFLTSLSEILPKRDDGSVPSIVFHGVKGENYQTNDSPSWYNPHEVAQVFYYV 1007

S+ DS EA LT L E+LP+ G S++FHGV GEN+QT DSPSW+NP+E +QVF+Y+

Sbjct 962 SEVDSNEASLLTQLEELLPRDRYGKTASLIFHGVIGENFQTEDSPSWFNPNEASQVFFYI 1021

Query 1008 NELYRLGCEPTNLGIITPYSKQVKEIKSILREAEFDLPKIGTVEDFQGQEFGVIILSTVR 1067

NEL+RLG + +++GIITPY Q+K++ + +EA+FD PK+GTVE+FQGQEF VIILSTVR

Sbjct 1022 NELFRLGVKSSDIGIITPYKAQIKQLLELFKEADFDFPKVGTVEEFQGQEFNVIILSTVR 1081

Query 1068 SSRDYIPSDLEHSLGFVSSPRRLNVAISRSKSLLIIIGNPNLLCYDTYWRTVITYCMEKG 1127

S++ ++ SDL++++GFV+SPRRLNVAI+R+K+LLIIIGNP LL D WR+VI YC+E+G

Sbjct 1082 SNKKHVASDLKYTMGFVASPRRLNVAITRAKALLIIIGNPKLLSMDRSWRSVINYCIERG 1141

Query 1128 AYTGC 1132

+Y GC

Sbjct 1142 SYIGC 1146

Graphical representation


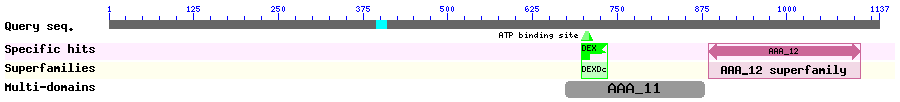


**Homeless (spindle-E)**

>Cp.comp40635_c0_seq2 len=4536

GCTTCATCACAATAATAAAAAAATATATCATCCGCCGAAACTTTAAATGGAACAAAAAAAAATTGAGATTCAGTCGTCGTCTTCGCAATCCGACTCATCCAATTGTTCCCGGTACTTGGCCTCTTCGTCTTTGTGCTGCTGCGACACCAAATGGAACCTCAACTCGTGAATAAACTCCAACTCTTTGTCGCACAACATGCAATTAATCCGTTCGAACGGTACGACCTCAAAGCTCATATCCTCCATCATTTCCAAATTCGTGGCGATTTTCCTGAGACGTGGATTATAACCGGCCAATTTAACGAACCACAAAAGACTCCAAATGTCCTGTTCGTCGTTGTCCATGCTCGGTTTGAGCAGGCCCAGACCGGCGGCGTGTTTGCCCCACGTCGTGTTTTGGGGCACGACGTTGCGTCTTTCTATCGGATTCCTCTCCATTCTGATTAAATCGAATAAGTTTTCTTTTATTTTGATTTGGGTGTGTATCAGTTCGTTTTGGTTCGACATGTTCTGTTCTATGTCGTGTAACAATTTGATTCCGTTGTTCATGAGATATCTTAAACGGTTTACCATTTCCAACTCTTCAATCTTCAATTCCGTATCCAAACCCAGGCAAATGTCGTGGGCGGGGTAAAGGGCCTTTCCGGTGATCTCGTTGTACCCCAACCCGCACAAAATGGCCGCCACCAGCATATTATCGCTGGTAAGTTTCGGTTCCATCGTCGGACAAAACATGGCGGTCATAAGCATCGGGAAACCGGGCAAGCTGGGCATCAACGTGGTCTGTCTAAGCTTCAATTTCATACCGTCTACCGATTGGCCAACGTGGACGGCCACTACCAACCTCGAATGGGGACTTTCCGGTTCGTTATCCAACAAAACGGCATTAACGGAGGTGCCTTCGACGAATACGCTTTTGGTACTGCCCATGTTGGTACAACTGTACAGTTTAACCTCCAAAGGGCTGAATGGACCTTTGAGGTAGATGGTCGAACACTTTGAATGGTTCAATTGGGGCGTTACGAAGTCGGAGTAGCTTATTATTTTGTCGTAATTAAGTCTGCTCGCTTCCTGGTCCGGGTTTTCGGCCATTTGTACCGTTTGTCTTTGCTCGTGGTTTTGCTTTGACAAAAAACTGGGAGTACTAGGCTCGGCAAATTGTCTGTTTAACAAATACTGGTTCAACGATAGATCTCTATTTTGGCTCCGATACAGAATGAGTTCGACGACGTCGCCGACCACCGAATAGACCTCTCCGTACAGTAAAATTCCGTCGGTTAATCGTTTAAAGTCCTCGTTCACTTTGCCGTCCCACATCCCAACCGGGTTCAGGCGATAGGTCGGTTTTACGCCGTGCAGGATGCACTCGAAAGCCAAAGGGGCCATCACGCATTGGGGTCTTTGCGGAACGTAGTATAGTTCGTCCGATCTGACCTCTTGCAAGTTGCCATAGTCGATGAAGAGAATCTGATTAAGTTGTCCGCCCAGGACAGTAACCTTGCACCGGTAAAACTGACCGTCCTCCGCGTACCGGGCGGCATAAATCTTATCGGTTTTGATGTTTTCCGTCACCTGTTTCAACACTTGCTTGTTGAGAGCCTCTTCGATCTGCTGCAGGAACACGTTCGTTTCTTCGGTAGCGTTTTGTGCCCAAAAGTGACCCGCATCTATATGATTTGAAATATTTAACGAAATATAAGTCGTGTCCATCGACGGTAAAGGTGAGTATTCCACCGAAGAGTAACAATTCCGCTCTTGGTAGGGCAACTTGCCCGAAGGCATGTTCCGTTTTAAGCCGTGCTTTTCCGCAAACTCCCAAGCCTCGTTGTACGGCAACACTTTTAATTGAAACGGATACTGCAATTGTCGTTTCCTCAGGATTTCGTACATGGACATCGGTATCCTGCCCGGTATATTCGTCAATTTTTGAAAACCGTCCACGTTCACCGTAATCGGTTCTCTTTGGAGCACATTTTTGAACTCTACGTATATTTTAGAGCTACCGTCGAATCCCACTTGTACGTCCATTTCTCGTTCGCCGTTTTCCCTTATTAATTTTTTTATTGGTCTTACGTAGACCTGTCCCGGTTGTCGCGGGTCCATTCCGGTAAAATAAACGGTCGAAAATGGATCGCGACCGCCTACGATTTTAACCGCCTCTCGTTCCGTCATTTGGCTCGAATGGGGGGACTTTATGAAGAAATTGGGGTAAAATGCTCCGGCCATTATTATCTTCAAAACTGTGGGTTTTTCGATATCGCTCAGAATGACCCGGCCCGGTCCGTCGGTCTCCTTAATCTGCATCCTTTCCAAACGCTGTCTAATTTCCGTTATGAGCAAATTCCACTCCTTCAGGCCTTTTAAACTGACCAAATTGGTACGGCACCATTCCAACTCTTGCCTGTTCGATCCGAACGCATTTTCCCGCTTCAGATTCAACCACACCGTGTACAAATTCAAAAGGGCTATTAAGTCGCTGCAAGAACCGTCCGACCACAGCAGCTGCTTCCTATAAGCGTCGAGTCTCTTCTGGAACGGTATGGAAAAAATGTTCTGGATGGAACAACCGGCGGCGATAATGATGGTCTCTTCGAGACAACTAAACAGTTGCCCTAAAACGATGAGTTTCGATAGGTGTACGTCTATCGGCAAATTGGCCATAACCTGACCCAAAAAGGTTATGTCGCCGTCGGCGTTCGCCTGGATACCCCGGCAAGTTCGTAACATGCCTCCGATCTCTTTCAATTGCCAAATCGTGTCCTCGATGTTCTTCAAACCCGGAGGGGTCATGGCCAGGGCCAAGATTTGGGCCGGAGTTTCGTTCAGCTCGAGCATTTTCGCTTGCAAAACGATCCTTTCCAGCGGCGCGCTTAATATCTCCGGCTTGCTGTGTTTCTCCATTTCGGACATGTAAAATTGTCGATTAACAAGCCGATAGACCCTGCCGTTGCACGTCCGGCCCACCCTGCCGGCCCTTTGGTCGCAATTCACGTGGGAGGCCCACTCGAGCTTGAGGCTCATGTATTTGGTCTCGGGATGGACCGTCATCACTTTGGTTTGGCAAAAATCGATCACGAACGATGAGTCCGGCACCGTAACGGAACTCTCCGCGATATTGGTCGACAAAATGATTTTTCTTAAGCCCGGTTTGCGGGGTGCAAAGGCCCTTCCTTGTTCGTCATTCGGAAGGGACGAATGTAATGGTATGATCTCCCATTTGTTGGTATTTGTCTCTTCGTTCATTAACCTGCGGTGGGCCTCTTCGATTTCGTAGATACCGGGCAGGAACACCAAAACGGTCCCGTTTTTGTTGACGGTGGCTTCGCCCTCTTCGAGTCGATCGAAGATGCTGATGAGGAAAACAAATACGGCCCAAACCTCTTCCGATATTTCGGGTTTATAGATGTCGAACTTGGGCGTCTTCTGAGTAATTTTGTTGATCATGTCCAAATAAAATATAGTTTTGGTGTACTGGCTGACTTTGTCGATGGATATTATCGGCGCCGAAATGGACTGTCCGAACGTCTGCCTTCGGAAATAATAAGCGAACTCGTTGGCTTTTATCGTCGCCGACATCAAGATAACCTTCGTCTGGGGCGAATTGGTGAACAAAAACTTACGAACAAGCAAAAGCAAAAAATCCAATTCTTGGTTACGTTCGTGAACTTCGTCGATGATTATATGAGTGTACGCCCTTAACGATTTGGCTCGTATCAACTTCTGAAGGAGGACGCCTATGGTCATGTAGGTTATAATCACGTCCGGCGACAGTTTCTTTTCTAAACCCACCTGGTATCCGCATATGGTGCCCAACGGCCAGCCCCGTTCCTCGCACACCCTTTTCGCCACATTTATGGTGGCGATTTTCCTCGGTTGCGTCACCACTATGTTACAATATTCGTTTTTCAGCCGGAAACTGTCCATAATCATCTGCGGCACTTGGGTAGTCTTACCGCAACCCGTCGGACCTTCGATAACTATAACCTGATTAAGATCGATCCTAGACAAGATTTGTTGCTTGTAGGAGTCGATCGGTAGTTCTTTTTTAATATCCGTGCTCAATTTGAAATTGTACGCCTGATAAATGTCGCCCGGTAGGCTCTTTCTGTCACCGACGATACTGTCGATGTCCGAAAGGCCACCGTAGTTGCTCACATCGTCGGCGAAGCCTTTGGATATCCCGGCCGCAGTAAGTTCCGAATATCTCTTCATTTCTTGCTGGACGAACTCTTGTTCGTAGGGTTTATCGGGACCGTTGTCGTCGTCGCTGTCGGACGTATCAAAATCGTCGTCGTCAATGTTTGGGGCTAAAATCGCCCCGTTCACTCGCCCCACGGGGTAAGTGAGATTCTTGCCCACCTTTTGGAGTAGCTGATTCATGGCTACTAGGATTTAATGGGAACGTCGATATGCACCGAGTGGTTTGTCGTTATATCACTCTTGTGCTTGGAAAAATCATCAGTGATTGTTTAAACAATTTCAACAATTCCTTTTCGACAAAGTTGGAGTAGATATAAATATTTGGTTAGTTTCCTG

Protein RF -1: -4380->-70 (1436AA)

Comparison with *Tribolium* PREDICTED: similar to ATP-dependent RNA helicase A/ spindle E (1431AA)

Query 49 EQEFVQQEMKRYSELTAAGISKGFADDVSNYGGLSDIDSIVGDRKSLPG-DIYQAYN--- 104

+QE++++E+ Y T A S GGL+DI+ G +S+ G DI + +

Sbjct 49 QQEYIKKELSTYFPETEAPCS----------GGLTDIED--GHEESVLGTDIMELLHVPK 96

Query 105 ----FKLSTDIKKELPIDSYKQQILSRIDLNQVIVIEGPTGCGKTTQVPQMIMDSFRLKN 160

+K T KKELPIDS + +IL I+ N V++I GPTGCGKTTQVPQ I+D R

Sbjct 97 VFETYKFDTYYKKELPIDSSRDKILDMINTNSVVIIHGPTGCGKTTQVPQYILDHCRATK 156

Query 161 EYCNIVVTQPRKIATINVAKRVCEERGWPLGTICGYQVGLEKKLSPDVIITYMTIGVLLQ 220

CNIVVTQPR+IA IN+A+RVCEERGW +GT+CGYQVGL+K + DVI+TYMT VLLQ

Sbjct 157 SPCNIVVTQPRRIAAINIAQRVCEERGWAIGTVCGYQVGLDKNVGDDVILTYMTTEVLLQ 216

Query 221 KLIRAKSLRAYTHIIIDEVHERNQELDFLLLLVRKFLFTNSPQTKVILMSATIKANEFAY 280

KLI K+L +TH+IIDEVHER++ LDFLLL+VRK+LFTNS K+ILMSAT++A +FAY

Sbjct 217 KLISQKNLNRFTHVIIDEVHERSKSLDFLLLIVRKYLFTNSSSVKIILMSATMEAQDFAY 276

Query 281 YFR---RQTFGQSISAPIISIDKVSQYTKTIFYLDMINKITQKTPKFDIYKPEISEEVWA 337

YFR R Q + AP++ + K S+Y +I+Y + P ++ +P + +E +

Sbjct 277 YFRSISRNNPQQYLLAPVLPVTKKSEYKVSIYYNE---HFASAMPPYNFEEPCMHKEQYD 333

Query 338 VFVFLISIFDRLEEGEATVNK----NGTVLVFLPGIYEIEEAHRRLMNEE-TNTNKWEII 392

V LIS+FD+LEE E+ + NG+VLVFLPG +EIEE H+ L+ E T+ +WEII

Sbjct 334 VCAKLISLFDKLEENESKLTLAERINGSVLVFLPGFHEIEEMHKVLVRERNTSVLEWEII 393

Query 393 PLHSSLPNDEQGRAFAPRKPGLRKIILSTNIAESSVTVPDSSFVIDFCQTKVMTVHPETK 452

PLHSSL + +AF + RKIILSTNIAESSVTVPD +FVIDFC TK MTV+ TK

Sbjct 394 PLHSSLAQEHMVKAFQKPRQRCRKIILSTNIAESSVTVPDVNFVIDFCLTKNMTVNEVTK 453

Query 453 YMSLKLEWASHVNCDQRAGRVGRTCNGRVYRLVNRQFYMSEMEKHSKPEILSAPLERIVL 512

+ SL L+WAS+ NC QRAGRVGR NGRVYR+V FY+ EM++ + PE+ APLE ++L

Sbjct 454 FSSLSLQWASYTNCIQRAGRVGRVANGRVYRVVPTSFYLHEMKQTTVPELQRAPLENVIL 513

Query 513 QAKMLELNETPAQILALAMTPPGLKNIEDTIWQLKEIGGMLRTCRGIQANADGDITFLGQ 572

K+L LN+TP +L+LA++PP LK++E +W LKE+G +L+TCRG + ADGDITF+G+

Sbjct 514 YMKLLGLNDTPKNVLSLALSPPNLKDVEQCVWHLKEVGALLQTCRGHRTPADGDITFMGR 573

Query 573 VMANLPIDVHLSKLIVLGQLFSCLEETIIIAAGCSIQNIFSIPFQKRLDAYRKQLLWSDG 632

VM +LPID+HLSKLI+LG +FSCL+E +I+AAGC +NIF F R YR++L+W+DG

Sbjct 574 VMGSLPIDIHLSKLILLGHMFSCLDEAVIMAAGCMTKNIFVQNFYDRFRTYRQKLVWADG 633

Query 633 SCSDLIALLNLYTVWLNLKRENAFGSNRQELEWCRTNLVSLKGLKEWNLLITEIRQRLER 692

S SD + LLNLY VWL++KR+ AF S+ QE+ WC+T+ V+LKGL+EW++LI EI RL+R

Sbjct 634 SHSDFMILLNLYNVWLSMKRDRAFSSSHQEIGWCKTHFVNLKGLREWDILIQEIHSRLKR 693

Query 693 MQIKETDGPGRVILSDIEKPTVLKIIMAGAFYPNFFIKSPHSSQMTEREAVKIVGGRDPF 752

+ I++ GP + LS +EKP VLK+I+ GAFYP +FIKS + +EAVKI+ GRDP

Sbjct 694 LNIQKLPGPSSIPLSIVEKPMVLKVIICGAFYPYYFIKSSDFGNVDAKEAVKILNGRDPC 753

Query 753 STVYFTGMDPRQPGQVYVRPIKKLIRENGEREMDVQVGFD-GSSKIYVEFKNVLQREPIT 811

+TVYFT M QPGQ+YVR IKKL+ N E + +VQ+GFD S+K++VEFK R+P

Sbjct 754 NTVYFTNMKMNQPGQIYVRQIKKLM--NCEDKPNVQIGFDPQSTKVFVEFKAT--RQPEQ 809

Query 812 VNVDGFQKLTNIPGRIPMSMYEILRKRQLQYPFQLKVLPYNEAWEFAEKHGLKRNMPSGK 871

V +DG Q + + I + +YE +RKRQ++ PF L++LP ++AWEFA KR +

Sbjct 810 VTIDGRQYIATVASNIAVDVYEAVRKRQMRVPFVLRILPDSKAWEFANMTQAKRQIAES- 868

Query 872 LPYQERNCYSSVEYSPLPSMDTTYISLNISNHIDAGHFWAQNATEETNVFLQQIEEALNK 931

++ NC+++++YSPLP++D YI++ +++ IDAGHF+ QN EET + L QI ALN

Sbjct 869 ---EDVNCFTTLDYSPLPTLDIEYITVTVTHIIDAGHFYCQNWNEETRMLLDQIFAALNG 925

Query 932 Q--VLKQVTENIKTDK-IYAARYAEDGQFYRCKVTVLG-GQLN--QILFIDYGNLQEVRS 985

L+ E IK + IYAA + EDG+FYRCKV L GQ N Q+ FIDYGN+Q V

Sbjct 926 PGVFLEPAGEKIKVNSDIYAALFNEDGKFYRCKVIDLTPGQPNVAQVCFIDYGNVQRVPK 985

Query 986 DELYYVPQRPQ-CVMAPLAFECILHGVKPTYRLNPVGMWDGKVNEDFKRLTDGILLYGEV 1044

+ LY +P+ + C + P+A C+L GV+P LNP +W VN ++ T G+LL +V

Sbjct 986 NRLYKLPENSEPCRVQPIAMCCVLSGVQPDLVLNPKALWSESVNNILRKKTTGVLLNAKV 1045

Query 1045 YSVVGDVV--ELILYRSQNRDLSLNQYLLNRQFAEPSTPSFLSKQNHEQRQTVQMAENPD 1102

+SVV +VV EL L +S NQ+L+N + S SK +HE R VQ +E+P

Sbjct 1046 FSVVDEVVHLELFLQNPGRNSVSFNQWLINEGLGQKCEESQRSKMDHEMRLKVQSSEDPS 1105

Query 1103 QEASRLNYDKII-SYSDFVTPQLNHSKCSTIYLKGPFSPLEVKLYSCTNMGSTKSVFVEG 1161

++ N ++I+ SY+DF P+ + + I LKGPFSPLE+K+ V V+G

Sbjct 1106 NMSALFNKNQIVTSYADFEAPESSEAT-EIIELKGPFSPLEMKVCGLVQASQGAPVHVDG 1164

Query 1162 TSVNAVLLDNEPESPHSRLVVAVHVGQSVDGMKLKLRQTTLMPSLPGFPMLMTAMFCPTM 1221

SVNAV+LD+ PE H+ L+VA V QS +K+ QTT+MP++PGFPMLM +FCP M

Sbjct 1165 DSVNAVMLDDNPEDYHATLLVAGQVSQSSTTSAVKISQTTIMPNIPGFPMLMCLLFCPQM 1224

Query 1222 EPKLTSDNMLVAAILCGLGYNEITGKALYPAHDICLGLDTELKIEELEMVNRLRYLMNNG 1281

EPKLT D VA+ILCGLGY E+T +A +P HDICL LDT+L+ E + +N LRY MN

Sbjct 1225 EPKLTPDGSRVASILCGLGYKEVTQRANFPMHDICLVLDTDLRSEIITKINALRYYMNEA 1284

Query 1282 IKLLHDIEQNMSNQNELIHTQIKIKENLFDLIRMERNPIERRNVVPQNTTWGKHAAGLGL 1341

IK++ I++ ++ E+ TQ +K+ LF L+ M + ++R NV + W K + +

Sbjct 1285 IKIMSQIQEELARPEEMYTTQRFLKDELFHLLHMRQQTVDRVNVRYPD-VWNKGLDNMEI 1343

Query 1342 LKPSMDNDEQDIWSLLWFVKLAGYNPRLRKIATNLEMMEDMSFEVVPFERINCMLCDKE- 1400

L+ MD+DE+ IWS LWFVK I NL+ + ++ P I C LC+

Sbjct 1344 LRIDMDDDEEAIWSYLWFVKFGEDRLSKMSINKNLDELTQIARRTAPEREIKCELCNSAT 1403

Query 1401 LEFIHELRFHLVSQQHKDEEAKYREQ 1426

L I ++R HL ++HK AKY Q

Sbjct 1404 LRNISDVRIHLFREEHKSNLAKYLNQ 1429

Graphical representation


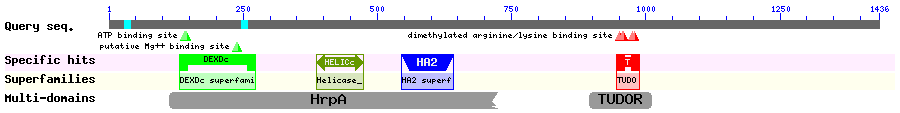


**Maelstrom**

>Cp.comp35977_c0_seq3 len=2283

AATAGGCAAGTCTTCTTAGCAGAATGCACAAGCCTGTCAGACCTGTAAACATAATCGATAATAAGTTCATATTACCGGAGAGAACTTAGGATTAATTTTATGGCGACTCTCTGTTTCTATTTCTTCGGGTAATCGTAACAACCTAGCTAATCTGAGTAAATCTCTACGTTCAAAGCGTAAGTTTAGTTTTATTTCGTCGTCTGATAGTTGATTTAAATCGAAATTTAAATCAGGATCTATTATGTGATCTCTGTCGATCAAAACATTGTCCAGTATAGCATATTCTATATCATCGTCATTGCCGCCTCCAAGAATAATCGCTTCCAAATAATCTTCCATTACAAAGAAAATAATATAACTGTAATTCTTTTTAATTGAAAACTGAAACTAGACCAAAACTAAACACCACAATAAAAAACCAAAAACAACAAATAGAGGTTATGTTTTAAAATAAGTTTGTAACTATGGGTCATTGCGATAGCATAAATATTTGCCTCCAATAATGCCATCCATTATTTTAGCTAGGGCGCTTTAATTAACGGTTGTCAGTAATAAAGTAAAGTTGCGATAACTCTCTAATTTGTTAAAGCGAAGCCACCGAGCCAAATAATCGAATATGGCACCGAAAAAGCCGGCAAAGCCCAATGGATTCTTTATGTTCGCAAGTGAATTCAAGAATCGTCATGGCAGGAAATATGCGAGTATGAAGGAGGCATGCGATGCGGCCGCTCCTTACTGGGCTAAAATGAACGCCCAGGAAAAACAGAAGTACCAGGACATGGCCAGAGAGGAAAAAGGAAGCGGCAAGAAATACACTTCCGAAGGCCTGGACGTTGAAGAATTGACCAGAAAGGAGAAAGAGCAGGAACAGTTCCAGGAAAAAATGAGGCAGGATATTAGACAGATCATTGAGCTGGCTTCGATGAAGGGATCGGAAGCATTAGCAAGCGAGACATTCTTCCTGATCCATTTCAATATTTTTTGCTTTCACCCACCCAGCGATCAGTATTATCCAGCCGAAGTGGCCGCTGTGTGTTTCAATTTGAAAGACGGCGTCAAACCTGAGAACGTTTTTCATGAGTTTGTGAAGCCAGGTCCTATGCCGCTGGGATATTCTTTCGATGCCAAGCAGCATTCCGAAGAGACTCATAAAATAAGTGTCCCATATGACGATGGAGAAAACAATATGGAAGAGGTTTTCGTTAAACTCACTACATTCTTAAAGAAAAAGAAACCGGCCAATGTCACTCGCCTGCCAATATTGTTCGCCAACGACAAATACCAAAAAATGTTCCAGAACATTCTGGACAGGTGGAGCTGGGAATACGGCGGTGAAGAGAACCTGTTTCGTGTCTACAGTCTTCAAGTGTTGTTATTCTGGTTGCGAAACAAGGTGTCGGGTGGGGAAGTGTGGCACACCGTCACCTTCAGCGACCGTGAAATTGAAAAGGACGTTTACGCTTACGTTCCCGACATAGCCTGTCATTATCACAGTAAAATGTCCAGCTCTCTCTACTGCAGCCAATCTAACGTCTTGCGAATGGCCTATGTGGTGTGCGACAATTGCTGTGAAGATCTGGATATCGAACTGTTGCCAGGGCAGCATGTGCCTGTCAGATCTTCGGTACCGATTGGGACATCCAGTGTGAAGAGTTTTAGTACCCGTACTAGCCGTAGCACTGAGCAGAGGGGTAATAAGTCTCATTGGGAACAAGAGAGTGATTTTGATGCCACTTCTATGACTGATTGGGAGAATAGATCTCTTATCTCCGAGACTTCGGCGACGACGACAGTAAATACTGATCGCAACTTTCCTCCTTTGGGACCTAGAATGGATAATAAACGTCAGTTTCCTAACTTACCACAGAGCAGTTCAACAGTGGGTGCGAGTTATTCTGACGCGTTCAAATCACTTCCCGCCGCCTTTGAAAATATGGTTATTAGATCTGCTAAAGGTAGGGGCATACCTAGGAAAGATGATGACACCACCAGCGTCACCTCCTCAGTGGTTAGCAAGGGTAGGGGGTATAGTGTCAGCAGTGGTGATGATTCGGCGCCAAAGGGGAGGGGTTTTCGGCGCGGCGCACCAAAGAGACCAGGACCAGCTAACTGAATTTTTTTATGATTTCACAAGAAGAACAATTTTTTATAAGAATATGGTTGTTTTCGTTTTTTTTAAACATATATTTACTGTTGACTAAATAACTGAGGTTATGACTTGTAAGTAAGTATATATGGAATTTATCTTGTTTCATTTAATGAATATATATTTTGTAATAAAAA

Protein RF 2: 617->2113 (498AA)

Comparison with *Tribolium* maelstrom (437AA)

Query 12 FFMFASEFKNRHGRKYASMKEACDAAAPYWAKMNAQEKQKYQDMA--REEKGSGKKYTSE 69

++ F + +N+H K +M E + AA WA M+ +E++ Y++ A E S +YT++

Sbjct 14 YWEFVLDCRNKHPNK-QNMHEVQEYAARKWASMSKEERRPYEERALLAREMYSPARYTTD 72

Query 70 GLDVEELTRKEKEQEQFQEKMRQDIRQIIELASMKGSEALASETFFLIHFNIFCFHPPSD 129

G+D+E + RKE+++ + +++M+ DI + ++ A + L + F +IH N ++P D

Sbjct 73 GIDIEVVERKERDEARKKQEMKDDITRTLKAAYF--ATDLDEKIFLVIHINHLAYYPTED 130

Query 130 QYYPAEVAAVCFNLKDGVKPENVFHEFVKPGPMPLGYSFDAKQHSEETHKISVPYDDG-- 187

+Y+ E+A +LK+GV E+VFH VKPG +PLGY A HS+ETH++ D

Sbjct 131 KYFICEIAIAAVSLKNGV--EDVFHRIVKPGKLPLGYYGGALTHSKETHQMLELVQDEPY 188

Query 188 ENNMEEVFVKLTTFLK--KKKPANVTRLPILFANDKYQKMFQNILDRWSWEYGGEENLFR 245

ENN EVF ++T+FLK + K ++ I++A++K +M ++D + E+ + + +

Sbjct 189 ENNTREVFNEMTSFLKLWRGKGSD----SIVYADEKTHEMITKVIDNFCQEFNYPDEI-K 243

Query 246 VYSLQVLLFWLRNKVSGGEVWHTVTFSDREIEKDVYAYVPDIACHYHSKMSSSLYCSQSN 305

VY+ Q L F LRN V+ VW T T+S E+EKD+Y+Y PDI+C +H S+YCS+S

Sbjct 244 VYNFQYLFFALRNSVAARTVWPTETYSSTELEKDLYSYTPDISCEFHEMSDISVYCSKSI 303

Query 306 VLRMAYVVCDNCCEDLDIELLPGQHVPVRSSVPIGTSSVKS 346

V R Y +CD+CC DL+I+L+ G HVP S + + +S S

Sbjct 304 VTRYCYTLCDHCCTDLNIQLVAGFHVPKNSRIAVDSSRTNS 344

Graphical representation


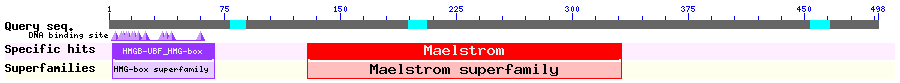


**HEN1**

>Cp.comp39152_c0_seq16 len=2745

TTTTTTTTTTTTGCTGCGATATCCGTCTTTTATTAATTGCCCGTAGAAAACTTTCAGTCTTAAGTGAATTTATGCAAATAAACGGTTACTTATCTTTGCTCCGGGAACTGTCGGAAGATACGTTTCTAGCGTCGATGCCCTCATCGGTACTATTGGCCAACGACTCTTCGGGTAGTGAGCTCGTGTCGTGATCCGAAGCACCCGGTTCAACTTCGGCAAAGTCCGCAGTATTATTAGAAGATTCGGTAGTTGTAGCGTCGTCTCCTTCGTCCGCGTCCAGAACGGACACCGACGGATGGACACCTAAACCGTCTCCCTGACAGTAAAAATGGGGCTCCACGTCTGTAGATATCGCGTGACCGCCTAAAATTTGCAAGAGCCAGCTCGGGAAACCGCTGATGGTGTTGGTTTGAATGGACGCTGCTGGGGGTTCCTGCGAACTAGATTGAGACGGTAGGTCGAAATCTTGCAATAGGAGGTCTTGGTCGGAATCGGGATCGAATAACGCTTCTCTGCTTGCTTGGGCCACATCGTCCAGCTCAATCCTTTGTTCCAATGGTTCTTCGACGATTACCACTATCGGAGCCTCGAAAGCGCCCAAAACATTATCATCATTGTTATTTCCTGCAACAGCCGCGTTGTCTGCCACTGGTGCTACCAATTCTTCGACGTTATCATTCAAAACGTCGATTCCTTCGTCCGGTTCCACAACGTCGGCCACAAAATTATTACCTTCATCGTCTCGATTGTTATTGGCCAGATCTCCGTTTTCGACTACCTGAGGCTCCGCTTGTTCATCTTGTCGTCGCTCCTCTACGTTCTGTATCAAATTGTCGTCTTCGAAATCGATTTTGTTTAAACTATTGTGAATGATACAATCGGCGATACTTTTGACGTCATCTAACGGACTATCGTCGCTCTTTATCTTGACTTTCTTCTTCTTGGGCGAACTGGACGGTCCGGCAACGGGAAAATCCTTTGGCGGAGATTTCTTGGGAAGTTTATCCAATTCGTTTACTCTATCAGCGTCAAATTTGATCCTATGTACCTCCGTTAACAGATGGGAAAGTACAGGATTGTGGGGCTTTTTGAGGCAATCAAAGTGGTCGGTTTCTTCCAAATCTGATTGTAAACTGTCGTCCTGAGGCGAAAGCGACTTCTGATAACCCGAGTCCAGCAAAGAATCGGAATGTGGTTTCTTGTCGTCCTTAACAGCCAACAACGGCGCACTTGCTTCCACTTCCTTAAAGCTTTCCCATTGGTCGTTGTCGGCGTCCCAATCGGACATCTCGTCCCGATCTCTCATGGTAGGTTCAGACTTGATGCTAGGGCTCGTATAACCTGAAGCTTCAGAAGTACTTTCGAAATCTTCCATTTGTTGCGGCTCCCAAATTACGCAATTTTCACTTAACCCGGTTTCCGGAATGATGCATTTTTCCACCGTGTAGCCCGATTTATTTAAAATGTTGCAAACTTCAGGCTCGGAAACGTTTATATTTCCATACAAGATATTAATCACCGGTATTTCACACCTGTCCCGTTCCTCCACATAAAACCGGCTGTTTATATAGCCGAAAGTGTGTATTCTATACCTTAAAGTGTCCAATAACTTTTCCTCGTCGCTCCTTTGGTCAATTTCGTACGGATAAACAATCTTTTCAACCATCTTGTAGTAGATGTTAAACGCAGGATTGTATTCTACATTCTTAATGTCACTAGCGTTAACCGAATGCGTTAAGTAAGTGCAAATACCGACGCTATGATCCGGCTGACATAATCGGCACACGCAACCGCACAATAATTTACCTTCCGCCACGGCACTTTTGCAAAAACTGTCCGATTTGCACAAGCACTGTCTGGTATAAGCACAGGAAACGTATTTTTGACAAATGAGAGTCTTGTGGATGAAAACGGCCATCTGCGAACACGCCCCTAGCTCTTCAGAGCCAGAAGGGCCCCACCCTATGGCTGAAAATTCGACTGTGTAATCGGGAAACCTGGTAACTATGTTTTCCGCCCAGTCTTCGAATTGTTCTCGAGTCCACTCGAATTTGTGGTCAAAATGTCTGAAACCACCGACCCTGTTGAACATTACGTTAAAATCCGCATTCGGGGTTGTTATAACAACGATTTTCGGCTCGATGAATGAAAACACATTGTAAGGGAACGCATCGAGGGTGTCCGGATAGAGGTGTTCGATTAGCTCTATGGCGGTGACTATGCTGGTGTCTTGTAACCTATAATCGGGATCTTTAACGCTGCCCGCAAACACTGACACATCCAGGGGATGGTTGCGACGCATTATCATATCGCACAATAAAGGCTCAATCCGAAACATTTTTTCTTTGAGCAGCTCTTCATCGATATCGATAAAAAACAACCGTTCCAGAGTTAAGTTCTTGAGGAATATAAACATGCCAAACTCGGCACAGCCGAAATCCACCAGCTTCTGGAGGTCTTTTCTCCATCTTTCGTCTATTAAAATTTGGTAAACTTTCTCGTATCTTTGCTTGAAAACTGGAGGATCGAATTTCAATTCGTATTCCTGTTCCCTTTCGACGGTATCGTTTTTCAGCCTGATTATGTTCCGAAACAGCCGATAAATCAACATGTTCAAGCAGTGGAACACGAATATCATTTTCACGAATTTTGTTTATTATATTTTACCGAAAGTTGACACTCTAATAGGTTAGGTACATTTCCTTCTTTTTGTGTTTTATATTTGACCCTCCTGCCAGATCGAGG

Protein RF -3: -2638->-86 (850AA)

Comparison with *Tribolium* hypothetical protein TcasGA2_TC004824 (856AA)

Query 27 VEREQEYELKFDPPVFKQRYEKVYQILIDERWRKDLQKLVDFGCAEFGMFIFLKN-LTLE 85

+R+ E ++KFDPPV+KQRYE+ IL+DE+W+ + K+VDFGCAEFG F+FLKN L+L

Sbjct 25 AQRDAENDIKFDPPVYKQRYERAVDILLDEKWKNQVNKVVDFGCAEFGFFVFLKNRLSLS 84

Query 86 RLFFIDIDEELLKEKMFRIEPLLCDMIMRRNHPLDVSVFAGSVKDPDYRLQDTSIVTAIE 145

L IDID+ LL + ++R+ PL D ++ R PL V+V+AGS+ +PD L +T V A+E

Sbjct 85 ELLLIDIDDLLLNDYLYRVYPLNADHLVGRPKPLTVNVYAGSIAEPDPSLLNTDAVIALE 144

Query 146 LIEHLYPDTLDAFPYNVFSFIEPKIVVITTPNADFNVMFNRVGGFRHFDHKFEWTREQFE 205

+IEHLYPDTLDA PYN+FS+I PK+V++TTPNA+FNV+F ++ FRH DHKFEWTREQF+

Sbjct 145 IIEHLYPDTLDALPYNIFSYIRPKLVIVTTPNAEFNVLFTKLQKFRHADHKFEWTREQFQ 204

Query 206 DWAENIVTRFPDYTVEFSAIGWGPSGSEE-LGACSQMAVFIHKTLICQKYVSCAYTRQCL 264

WA NI +RFP YTV+F +G GP G+++ +G CSQ+AVFI K +IC Y C

Sbjct 205 SWATNITSRFPSYTVQFDGVGLGPHGTDDSIGCCSQLAVFIRKDVICD-----TYEETCN 259

Query 265 CKSDSFCKSAVAEGKLLCGCVCRLCQPDHSVGICTYLTHSVNASDIKNVEYNPAFNIYYK 324

+DS CG YYK

Sbjct 260 VSNDS------------CG--------------------------------------YYK 269

Query 325 MVEKIVYPYEIDQRSDEEKLLDTLRYRIHTFGYINSRFYVEERDRCEIPVINILYGNIN- 383

++ I YPY++D R+++EK+LD L+YR+H F FY E +IP+ ++Y

Sbjct 270 LIASINYPYDVDTRTEDEKILDELKYRMHLFENSEEEFYNVETKCFQIPLNQLIYHITKP 329

Query 384 -VSEPEVCNILNKSGYTVEKCIIPETGLSENCVIWEPQQMEDFESTS--EASGYTSPSIK 440

EP++ IL K Y +E+C P T E+CVI+E +M+ S S EASGY S +

Sbjct 330 FPPEPDIRKILLKYNYKIEECRNPITKKLESCVIYE-AEMDSGGSGSDTEASGYGSDNKY 388

Query 441 SEPTMRDRDEMSDWDADNDQWESFKEVEASAPL----------LAVKDDKKPHSDSLLDS 490

+ D + SDWD ++ W S E + L V + K P +L DS

Sbjct 389 N----VDEGKFSDWDENDLTWTSSTSKENEPAVSSKGVKTQLHLEVNESKNPQ--ALFDS 442

Query 491 GYQKSLSPQDDSLQS-DLEETDHFDCLKKPHNPVLSHLLTEVHRIKFDA-DRVNELDKLP 548

GYQK SP DDS QS D F L N LSH+L+ FD D++NELD +

Sbjct 443 GYQK--SPPDDSPQSKDQAVALDFKSLNDRPNK-LSHILS-----VFDNFDKINELDAVR 494

Query 549 KKSPPKDF--------------------PVAGPSSSPKK----KKVKIKSDDSPLDDVKS 584

++ +F P+AGPS PKK KK + D +DDVKS

Sbjct 495 REKKYFNFASNLPHREVMKKICQEIAKHPIAGPSRDPKKGKQLKKSQSSDSDESVDDVKS 554

Query 585 IADCIIHNSLNKIDFEDD----NLIQNVEERRQDEQ---AEP-QVVENGDLANNNRDDEG 636

I CI+ NSLNKI+ +D+ NLIQ + E+ EP +VENGDLANNNRD EG

Sbjct 555 ITTCILENSLNKIECQDEDIRGNLIQELIPEENLEEIPVVEPILIVENGDLANNNRDLEG 614

Query 637 NNFVADVVEPDEGIDVLNDNVEELVAPVADNAAVAGNNNDDNVLGAFEAPIVVIVEEPLE 696

NN+ A+ VE ++ I DN + NN+D + +A +E

Sbjct 615 NNYPAEDVEQNDEI--------------VDNQELINANNNDVEVAVAQAEEDEEENIEIE 660

Query 697 QRIELDDVAQASREALFDPDSDQDLLLQDFDLPSQSSSQEPPAASIQTNTI--------- 747

+++ +VA +SREALFD +S D LL+DF++ ++S S + +

Sbjct 661 PPVDV-EVAWSSREALFDINSQVD-LLEDFEM--EASDVVVNGVSFPNSCVIVAENNDPV 716

Query 748 -----SGFPSWLLQILGGHAI---STDV--EPHFYCQGDGLGVHPSVSVLD 788

SGFP+WLLQI + D+ EPHFYCQGDGLGVHPS ++

Sbjct 717 LPPEESGFPNWLLQIFDEAEVLPPEDDLHDEPHFYCQGDGLGVHPSTVAIN 767

Graphical representation


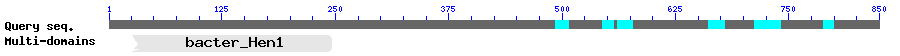


**ATP-dependent RNA helicase Belle**

>Cp.comp37673_c0_seq4 len=5386

CCTATTTACATACTTTATTTGTATAAATTGTTATAAAATAAAACGATTGTTAACTTTAAATTTTTATTTAGATATAATTTCAAGTATGCTTTAAAATCACTTCTACATGAACAAACTGTAAATGCATTATTACCAAAGGTAAAATTCTAGTGGGGAAACTAAATATCGGAGCATTTTACCATTCCTTTTCAGATAGAATAAAAAAAAAGAACAGGTTCCCTAAAATATCGCTTAACATATATTCCAAAAATTATGTTCGTTAACTGGTTAAGTGATAGAGAACAAGTAACTAAAGCACCACCATTAGTACAATAGTACGACAATTGCCAAATACTCACTTGTCATTACTTATCCTCTGAATGGGTAACCCGTTTTGCACAGTTGCAGTCACAGCTCCCAACTTGAAAAAGTGTTCAGAACTCATTCTGTTAGAATAGGCACGCAATTTTCCAACCATATCACAGGATGATAAGCATATAACAAATCTTTATATAATTTATCTGAAATGTTCCAAATGTCAACAGAAAAGCCTTACAGGCACAAAAATATTGTGCCCACTCTAACTAATGTTAAACACTGATTTAATATTTATTTTAAATAAATAAAAATAAAATTACAAAAAAAGTTAAATTAAAACATTATAATTCACACGCTAAGATTTATCCACTGAGTCTTGATGACTGCTTATGACATTCGTTTCTCGGCATATACACGGTTTAAACGTACTATGTTTATTACAATATAGATCGTTAATATCACAGTCAAATTCAAGAATACTCTTAGCGCTCTAAATAGCTGTAACTGCAACGCTATTTGAGCCCTGGACCCCTGATTAAATTATTAGCCTTAAATACTCTTATTCTACTTTAATCAAGTTTTTCCAAGCAAAAGTGGTCGCTGGTAGGAGAAAAAACGAACGTTTTACAATGACAGAGATGAAAACATTACGAGGATTAGAATGCACCATTTGGAGATTTTCGTTTCCCTAGGTTATTCGGACAACAGCTACGGTTCTCTCAAACCAGATTTTTAATAATGGTTCTTTAAGTATTATAAAAGCATAGGTATATAAATTGTTACAATTATTGTTAGATAACAAATAAAAATTCAAAGGAAACTGTAGTATTTAAAATTGTATATATAAATTATGAGGTATTCCTAAATGTCATGTTGATGTTAGCGTTGCATCGATTAAGCGTAAGGAAAGGCGATTCCCTCAAGAAAATAATGTTCAAGAGGAAAAAGAGGAAAAACGACAGGGACAAGCTTATAATGGTAATGCTATTAGAAGAGGCTATGTTTCTTTTCATATTTTGAAGGTCTGTTTGACATTCTCATATATTATGACAGCAATCCTACTTTAAATTGGCTTGACTACTATGAAGAACGTCAGTTTCTCAAAAGAATTTAATGAAAAACGTGATTTTTTAAAGATTTTTTTTAATTAATAAAAACTGACATGACACTCAGGAGCACCTAGTATAATAACTTTTAGCCTAACATTGAGGAGGTTTCTTGAATGTGTTTATTTTTTTATTTATATGAACTTGTTTTTGTATATACAGTGTGTGCCATAATGTTGAATATATAAATAAACTTTTTGGGCCAAGTTATTTTGCCAACTAAACAAAAAAAAAATGGTTCTATGAACTATTGAAGAATGTTAATTAATTTTTTCAATCAATTTTCAATTTCACATAATAATATAGTGAATGTCGCCATTCAAGCTCTAAATTTGCGCATTCAAAATGGTTTTGAAAACAAAGGCTATAGTCCCAAAAATTTATTTTGTAACAGGTACATTAAAAAACACAATCCCAATTTATGCCACACACTGATTTAACTTGATTAAAAAAAGTAATGTTCATAGAGTTAAAAAAATATTTTGGATTAGAAAAATTTGTATATGAGCACTTACATGAGAGTTCCACAATACTGAAGATACGTTGCAAACGCTTTTTTTAAATTAAGAGTGAACAAAATTATTTATCCTTTTGCTTTTAATTTTTGGCCATTTTACAAAAAAAAAATTGTGATCAAATTTCGTGAAAAGTTGCGAGAACAAAACTTACGGCAATAGTTCTGTGCACATTATCTGCGAAAAATCTCGCTACTCTTAAGCTGCATTTATAATCACTCTGCACTGCAGAGTGCAACATGAAACCGTGCTGCAATTTTATTGTTATCATGTTTCACGGTAATGCATGAAATATATTTATAACCTAAAAAACAGTAAACCAATAGATCCAGTTAATTCAATCATTCAGATTAGTGTGTAAGCTTGCATAACAGTAGATCATCAGATAATGGTATATATTGTTGAGAATGTCAAATTCTTGCCTAAAGAAAACTAGGTTAAACTTAGTTTAAATATGTAATTATTGAACTGCAGATCAAGTTGAACTCGGATTAAAATATGATTCTACAACCAGGCCTAAGGAGCACTAACTTTTTCCTTGTCTGAAAAAATGTAGTGCATTTTTTTCTGTGATCAATTTAAAGAAAGTAAAAGGCGCAAACTGTTGCAAGTAAAATTAAATGAAATAAACTGAGAAGTTCAACTTGTATTATAATACTCCACCTAGAGAAGAACTGCATCATTTGGGAAAATAGGAGTTGGTAAAAAGTTTGAAATTAATTTTAATGGAAGAAAAATTACATATCGCTCTTGGAATAGTAAAACTTTTTCAGCAAATCAATTTTAGCGAAAGAAATAAGCAAAATAAACACATCTAAAGAATTATGCAGAAGTTACAGTGAAAGTGCAGACAGAAGTTTAACTGAAGGAGTAGATTGGTATAAACAAGTTTGTGAAACAAATTTACATGTTGTTGTGTATTTACTTTTACCTTATTGGTAAGGATAAAGCTAATATCTACTTTTATTAATAAATATGCATTTTAATTATCTATAGTATAGATTTGGAAAATGTTAAAAACAAAATGTTTAATTATCAGATGATGATTTTGAATATGCTTTTCTATGTTAAAAAAAAACTCTTGAAGTACAATAAGATGCTTGATGTATTTTTCGATAAGGGAAACACATCTATGTGGATCATTTAGGTTTACCACCAATCATCCTGATTGCTGGAATTGTAGTTTCCACCAAAGTTGCTTCCTCTATCCATACCTCCTCCACCATAATGGTTGCCACCTCCATTGCCATATCCATTGTTACTATATCCCCCTCCGCCTGATCTCTGATTACCTCGAGAATTGCCGCCAGACTGTTGACGGTAATCTCTGCTACCAAAACTGCTACCTCCACCGCCACCTCCACCATAACGGCCTTTACCACCTCTTCTGCCGGAATTGGGCATTCGACTATCGGAAGCAACACTTTCTAACCAGGATGGATATTCCTGCTTTGCTTCGATAAGCAAGTCGAGCATTCCGCTGGCCAGATTTCGGTTCCTATCATTGAAGAAAGAAGTGGCCAAACCAAGATTGCCCATTCTGCCGGTACGTCCGATTCGATGAACATATTCTTCGATATCTGAAGGTAGGTCGAAATTAATTACATGTTTAACATGGGGAATATCTAAGCCACGAGCCGCAACGGCAGTGGCGACCAGTATCGGAGTGTTTCCTGAACGGAACTGTTTCAATGCATCTTCTCTTTCTCTTTGTGTACGATCGCCGTGTATCGAAGTCACCGGGTAACCTTCTTGATGAAGAAAATCTTCGAGAGAGTCCGCTCCTTTCTTGGTTTCGACGAATACCAATGTCAAGCTTTCTGCTGACGGCTGGTGCAAATCATTTACGTTCAACAGGTCGAGTAGCAGCGATCGCTTGTCGTGTTCTTCTACCCACATAACCTTTTGTGTAATATTCTCAGAAGTGGAACCGACACGACCCACCGCCAAAAATATATAATTATCAAGAAAGTCGCGTGCCAACATCTGAATGGGATGAGGAAAAGTGGCTGAAAACATAAGGGTTTGCCTTTCCCCTGTTCTAGGCATAGATTCTTTTTCGACGATTCTGCGGATTTGTAACTCGAAACCCATATCTAACATTCTGTCGGCTTCGTCTAGAACCAAGTATTTGCAATAGTCCAGGCCAATTCTGCCTCTGTCGATCATGTCTAGCAGACGTCCCGGGGTCGCTACCAACAAATGACACCCACGCTCCAAGTCACGTAGCTGGTCTCCAATGTGAGCACCACCGTAAACAACACAAGGACGCACTCTAGATCTGTAAGCAAATTTTTTGCTTTCATCGTAAATCTGGGTCGCTAATTCTCTAGTGGGCGCTAACACAAGGCCGAGAGGGTACTGTTTGCTGCGACGACTACGACCGTGTTGAATATTTGGCGGCCCCACTTCATACATCTGATTCAATATGGGAACAAGGAAGGCTGCGGTTTTACCAGAACCGGTCTGAGCACAAGCCATTACGTCTCTTTTACTCAAAATAATCGGAATTGCATATTTTTGCACCGGAGTCGGTGTATCGTAACGAGCAGCCGCAATGTTACTGCGAATTATTTCTGTCAACTGTACTTCCTCAAAAGATGCAATGTGGCGCGGGACTTTATCCCCGGTTGCTTCAACAGGTATATCTTCGTATTTGCTGAAGTTAATACCCGTATTCCCTGTGCCGAAAAGTTCCATTTCGAGGCGTTCGTCCCTCGCCAAGGGAATAGTCCAATCGTTTTCGTTGCGTCTCTCACGATTGTCATTCCATCGACCATTCCCTCCTTCTCGGCTATTTGAACTTTCATTCCAACGATCTTTGGGTTTTTCAGGCTCTTGCCAACGATCGTTGCGTGGTAAATCGCGCTCTCTTTCCTGAAATCTGCCACCTCCTCGTGCCTCTCTTCCCCACTCCTCCTTTTCCCTATTCTCAAATACCTCCCCATTCTGAAAATTGTCTCTTCTATTCCTTGTGTTGAAACTGGAAAAATCTCCACGGTTTTCACGAGCGTCGCGGCTTCCTCCGCTCCCCCCGCCTCTGTTGAAGTTGCGTCCTCCTCCTCTCGACTCGCCTCGTCCCTTCTCTCTATCGTAGCTAGAACCTGACTCGGGCTGGCTACTATGCTGTTGCTTATTACGCAAGTGCGGTGGCACGTAACGGTCGCTTATTCCGCGACTCTGCAAGTCCAGACCAGCAAATTGCTGCTCTAGACCTGATCCATTTTGATTGGGTGCATTACTCATATTACTACAAATCAGTACTTATGGTAGAAGTTTCGATTCTCAAACTTCTAAAATTTATTCCTTAACAAAATATCTTTCACCAACACAAAAGCCAATAGTTTATCCTCTACGAGATTTTTTTCCTTGAATTGCGACTGCGATTGCGCGACTGAAATAACTGGTAGCACTGGCTGTCAAATTAAAGGGAAATTTTATATTTCCACCATATAAAAAAACTAAATT

Protein RF -1: -5164->-3068 (698AA)

Comparison with *Tribolium* ATP-dependent RNA helicase belle (699AA)

Query 1 MSNAPNQNGSGLEQQFAGLDLQSRGISDRYVPPHLRNKQQHSSQPESGSSYDREKGRGES 60

MSNAPNQNGSGLEQQFAGLDLQSR S RYVPPHLRNKQ + SSYDR+ RGES

Sbjct 1 MSNAPNQNGSGLEQQFAGLDLQSRAPSGRYVPPHLRNKQSSAE-----SSYDRD--RGES 53

Query 61 RGGGRNFNRGGGSGGSRDARENR-GDFSSFNTRNRRDNFQNGEVFENREKEEWGREARGG 119

RGG N GG R+NR GD+SSFNTRNRRDNFQNGE FE E G GG

Sbjct 54 RGGSGRSNYSSRGGG----RDNRAGDYSSFNTRNRRDNFQNGETFEREEWGR-GGGGGGG 108

Query 120 GRFQERERDLPRNDRWQEPEKPKD----RWNESSNS-------REGGNGRWNDNRERRNE 168

GR QERERDLPRNDRWQEPEKP++ RW+++ N GG GRWNDNR+R NE

Sbjct 109 GRQQERERDLPRNDRWQEPEKPREGGGGRWSDNRNENRGGGGGGGGGGGRWNDNRDRHNE 168

Query 169 NDWTIPLARDERLEMELFGTGNTGINFSKYEDIPVEATGDKVPRHIASFEEVQLTEIIRS 228

NDWT+P+ RDERLE ELFGTGNTGINFSKYEDIPVEATGDKVPRHI SFEEVQLTEIIR+

Sbjct 169 NDWTVPMPRDERLEQELFGTGNTGINFSKYEDIPVEATGDKVPRHITSFEEVQLTEIIRN 228

Query 229 NIAAARYDTPTPVQKYAIPIILSKRDVMACAQTGSGKTAAFLVPILNQMYEVGPPNIQHG 288

NI ARYDTPTPVQKYAIPII+ KRDVMACAQTGSGKTAAFLVPILNQMYE GPPNI HG

Sbjct 229 NINLARYDTPTPVQKYAIPIIVGKRDVMACAQTGSGKTAAFLVPILNQMYEHGPPNITHG 288

Query 289 RSRRSKQYPLGLVLAPTRELATQIYDESKKFAYRSRVRPCVVYGGAHIGDQLRDLERGCH 348

RSRR KQYPLGLVLAPTRELATQIYDESKKFAYRSRVRPCVVYGGAHIGDQ+RDL+RGCH

Sbjct 289 RSRR-KQYPLGLVLAPTRELATQIYDESKKFAYRSRVRPCVVYGGAHIGDQMRDLDRGCH 347

Query 349 LLVATPGRLLDMIDRGRIGLDYCKYLVLDEADRMLDMGFELQIRRIVEKESMPRTGERQT 408

LLVATPGRLLDMIDRGRIGLDYC+YLVLDEADRMLDMGFELQIRRIVEKE+MP+TGERQT

Sbjct 348 LLVATPGRLLDMIDRGRIGLDYCRYLVLDEADRMLDMGFELQIRRIVEKETMPKTGERQT 407

Query 409 LMFSATFPHPIQMLARDFLDNYIFLAVGRVGSTSENITQKVMWVEEHDKRSLLLDLLNVN 468

LMFSATFP PIQMLARDFLDNYIFLAVGRVGSTSENITQKV+WVEEHDKRS LLDLLN

Sbjct 408 LMFSATFPSPIQMLARDFLDNYIFLAVGRVGSTSENITQKVVWVEEHDKRSFLLDLLNAA 467

Query 469 DLHQPSAESLTLVFVETKKGADSLEDFLHQEGYPVTSIHGDRTQREREDALKQFRSGNTP 528

++ QPSAESLTLVFVETKKGADSLE+FLH EGYPVTSIHGDR+QREREDAL+QFRSGNTP

Sbjct 468 EMSQPSAESLTLVFVETKKGADSLEEFLHFEGYPVTSIHGDRSQREREDALRQFRSGNTP 527

Query 529 ILVATAVAARGLDIPHVKHVINFDLPSDIEEYVHRIGRTGRMGNLGLATSFFNDRNRNLA 588

ILVATAVAARGLDIPHVKHVINFDLPSDIEEYVHRIGRTGRMGNLGLATSFFNDRNRNLA

Sbjct 528 ILVATAVAARGLDIPHVKHVINFDLPSDIEEYVHRIGRTGRMGNLGLATSFFNDRNRNLA 587

Query 589 SGMLDLLIEAKQEYPSWLESVASDSRMPNSGRRGGKGRYGGGGGGGSSFGSRDYRQQSGG 648

SG+LDLLIEAKQEYPSWLE VA+D RMP+SGRRGGK RYGGGGG RDYRQQSGG

Sbjct 588 SGLLDLLIEAKQEYPSWLEGVAADGRMPSSGRRGGKSRYGGGGGSSFG--GRDYRQQSGG 645

Query 649 NSRGNQRSGGGGYSNNGYGNGGGNHYGGGGMDRGSNFGGNY----NSSNQDDWW 698

SR NQRSGGGG G G GG+DRG NFGGNY NS+++DDWW

Sbjct 646 MSR-NQRSGGGG-GYGNNGFGNNGGGHYGGLDRGGNFGGNYNSNSNSNSRDDWW 697

Graphical representation


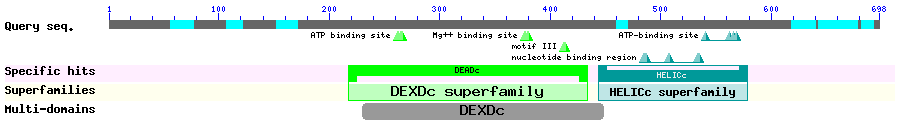


**Similar to pre-mRNA splicing factor ATP-dependent RNA helicase PRP16 (Tribolium), mut6 homolog (Chlamydomonas)**

>Cp.comp39484_c0_seq1 len=4010

CGTCGCCCTCTTGGATTTCTACAACACCAAAAAGTCTCAGAAACCTTGTTGAAAAAAACGTCATGTGACGTCATATTTGTTGCAAAAGCATCTCTGTTTAAACACAAATTCAAATTGTTGGTCCCGAAAATGCGTCTTAATCACGTGAAATATAATTAGAGGCACTTTTCGCCAACTAAGTTAGCATAAAGTGACACAACTATAAGAAAAACAAAGCCAAAAGAGGAAAATGGCGCTCTACAACTAAACAAAGAAGAAGACATTTTAAGGTTAAGTTAATATTTTCGAACAAAGAATTGTTTTGAATTTTCAATAGAATTAGCTAATAATGGACGAAGAAGACGGCGTGCACCGCTTGGAAGGCACGACGGGCAACGAGAAGGGGGGTCTCGTAGTTAAAAAGAAAACCGGGAGTACTTTCAAGGTGCCCCAGGTGTCCCTGCTCGGTTTGGATCGTTTGGCGGCGATCAAACGTAAAGAAAAAGAAGAGGCGGCGAGGAAAATGTCGTTCTCGATGGACGACGAAGCGGCTGCGGAAAATTCCGACGATCACGAGGGTCGTAAAGTGCCAAAAGACGGTCGAAAGTTTCGGTCGCCTGCCGAAGAGACCCCTACGTATACCGGGGGAGTAAGCAAAGAAGCGAAAGACAGATTAAGAGAACACATGAAGAATAAGTACAAGGAAAAAGGCGTGTACGCTTCTACTAAAGATAGACGCGACAGGGAAAGAAAACGGGACAGAGGAGATAGATCCGAGAGAAGGAGAAACAAAGATAGTTCTAGGCGAGAGGAAACTCCTAAGTTTAGGGACGAACCGCGCACCCCTAACATCAAAATAAAAGACAATACGGCGAGGACTTCGTGGGAAGACGAAGACGAATCAGTTCCGGTTAAAAGATCGTCGTGGGACTTTCCGACACCGACGAGTTACAAGCGAGAAGACCCTAGCTGGTCTGAGAGAAGTTTCCGAACGAAATACGAGTCGTCCAGGTCGTTTAAACACGAACGGAGTAGCCGAGACTCGAAAAGCTTTAGAAGATACTCCGATGATACTCCGAGAGCCACTCCCGCTCATAAATTCAACAACTGGATGAAGGACCGGAAGAGAACCGGAGCTACTCCAGGCACCGGTCGCGACAAGGACGAACCGTTGAAATGGGACGCCACAGTGGACAGAGAAAACTGGGAGGAGGAGCAGAAGAGACTGGACAGGGAGTGGTACAATATGGACGAAGGGTACGACGACGAAAACAACCCCTTTTCGAGCGTTAGCGACGAGTACACGAAGAAAAAGGAAGAACAGCTGGAACAGAGGAAGAAGAAACGAATGTCGGCCCAACAAAGACAGATCAATAAGGATAACGAATTGTGGGAAAGAAACCGAATGTTAACTTCAGGTGTCGTACAGTCTGTAGACTTGAACGAAGACTTCGACGAAGAATCCATAGATCGGGTTCACTTACTGGTCCACAATATAGTACCACCGTTCTTGGACGGTCGAATCGTCTTTACCAAACAACCGGAGCCTGTCATACCCGTCAAAGATCCCACTTCTGACATGGCGTTGGTGGCCCGCAAAGGTTCCCATCTGGTCCGAGTGTACCGCGAGCAAAAAGAGAGAAGAAAGGCCCAAAAGAAGCACTGGGAATTGGGCGGCACGAAAATAGGCAACATTATGGGCATCAAGAAGAAAGAAGACGAAGAGGACAAAAAATACAACAAGGAGGACGACACGACGGATTATAAAGCGGACCATAAGTTCGCCGAACACATGAAAGGTTCCAGCGAGGCGGCGAGCGATTTCGCCCGCAAAAAATCCATATCGGAACAGAGAAGATATTTACCTGTATTTGCCGTAAGGCAGGAACTGTTAAATGTTATTAGGGAAAATTCCGTCGTTATTATCGTCGGCGAAACCGGTAGCGGCAAAACGACCCAACTGACCCAATACCTACACGAAGACGGCTACAGCAAGTACGGCATGATCGGGTGCACCCAACCCCGAAGGGTGGCGGCCATGTCGGTCGCGAAACGAGTCAGCGACGAAATGGGCACTCAACTCGGCGACGAAGTCGGTTACGCCATCCGATTCGAGGACTGCACCTCGGAAAATACCGTAATTAAATACATGACGGACGGTATTTTGCTTCGGGAAAGTTTACGCGAGCCCGACTTGGACCATTACAGCGCCATAATCATGGACGAGGCCCACGAACGGTCATTAAGTACCGACGTTTTATTCGGTCTGTTAAGAGAGATCGTAGCGAGACGTCACGACTTGAAACTCATAGTGACGTCGGCCACGATGGATTCCAGCAAGTTTTCCATGTTTTTCGGGAACGTACCCACGTTTACCATACCGGGACGGACGTTCCCGGTCGAAGTTTTGTTTAGCAAAAATCCGGTGGAAGATTACGTCGACGCGGCCGTTAAACAAGCGTTGCAGATCCATTTGCAGCCCCCTTCGGGGGATATTTTGATCTTTATGCCCGGTCAGGAGGATATCGAAGTTACTTGCGAGGTTTTGGCCGAGCGGTTGGGCGAAATCGATAACGCCCCCGAACTGTCGATTTTGCCGATATATTCGCAGCTGCCGTCCGATTTGCAAGCGAAAATCTTCCAGAGATCGCCGGAAGGCATCCGGAAGTGCGTCGTGGCGACTAATATTGCCGAAACGTCTCTGACGGTCGACGGGATCATATTCGTGATCGATTCAGGCTACTGCAAACTGAAAGTCTACAATCCGAGGATAGGTATGGACGCTTTGCAGATTTACCCCATAAGCCAAGCGAATTCGAACCAAAGGTCGGGAAGGGCGGGTCGTACCGGTCCGGGCCAAGCGTTCCGCCTCTACACCGAACGCCAGTACAAAGACGAACTGTTGGTAACGACCGTACCGGAAATACAACGTACCAACTTGGCAAACACCGTACTGCTCTTGAAGTCGCTCGGTGTGCAAGATCTGCTCCAGTTTCACTTCATGGACCCGCCGCCCCAAGACAACATACTAAACTCGTTGTACCAGCTGTGGATTTTGGGCGCCCTAGACCATACGGGAGTGTTGACAAAATTGGGAAGACAAATGGCGGAGTTTCCATTGGACCCGCCACAATGCCAAATGCTCATAGTGTCCACCCAGATGGGCTGTACGGCCGAAATTTTGATCATAGTGTCCATGTTGTCCGTCCCGTCGATATTTTACCGGCCAAAGGGACGGGAAGAGGAAGCAGACGGAGTGAGGGAAAAGTTCCAGGTGCCCGAAAGTGACCATTTGACGTTCCTCAACGTTTACAATCAATGGAAGCAGAACAATTATTCGTCGCATTGGTGCAACGAGCATTTCATACACGTAAAAGCGATGCGCAAGGTGCGAGAGGTCAGGCAGCAGCTTAAAGATATCCTGGTGCAGCAAAAGTTCGAGGTTAAGTCGTGCGGTACCGACTGGGACATCGTTAGGAAGTGCATTTGTTCCGCCTATTTCCACCAAGCGGCCAGATTGAAAGGTATCGGGGAATACGTCAACTGTCGTACGGGCATGCCGTGTTACCTCCACCCGACATCCGCATTGTTCGGTCTCGGAAACACCCCAGATTACGTGGTGTACCACGAACTAGTGATGACTGCCAGAGAATATATGCAGTGCGTCACATCGGTGGACGGACACTGGCTGGCCGAACTCGGACCGATGTTTTTCTCCCTCAAAGAAACGGGCAAATCGGGCAGAGCCAAGAAAAAGCAAGCGGCCGAGCACCTGCTAGAGATGGAAAATCAGATGCAAGTGGCGCAGGAAGAGATGAAAGCTAGGAAAGAGGCGGCTGACAAGAAGGAGGCGGCTATGTACAAGGGGCAGGAAATAGTCAGTGCCGGAACCACCCCGAGACGGACACCGGCTAGATTTGGATTGTAAACGAGAAAATTTTGAAATTGTAAGTTTATTGTTTAATAAAAATTAATTTTTGTTCATCATTCGGGTAGGTCAACGAACGAATTAAACGCCGCACTTTCACAGA

Protein RF 2: 329->3907 (1192AA)

Comparison with *Tribolium* (AA)

Query 1 MDEEDGVHRLEGTTGNEKGGLVVKKKTGSTFKVPQVSLLGLDRLAAIKRKEKEEAARKMS 60

M+ E+ +HRLEG + ++KGGL+VKKK TFKVPQ SLLGLDRLAA KR+EKEEAARKMS

Sbjct 1 MESEENLHRLEGIS-DQKGGLIVKKKP-PTFKVPQPSLLGLDRLAAAKRREKEEAARKMS 58

Query 61 FSMDDEAAAENSDDHEGRKVPKDGRKFRSPAEETPTYTGGVSKEAKDRLREHMK-NKYKE 119

F+MDD ++S K KD RKFRSP ETPTYTGG+S EA++RL E +K NK KE

Sbjct 59 FTMDDNDNTDDSSSLLKEKHSKDSRKFRSPHNETPTYTGGISDEARERLIERLKSNKQKE 118

Query 120 KGVYASTKDRRDRERKRDRGDRSERRRNKD---SSRREETPKFRDEPRTPNIKIKDNTAR 176

KGVYA+TKDR + D+ R SS R +TP+FRDEP+TPN+ KD ++

Sbjct 119 KGVYATTKDRHRDRDRDRERDKDRDRGRHRDRESSHRSKTPRFRDEPKTPNLGHKDEISK 178

Query 177 TSWEDEDESVPVKRSSWDFPTPTSYKREDPSWSERSFRT-KYESSRSFKHERSSRDSKSF 235

+SW+D+D+ P K+SSWDFPTP++YK WSERS ++ KY+ S+ RSSR+SK

Sbjct 179 SSWDDDDDVGPSKKSSWDFPTPSTYKGSGGDWSERSTKSRKYDESK-----RSSRESKR- 232

Query 236 RRYSDDTPRATPAHKFNNWMKDRKRTGATPGTGRDKDEPLKWDATVDRENWEEEQKRLDR 295

R+Y D++ R TPAHK+N+W KDRKR+GATP G KD +KWD TVDRE WEEEQKR+DR

Sbjct 233 RKYEDESARFTPAHKYNSWAKDRKRSGATPMPG--KDGVIKWDNTVDRELWEEEQKRIDR 290

Query 296 EWYNMDEGYDDENNPFSSVSDEYTKKKEEQLEQRKKKRMSAQQRQINKDNELWERNRMLT 355

EWYNMDEGYDD NNPFSSVS+EYTKKKEEQLEQRKKKR+SAQQRQINKDNELWERNRMLT

Sbjct 291 EWYNMDEGYDDGNNPFSSVSEEYTKKKEEQLEQRKKKRLSAQQRQINKDNELWERNRMLT 350

Query 356 SGVVQSVDLNEDFDEESIDRVHLLVHNIVPPFLDGRIVFTKQPEPVIPVKDPTSDMALVA 415

SG V S+D NED+DEESIDRVHLLVHNIVPPFLDGRIVFTKQPEPVIPV+DPTSDMA+V+

Sbjct 351 SGAVHSIDFNEDYDEESIDRVHLLVHNIVPPFLDGRIVFTKQPEPVIPVRDPTSDMAIVS 410

Query 416 RKGSHLVRVYREQKERRKAQKKHWELGGTKIGNIMGIKKKEDEEDKKYNKEDDTTDYKAD 475

RKGSHLVRVYREQKER+KAQKKHWELGGTKIGNIMGIKKKEDEEDK++NKEDDT DYK D

Sbjct 411 RKGSHLVRVYREQKERKKAQKKHWELGGTKIGNIMGIKKKEDEEDKRFNKEDDTADYKTD 470

Query 476 HKFAEHMKGSSEAASDFARKKSISEQRRYLPVFAVRQELLNVIRENSVVIIVGETGSGKT 535

KFAEHMK S+EA+SDFA+KK+I EQRRYLPVFAVRQELLNVIRENSVVIIVGETGSGKT

Sbjct 471 QKFAEHMK-STEASSDFAKKKTILEQRRYLPVFAVRQELLNVIRENSVVIIVGETGSGKT 529

Query 536 TQLTQYLHEDGYSKYGMIGCTQPRRVAAMSVAKRVSDEMGTQLGDEVGYAIRFEDCTSEN 595

TQLTQYLHEDGYSKYGMIGCTQPRRVAAMSVAKRVSDEMGTQLGD+VGYAIRFEDCTSEN

Sbjct 530 TQLTQYLHEDGYSKYGMIGCTQPRRVAAMSVAKRVSDEMGTQLGDDVGYAIRFEDCTSEN 589

Query 596 TVIKYMTDGILLRESLREPDLDHYSAIIMDEAHERSLSTDVLFGLLREIVARRHDLKLIV 655

TVIKYMTDGILLRESLREPDLDHYSA+IMDEAHERSLSTDVLFGLLREIVARRHDLKLIV

Sbjct 590 TVIKYMTDGILLRESLREPDLDHYSAVIMDEAHERSLSTDVLFGLLREIVARRHDLKLIV 649

Query 656 TSATMDSSKFSMFFGNVPTFTIPGRTFPVEVLFSKNPVEDYVDAAVKQALQIHLQPPSGD 715

TSATMDSSKFSMFFGNVPTFTIPGRTFPVE+LFSKNPVEDYVDAAVKQALQIHLQPPSGD

Sbjct 650 TSATMDSSKFSMFFGNVPTFTIPGRTFPVEILFSKNPVEDYVDAAVKQALQIHLQPPSGD 709

Query 716 ILIFMPGQEDIEVTCEVLAERLGEIDNAPELSILPIYSQLPSDLQAKIFQRSPEGIRKCV 775

ILIFMPGQEDIEVTCEVLAERL EI+NAPELSILPIYSQLPSDLQAKIFQRSPEGIRKCV

Sbjct 710 ILIFMPGQEDIEVTCEVLAERLAEIENAPELSILPIYSQLPSDLQAKIFQRSPEGIRKCV 769

Query 776 VATNIAETSLTVDGIIFVIDSGYCKLKVYNPRIGMDALQIYPISQANSNQRSGRAGRTGP 835

VATNIAETSLTVDGIIFVIDSGYCKLKVYNPRIGMDALQIYPISQAN+NQRSGRAGRTGP

Sbjct 770 VATNIAETSLTVDGIIFVIDSGYCKLKVYNPRIGMDALQIYPISQANANQRSGRAGRTGP 829

Query 836 GQAFRLYTERQYKDELLVTTVPEIQRTNLANTVLLLKSLGVQDLLQFHFMDPPPQDNILN 895

GQAFRLYTERQYK+ELLVTTVPEIQRTNLANTVLLLKSLGVQDLLQFHFMDPPPQDNILN

Sbjct 830 GQAFRLYTERQYKEELLVTTVPEIQRTNLANTVLLLKSLGVQDLLQFHFMDPPPQDNILN 889

Query 896 SLYQLWILGALDHTGVLTKLGRQMAEFPLDPPQCQMLIVSTQMGCTAEILIIVSMLSVPS 955

SLYQLWILGALDHTGVLTKLGRQMAEFPLDPPQCQMLIVS+QMGCTAEILIIVSMLSVPS

Sbjct 890 SLYQLWILGALDHTGVLTKLGRQMAEFPLDPPQCQMLIVSSQMGCTAEILIIVSMLSVPS 949

Query 956 IFYRPKGREEEADGVREKFQVPESDHLTFLNVYNQWKQNNYSSHWCNEHFIHVKAMRKVR 1015

IFYRPKGREEEADGVREKFQVPESDHLT+LNVYNQWKQN YSSHWCNEHFIH+KAMRKVR

Sbjct 950 IFYRPKGREEEADGVREKFQVPESDHLTYLNVYNQWKQNKYSSHWCNEHFIHIKAMRKVR 1009

Query 1016 EVRQQLKDILVQQKFEVKSCGTDWDIVRKCICSAYFHQAARLKGIGEYVNCRTGMPCYLH 1075

EVRQQLKDILVQQK E+KSCGTDWDIVRKCICSAYFHQAARLKGIGEYVNCRTGMPC+LH

Sbjct 1010 EVRQQLKDILVQQKLEIKSCGTDWDIVRKCICSAYFHQAARLKGIGEYVNCRTGMPCHLH 1069

Query 1076 PTSALFGLGNTPDYVVYHELVMTAREYMQCVTSVDGHWLAELGPMFFSLKETGKSGRAKK 1135

PTSALFGLG+TPDYVVYHELVMTAREYMQCVT+VDGHWLAELGPMFFSLKETGKSGRAKK

Sbjct 1070 PTSALFGLGSTPDYVVYHELVMTAREYMQCVTAVDGHWLAELGPMFFSLKETGKSGRAKK 1129

Query 1136 KQAAEHLLEMENQMQVAQEEMKARKEAADKKEAAMYKGQEIVSAGTTPRRTPARFGL 1192

KQAAEHL EMENQMQVAQEEM+ARKEAADK+EAAM KGQEIVSAG TPRRTPARFGL

Sbjct 1130 KQAAEHLQEMENQMQVAQEEMRARKEAADKREAAMNKGQEIVSAGATPRRTPARFGL 1186

Graphical representation


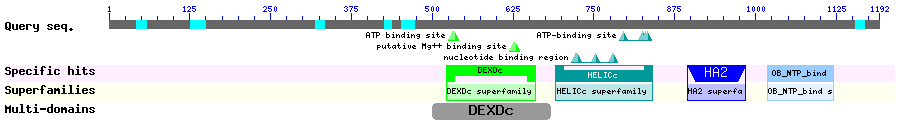


**Gemin3 homolog**

>Cp.comp40453_c0_seq1 len=1520

AAAGAATTTGTTGGTTAATGTCTCATAAACATAAACAATCTTGAATTTATTCGATAAAATCATGTCAAATCTTAAGCAATTGGCTCATTCCTTGGAAGATAAAATCAGAACCAAAGACGTTATTTTGCAAGAAAATGTTTCGTTCGATTCACTTCTTCTACCCAACAATATCCTTAAGGGTTTGGCGAAACATGGTTTTAAGAAACCGTCACCCATTCAAATTAAAGCAATTCCCATAGGAAGATGCGGGTTCGATTTGTTAGTTAAATCCAAATCGGGAACTGGCAAGACTCTGGTGTTTGTAATCGTAGCACTTGAATCTATAGATTTGTCTAAAACGAGACCTCAGGTTATAGTCGTAGCTCCGACAAGAGAAATAGCGGTACAAATAGGAGACGTATTTAAAGATATTGGAGTCAATTTTGATGGGCTAGTAGTGGAGAATTTCATAGGAGGTTTCTCCATTGAATCAGATAAACTCAAATGCAAGTCTTGCCACGTTGCAGTCGGTACACCAGGTAGGATAAAACAGTTAATAAAAATCGGAAGTCTAAACGTGAGTGCTGTGAGAATTTTCGTACTCGACGAAGCCGATAAACTGATGGAAAATAGCTTTCAAAACGACATTAATGAAATTTACAACAGTCTACCTAGCAAAAAACAGGTTATAATGTCTAGCGCTACTTATACGAAAGAACTGGAGACTCTACTGACCGACTATATGCTCAGTCCGACTTATGTCAGCGCCGAATTGGAGACCCCGTTGCTTTTAGGACTGAAACAATTCGTTAGGATAATAAAATCGTCTAATAACGTAGTGCAACAGACCAAAGTTAAAAACGAAGAATTGTTAAAAATTTTCACGCTTATACCTTTTACACAATGCTTGGTATTTTCAAACTATCAAACACGAGCCGAAAGTATAAGCAATTTCCTTAACCGCAACAGTTGGAATTCCTTGTACATATCCGGCGCCCAAAATCAAACCGATCGACTCGCAGCCCTATCCGATTTGAAACAATTTCATTGCCGGATATTGTTATCTACAGACCTTACGGCTCGAGGTATCGATGCCGCCAATGTTGATTTAGTTATCAATTATGACGTACCAATAGATGCCATGACTTATTTACATCGCATGGGCAGAGCCGGTAGGTACGGCTCCTCAGGGATTTGCATTAATTTGGCATCCGAAGGTCCGGAGGTTCAACTGTTGCAAAATATTTTGGGCGTTATTGGGGGCACAAATTTATCCGTACCGAAATTAGATGATTCGTTTAATTTAAAAGAAGTATCCAATGAGAATTTGCTCTTTGGAGTTGTTCCGGAAAACAGTGATGTGTTCGATCAGTTTAAAGCTGAAGTGAGGAAATTAAAGGCAAAATCTGCAAAGAAAAAGAATAAGTCTGACAAAATAAAAGACGGTTCAGATACAGTTAAAGAACATTTGATGGATACTGACCCTATGTCAATTTTGGGCCAGTTAGCTGAGGGAAATTTTGAAATGAGTAACGGTACAG

Protein RF 2: 41->1519 (492AA)

Comparison with *Tribolium* hypothetical protein TcasGA2_TC003675 [Tribolium castaneum] (688AA)

Query 14 LAHSLEDKIRTKDVILQENVSFDSLLLPNNILKGLAKHGFKKPSPIQIKAIPIGRCGFDL 73

+AH L+ K RTKDVIL EN+SF SLLLP++I +GL+ GFKKPSPIQ KAIP+GRCGFDL

Sbjct 5 IAHDLDAKERTKDVILDENISFASLLLPDDIKQGLSVSGFKKPSPIQFKAIPLGRCGFDL 64

Query 74 LVKSKSGTGKTLVFVIVALESIDLSKTRPQVIVVAPTREIAVQIGDVFKDIGVNFDGLVV 133

+VKSKSGTGKTLVF +ALE+++ +K QV+++ PTREIAVQI DV + +G + +GL +

Sbjct 65 IVKSKSGTGKTLVFSTIALETVNTAKDHLQVLILVPTREIAVQIEDVLRSVGCHVNGLKI 124

Query 134 ENFIGGFSIESDKLKCKSCHVAVGTPGRIKQLIKIGSLNVSAVRIFVLDEADKLMENSFQ 193

E+FIGG +E D K CH+AVG PGR+K L+K+G+L + V++FVLDEADKLME SFQ

Sbjct 125 ESFIGGRPLEDDLKKSSKCHIAVGAPGRVKHLLKMGALTTNLVKLFVLDEADKLMEESFQ 184

Query 194 NDINEIYNSLPSKKQVIMSSATYTKELETLLTDYMLSPTYVSAELETPLLLGLKQFVRII 253

+DINEIYNSLP +KQ+I+SSATY +EL+T L +YM SPT+V++E ETPLLLGLKQF ++

Sbjct 185 SDINEIYNSLPPRKQMIVSSATYPQELDTFLANYMQSPTHVTSENETPLLLGLKQFAAML 244

Query 254 KSSNNVVQQTKVKNEELLKIFTLIPFTQCLVFSNYQTRAESISNFLNRNSWNSLYISGAQ 313

+ N VQQ K+KN+ L+ I T + F QCLVF+NYQ+R E++SN+LN+ W+S++IS AQ

Sbjct 245 RPGLNSVQQMKIKNDLLITILTKVSFVQCLVFTNYQSRTETVSNYLNQKGWDSVFISAAQ 304

Query 314 NQTDRLAALSDLKQFHCRILLSTDLTARGIDAANVDLVINYDVPIDAMTYLHRMGRAGRY 373

QT+RL A+ +LK+F RILLSTDLT+RGIDA NVDLVINYD+P DA+TYLHRMGRAGRY

Sbjct 305 KQTERLEAIDNLKKFKNRILLSTDLTSRGIDAPNVDLVINYDLPCDAVTYLHRMGRAGRY 364

Query 374 GSSGICINLASEGPEVQLLQNILGVIGGTNLSVPKLD--DSFNLKEVSNENL--LFGVVP 429

GS G+CIN SEGPEV LQ+ILG IGG NLS+ KL + +L +V + L + GVVP

Sbjct 365 GSGGLCINFVSEGPEVTKLQHILGAIGG-NLSIAKLPPLEGVDLWQVDLKTLEQIRGVVP 423

Query 430 ENS--DVFDQFKAEVRKLKAKSAKKKNKSDKIKDGSDTVKE 468

++ DV + K+EV +LK + KK K + D KE

Sbjct 424 SDTSNDVSENLKSEVMELKERKDGKKRKRVNPEASPDLQKE 464

Graphical representation


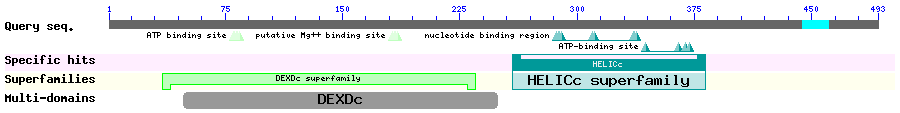


**Similar to gawky CG31992-PA**

>Cp.comp40223_c1_seq8 len=5663

CGTTTTTGTGGCTTTCGTTTCAAATTTCAAGTACCAAAATTAATTGAAACGTTATGGGTTGTTTCGATACGAATCGACCCTCAAAGTGAAAAATCTTCGGCGCCCGCCTCACACGTAAGCGCGACAAGAGCGACCGTACCGGGATACTCGCAACACATACAAACTTAGGTACTCGTTTAGGTTTTATACGAATAAACAAACAAAAATAACATTAATATAATATGAAAATCGTTAATATATAGGAAAATATCTTAATACGAGAGAAAATCTACGCTACGAATTTGAAATATATAAAAAATGTAATGAGTTTGGTCACATATATAATATATATTCAAATATACAATATATTACTATAAACCTTGCTTAATCGGGACTAGTTTGGGTTACAAGTTCATTACTTTCGTTTGCCACGTGTCGCGTGGGTGCGTTTCGAAAATTGAGCGCGAAAAAATCATAGAAATCTCCTTCGAGTCGCTTAAAACAAGTGTTGACGCAAAAAAAGTTACACCGTTAATACTTCCTCTGCCCGACGATTAGCTAAATATACACTAGGTCCGTAAAAAAAAAATAAATGGCAACAACTCCCGTAATTGAAGGAAAAAAAATTAAATATCAACTCTTCCAAAACATCGCTTCCTCAAAAACAATCTTTTCAATTAAATAGTACGATAAATTATTGATAAGTAGTTGTCAATAGAACAATATCACACAGAACAGTTTCGCTTATATAAATTCAATTTTTCAATCCACAATATACAGTGAGTCCACGGATGGAGTGAACAATTAACGAAGTAATCAAACGTTTCTATGGAAAAACTCTTAATAAAAATGACTGTCGTCGGTATCTTTCACTTTTAAGTGTACCTGCCCGGCTGCCCAGTCCACCCGTGGTAATACCGCGCACGCGCGCCCGATTTACTGTCTTCAAAATCGCCGCAGATTGTATTTGAGGCGCGTCAAACGACAAGTGTACATTCGGCCTAACCTTTCGTAGTTTGCAACTTAGCAGCAATAAAAAAATAAACAAAACTCCCTAAAGACGTCACCGTAAAAATTACCACCTACCGTACGTACAATACAATAAATATCAAAAATTTTATAGTCGATCGATAATAATAATTAAAAAAAAAAAAACATCTGATAAAATTTTTCCCATCAACACTTACTTTATAGGTTTTTTTAACTCCATAATTAATTAGTCTCTCAAATTTTTGTTCTTTTCATCCAAGTAATATTTACATAGACTCACCGCCCAACAAGTCGTTGGGTAAAAAAGAATTAAGGCTCGAAGGTGTGGCCCGGGCGGGATCGTTCGAGTCCAGCGACGGTGTCGCCCACAGACCGACTCCGGACGGGTTATTCGGCCAGCCTGTACTCCAAGTGTCGCCGCCGGTTACGCCCGGTTTAGACGACGACGTTCGCCATCCGGCCGAAGAGGCGCCCTGCTGATTGGCGATGCTCTGCAGTAAAGTGCTGGCGTCCCAATCGGTCGGGTTTTCCGCCAGTATTGTTGTGTTGCCGAGTACGCAATTGTTTAAAGCGGTCTGTGCTTTGGTGGCTTCCTCGCGCGACGAATACTTGGCGAGCGCGAAACCTTGGTGGAGGTGTAGATGGAAACTTTGCAACGGGCCGTGCTGCATGCACAATGTGCGTAACGTTGAACCGTCGATCTGGGCCGTCAAGTTCCTGAGCAGCAACCATTGGGACTGCGAAACGCCCCAATTGCCCGAGTTACGTTGAGCGGCACTGCCGGACGCTCCGGCCCACGGTACCGAACCGGCTATATTGCTCGACCATCCGTTGGCCAGGGAAGCTCCGCCGCCCCCTTTACCCGAGAGACCGGGCGGGGGACCGCGAGACTTGGGCGCGCCCCACAGTTCCGACGTGACCGCCATCGAGGGGTTAAGATCGCCGAGGCCACCCTTCGAGGTAGGCAGTTTGATTTGAGGGCTGCTAGTAAAAGCACTGGAGGTAGAAGTGGGGTTGAAGCTCCATGTGGACGAGCTGAGACTCAGCGACTGCATCGCGTCGCTAACCGGCGGGCTCTTGCTCGTGTTCATGCTGAAAAGTTCGTTATCCTTGATCGTGGCGATTGACAATGGCGAACGCACCACCGAACCGGGCGTAATACTGGGGTCGTCTTCTATTTTAATCTGATTGCCCTTCCAGGGTTTTCCGGGTTCAAATTCAGGTACAAGATCAGTCAGGGAAGGTTGAGCAGTCGTTGCCCACTGTGCGTCTTTGTCGTTCGAAGAATCGCCGCCGCTCGAATCCGGCCAACCGGTATCTGCTGTACGACCCGTCGACCATGGTCCGTCAGGTTGAGTCAAACCGAGCGGAGCCATGTTGGGGGAGGTAGCTACGGGCTTGGAAGATGAGCCTGGAGCTCGGCTAAATTCCCCGTTCTCTTCCTTGTCTTTGTTTATCCACTGGTTAAGTCTCGATTGCTGCTGATTCACTTGGGAATCTTTGGTGATGGCGAGTTCGCCAAAGTTGCCTTGAAGGGCGTGTATCGTGTCGTGCATCGTGTTCGTCTTGAACGTATCGTAGCCGATGTTGCCGTTGTTCTGCTGCTGCTTGACGTAAAGCGCCTGTTGAGCTTGAATTTGATTCTGAATATTCAAAATTTGCTGCTTGGTTTTGGTAATCATAACTGAATAATTCATGAGGACGTTATTGGTCAAATTCCCTTTGCCCGAGGCGGCCGCAATCGGGTGCTGGTTATTCAACTGTTGCAAATTCTTGATTTGCTGCAAAAGCTGATTCAAAAGGACCAAAGTTTGAGGCGCTAAAGGTTGATTAAGAATCTGGTGATTCAAATAACCCGCCTGAACTGCCATCTGTATTTGTTGCACGAGCATTCTAAGCTGCTGAGTGGATGGCTGAGACTGGGGCTGCAGAGGGCGACCTCCAGTGGGGCCGCCACCGCCGCCGCCAAAACTTTGGGCCGCTCCACCGCCGCCGCCCTGCTGGGTCAACATTTTTTGCATGATGGCGGGCGAGATGTTGTTTATCAGAGAACTGTTCGATTGTCCGCCGCCAGTTCCGGGCATGTTGTTCAACAAATTCGGCGCGTTGTTCGGCGGAAACGCGTTCGATAACGGATTCGTCGGTCCGACGTTGGGAAAACCGCGTTGCGCTCCCATACCGGGAAACTGGGGATGATCGTAATGGTCGTCGTGACGCGTGATCCAACCGGGATCGCCGCGGTTACGCGAAATGCTTAACATGTCCAACGCTTCGTCGAAGTTCATTTCGCGCATTCGTAGGGCGTTCTCTACGTCCTCTTTCTTATGACCCATATCAACCAGCGTCCTAAATTGCTTAGAATTCCAGACCATTTCTTTAGTGAGTTGAGGTGGTTTGATACTCTGTTTATGACCCCAGTCCAAATCGCCGGTGTCCCATAGTCCGCCGCCGCCACCGCCAGCGGCCCCGGGAATTTTCTGCTTAGGCCATGGACCGACGGTCGGGTCGTCCCAGCCGCTGGCAGCGGTCGGTCCGGCGGCCACGTCGTCCCAACCGTTGCCGCGACCGACGCCGGCGCCGCCGGCCGGACCGCCGCCCCATACCGAACCGTCGGGTTTTAGGCCGCCGCCACCGACCCTAGACGGGGCCATGCCGGGCGGACCGCCGGGACCGCCTCCGCGTCCTCCCATGCCGCCCGCTCCTTGTTGCGGGTTGCCCCACAAAGAGGTTCCGTCGTCGTAGTTGGGCATGGTACGTCTCTGCGATGGCGGGGAGGGTTCGTCCCAGGCGGTTCCGTTTCCGGGACCCACGCCCGGCATCTTGTTTTGGTGGTGCATACCGGCCGGACCTCCAAGCGGTCCGGTTTGGCCCCAAAATTCGGCTCCGGCGCCGTTCAATCTGCCCGTTATACCTCTCGGATCTCCTCTCATGTCGCCGCCTCCGGTCATGTGTCTCATTTGTTCGCGCGGATCCAATTGCCTGGGATCCAGATTTCCGGCCGCGGCGGCCACTCTCATATCGGGCCGCATGTCCATGGCGGACACCGCCGCCACCGCCGCCCTCGGGTCGGCGGACCGCGGATCGCCCCAACCCGACGTCGGGGCCGCCCCTCCCGTTCCGGCACTCGCTGCCGCCGCACCGCCCCACATAGCGTTACTGCCGTTGTTTCCCCATTGTTGCTGATTGGAAGGTACACCGGTCCATACGTTGGAACTGTCGGCCGCGTCATCGTCTTCGCCCCAAGTGCCGCCGATATTCGTCGACGGTGTATGACCCCAGGGAGTTTTCTGTTGGGGTTGCGGGGGCGGCTGACCGCCGTTGCGCAAATTCGCCTCCCACAACTCGGTACCGTTATTGACGGCCGGTTTCCACGGAGGCGGACCGCCCGCGCCGTCCATCTTGATTTGCGGCTCAGGACTGGACGGTATGTCCCAAATGGTGTCCTGGTTGACGTGTTGCCCGCCCCATCCGTCTTGGCTGAATATGGCCTCTCTCATGTTGTTCAGTTGCTCGAGTTGTTGTTTGGTCGATGTCGTTGTATTATTTTGGTTGTTGTTGTTTGGTGTCGCGTTATTAGTAGAAGCGCTCGAGGTGGCCGGACCGTTAGTGCCAGTCGGCGGTTTGGCGCCCGGTTGACCCCAACCCCCCGCGGTATTCGACTGAGTTGTCGGGGGTTGTTGTCCGTTTGTATTCGATTTGTTGGCATTGTTATTACTGGCCTGTTGAGCCGGTGGTGTACCCCAACCGGAGGTGCCCGTGCTAAGTGAGCTCTCGCCGCCACCTCTGAGTCTGAGGGGAACGTGACAACCCTTTAAGCTTTGGTCGTCTTCGTTAGTTGTGCGCAATTTTTGCATCTTGTTGCACAAATAATCATCATCAAGTTGTTTGTCATAATCGTTGTTATCGCAGTAGTTAAAAGCATTTACGTTAAGCATAAATTTCAGTGCACTGAGGTCGTCGAGTACTTCGAGTTCTTCAGACTTTTGGTTGGGAATGTCTCGTCCGAGGGGCAAAGCACCAAACATACTATCGTCTAACTTATCGCTATTTATATTATTATTATTATTATTTAAATTATTGTGGGTGGCCGTGTGACGGCCGTTACGTTGTATTTTTGGTACGGTAACAGAGTTCAGAAGACAGTTATCGGTCACAGATTGCATCTTAACATTAGAGTTGCCGCCTGGCGCGCGCGCGATCGTCGTCGCGTCGGGACGGCGGCAACTTGAGCCGTTTATCACAGACGGGGTGGCGCCACCGGCGACGGGGGCGCCGTGAGGGCCGGTCGGGGGGTCGGCTCGACCCCCCCAAGCTGCCCGAGCGACTTTATGTTGTACTGATTGGGGTGCGCTGCCCCTCATGGCTGACTCTTGAGGCACTTGGTAGGTAGGAAATGTATTAGACTTCGGCTCGGAGGGGGTAGGGGCGCGCATCATCACTAGTGCTCCATATCAGTCTGGCCAGCCAGAGATCTTTTTGTCACAAAATCCGCACAGAGTTGGTGTAATTTATCACACTGAACTAATGAGAATACTTATTATCACAATATTTATTAAACGATTTGGCGTTAAAATACTCTTGCATTTGCGGACACCACAGATTTTTGTTTATCGATCCGCGATATTACGTGGCACTGTGGCAGCGTCGCCGCACAATAACCGACTACGCGACTCGGCCGGTGTCGGAGATTTACG

Protein RF -1: -5405->-1236 (1389AA)

Comparison with *Tribolium* PREDICTED: similar to gawky CG31992-PA [Tribolium castaneum] (1014AA)

Query 314 MREAIFSQDGWGGQHVNQDTIWDIPSSPEPQIKMDGAGGPPPWKPAVNNGTELWEANLRN 373

MREAIFSQDGWGGQHVNQDT WDIP SPEP +KMDG+ PPPWKPA+NNGTELWEANLRN

Sbjct 1 MREAIFSQDGWGGQHVNQDTNWDIPGSPEPSMKMDGSA-PPPWKPAINNGTELWEANLRN 59

Query 374 GGQPPPQPQQKTPWGHTPSTNIGGTWGEDDDAADSSNVWTGVPSNQQQWGNNG-SNAMWG 432

GGQPPPQPQQKTPWGHTPSTNIGGTWGEDDDA DSSNVWTGVPS QQQWGN S MWG

Sbjct 60 GGQPPPQPQQKTPWGHTPSTNIGGTWGEDDDA-DSSNVWTGVPSGQQQWGNTANSGGMWG 118

Query 433 GAAAASAGTGGAAPTSGWGDPRSA-DPRAAVAAVSAMDMRPDMRVAAAAGNLDP-RQLDP 490

G S G AA GWGDPR+A DPRA + DMRPD+R AG+ DP R LDP

Sbjct 119 GPKKESE-WGAAAGNPGWGDPRTATDPRA-TGGIDPRDMRPDLR-DMRAGSSDPMRLLDP 175

Query 491 REQMRHMTGGGDMRGDPRGITGRLNGAGAE--FWGQTGPLGGPAGMHHQNKMPGVGPGNG 548

REQMR G DMRGDPRGITGRLNGAGA FWGQ GP G +HHQNKMP VGPGNG

Sbjct 176 REQMR--LAGSDMRGDPRGITGRLNGAGAADAFWGQAGPHTGTQHIHHQNKMP-VGPGNG 232

Query 549 TAWDEPSPPSQRRTMPNYDDGTSLWGNPQQGAGGMGGRGGGPGGPPGMAPSRVGGGGLKP 608

W+EPSPP+QRR MPNYDDGTSLWGNPQQGA MG G GPPGMA SR+ KP

Sbjct 233 AGWEEPSPPTQRRNMPNYDDGTSLWGNPQQGAS-MGR--GSTAGPPGMAQSRI-----KP 284

Query 609 DGSVWGGGPAGGAGVGRGNGWDDVAAGPTAASGWDDPTVGPWPKQKIPGAAGGGGGGLWD 668

DGSVW GR WD+ GP GWD+ +VG W KQK+ G LW

Sbjct 285 DGSVWC--------HGRNGSWDET--GP----GWDE-SVGGWNKQKMAGTH------LWG 323

Query 669 TGDLDWGHKQSIKPPQLTKEMVWNSKQFRTLVDMGHKKEDVENALRMREMNFDEALDMLS 728

++DWGH + K LTKEM+WNSK FR L+DMG+KKEDVE ALR +MN+++AL++L

Sbjct 324 DNEIDWGHNKGPKQ-NLTKEMIWNSKCFRMLMDMGYKKEDVETALRRGDMNYEDALEILG 382

Query 729 ISRNRGDPGWITRHDDHYDHPQFPGMGAQRGFPNVGPTNPLSNAFPP-NNAPNLLNNMPG 787

+R GW RHDDHYDH QFPG QR FP+ GP +S FP NNAPNLLNNM

Sbjct 383 ---SRNPDGWRNRHDDHYDHQQFPG---QR-FPS-GPPGQMS--FPQGNNAPNLLNNMNS 432

Query 788 TGGGQSNSSLINNISPAIMQKMLTQQGGGGGAAQSFGGGGGGPTGGRPLQPQSQPSTQQL 847

+GG N+SLINNISPA + KMLTQ GGG + GR LQPQSQPSTQQL

Sbjct 433 SGG--PNNSLINNISPAGVHKMLTQGGGGSQGFGAVS------AAGRNLQPQSQPSTQQL 484

Query 848 RMLVQQIQMAVQAGYLNHQILNQPLAPQTLVLLNQLLQQIKNLQQLNNQHPIAAASGKGN 907

RMLVQQIQMAVQAGYLNHQILNQPLAPQTL+LLNQLLQQIK LQQL Q +A +

Sbjct 485 RMLVQQIQMAVQAGYLNHQILNQPLAPQTLILLNQLLQQIKTLQQLMTQQSVAQSQCING 544

Query 908 LTNNVLMNYSVMITKTKQQILNIQNQIQAQQALYVKQQQNNGNIG---YDTFKTNT-MHD 963

N+ L+ SV+ITKTKQQI N+QNQI AQQA+YVKQQ N+G+IG D FKT MHD

Sbjct 545 KPNSALLQCSVLITKTKQQITNLQNQIAAQQAIYVKQQ-NHGSIGGGQSDLFKTAAPMHD 603

Query 964 TIHALQGNFGELAITKDSQVNQQQSRLNQWINKDKEENGEFSRAPGSSSKPVATSPNMAP 1023

+I+ALQ NF +L I KD QVNQ QSRLNQWINKDKEE GEFSRAPGSSSKP+ATSPNM P

Sbjct 604 SINALQSNFADLGI-KD-QVNQSQSRLNQWINKDKEEGGEFSRAPGSSSKPLATSPNMNP 661

Query 1024 LGLTQPDGPWSTGRTADTGWPDSSGGDSSND-KDAQWATTAQPSLTDLVPEFEPGKPWKG 1082

LGLTQPDGPWS+GRT D GWP+S GGDSSND KDAQW T QPSL+DLVPEFEPGKPWKG

Sbjct 662 LGLTQPDGPWSSGRTGDGGWPESGGGDSSNDGKDAQWPTPTQPSLSDLVPEFEPGKPWKG 721

Query 1083 NQIK-IEDDPSITPGSVVRSPLSIATIKDNELFSMN-TSKSPPVSDAMQSLSLSSSTWSF 1140

NQIK IEDDPSITPGSVVRS LSIATIKD ELF MN +KSPP D +Q LSLSSSTWSF

Sbjct 722 NQIKSIEDDPSITPGSVVRSSLSIATIKDTELFQMNPNNKSPPAGDTIQPLSLSSSTWSF 781

Query 1141 NP-TSTSSAFTSSPQIKLPTSKGGLGDLNPSMAVTSELWGAPKSRGPPPGLSGKGGGGAS 1199

NP +STSSAFTS PQ KLP+SK GLG+LNP+ AVTSELW APKSRGPPPGLS KGG +

Sbjct 782 NPPSSTSSAFTS-PQNKLPSSKSGLGELNPTTAVTSELWAAPKSRGPPPGLSAKGG---A 837

Query 1200 LANGWSSNIAGSVPWAGASGSAAQRNSGNWGVSQSQWLLLRNLTAQIDGSTLRTLCMQHG 1259

L NGWSS AS QR SG+WG S WLLLRNLTAQIDGSTLRTLCMQHG

Sbjct 838 LVNGWSS---------AASWGGGQRGSGSWG--GSPWLLLRNLTAQIDGSTLRTLCMQHG 886

Query 1260 PLQSFHLHLHQGFALAKYSSREEATKAQTALNNCVLGNTTILAENPTDWDASTLLQSIAN 1319

PLQSFHL+LHQGFALAKYS+REEATKAQTALNNCVLGNTTILAENP++WDA+ LLQ +A+

Sbjct 887 PLQSFHLYLHQGFALAKYSTREEATKAQTALNNCVLGNTTILAENPSEWDANALLQQVAS 946

Query 1320 QQGASSAGWRTSSSKPGVTGGDTWSTGWPNNPSGVGLWATPSLDSNDPARATPSSLNSFL 1379

QQ +S A WR S+ +P TG DTWSTGW N+ S LW + +LD+ DPARATPSSLNSFL

Sbjct 947 QQSSSGA-WRGSTKQPS-TGSDTWSTGWSNSQSSASLWGSTTLDTTDPARATPSSLNSFL 1004

Query 1380 PNDLLGGESM 1389

P DLLGGESM

Sbjct 1005 PGDLLGGESM 1014

Graphical representation


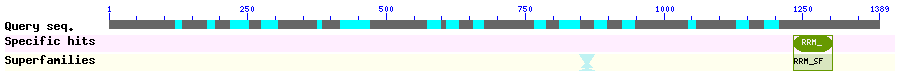


**Staufen**

>Cp.comp28896_c0_seq2 len=3714

GTTGACTCCCTTTTCTACACCTCCGAGACTCAAAAATTGGCAATGAATTTTCTTGCCTACTTTTATATATTTTTTGTACCCAGTTTAATAAAATTCTACCTAAGCGGCAACGCGAAATTTCCGTGCAGCAAAAGCGAACGAGAAAATAATTCGAATTTTCGCGCAGGCTTAATGAGCTAAGCGCACAGTGCTGCTGCTAGCGAAAGCGGTTAGCTGTGAAAGAGCGAGAAGAAAATAGCGACATTAGCGATCGCTATGCGTTGATTTCATTTATAAAATCCATCGTCGTGATATTTCTAACATGGCCGCATGTTAGTTGCCGATTTTTTTCTTTATTGTTTGCCAACGAACGTATAACATTTAGTAGTGGTGTTTGGTTAAAAAAAAGCAAGATTTGTTGTTAAGGATTAATAAAACGTATTGAGATTCGACCGTATTTCGATAAATAAATCGCCGACGCCGGTTGAATAGGTGCACTCCCAAACTTGTAGTCAGATTTTATTTTTTAATCGTTTCGCGTCTATTATCATGATTCAAGAGCATCACATTAATCAAAACGGCATGCAGAGAAATCCAAGAATGGGAATGCAGCAAATGGGGCCACCTCCAAATCAACGAACTCCTATGATTATGAGCATGCCTGCAACTGGGGTGCTTGTCTCAATGTCTCCCGGGCCAGGACCCGCTCTTATTACTACAACTTCAATTCCCCAACCCCAGCCACAAGCTAAGCCAAATCTTCAACAGCCTCAGGTTTATCATTACGAAGCACCACCTACACAGCCCCCTCCTCACACTCAACAACCAGAGCCAGATCAGTCACAGCAACAGCTGATGAATAACCAAGATCAACAACAAATGAATCAGAACTGTCCAAACACGAGTACTTCTTCGACGCCCAACACGACATTGGCAAACATAAAAGAAAAAACACCCATGTGCTTGGTGAATGAATTAGCACGGTACAACAAAATTCAACATCAATATCGGCTGACAGGAGAACAAGGTCCTGCACACAAGAAAATATTCACAGTTACCTTGAAACTGGGTAAAGAAGAATATGAAGCCGAGGGCCCCAGTATCAAGAAAGCCCAACATTCTGCAGCCGCTAAAGCATTGACCAAGACCGAATTTAAGCATCCGCCACAGAAAACAATTATTAACAGGCCCGGAAATAGGCCCTCGAATCCAGGTGTCGTCACTCCAACGGTCGAACTCAACGCTTTGGCCATGAAACGAGGAGAACGTACGGTGTATGTTGTTGAAAGTGGAGGGGCACCACCTCATCAGGGCTATATGAATCAGCCGAGTTACTATCCACGGCACAACATCAACTACCCTCAGGCTCAACCCCGCTATGGATACGATGCCCGGCGTAATATCAGGGGTCATTACACTTATGACAACCGCTATTACGGCCAGTTTAGGCCTGGGGCTCCGCACCATAATCCGGGTGACCCGTACACTGTACGGCTGAGAGTTGGTGAACGAGAGTATCCAGGTCACGGATATACAGTCCAAGCAGCTCGGCATGACGCCGCCGCCAAAGCGATCGAGCATATTAAACAGCTTGGCGGTGTTGAAGGTGCCGACAGTGTACCAGCAAACGAGAACGGTCAAATGAGCGACCAGCAACAAGCCCAAAGTTCGTTAACCAATGACGTGAACTCTGAATTAAAATCTCCGATTTCATTGGTTTACGAGATTGCTCTTAAGAGAAATCTGAATGTCACTTTTGAAGTTCTGAGCGAAAAGGGTCCGCCGCATATGAAAGTTTTCGTAACTCAGTGTAGGGTTGGTAGTTTTGTAGCAGAAGGGGAAGGCAATGGGAAAAAGATTTCCAAAAAAAGGGCCGCCGAGAAAATGTTGGAAGAGTTATCCAAATTACCACCCCTTCCTAATGTGGCCAATATATCCCAGATGAAACGCAAGAGGGTAACGAACAAGAAGAAAACAAGGAATCTTATTAAAGTTAACGTTGATAAGAACTCCGAAACCCAAGAGGAAATTAATCCGATCTCCCGTCTTATACAAATTCAGCAGGCTAATAAAGAGAGAGAACCGGTTTACACAGTCCTGGAGGAACGCGGAGCACCTCGCAGGCGAGAATTTGTTATAGAAGCTTCTGTCAGTGGCCATTCTTGCACTGGAGTAGGTCCCAACAAAAAGGTCGCAAAGCGTAATGCAGCAGATGCATTGTTGACCGAACTCGGTTACAGCACCACCGTACCCAAACCTCAGACTCAGACCAAAAGCGAAAAAGAACCAGAACCGTCGACCAGCAGTGATAAAAACCGTAAAGTCACTTTCGTGGAGGAGAAAGCCGAACAACCGCCCACTCAATCCATCGGAGGCAGCGGCGGACGTCAACTTGTACCCGGTGTTCTGTTAGTTACAGAACAAAATGCCGGTTTTAGCAAACCGAAAACTGAAAACACTGAGCAAAACAAACCCGTGCAAATGCAACCTGCTCCGATGAAAAATCCTCCGCCTGGAGTTAGATCCAAAGATCAATTGTTGTACCTGGCTCAGTTGATGAACGTTCATGTTCAATTTTCAGATTTTCCAAAGGCAAATCATGAAATGTATTTGACCCTAGTAAGTCTGGGAACAAATCCGCCTCAAGTTTGCCACGGCGAAGGTCCGACAACGGAAGCTTCGCACGAAAAAGCCGCTTTAGAAGCGTTGAAGGTCCTTTCCGAGTTGGGCCTGGACATTGCCCCTAAAGAAGGCAATGGCCAAATTGAAAAGTAAAAAAAGGGTTATTTGTCGCGTTTGTTTTCTTTCGTACAAAAGAAAAATTGTTTATTAATTTGTTTTATTAGACAACTGAAATGTTACACGTATTACGATTCGAACTGAGTTAATAGTATTAGTACGGATTTGTTAATTCAATTATTTCAAGACGAGTTGCGTCTTGCATTGTATTTTTGTTTTATTTTTTTGGACGGAATGAAACAAGATTATAAGATTATTAACTTTGAACTCTTTGTAGACTATACCAATGAATGTGGCTATTGATCACTATTTAATTTATTCTGTTGTCATGTGTGAGTGATATTTGCAGAGTCCTTTTTCATTGGTACAGAACACATTTACTACTGGGAGTTCCATGTGGCCATGTCAAAGAAAAAAAAATTAATATAAAGTTGCATTATTCATTATACTTGGATAACTTAGAATTATTATTAATCTCATATTTATGTGATGTCATTAACATATGTAATGATTTCATATATGACATAATTCTGGTTATTGCTTGCACTGTATATTTAAATTTAAAAAAAAATAAGACAGTGTTTACCGTATTTCGTTTTAGTTACAGGGCGACCGAAAACGAAATCGGGTCCAGTAGTTCAGAATTGATTTGTTAAAATTACTCTACTTGGACAAGTAATTCGAATTTTAATTGTCTTGCTATTTTATTAAACCGGATTGTGAATCGAGCTTAAAGGCTATGTTCACAATTTTTCCTCGTCCTTCTAGATGTAGTGTTAAACGCTTGACTTCTTCCATAAGACAATTCTGATCTGCTGAAATGTATTAATTTCACTTATTGTTTGTAGATTGATTTCATTATTCCGGGGCCTTTGGTATTCTTTCTTTTTGTTTGTTTCTGTTGTAGTATAAGAGTTAGACACTTCTATACCTTGGATAAGCATGCTAGCCTCATTTGAATTTGAAATCAATAAACATTATCACGTAAAAAAAAAA

Protein RF 1: 529->2754 (741AA)

Comparison with *Tribolium* staufen (724AA)

Query 18 MGMQQMGPPPNQRT-PMIMSM--PAT----GVLVSMSPGPGPALITTTSIPQPQPQAKPN 70

MGMQQMGPPPN R+ PM+MS+ P T GVLVSM PGP LI+T SIPQ QPQ K N

Sbjct 1 MGMQQMGPPPNHRSQPMLMSIQPPVTAAHAGVLVSMPPGP--PLISTPSIPQ-QPQPKNN 57

Query 71 LQQPQVYHYEAPPTQPPPHTQQPEPDQSQQQLMNNQDQQQMNQNCPNTSTSSTP-NTTLA 129

+QQ VYHYE +T E +Q+Q + Q +Q PNTSTSS P +TTLA

Sbjct 58 VQQ--VYHYE--------NTASSETEQAQH------NDTQQHQTGPNTSTSSAPPSTTLA 101

Query 130 NIKEKTPMCLVNELARYNKIQHQYRLTGEQGPAHKKIFTVTLKLGKEEYEAEGPSIKKAQ 189

NIKEKTPMCLVNELARYNKIQHQY+LT E GPAHKK+FTVTLKLG EEY++EGPSIKKAQ

Sbjct 102 NIKEKTPMCLVNELARYNKIQHQYQLTEESGPAHKKVFTVTLKLGNEEYKSEGPSIKKAQ 161

Query 190 HSAAAKALTKTEFKHPPQKTIINRPGNRPSNPGVVTPTVELNALAMKRGERTVYVVESGG 249

HSAAA++L KTEFKHPP KT NRPG R +NPGV+TPTVELNALAMKRGER VY+VE+

Sbjct 162 HSAAAQSLAKTEFKHPPSKTARNRPGTRATNPGVMTPTVELNALAMKRGERPVYIVEN-- 219

Query 250 APPHQGYMNQPSYYPRHNINYPQAQPRYGYDARRNIRGHYTY-DNRYYGQFRPGAPHHNP 308

PPHQGY++Q YYPR NI Q QPRYGYD RRN+R HY Y +NRYYGQ+RP PH NP

Sbjct 220 PPPHQGYISQAGYYPRQNIYGTQNQPRYGYDTRRNMRPHYPYHENRYYGQYRPTGPH-NP 278

Query 309 GDPYTVRLRVGEREYPGHGYTVQAARHDAAAKAIEHIKQLGGV---EGADSVPANENGQM 365

GDPYTVRLRVG+REYPG GYTVQAARHDAAAKAIE IKQLG G P G

Sbjct 279 GDPYTVRLRVGDREYPGVGYTVQAARHDAAAKAIEDIKQLGDCTDQSGTVEAPVENTG-- 336

Query 366 SDQQQAQSSLTNDVNSELKSPISLVYEIALKRNLNVTFEVLSEKGPPHMKVFVTQCRVGS 425

+ND+N+ELKSPISLV+EIALKRNL+VTFEVLSEKGPPHMKVFVTQCRVG+

Sbjct 337 ----------SNDINTELKSPISLVHEIALKRNLSVTFEVLSEKGPPHMKVFVTQCRVGN 386

Query 426 FVAEGEGNGKKISKKRAAEKMLEELSKLPPLPNVANISQMKRKRVTNKKKTRNLIKVNVD 485

FVAEGEGNGKKISKKRAAEKMLEEL+KLPPLPN+ N+ +KRKRVTNKKKTRNLIKVN+D

Sbjct 387 FVAEGEGNGKKISKKRAAEKMLEELAKLPPLPNMHNMVHLKRKRVTNKKKTRNLIKVNMD 446

Query 486 KNSETQEEINPISRLIQIQQANKEREPVYTVLEERGAPRRREFVIEASVSGHSCTGVGPN 545

K+SE EEINPISRLIQIQQANKEREPVYTVLEERGAPRRREFVIEASV+GHSCTGVGPN

Sbjct 447 KSSEFTEEINPISRLIQIQQANKEREPVYTVLEERGAPRRREFVIEASVNGHSCTGVGPN 506

Query 546 KKVAKRNAADALLTELGYSTTVPKPQTQTKSEKEPEPSTSSDKNRKVTFVEEKAEQPPTQ 605

KK+AKRNAA+ALL +LGY P+ K+EKE E DK RKVTFVEEK E P

Sbjct 507 KKIAKRNAAEALLNQLGYGNPPSNPKPVLKTEKESE---IGDKTRKVTFVEEKTESTP-- 561

Query 606 SIGGSGGRQLVPGVLLVTEQNAGF--SKPKTE--NTEQNKPVQMQP---APMKNPP---P 655

S+GGSGGRQLVPG+LLV +Q+ F +KPK N N V + MK PP

Sbjct 562 SVGGSGGRQLVPGILLVGDQSTNFQQNKPKESVMNNNSNSCVDTKKTGQTQMKTPPQPIQ 621

Query 656 GVRSKDQLLYLAQLMNVHVQFSDFPKANHEMYLTLVSLGTNPPQVCHGEGPTTEASHEKA 715

GVRSKDQL+YLAQLMN+ VQFSDFPKANHEMYLTLVSL TNPPQVCHGEGPTTEASHEKA

Sbjct 622 GVRSKDQLMYLAQLMNIQVQFSDFPKANHEMYLTLVSLSTNPPQVCHGEGPTTEASHEKA 681

Query 716 ALEALKVLSELGLDIAP--KEGNGQI 739

ALEALKVLSELGLDI KEG +I

Sbjct 682 ALEALKVLSELGLDIVGPNKEGGNEI 707

Graphical representation


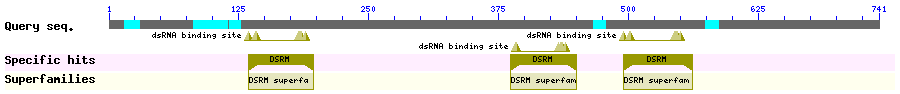


**Clp1 homolog (kinase)**

>Cp.comp34766_c0_seq1 len=2376

TGTTCTAAGCGTGTTTTGATTTTTTACCGTAACCTCTAAAATAGATTTGTTTAGTTTTTTTATATATAAATAATGGGCGAAGATAAAAAATCTCTCCTTCAAGAATTTAAACTAGATCCGGATAATGAGTTGCGCTTTGAGGTGGAATCAAAAAAACGAAAAAGTTTACCTTACACTGAAAAGCGGTCTAGCGGAAGTTTTCGGTACGGAATTGGTAAAAGGCAAAACGTACGAGTTCGTTTCCGGGGCAAAAGTCGCCGTATACACATGGCAAGGATGTACGATCGAAGTCAAAGGAAAGACCGACGTAATTTACACTGCCAAAGAAACTCCCATGGTAATTTATTCAAATTGTCATGCAGCCCTGGAATTGATGAGAATCGAGGCCGAAAAGGATAACAAAAGAGGACCTATCGCTATGATTGTAGGCCCTTGCGACGTTGGCAAATCCACAGTATGTAGAATATTGTTGAACTACGCTGTCAGGATGGGACGTAGACCTATTTATGTCGACTTAGATGTCGGCCAAGGATCTGTTTCTATTCCGGGGACAGTAGGTGCATTAGTAATAGAGCGACCTGCAGGAATAGATGAAGGCTTTTCGCAGGAGGCACCATTAGTTTATAATTTTGGACACAAAAGCCCAGGACATAATACAAAATTGTACAGAATGATTGTCGACCAATTGGCAACGATTGTCAAGGAAAGACTTGAAGTTAATAAGAAAACTCGACAATCTGGAGTTATAATTAATACATGTGGATGGATCAAAGGAGAAGGTTATAAGCAAATACTGCAATCTGGAAAATCCTTCGAAGTTGATGTAATAATGGTGCTGGACCAAGAGCGCCTGTATAACGAGTTAGTCAGAGATATGCCAAATTATGTGAAAATAGTATTTCTTCAAAAAAGTGGAGGTGTGGTAGAACGTTCGAAACAAGCAAGAAGCGAAGCAAGAGATCAGAGAATAAGAGAATATTTTTATGGAACGCCCAAAAATTCACTCTATCCTCACTCGTTCGATGTCAAATTTTCAGAGATAAAGATTTTTAAAATAGGCGCTCCAGCTTTACCGGATTCTTGTTTACCGTTGGGAATGAAAGCCGAAGATCATTTAACGAAAGTTGTTCCTATTACCCCCAATCCGGGTATTTTGCATCATCTGCTGGGTGTTAGCTTTGCAGAAAAAGAGGAGGACGACATCATACTGTCCCATGTTGCTGGATTTGTTTGCGTGTCAAATGTAGATCTTGAAAGACAAACTATCACCTTGTTGTCGCCTCAACCAAAGCCTTTACCAAATAATATGTTGGTATTGTCAGAAATTCAATTTATGGATAGTCACTAGCAAATAGTTTAGTTTTGTTGTTAAAATGCTTTATTTTTCAATATACTTTATACAAGATTTGTTGATATTATTACCTAAATAAATCCTATTATGCAATCTTTGTCAAAATGTTGCTGACATGACTTTGAAAGGACCAAAAACCAATTGGTTTGGCTACATTCATTTTTACATGATAAATTTTTCATATTGCTAATATTTCAGTACATGTCTGTACTGAATATAAATTCCTTATACCTATTTCCTGTGAAAAAAATAAATTTTGCATAATTTGTTAAAAAAAACATATATTAAAAATACTAATAAATAGCGATCACAGTGTTAAATTTCATTATGAAAAAGCAAAATTTATTACAAAAAAAATTAAACAAACATTTTAATCCAAAGTGAAAATTCTAGCAGCAATATCGTTAACCAACCAGTCTATACCAGTTAATAAATTTTCACTGGTAACTGCACTACACGAGACAATCTTCCAATGATGAGTTTTAATTTTGTCCAGTTCAAGAATCTCTTTAAGTTCGTCCATATTACATGCTCCAGGCAAGTCTTGCTTATTTGCGAATACAAGTAACGTCGCACCAGCCAATCTCTCTTCTTGAAGGAGTACATGAAGTTCTTTCTTACAATCTTCAAGGCGTCTTTTGTCTGCACTATCGACCACCCAGATAAGTCCGTCTGTGCATTCAAAGTAATTCCTCCAGTAAGATCTTAGCGATTTTTGTCCCCCTACATCCCACATGTTGAGTTTAAAGCTTCTGTGTTCTAAGGTTTTAATATTAAAACCCAACGTTGGCGAGATTGTATCTATAGGTTCACCATTAAATCTCTTTAGAATTGTAGTTTTGCCAGCATTGTCCAAACCTAATATTAAGATTCTCATTTCTTTTTCTTTTTGCTTCATTTTTTTCAAAACTGTTAGAAACCCCATGTTTATGTTTTTCTAAAATACAAAGTTTTTACAAATAGGTTAAAGCCCATAGATAGGGAGATTGGTTTTGGTTTCGTACTGGGGACTAATTCCAAGATGT

Protein RF 1&2: 73->1348 (425AA)

Comparison with *Tribolium* hypothetical protein TcasGA2_TC009961 (406AA)

Range 1: E=2e-05; bits=47,4

Query 73 MGEDKKSLLQEFKLDPDNELRFEVESKKRKSLPYTEKRSSGSFRYG 210

+ EDKK+++Q+FKLD DNELRFEVESK K Y +S + +G

Sbjct 3 LNEDKKTVIQDFKLDQDNELRFEVESKNEKV--YVTLKSGKAEVFG 46

Range 2: E=0.0; bits=714

Query 152 KNEKVYLTLKSGLAEVFGTELVKGKTYEFVSGAKVAVYTWQGCTIEVKGKTDVIYTAKET 331

KNEKVY+TLKSG AEVFGTELVKGKTYEF SGAKVAVYTW GCTIEVKGKTDV Y AKET

Sbjct 29 KNEKVYVTLKSGKAEVFGTELVKGKTYEFTSGAKVAVYTWHGCTIEVKGKTDVSYVAKET 88

Query 332 PMVIYSNCHAALELMRIEAEKDNKRGPIAMIVGPCDVGKSTVCRILLNYAVRMGRRPIYV 511

PMV YSNCHAALE MRIEAE++NK+GP M+VGP DVGKSTVCRILLNYAVRMGRRPI+V

Sbjct 89 PMVTYSNCHAALEFMRIEAERENKKGPTVMLVGPNDVGKSTVCRILLNYAVRMGRRPIFV 148

Query 512 DLDVGQGSVSIPGTVGALVIERPAGIDEGFSQEAPLVYNFGHKSPGHNTKLYRMIVDQLA 691

DLDVGQG +SIPGT+GAL+IERPA IDEGFSQEAPLVY+ GHKSP N LY M

Sbjct 149 DLDVGQGQISIPGTIGALLIERPASIDEGFSQEAPLVYHTGHKSPQPNIALYSM------ 202

Query 692 TIVKERLEVNKKTRQSGVIINTCGWIKGEGYKQILQSGKSFEVDVIMVLDQERLYNELVR 871

SGVIINTCGWIKG GYKQIL S K+FEVDVI+VLDQERLYNELVR

Sbjct 203 --------------ASGVIINTCGWIKGTGYKQILHSAKAFEVDVILVLDQERLYNELVR 248

Query 872 DMPNYVKIVFLQKSGGVVERSKQARSEARDQRIREYFYGTPKNSLYPHSFDVKFSEIKIF 1051

DMPN+VK++FLQKSGGVVERSK RSEARDQRIREYFYGTPKNS+YPHSFDVK+SEIKI+

Sbjct 249 DMPNFVKVIFLQKSGGVVERSKSVRSEARDQRIREYFYGTPKNSMYPHSFDVKWSEIKIY 308

Query 1052 KIGAPALPDSCLPLGMKAEDHLTKVVPITPNPGILHHLLGVSFAEKEEDDIILSHVAGFV 1231

KIGAPALPDSCLPLGMKAEDHLTK+VP+TPNPGILHHLL VSF+E E++DII SHVAGFV

Sbjct 309 KIGAPALPDSCLPLGMKAEDHLTKLVPVTPNPGILHHLLAVSFSEGEDEDIISSHVAGFV 368

Query 1232 CVSNVDLERQTITLLSPQPKPLPNNMLVLSEIQFMDSH 1345

CV+NVD +RQ +TLLSPQPKPLPNN+L+LSE+QFMDSH

Sbjct 369 CVTNVDTDRQIVTLLSPQPKPLPNNILLLSELQFMDSH 406

Graphical representation


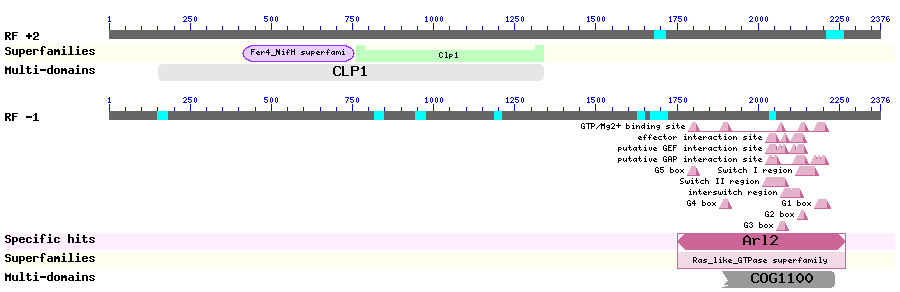


**Elp-1**

>Cp.comp40424_c1_seq1 len=7923

AAGTATTGTTTGATTGTACAATTCTGACTTATTCTAATCTTTATATTTGGTGGAATAGTTAAAAACTAGAACAAATTCTATCAAATAGGCCGAATGACCAAAACAAAATTTATATGAAAAGAACGTGGACATTATAAGAACCTAACCTGATTTTAACCTAAGTAATTTTTTGAGCTGTTTTTGTGTTTTAAAATGAACAATCTTGAAATCCTTTGTTGTGAAGTTCAAGAAATCAAGCGTCACGAAACTGGAAATATAAATAGAGGGATTGTAAACGAAAGAGTTTGCTGTTTTTGGACTGACCGAAATCACTTATACTTCTACGAAGATTTAATTAATCATACCAATCCAGTAGGCGAAAACATCAGAGAAGTTAGATATCTCCAATACCTCTCCATCCACCAAGTGTTGTTTATAACAAACGATTTCGACTTAATTATTTACAATGGAAACTCGAAGGAATTTGTCGTGGTTTACTCTTCTCCTATCAAAATTGAAAGTGTCGCCTGGAACCCTAGTGAAACTTTTTTGATTCTTTGCGACACTGAAGAAAATTTGTATGGATTGACGTTTGAATGCAACATTGGTTTTAAAGAATATAGATTCATTGAAACTTGTAAATCAAACTTGCACGATCCTGTGCCTAATTCAGTTTATGTAGGTTGGGGTTCACACAATACCCAATTTAAAGGGTCCAAAAAGATAATAGATGAAGAAATCCAAGAAATTCCACAACAATATAGTCCACCGATAATAAGTTGGAGAGGCAACGGGGAACAGTTTGTAATAAATTTTTGGAAAGAAGAAAGGAGATACTTGAAGCTGTTTGATAGCACTTTACGACCTCTCTATCAATCTGAGTCTTATCCAAATCTCTTAGCGCTGACATCATTCAAACAACAAGGGAATTTTATAGCATGCGCGACAATTCATAATAATCAAAACAAGATTGTTATTTTTGAGAAAAATTGCTTGGTAAAATCGGAATTTTTAGTTCCAGAAGTCAAGGGGGTAATCAGGAACATTTTATATCACCCATTCCTTCATATATTAGCAATTTACAGCCTCGAAGAGGCTGGTAATGGATACATTAACATTTTCGTAAATTCAAATGCAAAATGGTACTTGAAACAGCAGCTGTTGTATGTGACTGAAAAGCAAGTAAAATGTCTTAACTGGGCCGATAGTCTGGAAAATTCCATATGCAAATTAAATATTCTGACGTCATCCAGTATCGAGCGTATTGTTTTTAGAATGGTCATTAACAGAAGCCCTAATAACGGGACCGTAGCTGTCGTTGATGGTAGCATAGTACATTTTACATTTTTTGACTCTGAAATTGTACCTCCTCCAATGTCAAGTGCCTTTTTGAAATTTGATAAGCCTATAAATAGGATACAGTTTCATCCCAATTTACCACAATGCATTCTAACAGACTCTACTTCAAATGTAAAATTGCTTAATTTCAAAAATAACACTTTATCAGAAATTTTAGAAGATGATTTAGTTGACAAAAATTTTAAACCGGAAACATTCATACCAAATTACCTGTCGTTTAATGATGTCACTGAGATCATGGAGCCAATAAATTTAAATGACAGAGATACTGCATCTTTAGTATATTGTACAGGGTGTAAGATAAACGATAATTTTTACGAGTTTGCACTGAATCGGTCCCAAGATCTGCTGATAAACCGAAACGTAATGTGCAGTAAAGTATCGTCAGTGTTTATATATAAGAGTTATTTGCTTTTTATTCATGTCTCGTGTAAATTGTACTGCGTGAGACTTACATCAGACATTGTTCATGAATCCAGTTGGGAGTTGAAAGATTTTTATTTTAGAGATGTCGAACAAGGTGCAAAAATTGTTTGTGTCAATTCGAGACCCGAGATAGTTCTGGAACTGCCTAGAGGTAATTTGGAGACTATTAAATGTCGTTTAATTTCCATAGATATGGTAGAAGAAATGTTATCCGATGGCAAGTGGCAAGAGGCTGTCAACTTAATCAGACTGCACAAACTGAATTGGAATCTTCTGATAGATTTAAATCCACAGAGGTTTGGCGAGAACATACCGAAATTTATTAAAGCAGCAGAAACGGTATCAATGTTGAATTCGATCGTGTCAGAATTTGGCACTTCAAACTGTCTGACGACTATGTACTCCCAGTGTGTTAAAGATAGGACTTTCCCCGTTCAAAACAAAACTAATAAATTGTTACAAATTTTGAATTACATCATCGATTTAGATCCAATTGCCAATCTGATTAGCATTGTTACTTTGCAAATAAAGCATTTTACTTTGAAGTCGGCACTAAAGTCTGTATATAAAGTATATGAGTTAAATCATTTAGAAATGTGCAAGAAAGCCATATTCCAGATACTGTTGTACTGTCAGACTCCTGCCGTTATAAATATGAGTTTTACTTTGTATAATATCGAATTTGTAAGATTTGTGCATATTTGTTGTTCAGAAGATCCTAAAGTGTTCGAACCACAGTTAGACAAATTAAAAGGCTTGGGAACTGTCGACTTAAGGTTTCAGATGAACGTGCTTGCAAAAAAATTACAAAGGAGCCGTTAAATTTCTCATCAGATGTTCAAATTACCAGGAAAACTATATTAGAGAGTTTATAACGAAACATAACACCTATGAGGAGGTGTATAAAAGCGTGACGCCGAAGAATCCCAACTTTACTTTGTGTTCTCGCCTTTTTGCGACATATTTATCGATGAAGCAAAGGTATAATGAAGCGGGGTTAATATTGCAAAAGGCAGGATTATACCAGGACGCCATAATCGAGTACAAAAAGTCCTTGAATTGGCGTCAGGTCATCGGGTTGTTGGCACTACTTAAAATCGATACAGCCACAAGTAAACAAATTATTTCCGAATTAGCAGGAGGTCTAGTCGACCAGAAACAGATAGACGAAGCTGTTTTACTGTACGAAATTTATTGTGAAGATTATCTTAAAGCTATTTCTGTTTTGCTGAATAAGGCGATGTTCCAAAAGGCATTGTGTATGGTAAGGAAGTATAATCGTCTAGATTTAATTGAAACAGAAATTCAACCAGCTATAATCAAATATAGATCCGAGTTATACGATAAGATAACAGCTTTAGGAGAGCAATTTTCAATGTATTGCACACGTTTATTAACGGTGCGCCACAATAAGTTACTAAAATTAAAAAACAGATATAAAAATGAGGTGTCTGATGTTTTGGGGGATGAGGACATTTACTCGGAGTACGGAAGTGTGATATCTTCAAGAGGTTCAACGTTGGGATCCAACAAATCAAAAATATCGACAGTGTCATCTAAAAACAGGAGAAAACTTAAAAAGAAAAAAATCGATCTGCGTGAAGGGGGTGTTTTTGAAGATATCGCATTGATAAGGCAGTTGTATCTATTGACAGTTACCATATTCGAATGCGGAGAGGAAGTTAGAGAAATTTGTTTGACATTAACGGACGATTTGTCATTTGATTTCTGTAAAATGTTACATAAAAATCTCGAGAACGTACAAGAAAAGGTTAAGTTAAATATTCCAGAAATATGGTCCGATACTTTTGTAAATGCGTCTGAATCTACAGAACCTGAAATATTAGCCGTTGTGCAAAACAGGGAAGAATTAGATATGGAATTTCGAACTCCACCACTTCAAGACAAACTCGACGAAACCTGGAAATTACTAATATTTCAGTAGTCATTAAGAAATTTATATTAAATTTATCCCATTGTAATATTTATTCAAAAGAATCAAGTCAAGTTTTTTTACTATTGTTATGTTCAAATAACTTTCATGATTTAAAAATTCTCGTGATAACTTTCTAAAAAATTCTAATCTTAGTTCGCAAAAAAAGCTTCAAATCATCAAAAATTGTTTTTAATTGTATTTTCCTATTTAACAAAACAAAATAATAAAAGCATTTAAAATAACTTATCATTATTTAAATAATTTTACTTGGTATCTAAATTATTCCATTCGGGCGCTATCTGGTTAGCCGTCTCGTACAGGTCAAGTAAGTAATTACATTTTTCTTCCACCTTCTTCTTTTGTTCAGTCAAGGCGGACGGCATTTTTTCTAGGTTGGTATTTAATAACACATCGAGGCGTTTGAAGACTTCTACATTTACTCGATGGGCCTCCATAATGGATTCTACGCAATTTTTGCCAGTTTCTTCAAAGCATTTTTCCACTTCTTGTAACTTTAACTCAGACTCTCTTAATTGCTCTTCTTGTTGACTAATTTTACTGTCCCAAGTCTCCCTTACTTGATCGATAAAAGTCTTCATATTGTTCTCCTCTTTTAAAATGAGCTCCGAAACCTAAAAAGGTGTAAAATTATCTGTCTCTCTAAACTACTAAAGTTACCTTGTTTATTTCTCGTTTAGTTTTCTCAATCTCGATTTGCAAATTTTGTATACTTATGGTCATCTCGTTTATCTCAACATCATCGGTTATTTGCTCTTGTTGTTCTGTTAGTTTGGCGATTTCTTTCTTCAACTGGTCGCATTCCGCTTGAAATATAGGTTCGAGAATTAACCTTGCCTTTTGCTGTTCTGTTATTTCCTCCAAAAAAGCCGGCGAATGAAACCCTCTACTTCGTAACTGAAGTTGTTCCATTTCAGGCAGGAAAGTTTTCATAATTAAAATATTCCACTCATCAATTTCTTTCTCAATCTAAATAAGTTATTTTAAATTCTCCATTGCGCATTTATGATGGATTATTTAGTTCAAAACGTAAGGATCACTTACGAATTTAATCAGTTCTATTTGTTGATGATCTAAGTTATCACGTATTTTGAGAATTTCGGAAAAGGTTTCTTTTCTTCGAACTATATATCTCTTTTTCTCCTCTATATCGTGTAACAATTCTTTTTTCTCTTTAAACTGACAAGGCTGAGCTAATATCTGTTCTTTTAGACATTTGATAAGTTCCTTTTTTTTCTAGTTCCTGGGCCTTTTGTGCAATTATTTTTTCCTTTGTTTCGACAATCTGTTCAGCCAAATTAATATCTTCTTGTCCATCATCCGCCCTGAATTCATCAATGACCTTTTGTTTATTCTCTAGGTCATTGAGAATTTCCATTTTGTTTTGAAGCAAATTTTCCAACTTTTTATCTGTTTCCGCTATGTCTGCTTTGAGTAATTCGTTTTCTTTCTTCAGTTTTTCCTGTTTCTCATTATCAATACTGAGTATTTCTGACAATTCAGTTTGAAATTCTTCTACTACGATTGGTTCACTGGCCGCCTGATCCAAGTGTTGGTAATTTGTTGTTAAAAAATTTAAAATACTTTCTTGTAAATTATAAGTGATTCTAAATTCGTTGTCAAGTTCATCGGAAAATGTATATTCCTGAGGATTGATTTTGTCAATCAAAAAAGAGACTATAGTTATGAATTGCGGGAAACCGTGCATCGTATTACCACTTTTTACACCCGATATCGTTAATGATCCTGGATATCTTAACAATTTCAAATGGGATACAACAACGTCCGCGAAATTCTGTGTGTTAATATTGACAACTGGAAAGATCTCCTTTAATAGTGTGGACAATAGCTGAACGAATATACTGATTGTCGGCGGTTTTATGGAGTCTGCCATGCGAGGGTCAAATAATCCACTGGTTTTAATAAAATGTTGGCACTTTGCGTATTGTTCTGCAACCCATTTTTTATCGTTTAACTGTTTTTGTTTCATTTTCATTTCTTTCGGTGATACAGACAGGGTGCCACTTTGTCTCATGCTGCTACAAGAGCCACTCCGTTTAAGCCCTCCGTAATTTGAAGTTGTGGGAGTTAAGTAGGATTTCTTTTGAGCGCTTAATAATTTTTTATCATTGAATCCCGGTCGTCCATCAATTGGGCTCATTTTTCTATCCAAACTTGGATTTCTTCCAGTTACGCCAGTTCTAGAAAGAGATCTTGTCCTTTCTTTCCCTTTTGGAAGAGGAATGTTTGTGTACGACCTCGATCGCCTCATTTTGAAAAATATTTCATCTTCTAAACACGTTTTTTAAATCTATCGTTCTTTACTGTCTGACAGACTTTTTGCTTTCACGTCGTTAACTTTTAAATTTGAAACTAAATTGACAGATGACAAATTCATCTTCTTTTTGTGGTCTTATCAAATTCGGCCAAAATGGGTTTATTAGTTTCTATTAATTTAATAACCAGGCATATTATAGAGGTGATTTCACTTTGCTGAGAACTATTAGTCACTCAATCAATTTTAAATATTAACACATTGTTTTGATTTCTATTCTTAATTCATATGGGAATATTGCACCAGGTTTTTGTTTAAAGATTAATTGTACAACAGGGCACCAACTTAAAAAAAGAAGAAAAAAGTGTAGGTTGCGTTGTTATTTATTTATATACAAGTGAATAATTTTTGTAAAATGGATCCCTCAGTTGAGCACGAGAAATTTATTCGAGAATTTATAGAGATTTATAAAAACAACCCAGTTTTTTGGGAAAAGGATTATTCCACAAATACTAAAAAGTCGGATCGAAACGTTGGAATGGAATTATTGATAGAAAAGTGTAGAGAATATTTTCCAGAAACTGGGGCAGATTTTGCAAAAGGCAAACTTGATACCTTAAGGGGATGTTTCAGGAAAGAGTATAAGAAAGTTCAAGAATCTAGGAAAAACGCGACTGCACCTGAAGAGATTTATAAACCTTCTTTGTGGTACTATAATCTATTGTTGTTCACTGTAGGCGAATCCCCTGACGAAACTGCGGAAACGTTTCCTGAATTTAATAGATTATCTTATTGGAACAGGGAATACACTCTACTGTTAATAGAACTGCTTAAAAAATATCCAGTGTTGTACTCAGCTGAACCAGACAATAACAAGGCCAAAAGAATTATGGCATTTAAACAGCTCACGGAAGAACTTATTAAAAATACTGGTAAAAAATTCAGTTTTCACGAAGTACGAAAAAAAGTAAGATTATTAAGGGATCAGTACAGACGAGAGAGAAGAAAGCTAAATTTGGGTGTAATAACTAAAAGTTCATTATGGTGTTTTGATCTGTTAAACTTTTTAAAAGCTAATTCTGTTACAGTGAAAAAGGAGAGTACACAAATCAATATTACATCTGAATCAGAGAATGAAAAGGATGCTAATAGTGAATCCGAAAGATTTTCAGATATTGAACCCTCCGAAAATGTTTTCAGAAGGGACAAAATTAAGACTGAAAATAGGATAAGCGATCCTACGGATGTTTTCGGTAGAAATTTGGCCTCTAAGTTTCGAGATATGCAGGATGAACAACGAAGTTATGCGGAAAGCCTTATTAATGAGGTTATGTACCACGGAATGATGGAAACTTTAAAAACTAAATCACAATTATTGATTTAAAAAAATTGGATGGACTCTTAACTCTGAAAAATTTAACTCTGTTAAATAAAACAATTTTTTTCTTATTTCGATTATTTTCTTGGGAAATTGCACAAATCTGCATGGATGACGTCACGGTCATAGTGATGTCGGGTTCATAGACAACACCACCAAGTCGGGTTTAGCTAAACGGTCTTTTAATGTCAACCAACATAATAATTTAATTAACCAGCACTATAACTGTGTTATATTTAATCTAAATCGCAGTATAACACTTTCTATTTTTGGGGTTGAAATATAGTCTAAGGGGTGAATATTAATTCGCTCCAAACCACGTGATCTAGTATGGCCGGAATAGTCAACATTGTAGAAGTTATATTTGTGTATTGTTATTATATATTATATTGTTTTTTAGCATTCTGTTTTGTGGGTTAAGTGATTTTGAGATGGAGAGCAGTTATTTAAAGAAGTTTGACCAGTGAAAATATGTATATTAGAAATTATTTAGTGTTTGCTCATCGTCGCGCAGCACCTTTAAATTTTCTTTTGATGTTTTATAGTTACCTA

Protein RF 1&2: 490->3709 (1182AA)

Comparison with *Tribolium* PREDICTED: similar to CG10535 CG10535-PA (1172AA)

Range 1: E=3e-137; bits=335

Query 490 KIESVAWNPSETFL--ILCDTEENLYGLTFECNIGFKEYRFIETCKSNLHDPVPNSVYVG 663

KI ++WNP+E + + D E + + + +E F + KS+ +P++VYVG

Sbjct 97 KIIEISWNPTEELVAVVFDDGEISTFCIDYENAEAFPQ------GKSSTDAGIPDTVYVG 150

Query 664 WGSHNTQFKGSKKiideeiqeipqqY--SPPIISWRGNGEQFVINFWKEERRYLKLFDST 837

WGS +TQF+GS+ I ++ + P ISWRGNGE FV+N+WK+++R +F++

Sbjct 151 WGSKDTQFRGSEGKIKDQTPVETKPVFDQKPRISWRGNGEMFVVNYWKDQKRQFIVFETP 210

Query 838 LRPLYQSESYPNLLALTSFKQQGNFIACATIHNNQNKIVIFEKNCLVKSEF-LVPEVKGV 1014

+ LY+SE P L +++ GN IA ++ N Q KIVIFEKN + +F L +V +

Sbjct 211 CKALYRSEECPGLQPQVAWRPVGNMIAGLSVTNRQ-KIVIFEKNGQRRFDFDLTFDV--M 267

Query 1015 IRNILYHPFLHILAIYSLEEAGNGYINIFVNSNAKWYLKQQLLYVTEKQVKCLNWADSLE 1194

I+N+ + P ILAI+++ G I++ +SN KWY KQ L + E + +W D+

Sbjct 268 IKNLKWSPCAQILAIHTVTPTGQT-IHLLTSSNYKWYEKQVLEFPAENALLDFDWLDT-- 324

Query 1195 NSICKLNILTSSSIERIVFRMVINRSPNNGTVAVVDGSIVHFTFFDSEIVPPPMSSAFLK 1374

+L ++T S + + FR V++ + + V+DG ++ T F++ ++PP +

Sbjct 325 ---NQLQVVTQSDVIKYTFRNVVHHN-SAAICGVIDGKHLNLTDFNNAVIPPISYARRFT 380

Query 1375 FDKPINRIQFHPNLPQCILTDSTSNVKLLNFKNNTLSEILEDD--LVDKNFKPETFIPNY 1548

DK IN + F +L ++ DS +++K+ N + E D LV N K +P +

Sbjct 381 NDKQINFVTFRHDL--AMIIDSENDLKIFN--------VAEPDALLVTINLKKIVDLPQF 430

Query 1549 -LSFNDVTEIMEPINLNDRDTASLVYCTGCK---------INDNFYEFALNRSQD----- 1683

LS + E + S+ Y K I DNF L S D

Sbjct 431 ALSCHHFLLSSESVYFAVTSDESVFYSLDWKNPQANFLTAITDNFSHL-LQISGDYDNIS 489

Query 1684 --------LLINRN--VMC------------------------SKVS----SVFIYKSYL 1749

L IN N ++C +K+S S ++ SYL

Sbjct 490 GLKSLGNNLFINNNDSILCQIQTLGDQTYVCNLTSNNHFYLNEAKISDCANSFTLFDSYL 549

Query 1750 LFIHVSCKLYCVRLTSDIVHESSWELKDFYFRDVEQGAKIVC-VNSRPEIVLELPRGNLE 1926

L+ +L+C+RL E ++F R+VEQGA IVC V + P+IVL+LPRGNLE

Sbjct 550 LYTTKQSELFCLRLGQ--------EGQNFR-RNVEQGATIVCAVPNSPQIVLQLPRGNLE 600

Query 1927 TIKCRLISIDMVEEMLSDGKWQEAVNLIRLHKLNWNLLIDLNPQRFGENIPKFIKAAETV 2106

TI CRLISID+++++L++ KW EAV IRL KLN NLL DLNP+RF I F++ T+

Sbjct 601 TISCRLISIDILDKLLNEQKWAEAVRFIRLEKLNANLLFDLNPERFLRQIAHFVQGVHTI 660

Query 2107 SMLNSIVSEFGTSNCLTTMYSQCVKDRTFPVQNKTNKLLQILNYIIDLDPIANLISIVTL 2286

+ L +I EF N LT++Y K FP Q + Y +D + +IV +

Sbjct 661 NELTAICLEFEEGNVLTSIYKNWGKTTDFP-QKINTIFASLFKYFDSVDYSVYITTIVAV 719

Query 2287 QIKHFTLKSALKSVYKVYELNHL-EMCKKAIFQILLY-CQTPAVINMSFTLYNIEFVRFV 2460

+ F L+ AL + +Y +L E A+ + +Y C + LY++E F+

Sbjct 720 NLNFFKLRDALIYLQDLYRRTNLKEKLLNAVNTLKIYGCDHEKLYTECLLLYDLELAGFI 779

Query 2461 HICCSEDPKVFEPQLDKLKGLGTVDLRFQMNVLAKK 2568

CC DP+V+EP L +L GL V++R+++N+ AKK

Sbjct 780 ASCCQLDPRVYEPHLKQLSGLNEVEMRYEINLFAKK 815

Range 2: E=3e-137; bits=175

Query 2564 KNYKGAVKFLIRCSNYQENYIREFITKHNTYEEVYKSVTPKNPNFTLCSRLFATYLSMKQ 2743

K K A+ +L+RC Q + + FI HN + +++ PKN + S FA LS K

Sbjct 814 KKPKTAIIYLLRCPKAQTSDVLAFIKTHNVSRQAFENCPPKNRFYQSVSHAFAGDLSAKG 873

Query 2744 RYNEAGLILQKAGLYQDAIIEYKKSLNWRQVIGLLALLKIDTATSKQIISELAGGLVDQK 2923

+ EAG++L++AGL ++A+ E++ LNWRQV+ LL L +D +I+++LA LV Q

Sbjct 874 CHTEAGVVLKRAGLPEEALAEFQLGLNWRQVLNLLEELNVDKVEKIKIVNDLATRLV-QS 932

Query 2924 QIDEAVLLYEIYCEDYLKAISVLLNKAMFQKALCMVRKYNRLDLIETEIQPAIIKYRSEL 3103

+ +A +L+E Y ++Y A+ VL+ F++A+ + K+ R D+I +++ P ++K++ L

Sbjct 933 NVRQAAILFEFYADNYEMAVKVLIEGFFFEEAIHIAMKHKRGDIIVSDVIPMLMKHKIYL 992

Query 3104 YDKITALGEQFSMYCTRLLTVRHNkllklkNRYKNEVSDVLGDEDIYSEYGSVISSRGST 3283

+K+ L E ++ Y RL VR +R+ N++ D +D++S+ GS IS +

Sbjct 993 EEKLQNLNESYNKYKQRLAQVRQQ----KFSRFNNDLDDCDERDDLFSDAGSTISKSSRS 1048

Query 3284 LGSNKSKISTVSSknrrklkkkkIDLREGGVFEDIALIRQLYLLTVTIFECGEEVREICL 3463

S S S+ + + K K+ DLREGG++EDIALIR L+ + GEEVR C+

Sbjct 1049 SRSRSSTASSRNRRKEEKKKQ---DLREGGIYEDIALIRALHSTIKEFYNKGEEVRNCCV 1105

Query 3464 TL--TDDLSFDFCKMLHKNLENVQEKVKLNIPEIWSDTFVNASESTEPEILAVVQNREEL 3637

L D+S+D K +H +++ + EIW F ++ + LA ++N E L

Sbjct 1106 LLLQESDVSYDEIKRIHDFYWKFDAEIQNGVAEIWPPHFYKNYQTLDVRELADLENFEHL 1165

Graphical representation


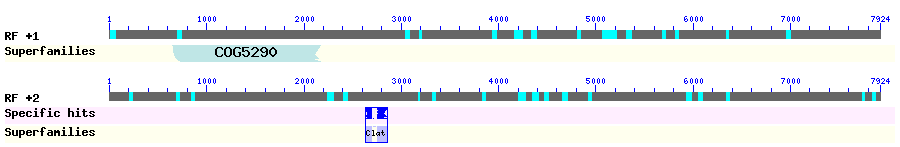


**GLD-1 homolog**

>Cp.comp39799_c0_seq20 len=2986

TTTTTTTTTTAACATTATAAATAATTTAATTATACTTTCTGTGCGCACAATTTCAATGACAAAGTCCTGACGTTTAGATATGTCGACATACCTACCGCTTCGGATCTCGTCCTAACCAATCTAAATTATATATATGACACTTTAAAAAATAAAAAAAATATTAACTTTGAAAAAGTCAACGTGTGTATGAAGCGGTTACGTCGCCATTATCGACCAAGGCAAGCTCGATAATTGCGACGTGACCGCACGTCCAAATAAAAATTACTAAATAATGCCTTGATCTGACTTGACGCGCATTTTAATTCCTAATGACATCGATATAATATAATATAAAGTTTCATATAATCAAATCTATAAGTACAACATTAAATATATACCTATATAACACACAATGTAACGTTTCCCGTTGCTCCGTTTAATAAAACAATTAAATTCAATTTTTTTCTAAAATAAAAACTTTGAGATTGCACAATCGGCCTCCCGATCAAAACTCGGACCGCCTAGTTATTGCACCAAAAATTACATTCTCTTTAGTAGTGAGTATTTTTTTTGTATTAACTGAAATGCGAACGCTGCTGGACGGACGACTTGATTATTTTGCTATTAAAATGAGTGACGTACCAATTCTTCATCTCTACATACTACGCGTACTACTTGTTGTCCAGAATACAGATTAAACTAAATCTCTAACGCACTTACCTTTACATATAATTGTATATATAATATATGTATAGCCTTAAAAAGTATTGCTTCTCATCGTCCGGAAAAATTTACGACCCGTATAAGTACTTGCAGAGAATTATTCGAGAATTTACGCCAAATCCGTCGATAAATGTTTCCGGAACTTTCCTACGTGCTACACATGGTTCGAAAAAACGTATTGGCACTATTAAACTTTTGTACTTCACCGAAGACACGTTATATAAAAAGGTATTAGTAAAATTATTAATGTCTAAACTAATGTGGCGCCGCCTGGCGCGCGGACGACGACAACTCTGCGTCCGCTGCCGCCCACTTTTCGGCGCCTCTTAAGCCCCGTTCACACGCCGCGCCGTCACACGAATTTCCGAGGCCATAAAACAAATACAACGTGCGAACCGGGCTTAAACAAAATTAACTGACACGATGACAAAATTAAAAAAAAAGAGTGAGAAAACAAAATTAAATTTAAATTAAATTAAAAAAAAAGTGTTAGTGATGTAATTGTTTTCGGTTAGACGTACCGCTACCCAATTTTTCTATAGCCGGGAGGGTGGGGGGCGCCGCCTCGAATGGTTTCGTTAGTTGGTACTTAGGAGTTGGCATGGATGTGTGACGTTTGTTAGCACTCCGCAAGCTTATATCGTGTAAAAAAACGGTTAAGAGAGAGCTCCGGCCCTCTGATAGGGGTGCTCTCTTATCTGCCCCAGATGCCTGCGTTGCTTGGCAACCGCCGACGCACCAGAATGGTCCGCCGTCGCGTACTCCGTGAGGAGGGGGGTGGCTGCCAATGCCGCGTAGTTCGTGTAGTCCGCGTAGGGGGCGTACAGGAGTCCGTGCGGGTCCCCCGGCGATAATAGGGATCCCGGGGGCGCCGATCCATTCAAAATGGACGCTGCCGTTGTCGGAACCGACATCCTGGGCGAAAGTATGAGGGGGGCGCCTAAAGGGGCGGCCGGTCCTCGCATCGGCGCCGCCAGTCCGGGAATCGCGGGCGACAGGAGCCTCTGGGCGTCGACCGCAGCCGCCGCCACTCGCCGCCATTCCTCGTCACACGCTGATGCCGAGGCAGCTTTCGCGCTCGAATCTCTGTACGTTCCGTTTATGATCGCCAGCTCCATCAGCTGCCTCTTTTTAAGCTCGTCTTCGCCTTCGGCTTGAGGTACGAGTAGCTTCCTGACTTCGTCGACGGCCCTTTGGAGTTTGATCTGGGCCCTGTTTTCCGTGTCCTCCACGGTCAAGAGCACGTGCAGCTCGTCGGAGAGGTGTTCCCAGTTGGGTTTACCCCGATTCTGATCCTCCTTCTTTTTGTCCCGCATCGAGCCCTTCCCCCGCACCATGATTTTACAGCCCGTTTCTTGCTCTAATTGCTTGGCCGTCATGCCTCTGGGCCCCAGGATCCTTCCCACGAAGTTGAAGTCCGGGTGTTCTTTTACAGGGACGTACACTTTTTCAGTCAGAGTCGTCACCGGTCCGTCGGGTTCGGGCAGGATCAGGGGCTCCTTTTTCACCCCATTTATTTGGAACAGGCTCGCCCTCACCTTCGCTATTTCTTCGTCCAGGAGCCTCTCGACGTGAATAAACACGTTGGGAAACGCCGCTAGCTGTTTCCTGTCTTTAAGCAATTGGGCCAGGTAGTCCGCTATGCTCTGCGTCGATGCGGCCGCATTTTGATCGCACATTTTGATACCACAAAATTAAAACAGTTCGAAATTTAAATAAACATCAATGCACAAGTGTTTGTGGCGTTTCAATTCGACCGGAACAGGACTCGACTTAAGCGGCTCGTGTACGCGACTGACTGGCTGAGCCGACTTGATCTACTGAAGTGTGGCGTACGGCGCGAGTGCGCGTGCGCTCGCGCCAAGAACGCTATGCGCGGTGTGGGTTGGCCTTATATGGGCCGCCCGTGATTCTTCAAAATTCAATCTTTATGCTTTTTCTGTATGAAATTTCATCGCCGATACGGCGTCGGATCTCGCCCTTATTCACAAGGCGCCGGATAATCTACTTTAATACTAGATAAATAACGAGTAGGTATTCGAGCGGTCTGGAGATTGTTTACATGAGACAGTAGGCCAAGATTTTTGTTTTGCTGCTTTTTTTGCACGCTATACTCTGCTAACTGTTATCTTCTGTATTAAGCCTAAAATGTTGTACGGGTGTGAAGTAGCAGCGAAAAGTTGGCAGCCGAAATTCCGAAAGTAGCCGAATGACCAGTAGCGGCGGTGAATTTATGATATTAGTAGATATACCATTACGAGGCCAACTGTTGCCATGATAATAA

Protein RF -3: -2381->-1359 (340AA)

Comparison with *Tribolium* held out wings (340AA)

Query 1 MCDQNAAASTQSIADYLAQLLKDRKQLAAFPNVFIHVERLLDEEIAKVRASLFQINGVKK 60

MCD ASTQSIADYLAQLLKDRKQLAAFPNVFIHVERLLDEEIAKVRASLFQINGVKK

Sbjct 1 MCD-TTNASTQSIADYLAQLLKDRKQLAAFPNVFIHVERLLDEEIAKVRASLFQINGVKK 59

Query 61 EPLILPEPDGPVTTLTEKVYVPVKEHPDFNFVGRILGPRGMTAKQLEQETGCKIMVRGKG 120

EPL+LPE DGPVTTLTEKVYVPVKEHPDFNFVGRILGPRGMTAKQLEQETGCKIMVRGKG

Sbjct 60 EPLVLPEADGPVTTLTEKVYVPVKEHPDFNFVGRILGPRGMTAKQLEQETGCKIMVRGKG 119

Query 121 SMRDKKKEDQNRGKPNWEHLSDELHVLLTVEDTENRAQIKLQRAVDEVRKLLVPQAEGED 180

SMRDKKKEDQNRGKPNWEHLSD+LHVLLTVEDTENRAQIKLQRAV+EV+KLLVPQA+GED

Sbjct 120 SMRDKKKEDQNRGKPNWEHLSDDLHVLLTVEDTENRAQIKLQRAVEEVKKLLVPQADGED 179

Query 181 ELKKRQLMELAIINGTYRDSSAKAASASACDEEWRRVAAAAVDAQRLLSPAIPGLAAPMR 240

ELKKRQLMELAIINGTYRDSS+KA SA+ACDEEWRRVAAAA + QRLLSPAIPGLA P+R

Sbjct 180 ELKKRQLMELAIINGTYRDSSSKAVSATACDEEWRRVAAAAAETQRLLSPAIPGLATPLR 239

Query 241 GPAAPLGAPLILSPRMSVPTTAASILNGSAPPGSLLSPGDPHGLLYAPYADYTNYAALAA 300

PA PLGAPLILSPRMSVPTTAASILNGSAPPGSLLSPGDPHGL+Y PYADYTNYAALAA

Sbjct 240 TPATPLGAPLILSPRMSVPTTAASILNGSAPPGSLLSPGDPHGLIYTPYADYTNYAALAA 299

Query 301 TPLLTEYATADHSGASAV-AKQRRHLGQIREHPYQRAGALS 340

+PLLTEYATADHSGA+AV AKQRRHLGQIREHPYQRAGALS

Sbjct 300 SPLLTEYATADHSGAAAVAAKQRRHLGQIREHPYQRAGALS 340

Graphical representation


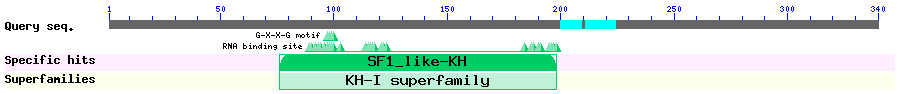


**ACO-1 homolog**

>Cp.comp40176_c0_seq1 len=3158

TAACACTTTGGTTGTCACTTGGTGGTGTTGTCTATGGTTTTCACGATTTGGAACATTGATATTAATCTGTTTACAAAAGTGAAATAGTGTAAAACCTTGTAAACTGATAAAACAATCAGCTATTCATCGCAATAACTGCCTGTAATCTTGGTTATCGTTGGCATGCTAATACGCAAATACTTTAGTAAAGAGAGTACGTGACTCAATTCTTCGCAGATATGGCAGAAAGCAACCCGTACAACAAATATTTGAAAACTCTCACTGTGGGGAGCAAAGAATATACCTACTATGATTTATCGGCTTTAGGAGAACAGTATAATCGGCTACCCTATTCAATAAGAATCCTGTTAGAATCCGCAGTGAGAAATTGTGATAATTTCTCAGTGAAAGAACAGGATGTCCAAAATGTCCTTAACTGGGAATCGAATCAAAATATTGAAGATGGTGTGGAAATTCCATTCAAGCCCGCTAGAGTCATTTTACAGGATTTTACTGGAGTGCCTGCAGTAGTCGATTTTGCAGCTATGCGGGATGCTGTCAGAGATTTAGGTGGCAATCCGGAGAAAATTAATCCAATTTGTCCCGCAGATCTTGTTATTGATCACTCGGTTCAAGTGGACTTTGCCCGATCACCAGATGCTTTACAGAAGAATCAAGATCTGGAATTTGAGAGAAACTTCGAGAGATTTGTGTTTCTGAAGTGGGGTGCAAAAGCCTTTAATAACATGTTGATTGTACCACCAGGGAGTGGTATTGTTCACCAGGTCAACTTGGAATATTTAGCAAGAGTAGTATTCACTGGATCCAATAAACCTATTTTGTATCCAGACACTGTGGTTGGTACCGATTCTCACACTACCATGATAAATGGTCTAGGTGTTTTGGGATGGGGTGTGGGTGGTATCGAAGCAGAAGCCGTTATGCTAGGTCAAGCAATTACAATGTTACTACCCCAAGTTGTCGGATACAAACTTTATGGCTCATTGGGGCCGTACGTTACATCCACCGATTTGGTATTGACCATAACTAAGCATTTGAGACAAATTGGAGTAGTCGGAAAATTTGTTGAATTTTATGGACCTGGAGTGGCGGCTTTGTCTATCGCCGACCGTGCCACCATCTCCAATATGTGTCCCGAATATGGAGCTACAGTTGGTTACTTTCCCGCAGACGAAATTTCTTTGTCCTATTTGAGACAGACAAATCGATCAGAAGAGCAAGTCAAATTGATCGAGGCTTATTTGCTAGCCACAAGGCAAATGAGAAATTATGAATCCGAAGAAAATCCTGTTTTCAGTCAGTCTGTTGAATTAGATCTTTCGACCGTTGTGTCATCTGTCAGCGGACCCAAGAGACCAAATGACAGGGTCTCCGTATCTAATATGAAAAAAGACTTCTTAAGCTGTTTAACTAACAAGATTGGATTTAAAGGTTTCGGAATACCAGAAAACAAAATCGGCACCAAGACTAAATTCATGTTTGACGGAAAACAATATACCATAGGACATGGTAGTGTCATTATTGCCGCTATAACGTCTTGTACAAATACTAGCAATCCTAGTGTTATGCTCGGAGCCGGTTTGCTTGCAAAAAAAGCTGTCGAAGCAGGTCTAAGTGTGGAACCGTACATAAAAACCAGTTTATCACCCGGTTCAGGTGTGGTGACTTATTATCTTCGAGAATCAGGTGTAATACCAGCTTTAGAGCAACTCGGATTTAATATTGTAGGCTACGGATGTATGACGTGTATCGGAAATAGCGGAGGAATTGACGAGAATATCGCCAACGCAATTGAACAAAACGATTTAGTATGTTGTGGAGTTTTGTCCGGTAACAGAAACTTTGAGGGAAGAATTCATCCGAATACTAGAGCCAATTATCTAGCTAGTCCGCTACTTGTAATAGCTTACGCTATTGCTGGCAGAGTTGATATCGATTTCGAAACTGAACCATTGGGTGTCAACGCTAATGGCACTCAAATCTTTTTGAGGGACATTTGGCCTACAAGGCGGGAAATTCAAAGTGTCGAGCAACAGCATGTCATCCCTGCAATGTTCAAAGAGGTGTACGCGAAAATTGAGAGTGGATCTAGTCAGTGGCAAACAATGAAAGCCCCAGAAGGTAAACTATATCCCTGGTCCAACGAATCCACCTATATCAAAAAACCTCCATTCTTTGATGGAATGACAAGGGAATTACCAAAGTCAAAGCCTATCCAGAATGCAAGAGTGTTGCTTTATTTAGGCGATTCGGTCACTACTGACCATATTAGTCCTGCTGGATCCATTGGCAGGAATAGCCCAGCCGCTAGATTTTTGGCGTCAAAAGGTTTAACACCCAAAGATTTCAATTCTTACGGTTCTAGAAGAGGTAACGATGCTATCATGGCCAGAGGCACCTTCGCCAATATTCGTTTGGTAAACAAGTTCATGAGCAAATCTGGACCCAAGACATTGTATTTGCCAAATAATGAAGAGATGGATATATTCGATTGCGCTCAAAGATACGCCACTTCAGGTACTCCCTTAATAATCATTGCCGGGAAAGATTATGGATCGGGATCGAGCAGAGATTGGGCTGCAAAGGGTCCATTTTTGCTGGGAGTACGAGCTGTAATAGCTGAATCTTTTGAAAGAATTCATCGTTCTAACCTGGTCGGAATGGGTCTGGTTCCCTTACAGTTCCTCCCGGGTGAAAACGCAGAATCTTTAGGCCTTACAGGCAAGGAGATATATAATATTGATCTGCCCGAAAACTTGAAGCCTCACGAACACATCACAGTCTCGACGGATACCGGGAAGAGTTTCAAAGTTCTGCTTAGATTTGACACCGAAGTTGATTTATTGTTTTACAAACATGGAGGTATTCTTAATTATATGGTTAGGAAAATGTTAAAAGGCTAAAGACTTGACACAGGACAAACATGGGGACGGTAGGGATGGTAATATTTAAGTCTTATTTAGAGAAGGGTTCCTCTGGGCTTTACACTGGGTTTATTATCGATTTTACACGCAATATCAATAGTTTGAAACAGAAGATATCTGGAGAAGTGGTCATATTATTTGTAAAGGCTTTTTACTTATTAGACGGTAAGAAAAAATGTTAAATCTATGATTTTTATGTATTGTTAGCAATAAACCAGCAATATTAAAAAAAAA

Protein RF 3: 219->2903 (894AA)

Comparison with *Tribolium* PREDICTED: similar to aconitase (997AA)

Query 4 SNPYNKYLKTLTVGSKEYTYYDLSALGEQYNRLPYSIRILLESAVRNCDNFSVKEQDVQN 63

+NP++KYLKTLTV SKEY YYDLSALG QY+RLPYSIR+LLESAVRNCDNF VKE DVQN

Sbjct 107 ANPFDKYLKTLTVESKEYKYYDLSALGAQYDRLPYSIRVLLESAVRNCDNFQVKENDVQN 166

Query 64 VLNWESNQNIEDGVEIPFKPARVILQDFTGVPAVVDFAAMRDAVRDLGGNPEKINPICPA 123

+LNWE NQ++E G+EIPFKPARVILQDFTGVPAVVDFAAMRDAV+ LGGNPEKINP CPA

Sbjct 167 ILNWEQNQSVEGGIEIPFKPARVILQDFTGVPAVVDFAAMRDAVKGLGGNPEKINPSCPA 226

Query 124 DLVIDHSVQVDFARSPDALQKNQDLEFERNFERFVFLKWGAKAFNNMLIVPPGSGIVHQV 183

DLVIDHSVQVDFARSP AL+KN+DLEFERN ERF FLKWGAKAFNNMLIVPPGSGIVHQV

Sbjct 227 DLVIDHSVQVDFARSPSALKKNEDLEFERNQERFTFLKWGAKAFNNMLIVPPGSGIVHQV 286

Query 184 NLEYLARVVFTGSNKPILYPDTVVGTDSHTTMINGLGVLGWGVGGIEAEAVMLGQAITML 243

NLEYLARVVFTG +KPILYPDTVVGTDSHTTMINGLGVLGWGVGGIEAEAVMLGQ+I+ML

Sbjct 287 NLEYLARVVFTGKDKPILYPDTVVGTDSHTTMINGLGVLGWGVGGIEAEAVMLGQSISML 346

Query 244 LPQVVGYKLYGSLGPYVTSTDLVLTITKHLRQIGVVGKFVEFYGPGVAALSIADRATISN 303

LP+VVGY+L+G+LG YVTSTDLVLTITK+LRQ+GVVGKFVEFYGPGVAALSIADRATI+N

Sbjct 347 LPKVVGYRLHGTLGQYVTSTDLVLTITKNLRQLGVVGKFVEFYGPGVAALSIADRATIAN 406

Query 304 MCPEYGATVGYFPADEISLSYLRQTNRSEEQVKLIEAYLLATRQMRNYESEEN-PVFSQS 362

MCPEYGATVGYFP DE SL+YLRQT+R +EQ+KLIEAYL AT+Q+RNY +E N P+FSQS

Sbjct 407 MCPEYGATVGYFPVDEHSLTYLRQTSRPDEQIKLIEAYLKATKQLRNYANEMNEPIFSQS 466

Query 363 VELDLSTVVSSVSGPKRPNDRVSVSNMKKDFLSCLTNKIGFKGFGIPENKIGTKTKFMFD 422

V LDLSTVVSSVSGPKRPNDRVSVS+MK DF CL+NKIGFKGFGIPE K+ T+ KFM++

Sbjct 467 VSLDLSTVVSSVSGPKRPNDRVSVSDMKNDFRLCLSNKIGFKGFGIPEAKLNTEAKFMYN 526

Query 423 GKQYTIGHGSVIIAAITSCTNTSNPSVMLGAGLLAKKAVEAGLSVEPYIKTSLSPGSGVV 482

G QYTI HGSVIIAAITSCTNTSNPSVMLGAGLLAK AV AGL+V PYIKTSLSPGSGVV

Sbjct 527 GSQYTIRHGSVIIAAITSCTNTSNPSVMLGAGLLAKNAVAAGLTVAPYIKTSLSPGSGVV 586

Query 483 TYYLRESGVIPALEQLGFNIVGYGCMTCIGNSGGIDENIANAIEQNDLVCCGVLSGNRNF 542

TYYL+ES VI AL QLGF+IVGYGCMTCIGNSGGIDENI NAIEQNDLVCCGVLSGNRNF

Sbjct 587 TYYLQESKVIDALTQLGFDIVGYGCMTCIGNSGGIDENIVNAIEQNDLVCCGVLSGNRNF 646

Query 543 EGRIHPNTRANYLASPLLVIAYAIAGRVDIDFETEPLGVNANGTQIFLRDIWPTRREIQS 602

EGRIHPNTRANYLASPLLVIAYAIAG VDIDFE EPLG +G+ +FLR+IWPTR+EI +

Sbjct 647 EGRIHPNTRANYLASPLLVIAYAIAGTVDIDFEKEPLGKRPDGSPVFLREIWPTRKEIHA 706

Query 603 VEQQHVIPAMFKEVYAKIESGSSQWQTMKAPEGKLYPWSNESTYIKKPPFFDGMTRELPK 662

VEQQ+VIPAMF++VY++I+ GSS WQ++ AP G LYPWS+ STYIKKPPFFDGMT++LP

Sbjct 707 VEQQYVIPAMFQQVYSRIQLGSSSWQSLNAPSGILYPWSDSSTYIKKPPFFDGMTKQLPP 766

Query 663 SKPIQNARVLLYLGDSVTTDHISPAGSIGRNSPAARFLASKGLTPKDFNSYGSRRGNDAI 722

+PI ARVLLYLGDSVTTDHISPAGSIGRNSPAAR+LA GLTP++FNSYGSRRGNDAI

Sbjct 767 MQPISGARVLLYLGDSVTTDHISPAGSIGRNSPAARYLAQNGLTPREFNSYGSRRGNDAI 826

Query 723 MARGTFANIRLVNKFMSKSGPKTLYLPNNEEMDIFDCAQRYATSGTPLIIIAGKDYGSGS 782

MARGTFANIRLVNKFMS +GPKT+YLP NEEMD+FDCA+RY ++ TPLII+AGKDYGSGS

Sbjct 827 MARGTFANIRLVNKFMSNAGPKTVYLPTNEEMDVFDCAERYKSAKTPLIILAGKDYGSGS 886

Query 783 SRDWAAKGPFLLGVRAVIAESFERIHRSNLVGMGLVPLQFLPGENAESLGLTGKEIYNID 842

SRDWAAKGP+LLGVRAVIAESFERIHRSNLVGMG++PLQFLP E AE+LGLTGKEIYNI+

Sbjct 887 SRDWAAKGPYLLGVRAVIAESFERIHRSNLVGMGIIPLQFLPNETAETLGLTGKEIYNIE 946

Query 843 LPENLKPHEHITVSTDTGKSFKVLLRFDTEVDLLFYKHGGILNYMVRKML 892

+P +LKP ++I +STDT K+F V+LRFDTEVDLLFYKHGGILNYM+RK++

Sbjct 947 IPADLKPGQNIKISTDTSKTFNVVLRFDTEVDLLFYKHGGILNYMIRKIV 996

Graphical representation


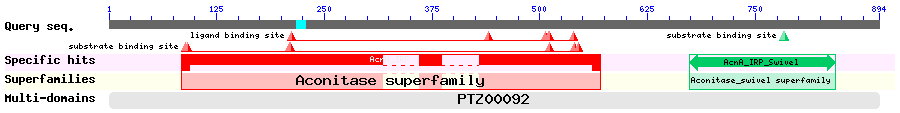

Supplement: S2 Supporting Information — (DOCX) [file pone.0115336.s002.docx]
