# Supplementary material for: Transcriptome Analysis and Systemic RNAi Response in the African Sweetpotato Weevil (Cylas puncticollis, Coleoptera, Brentidae)
Source: PLoS One. 2015 Jan 15;10(1):e0115336. doi: 10.1371/journal.pone.0115336 (PMC4295849; doi:10.1371/journal.pone.0115336)
Supplement: S3 Supporting Information — (DOCX) [file pone.0115336.s003.docx]

**Supporting information S3:** Sequences of *C. puncticollis* proteins involved in dsRNA uptake.

**Scavenger receptor SR-C-like protein**

>Cp.comp35050_c0_seq1 len=2249

GCAGCAGCGAAAACATTTCATAGATTATCATTTCCTTTTCCGAAATTATCTCGTCCCAACATCCGCTACCGATAGAGCTTTTCAAGTTTAACTGTGAATTTTTCAGTTATTTCGTGGCCAACTATGTTTTGCGCATTTTAGTGTTGCCAGAGTTATTAAAAATGCGAAATTTTTTAATTGTTTATTGTTTTCTCTTTAGTAGTGTTACGGTTCTAGACGTAATTGCAGAAGCTTGCCCGTCGGTGCGACTAAAAAACGGTAGGATAAGGTCGAGGCAAAGGGGGCGATTTCTCAAATTTATATGTAACACGGGGTACCTGTTGGCGGGAGAAAGGTACAGCATATGTACCAACGGGAAATGGGATTTGCCGCCGCCTAAATGCGTTCGTCCGACCTGTCAAAACAAAGCGAAGCCTCCGGAAAACGGACTGACGTTTCCATCGCACGGGGGCGGCGTTTTGAATTTCTACTGCAAAGCGGGATACGAACTGAGGGGCGCATCGGTGACGTACTGCGACGGTGTCAGATGGGACAACGTTCCGCCTACGTGTTTTCAATCGGATACAAAACCCAAATTGTTTTGCGACTTCGAAACAGACGACATCTGCAGCTGGACCCACGATTTAAATCACCATTTCGATTGGAAAAGGGAAAGTTACCAGACGCCGAGCGGATCGATCGGAACGGGGCCCAGTTTCGATCACACCAAAGGCGCTAAAGGGAAAAATGGGCACTACATGTACATCGAGTCATCTTCGAGGAGGGAAAACGACACGGCTCGTCTCATTTCGCCCGTGTTCGACAAAATGCCCAACAACACCTGTTTGGAGTTTTGGTACCACATGTTCGGCAAAACTACCGGCACTTTGAGAGCTTATGTGAAAAAAGTGCGAGACAATTGGCCCCTCGACCCCAACACTGCTATTTTTAGTAAAAGTGGCAACCAGGGTGACGTTTGGTATCGCTCTTTTCATAACCTGGGCACTATAGACGAAGACTTCCAGATTGTTATGGAAGGAGTGCGGGGCCCGGGATATGTCAGCGACATAGCCATAGACGACATCAAACTCATTGAGAATTGCAATCCAGAGGATTATACGTATACTACGACGGAAGAAACAGTCACAGTTGACGGCTTAATAAAAACGGTCGAATCTTGCGAGAACCGATGCGGACATATGGCTTCGGGTACCGAAACAAACTCTGTAATAGCCTGCGACTGTGACGATAACTGTTTCGACAGAAACCGTTGTTGTCCCGACTTTCTAGACATTTGCTACGCCAGTTTTTCCAGTACACCAACGTATGACGAGTCTACTGCCACCGAAACAGAAACTAGCGCTAGCGATACGACCTCAACGACAATTAGAGCTACAAAGCCAGCTCCCACTAGACGACCGCTCGTTATTCTTACCCCTCCTCCAGTACCTGAAATCGTGGAGCGAATTTTGCCGAAACAAGCGAAAAACGTAACCGATTCGTCAAAAATAACATTTCTGAAACCGAAAAAGGTCGAGCCTAGTGGAAACGTTGAAACCAACGATGTCATAGATGTCCCCTTGCTGAAGAACGAAGACAACGCGACGACGCCTCTGACGGAAGACGAAGACAACTCCATCTTCGAACGGGAAGAGTATATCGAAGAAATGGCTAAGGAATTTGACTTACCACACGAGCGCAGAGTCGACGAAAGCAAAAAAGAGTTCAGCCAGACTCAGGAAGAGCCGTCCAACGTGAAATTGTTGTTTGTCGTCATAACGGCGTGTTGCGGAGTTGGAGTTTTGGTCATCGTAACAAGTTTGATCGTCGTCAGGCACCGATATATAAAGTGTTACAAAAAACGAATGGTTTCGGGGAACGGGGACAGCCAGAGTGACGTCAGATTCTTAACGGCCGACGAAGTTTTGGATTTTTCGCTAGACAGAGACTACGAAGACTTGTGATTTGTACCAATACTAAATTATTAAACGAATTTTGTAAGAAGCTACACATTCCGAATTTGTCACCTTCAAAAAGCGTTAAAAACATCATTTTATGTAGAATTATATAAAATACGTCTCTTAAATAATTACATTTTTAAGCTATTCTAACCTAACCGCAGATATTATCATAGAACAAGATGGCGTTTGGTAAGGCTACACAATCATAGAACACAAATTGGCGAAAGATAAAGCTGGGTCTTAAATGATTTTTTTGTTTGACGGCATCCTTTATTCGTTAAGTAAAATAAAGAGAAAACGAAAAAAAGA

Protein RF 3: 1277->1618 (593AA)

Comparison with *Tribolium* PREDICTED: similar to scavenger receptor SR-C-like protein (543AA)

Range 1: E=4e-150; bits=455

Query 25 CPSVRLKNGRIRSRQRGRFLKFICNTGYLLAGERYSICTNGKWDLPPPKCVRPTCQNKAK 84

CP +++ NGR+R RQRG+ + +CNTGY LAG+RY++C G WD PKCVR TC+ AK

Sbjct 59 CPPIKVPNGRVRYRQRGKIARVLCNTGYTLAGDRYTVCVQGVWDNTYPKCVRATCR-AAK 117

Query 85 PPENGLTFPSHGGGVLNFYCKAGYELRGASVTYCDGVRWDNVPPTCFQSDTKPKLFCDFE 144

PP NGL +PSHGG VLNF+CK+ ++LRG+S+ YCDG +WDN P C +++ P L CDFE

Sbjct 118 PPANGLIYPSHGGAVLNFFCKSHFQLRGSSIAYCDGFKWDNPLPACLPTNSSPALSCDFE 177

Query 145 TDDICSWTHDLNHHFDWKRESYQTPSGSIGTGPSFDHTKGAKGKNGHYMYIESSSRREND 204

+ D+C W HDLNH FDW R +Y TPSGSIGTGPS DHTKGA GK+G YMYIESS+R ND

Sbjct 178 SGDLCGWNHDLNHDFDWMRLNYATPSGSIGTGPSHDHTKGA-GKDGFYMYIESSARNIND 236

Query 205 TARLISPVFDKMPNNTCLEFWYHMFGKTTGTLRAYVKKVRDNWPLDPNTAIFSKSGNQGD 264

TARLISPVFDK N C EF+YHM+G TTG+LR YVKKV + W LDP +++ K+GNQG+

Sbjct 237 TARLISPVFDKTDENVCFEFYYHMYGVTTGSLRIYVKKVNETWQLDPKKSLWEKTGNQGN 296

Query 265 VWYRSFHNLGTIDEDFQIVMEGVRGPGYVSDIAIDDIKLIENCNPEDY--TYTTTEETVT 322

W+R F +G I +D+QIV+EGVRG YVSDIAIDD+++I NC+P+D T TTT E T

Sbjct 297 RWFRGFVTIGAISDDYQIVIEGVRGSSYVSDIAIDDVRVIVNCSPDDAIETETTTAEPST 356

Query 323 VDGLIKTVESCENRCGHMASGTETNSVIACDCDDNCFDRNRCCPDFLDICYASFSST 379

+ +VESCENRC + + +I CDCD+ CF+R+RCCPD+ D C + S+T

Sbjct 357 WTPI--SVESCENRCDTNDTHLAHDWLITCDCDEACFERSRCCPDYFDFCLSETSTT 411

Range 2: E=1e-04; bits= 54,7

|  |  |  |  |  |  |
| --- | --- | --- | --- | --- | --- |

Query 547 LIVVRHRYIKCYKKRM-VSGNGDSQSDVRFLTADEVLDFSLDRDYEDL 593

+ V RY C+ +R+ + NGDSQSDVRFLT DEV+DFSL DY+ L

Sbjct 496 MAFVARRYQLCHWRRLKQTSNGDSQSDVRFLTGDEVIDFSLASDYDTL 543

Graphical representation


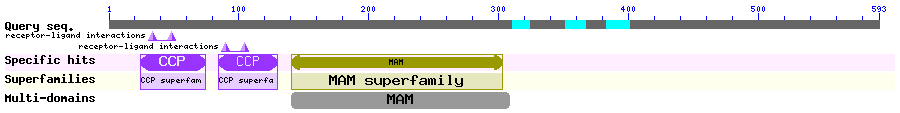


**Eater**

>Cp.comp38230_c1_seq1 len=1514

TTGTACAAACCCAAGAAAAACCACCCCATTAAAGTAAAGAAATCTGTCAAAAGTTATGAAATTAGACACTTTCAAATTACGTCAAAATACACCAAAGACGACAACAAAATACACTTCTTAAATGGAAAAACCTCAAACGGAAAAAACACAAATTTTTGAGCGCGAAAAAAAAGATTTTTTGTCCGCCCTGTAGAACAAACCGCGCTAGAAAAATAACCGAACGACTCGGATGACCCCGCTGTACCAAGAACGACCCGATAATTGTTTTCGATTCGGAGCTCTTATCGTCTTAGCGTCCGCCAGTTACGCTTTCTAATCTTCGAAAAATATACCGAATACGGGCAGTACTGTTTGGCGAGTGGCCGCGTGTATTTCGCAAAATCGATCAAAATGGATAAAATGTACGTTTCGTTCGCTCTCACGGCGGTACTTTCCATCTGGGCGGTACCAGGGTCGAGTCAACGTCTGTTGAATAGTACCGCCCATCACGTCAATGCCACTTCTTCTCACAGCAACGGGATATGTACGCTCGAAGTGCCTACCATCGATATTATAGCGCCTCAAGACAGGCACGGGGTGGTACCTAGAGGCAACGGGTCGCGAGAAGGCTTAAGCAGAATCGAAATTTGTTGTTCCGGGTACGAACGAGTACCCCACACGTACTACTCCTGTCAACCTGTGTGCGAAAACGGATGCGAGAACGGCAACTGTACCGCCCCCAACGTCTGTACCTGCAAACGGGGTTATATTAAGGCCGAGGACCCGAACAAATGCATCCCCACGTGCCCTATCGGGTGCCTAAACGGCGTCTGTTCCGTAAGGGGTTTCTGCGATTGCAACGCCGGTTACACTCACAGTACCGACGGCAAATATTGCGTACCGGTGTGTACCGGAGGATGCGGTATCGGCGGTAAATGCGTCGGGCCCGAAACTTGCAGTTGTTCGCCAGGGTTCGCGGTGAACAAGGACACGCGCAAGTGCGAGTACCACTGCGAGGGCGGATGCGGAGGCGGTTCCTGCGTAGGGCCCAACAAGTGTTCCTGCAAGCCCGGCTACAAATTGCTGGGCAATTCCTGCGCTCCGGATTGTCCTCAAGGTTGTTTGAATGGTATTTGTTCCGGTCCGAACACTTGTTCGTGCAAACCCGGATGGAACCTCGACAAAACCGGTACCAGTTGCGTGCCCCATTGCTCCTCGCCTTGCCTCAACGGGGATTGTACCGCGCCGGAGACTTGCACGTGCAAAAAGGGCTACGTAGAGGCTCCCGGTACTAACGGGCACAAGTGCGTGGCGTTTTGTCCAGAGGGTTGTTTGAACGGGGTGTGTTCCGCGCCCAATTTCTGCATCTGCAACGCCGGTTTTATGAAAGAGCGCAAGGGCAGTAACGCGTGCGTGAGGAGATTGAGGCGATCTTTAATGCACTTTGAACTTATACCGAAAGAAGTGCTGATGGGTCACTAAACACTATAAACAATGTATAAAATACAAATATACGGGCGTAAAATTGGAAAAAA

Protein RF 1: 391->1461 (356AA)

Comparison with *Tribolium* PREDICTED PREDICTED: similar to CG6124 CG6124-PA (1090AA)

Range 1: E=1e-43; 171 bits

Query 79 ICCSGYERVPHTYYSCQPVCENGCENGNCTAPNVCTCKRGYIK-AEDPNKCIPTCPIGCL 137

+C GY + P Y C P C C NG CTAP CTC ++K ++ +C P C CL

Sbjct 395 LCNPGYLKHPKNKYWCTPKCSKACINGKCTAPETCTCNNNFVKDLKNKYQCNPACTKPCL 454

Query 138 NGVCSVRGFCDCNAGYTHSTDGKY-CVPVCTGGCGIGGKCVGPETCSCSPGFAVN-KDTR 195

NG C C CN GY Y C P C+ C GKC PETC+C+PGF + K+

Sbjct 455 NGKCIKPETCACNTGYAKDAQSNYLCKPTCSKTC-TNGKCTSPETCTCNPGFTRDTKNKY 513

Query 196 KCEYHCEGGCGGGSCVGPNKCSCKPGY-KLLGNS--CAPDCPQGCLNGICSGPNTCSCKP 252

C C+ C G C GP C+C PG+ K L N C P C C+NG CS P C+C

Sbjct 514 LCNPTCKTACINGKCSGPETCTCNPGFIKDLKNKNLCNPVCKSACINGKCSRPEICTCNT 573

Query 253 GWNLD-KTGTSCVPHCSSPCLNGDCTAPETCTCKKGYVEAPGTNGHKCVAFCPEGCLNGV 311

G+ D K C P C+ PC+NG C PETC C G+V+ G + + C C + C NG

Sbjct 574 GFIKDLKNKNQCNPACTKPCVNGKCIKPETCACNTGFVKD-GRSNYLCKPTCSKTCTNGK 632

Query 312 CSAPNFCICNAGFMKERKGSNAC 334

C++P C CN GF ++ K C

Sbjct 633 CTSPETCTCNPGFTRDAKNKYLC 655

Range 2: E=2e-43; 171 bits

Query 80 CCSGYERVPHTYYSCQPVCENGCENGNCTAPNVCTCKRGYIK-AEDPNKCIPTCPIGCLN 138

C +G+ + + Y C+P C C NG CT+P CTC G+ + A++ C PTC C N

Sbjct 606 CNTGFVKDGRSNYLCKPTCSKTCTNGKCTSPETCTCNPGFTRDAKNKYLCNPTCKTACSN 665

Query 139 GVCSVRGFCDCNAGYTHSTDGKY-CVPVCTGGCGIGGKCVGPETCSCSPGFAVNKDTRK- 196

G CS C CN G+T + KY C PVC+ C I GKC GPE+C+C+PGF + +

Sbjct 666 GKCSGPETCTCNPGFTKNMSNKYLCNPVCSKTC-INGKCSGPESCTCNPGFTKDTSNKYL 724

Query 197 CEYHCEGGCGGGSCVGPNKCSCKPGYKLLGNS---CAPDCPQGCLNGICSGPNTCSCKPG 253

C+ C C G C GP C+C GY + C P C Q C+NG CS P TC+C G

Sbjct 725 CKPVCTKPCINGKCSGPESCTCNYGYAKDATNKYLCNPVCNQACINGNCSAPETCTCNSG 784

Query 254 WNLD-KTGTSCVPHCSSPCLNGDCTAPETCTCKKGYVEAPGTNGHKCVAFCPEGCLNGVC 312

+ LD K C P CS C+NG C APE+C C GY + N + C C C+NG C

Sbjct 785 YTLDVKNKYGCNPVCSESCINGKCAAPESCLCNPGY-KKDAKNVYWCYPVCSSPCINGQC 843

Query 313 SAPNFCICNAGFMKERKGSNAC 334

SAP C C GF + + + C

Sbjct 844 SAPETCTCYPGFTPDAQNRHVC 865

Graphical representation

NO PUTATIVE CONSERVED DOMAINS HAVE BEEN DETECTED

**Sid-1-related C precursor (Tribolium)**

>Cp.comp38247_c0_seq1 len=2656

TTTTTTTTTTTTTTTTTTTTAATTGTTACTCGTATATTTTCTTCTCATTTTGCATGAAACATTTAAAAAAAAAAAACTTCTTCACTTATATCACATTCAACTAACTTATTATCTCCGTAATGTGCAAACATCTTGCGATCCGTGCGACGTCAAAAATCAAGTTCGTGCACGCACATAGCGACAACTTTAAAACACCGTTATCTGACTGTGATGCGTGTGCGAAATGTCGTCGTCGAGAGTCAGCAGCACCATAAACGTAAAGAACATACCGATGGCGCTCAAAAAGTGCCAGATGTCGTGGTCGTCGTAAAATCTTAACCATTTACATTCGGTGTTGAATTGTCTCGATTGGGCCGGTGTTGCCGACCAGGATATTGTTCTATGTAGAAAGAAATACAATGCTGATATAGCGCAAAGAATGGATATAATTAGGAATATCCAACTGATGAATTTAACCCTTTCTCGATTCACATACTTCATAACGATGTAGAAGAAAAAGTACAACAGTGTATTAGACATGAACACCAATAAGAGGAAGACTGCGAAATTTTTCAGGTTATAGTGCAAGGCTACAAACGCCAGGCTCCAATTGCACATGTTTCCTAAGAAAAGCACACAGAAACGCACTTGGTAGCACGGCTTAAGCACGTTCAACGGTCCCGACCAGAAATCGTGCCTAACGATGTGTACTATCCGCATCAACGACATCTTATTTAGTCGCCAACATCCCATATAGTAAACCTTTACGCTCAAATAAAAGCAAGACAGGATGTAAATGACGACGAAGAATATCCAAAATCCCTTTGTACCTTCTAGTATGCCGATCATACCTAACAGTATTGCGATCGCCAACACTCCAAACGTTGTGTAAGCGTTTGCGTTAATGTCCGGATGTCGGTTTTGGTACAACTTTACCATACACAAAACCGCCATGACGTACATGAAACTAGTATCGAATTGGAAATTAGTCTGGCTCGGACAAACGTGGTAACTTCCGCTCAGTACCCCTTCCATTATCAGCGCCACTCCCATAGCGTAGAACAAACCGTAGTGCTGAGGTATACCGCAATGCCTATTAAAGTAAATATTGTTGTGCCAAAGTTCCCGGCGGTAAGTTATAATGAGGAACAGAAAACCCAATAAGACGTAACCTATGTTCGAGAAGACGTGATTGAAATCGCTCAAAAAACCCAACGGATGACTGCACAAGAAATTGTAATAGCAAAGGTCTTGCTGTCCCGTTTCGTTTAGTACCCTTTGGTATGTCACCACGAGTTGGACAACGGGCAAACCGTAAAACAAAGCGACCGTCAACACATTGTAAAGGTACAAGTAACTTTTTTTGGCTAAAACTTTCGGATGCCTCGTTGCCAAATCGTTCAGATACGGTTGCGTTCTGCACAACCTCATTTCCCTTTCAGACGGGAGATCTTCTATGGTATCGTAGTCTACTTCATCTATCGACTGACTTTCTTCGTTTGTGTTGGTACTTGAATTGACGGATGCAACCTCCGCATGAACTGCAAGGTCCACTTGCCGTGGTAAATAGTAGTGTTTGGAGCATAAAAAAAATGCTATGCCGAAAACAACATAAAATGCCCCTATTGAACCCAATGTCCAAGTAACGGCTAAAACATAATCGTTATAATTAATGCTGGGTTTAATAATGAGCGAAATTTTCTTTGTTCGATCCGTTGTCGTTTTTTTCGGGTCTCTCGTACACTCAAAGTCATCAGGTTTCGCTACGAAAGCTATGTAGAATCCATATGGAAATTTGTTTTTAGGTATGGTTATGCCCCCTTTAAAGTCAAAAGTTTCGTAAAATCCTCTGAAGGTTATGTCTTGATTCAAGTCAAACACTGGACAACTAATATTTTGAATACTGACAATGGTACAAACAGAATCCTCAGATTGTACTTCCAGAATAACAGTATCGTAATTAGATAGTTCATCAGTCAAATTTGTAGAAAAATTGTAAAAGAAATACCGAGGTTCGCTGGGCGATATCACAAAGTTATACTCGACAGAGTGGTGTAAATGGAACTCGCTTTCGGAATCTAACAGAATAGTAAAATCCACATCGACTTCCGACGCAGATGATATGGTTACTATAGGATTTTCACTTTCATATCTAGATTTTTTGATACTCTTTAAAATATCAAGGCATAAAGTCCTCGAAGTTTTCATATAGTATTCAACTCCCCTAGTCGTTTCAACTAATAGGGGTAAACTCCAGGACAATAAGTTTTTGGGCTGTGCCGCGACAATCATAAGTGGAGCCTCTACTGTGGCATTTTTTGACGTTACCGTTACTCGGGGAGGTATAGTCTCAAATCCATTCGAAAATTGCAAAATATATTCCACCGTATTATTAACACTGGCGGTAAATGGTGAAGAATAATTCAAATTTTTTATTATAGTTGTGTTCAATGTCCCACCCTTTGTATCGTTTACAAACAAAGAAAATAATATCACAGCAATTAAGTTAAACATTTTAATTCCGTAAGAGTACTATCTGTAGCTAGATATATTTACGTTAGTTCGCGACGGGCATTGTAAAGGACATAGAAAGGATATTTATATATCGGTGGTAAAGAATATTTGTCGTGACGGTATGATAAATGTTGGAAAAATTCTAAATATTTTGATAACTTTT

Protein RF -2: -2493->-187 (768AA)

Comparison with *Tribolium* Sid-1-related C precursor (768AA)

Query 29 NLNYSSPFTASVNNTVEYILQFSNGFETIPPRVTVTSKNATVEAPLMIVAAQPKNLLSWS 88

NL+YS+ +T S+N +VEYIL+FS PPRVT+ S +A ++ PLM+VA QPK LLSW

Sbjct 28 NLSYSNFYTFSINKSVEYILEFSAPELKYPPRVTINSSDAQIKTPLMVVARQPKELLSWQ 87

Query 89 LPLLVETTRGVEYYMKTSRTLCLDILKSIKKSRYESENPIVTISSASEVDVDFTILLDSE 148

LP+++E+ G + K SRTLC D+ + ++PIV++S+A+ +V FT+ +D +

Sbjct 88 LPMVLESDTGNHNFTKISRTLCHDMYRDYASRGITVDSPIVSVSTAAPRNVTFTVQVDYQ 147

Query 149 SEFHLHHSVEYNFVISPSEPRYFFYNFSTNLTDEL-SNYDTVILEVQSEDSVCTIVSIQN 207

+F + SV+YNF I+PSEPR++FYNF+ N+T+ SNY+TVILEV S+D VC VSIQN

Sbjct 148 KDFFIKPSVKYNFNITPSEPRFYFYNFTANITESPNSNYETVILEVFSDDFVCMTVSIQN 207

Query 208 ISCPVFDLNQDITFRGFYETFDFKGGITIPKNKFPYGFYIAFVAKPDDFECTRDPK-KTT 266

SC VFD NQDITFRGFYET + +GGITIPK KFPYGF+ FVAKPDD +CT P

Sbjct 208 ASCLVFDTNQDITFRGFYETVNTQGGITIPKYKFPYGFFAVFVAKPDDSDCTGIPSLYYD 267

Query 267 TDRTKKISLIIKPSINYNDYVLAVTWTLGSIGAFYVVFGIAFFLCSKHYYLPRQVDLAVH 326

T+RTK I+LI+KPSI+Y DYV AV TL SIG FY V F CSK Y+PRQ++ V

Sbjct 268 TNRTKTITLIVKPSISYQDYVNAVIATLSSIGIFYFVLIAGFIFCSKRGYVPRQMEY-VS 326

Query 327 AEVASVNSSTNTNEESQSIDEVDYDTIEDLPSEREMRLCRTQPYLNDLATRHPKVLAKKS 386

+E A+ ++ + S+DE +YD + + ++ +RL ++ YL+DLA + P+V KS

Sbjct 327 SEPATPSTCLGEEVDEISLDETEYDVVSEADQDKSIRLGKSVVYLSDLARKDPRVHKYKS 386

Query 387 YLYLYNVLTVALFYGLPVVQLVVTYQRVLNETGQQDLCYYNFLCSHPLGFLSDFNHVFSN 446

YLYLYNVLTVALFYGLPV+QLVVTYQR LNETGQQDLCYYNFLC+HPLG +SDFNHVFSN

Sbjct 387 YLYLYNVLTVALFYGLPVIQLVVTYQRALNETGQQDLCYYNFLCAHPLGVISDFNHVFSN 446

Query 447 IGYVLLGFLFLIITYRRELWHNNIYFNRHCGIPQHYGLFYAMGVALIMEGVLSGSYHVCP 506

GYVLLG LFL ITYRRE+ H ++ F R GIPQHYG+FYAMGVALIMEGVLSGSYHVCP

Sbjct 447 SGYVLLGLLFLGITYRREITHKDLNFERQYGIPQHYGMFYAMGVALIMEGVLSGSYHVCP 506

Query 507 SQTNFQFDTSFMYVMAVLCMVKLYQNRHPDINANAYTTFGVLAIAILLGMIGILEGTKGF 566

+ NFQFD+SFMYVMAVLCMVKLYQNRHPDINA AY TFGVLA+AILLGMIGILEG F

Sbjct 507 NTANFQFDSSFMYVMAVLCMVKLYQNRHPDINATAYATFGVLAVAILLGMIGILEGNLYF 566

Query 567 WIFFVVIYILSCFYLSVKVYYMGCWRLNKMSLMRIVHIVRHDFWSGPLNVLKPCYQVRFC 626

WI F +IY+LSCFYLS+++YYMGCW+L+ MR+ I ++FWSGPLNV+KP ++ R C

Sbjct 567 WIVFTIIYLLSCFYLSIQIYYMGCWKLDAGLAMRVWRICVYEFWSGPLNVIKPIHKARMC 626

Query 627 VLFLGNMCNWSLAFVALHYNLKNFAVFLLLVFMSNTLLYFFFYIVMKYVNRERVKFISWI 686

+L + N+CNW +AF ++ + K+FA+FLL +FM NTLLYF FYIVMK +N+ERV +S

Sbjct 627 LLIIANLCNWGMAFWGVYKHQKDFALFLLAIFMGNTLLYFSFYIVMKIINKERVNKLSLF 686

Query 687 FLIISILCAISALYFFLHRTISWSATPAQSRQFNTECKWLRFYDDHDIWHFLSAIGMFFT 746

FL +S+LCAISA+YFFL+++ISWS TPAQSRQFN ECK LRFYD HDIWHFLSAIGMFFT

Sbjct 687 FLSLSVLCAISAMYFFLNKSISWSRTPAQSRQFNQECKLLRFYDFHDIWHFLSAIGMFFT 746

Query 747 FMVLLTLDDDISHTHHSQITVF 768

FMVLLTLDDD+SHTH ++I VF

Sbjct 747 FMVLLTLDDDLSHTHRNKIVVF 768

Graphical representation


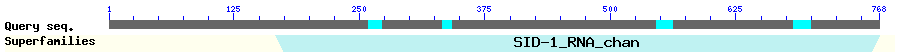


**FBX011 ortholog**

>Cp.comp39489_c1_seq2 len=3523

AAAAAAAAAAAAAAAAAAAGCCGCCCCCCCCCGCCCGCGAGCCGGAAAAACGGACCCGGGGCGGGAGGCGGCTCGGCTGGCTCTTCAAAAACTGCCGCCACCGCCGGGACCGAAAAACAGGAACACTTGGAAGCGGTGGCGCAACGCCCGCACCTAGTTGACCATGAGCGTGTGCGATTCCATCGGCGCGGCCGAGTCGTAAAGGGTGTCCGTGTCTTGGGTCGGTTCGCCTTGCAATTGGCACTGGTTGGTGAGCGTGCCCGCGCCGCAGTCGCAGAAAAACCTGTCGTGCCTGATGAACTCGACGTCGTGACCGGCGTGACAGGTCTTGATGCAGTTGACGCAGATGGCGTTGCGGTCGGTGGTGTTGCACGTCTGGCATCGATAGAAATCGTGCATCGGGAACGACGTATACGACGAAATCTTGTACAGGCATTGGCCGTTCGCGACCGCCTTTTCCACCGCGTCCTGGTTACTGAATATCTTGTTGCCCCTGACTATGGGCTGAACGCCGCTGGCCAGGCACAGACCTCCGAAACGGTTATTAAAAATCTGATTGAATTCCAATGTGGCGGTAGCGTTGTTGGTTATTTCCACGCCGGCCGCTAATCCGTCGAAAATCCGATTCCGTCTCAGTATCGGATGACTCTGGGTGGAGATCAGGACGCCGGCCTGGGCGTTGCGGAATATGTCGTTTTCCTCCAGAATTCCCTTGCCGCCGTTAAAGATACAAATCCCGCCGTCGCGTCCGTCGAATATCTTGTTCCGTTTCAACGTCGGATTCGAATCGGTCTTGATCCAAACGCCGGCCATGGCGTTGTCGAATATCTCGTTCTGTTCGAGCAGGCCCAGACCGCCGTTGTAGACGAGCACTCCGCCGTTCTGGCCGCCCCAAATCTTATTCCCCCTTATCACCGGATTACTACCGGTCCGGATCTGAACGCCCGAATACAAATGATTGAAAATATCGTTGTCTTCCAGTTTGCCGTGACCGTTATCGTAGAAATAAACTCCGACCTGTTTGCCGGAATGGATTCGGTTACGGCGCAGTACCGGCGTACTACCGGTCGTGATCCATACGCCCGCCAAAGTGTTGGCGTACACTTCGTTCTCCTCGATCAATCCCTGGCCCTTTTCGTGCACGTAAATGCCGCCGTGCTGACCGTGGTGGATTTTGTTGTGCCTCACGATCGGATCGCTGTTCGTTCTGATTTGGATGCCGGCTAAGGCGTTGCCGTAGATGTTGTTGTGCTCGATCAGGCCCCTGCCTTCGCCGAAAATGTATACGCCGCCCTGATGCCCGTTGTATATTTCGTTCCTCCTAATAGTCGGATTGCTATTCGAGGTGATCCAAACGCCGGCGAAATTGTTCGAGTGAATTTTGTTGTCGATAAACTGTCCGAGACCGTTCTCGTGTACGTATATGCCGCCCGTTTGACCGTGGTGGATCTCGCAGTGTACGACGGTCGGATTGGCGCCCGCTTTCACTTCGAAACCGGCGATCCGATTGTTGTGGATGTCGTTCGCCTCGAAATAGCCCAGTCCGTTGTCGAAGGTAAAAATGCCGACGTCGCGTCCGTGGTGGATGTGGTTGCGCCTCATGATCGGATTGGCGTAATTTTTGACCCAGATGCCGGCCAAGGCGTTCCGGCTGATTTCGTTGTCTTCGTACGTGCCCTGGGCGTAATCGGTAACGTAAAGGCCGACGTTTTCGCAATCGCTTATGTCGCAATGTCTGATTACGGGATTGGCGCCCACACCGCTTACGCATACTGCGGCACCCACCACTGAAGTGCTCCTGATGATGCAATGGTCGATGGTAGGACTGCAATTTTCGCCGACCTCCAAACAGTAGTGCTTGTGGTGGGGAGTCGCCGATGCCGGTTCCGGCGAGAATTTCAAAGTGAGATGGCCGGCGTAGGCGTTCTTGGCCCCCTCGACGAACATCACCGTCGATTCCGACTCTCTCTCCAGTATGACCGATTCGGCAACGTTTCCCGGAGCGGCGCCGATCAAAGCGATATCCGAATCGATAACGAGAAACTCTCCTCGATAAGTGCCGGCGTGGAGGAATATCAAAGCCCCTTGGCCGGCGTTTTCCGAGGAGTGACTACCGCCCCCGCTGACCGCCAAACCGGTACCGCTGCCGCTACCGCTCCGTTCGTCGGCGTAATCTAAAGCACCCTGAACAGTATTGAAATAAACTAAATTACGACCCTTGAAGCTCAACTCTTGGTAGCCGGGCCGGACGTGTATGCCCCGGTACAGTTGCCGGAACGATTCCTTCCACGGATTGGCCAGTTCCGACTCTTCGGAGCTGACGAATTCGAAACGGCACGGCGACGGATTGAACAGCGGCAAATCGTACTCGTACACGTTCTGATAGAGACGCTTCCATAGTTCCGTGTCGTTGGCGATCACCTGGAACCGCTTGCACACTTGGCTCACGCGGCACAGGTCCTGCTCGAGCAGGTAATTGAATATGGTGAGCAACACTTCGTCGGGCAGTTCGTATTGTAAGTAGTGAGCGGCCGTATTTGTACAATTTTCGTACGAAGCGGAACAAGTCCTGCGGGGCCGTTTCCTGGCCGGCAACATGGCCGAACTGTAGGCGCCCTGGGCGGCCGGCAAGTTACACGGCGTCGTCGGCGAAGCGGGGCTGCCGCTCGCGGTGGCGGCGCTCGTGCTAGGCCCGGGGCCCCCGTCGGCCACCGGGGCGCCGCCACCGTGATTGTGCGACGGCGACTTGCGCCTCAGGTCGTACGGCGACTGGTGGGCGGCAGCGGCGGCCGCCGCCGCGACGACCACGCCGCCGGCCGCGCTCGGTCCCGGCGGGGCGAACGCGCCCTGCTGACCCAACGGGTTGCCCGCCAAGGCCGAAGGGGCGGGCGCGTTCTGGTTCGGCTGGACGGGACATGACGGCTCGCACGGTTCAGACGTTGTCCTCGAAGGTGTGGGTATCCTGTTTGCGCCTTTCCTGCGGGATCGCCTCACGTACGACCTCGAGGCCGTGAACGAGGCGCTGGGCATCTTGGACGATCGCTCGCGCGCACTTAAACGATTATTTTCCACCTTCTAAACAAAGGAAACACCAGCACTACTACCACTGACGACGGCCATTTTCAGCAGCGAAGTCGGAGACGGTCACGGAGTGCGCGGGCGCGGTCGACTGGCCAATCGCGGGGTCGGGGCGCGCGTCACGTGACCCGCCGCAAACGTCAAAATGGGGCACGATTTCGACGCGATTTTCGGCTGTCTCGTGTCCGTCGGACTGTGCTTACTACTTTCCGTCGCTTATCTGGCGAGCCTGCACGTCTGGAACAGCCCTTTCAGCAGGTAAATGTTTAATTTCGAGCGCGATCTCGCGATTTCCGAACCGAGTCGACGTTGGTAACTTGGCTCACTTTGCGAACCTGCGGGGGCCGCCTCTCTCCCCGCACGTCGCGCGCCCGTCTGCCCCTACGAAATAAAAAGAAACACAAACGTCAAGGGTGACAACGAACCGGGTCAAAACGTTTCAGAG

Protein RF -2: -3030->-154 (958AA)

Comparison with *Tribolium* hypothetical protein TcasGA2_TC010102 (915AA)

Query 1 MPSASFTASRSYVRRSRRKGANRIPTPSRTTSEPCEPSCPVQPNQNAPAPSALAG----- 55

MPSASFTASRSYVRRSRRKGANRIPTPSRTTS Q + N P

Sbjct 1 MPSASFTASRSYVRRSRRKGANRIPTPSRTTSAS-------QSHANLPVLYLTKTFQHLR 53

Query 56 NPLGQQGAFAPPGPSAAGGVVVAAAAAAAAHQSPYDLRRKSPSHNHGGGAPVADGGPGPS 115

PL G + P +GG + AH SPYDLRRKSPSH+ GPGPS

Sbjct 54 RPLRALGGPSAMAPGTSGGAI-------PAHHSPYDLRRKSPSHH---------DGPGPS 97

Query 116 TSAATASGSPASPTTPCNLPAAQGAYSSAMLPARKRPRRTCSASYENCTNTAAHYLQYEL 175

TSAATA GSP SP TP AQG Y+SAMLPARKRPRRTCSASYENCTNTAAHYLQYEL

Sbjct 98 TSAATA-GSPTSPATPT--APAQG-YTSAMLPARKRPRRTCSASYENCTNTAAHYLQYEL 153

Query 176 PDEVLLTIFNYLLEQDLCRVSQVCKRFQVIANDTELWKRLYQNVYEYDLPLFNPSPCRFE 235

PDEVLLTIFNYLLEQDLCRVSQVCKRFQ IANDTE+WKRLYQ+VYEYDLPLFNP+PC F+

Sbjct 154 PDEVLLTIFNYLLEQDLCRVSQVCKRFQAIANDTEIWKRLYQSVYEYDLPLFNPAPCVFQ 213

Query 236 FVSSEESELANPWKESFRQLYRGIHVRPGYQELSFKGRNLVYFNTVQGALDYADERSGSG 295

F+S EES+LANPWKESFRQLYRGIHVRPGYQ+L+FKGRNLVYFNT+Q ALDYADERSGS

Sbjct 214 FISPEESDLANPWKESFRQLYRGIHVRPGYQDLTFKGRNLVYFNTIQAALDYADERSGS- 272

Query 296 SGTGLAVSGGGSHSSENAGQGALIFLHAGTYRGEFLVID-SDIALIGAAPGNVAESVILE 354

ALIFLHAGTYRGEFLVID SDIALIGAAPGNVAESVILE

Sbjct 273 ---------------------ALIFLHAGTYRGEFLVIDDSDIALIGAAPGNVAESVILE 311

Query 355 RESESTVMFVEGAKNAYAGHLTLKFSPEPASATPHHKHYCLEVGENCSPTIDHCIIRSTS 414

RESESTVMFVEGAKNAY GHLTLKFSP+ S PHHKHYCLEVGENCSPTIDHCIIRSTS

Sbjct 312 RESESTVMFVEGAKNAYCGHLTLKFSPDVTSTVPHHKHYCLEVGENCSPTIDHCIIRSTS 371

Query 415 VVGAAVCVSGVGANPVIRHCDISDCENVGLYVTDYAQGTYEDNEISRNALAGIWVKNYAN 474

VVGAAVCVSG GANPVIRHCDISDCENVGLYVTD+AQGTYEDNEISRNALAGIWVKN AN

Sbjct 372 VVGAAVCVSGAGANPVIRHCDISDCENVGLYVTDFAQGTYEDNEISRNALAGIWVKNNAN 431

Query 475 PIMRRNHIHHGRDVGIFTFDNGLGYFEANDIHNNRIAGFEVKAGANPTVVHCEIHHGQTG 534

PIMRRNHIHHGRDVGIFTFD+G+GYFEANDIHNNRIAGFEVKAGANPTVV CEIHHGQTG

Sbjct 432 PIMRRNHIHHGRDVGIFTFDSGMGYFEANDIHNNRIAGFEVKAGANPTVVQCEIHHGQTG 491

Query 535 GIYVHENGLGQFIDNKIHSNNFAGVWITSNSNPTIRRNEIYNGHQGGVYIFGEGRGLIEH 594

GIYVHENGLGQFIDNKIHSNNFAGVWITSNSNPTIRRNEIYNGHQGGVYIFGEGRGLIEH

Sbjct 492 GIYVHENGLGQFIDNKIHSNNFAGVWITSNSNPTIRRNEIYNGHQGGVYIFGEGRGLIEH 551

Query 595 NNIYGNALAGIQIRTNSDPIVRHNKIHHGQHGGIYVHEKGQGLIEENEVYANTLAGVWIT 654

NNIYGNALAGIQIRTNSDPIVRHNKIHHGQHGGIYVHEKGQGLIEENEVYANTLAGVWIT

Sbjct 552 NNIYGNALAGIQIRTNSDPIVRHNKIHHGQHGGIYVHEKGQGLIEENEVYANTLAGVWIT 611

Query 655 TGSTPVLRRNRIHSGKQVGVYFYDNGHGKLEDNDIFNHLYSGVQIRTGSNPVIRGNKIWG 714

TGS+PVLRRNRIHSGKQVGVYFYDNGHGKLEDNDIFNHLYSGVQIRTGSNPVIRGNKIWG

Sbjct 612 TGSSPVLRRNRIHSGKQVGVYFYDNGHGKLEDNDIFNHLYSGVQIRTGSNPVIRGNKIWG 671

Query 715 GQNGGVLVYNGGLGLLEQNEIFDNAMAGVWIKTDSNPTLKRNKIFDGRDGGICIFNGGKG 774

GQNGGVLVYNGGLGLLEQNEIFDNAMAGVWIKTDSNPTLKRNKIFDGRDGGICIFNGGKG

Sbjct 672 GQNGGVLVYNGGLGLLEQNEIFDNAMAGVWIKTDSNPTLKRNKIFDGRDGGICIFNGGKG 731

Query 775 ILEENDIFRNAQAGVLISTQSHPILRRNRIFDGLAAGVEITNNATATLEFNQIFNNRFGG 834

ILEENDIFRNAQAGVLISTQSHPILRRNRIFDGLAAGVEITNNATATLE NQIFNNRFGG

Sbjct 732 ILEENDIFRNAQAGVLISTQSHPILRRNRIFDGLAAGVEITNNATATLESNQIFNNRFGG 791

Query 835 LCLASGVQPIVRGNKIFSNQDAVEKAVANGQCLYKISSYTSFPMHDFYRCQTCNTTDRNA 894

LCLASGVQPIVRGNKIF+NQDAVEKAVANGQCLYKISSYTSFPMHDFYRCQTCNTTDRNA

Sbjct 792 LCLASGVQPIVRGNKIFNNQDAVEKAVANGQCLYKISSYTSFPMHDFYRCQTCNTTDRNA 851

Query 895 ICVNCIKTCHAGHDVEFIRHDRFFCDCGAGTLTNQCQLQGEPTQDTDTLYDSAAPMESHT 954

ICVNCIKTCHAGHDVEFIRHDRFFCDCGAGTLTNQCQLQGEPTQDTDTLYDSAAPMESHT

Sbjct 852 ICVNCIKTCHAGHDVEFIRHDRFFCDCGAGTLTNQCQLQGEPTQDTDTLYDSAAPMESHT 911

Query 955 LMVN 958

LMVN

Sbjct 912 LMVN 915

Graphical representation


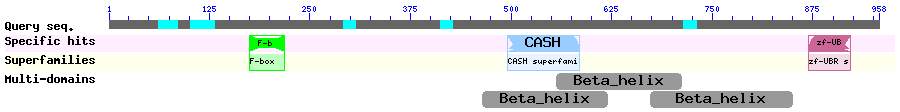


**CG4966=orthologous to the Hermansky-Pudlak Syndrome 4 (HPS4)**

>Cp.comp40396_c0_seq2 len=2644

CAAACACTCAATTTTATTCGCTACGAAAAACAATCGGAAACGTCTACCGAGCGACGCCCTAGAAGCCGACCGGAAGCACAAAAATGTTTGGCACAGAGAGAGAATATCGGCAATTAGGGCCCCGCTATAACTATAACTAAACCTAAGGACAGATGATTTCGAAACTAACCTAATGGTAAAATATCACAAAAACACTACCTAAAACTAACATACACCCATCTAAAACATGCACATTCGAGTATTAATAGTTAACGGTCGCCTTTTCAACTCGTTACGTACGTTCTTAGAGCAAAACGACAGCGTGGTCCCTTTCCAACCGTCGTTTGGCTTTTGTCTGTATCAGCCCGACGGGATCCGATGGAAGGGGCAAGCCGGACGCTGCTCCGGCACCCTCGTGATAAAACACTTCCGACGGGCCGCAATGGTATCCATAAATGGCGCTGTCTGTGTTTCTCAACAAAATCTCAGTAATATCAGGCGTATCCGTGAAATCTCGATGCAGAGACACCAAAGACGATAAATCGCCGGTGCCCCACGGTCCGCCCCTTTTCACCGTGTCCCATTTTGCGTCCAAGCACAGAAAGCTATACGGTTCGCTTTCCTGCGTCGCAGTCCCCGACGACTGAGATTCCAAGCAGAAATTGAGTTGTTTCTCGAGCTTGCCCAAATGTTCGGTGCAAGTTTCCCAGATTCTCACGATCGTTTCTTCATCTTTGCAGCACTCCTCCTTCATCAATAGAAGCGTAACAACATCCTGTTGGCCGCAGACGAACAATAGGCATTTACGCAACGCGCCGCCATCTTCTAACCTCAAATTTTTAATCTCTTTAGTTTTGTAAGTGAACGGAAAGGTGGGTTGAGTTGGGGTCGCCCCCAATGGCGTCGAATCTTTAATAGTCCTCTCCTCAAACGCTAGTACGCTCAATTTTGACATTAAGGGAGTGAGTTGAACCCCCAAAGAGAGCCTGCGCTCGTTCTTGGACCCTTCGGTACCACTGATTGGTTTCAACGGTAAAATTAATGAACGTCTGCGTTCCTTTGGCAGTTTATTGTCCTCCGCCACTTTGGAGATATCATTTGCACCTTTGGTGTCGATTTGGCACTCACGCGCCTCAATTGCCAACTGTTTATTCGGTGTCGAGTAACTAACAAGCGCCCGAGAGTCTTTCTCCACCAAATCTTCGTAATAATACTTAGAAATGGCAATTCCGTCTAATTTTAGTAGCGGAAACGACGGATCGTGAATGGATTTCGACCGGCGCGAATGCCTGTGCTTTCGGGGCGACAAACCCAGTTTCGCCTTAACACTAGGGTATCTGCCAGGGTCGTTTAGTATTTCGCTCAAGTGTAACGCGCTGTTGCTCCGCCCCTTGGGTTTTGACGGGCTCTTGTCTTTGTCGGGACTCTCGGTATCGTCTTCGTAAGTTTCCGGTTTGTTGATCAACGTGAGGGGGTTCTGATGCACGAAACGCTTTAGTTCAGTCATGGGGGTGCTCGCGACGCTCGTCTGTCCGCAGAACGGCGTCATCGGGGTGGCTTTCGACTCGTTCGACTCGACGCTAACACTTTTGAGGTTAAGAAACTTGGGACGTTGCTTCTTTTGGCTAGTTACGAATGTTATTTCGGAATTGTCCGGGCCTTCCTCCGGGACGGCTGTGAACAGTAACGACTGGTCCCGTTTCATAGCGGAAACAATCGGAGCTTCGCTCGAGGACTTCGAATGGGCTCTTTTGATGCTTTTGTTGTTTAAATACTGAAAAATGCACTTGTTCCGCATGGACGTCTCTAATAAATCGCAAAAATCTTTGTTGGTGATATAGACTTGTAAAAGTTGTACGCCTAAAGGTAGTTTGAAGGCAGTCTCTGCCGGCTCGGCGGGTGATTTGATCCTGTACGGATCGGCGATCACTATACGTTTCGTTATGTCAGTCGGCAGTTGAGTGGCTACAACTTTGTTGTGGTACAAAAGAGTGCCACCCAAAACGTGCGAGTACTCCCGGCATGCTTCTAGAAAAGTAACAGCTTCGGTAAATACCGCGCTGGCGCTCTTGGGCAAACACAATACAGGCACGTGGCTAAAAATACTGCCTCCGTAAAACAACATTCTCAGATACGTCTCGAACAAATGATACAATTTGGCTTTCAGCGCTTCGTCTTTGTATATTCGCCCCAACATATCAAAATCTTTGTTAAATAAAGTTACCAGAGAGTAAAGTAAGGTGCCTCGGTGCTCCAGAAGCCAGTCGGCTATGTTCCTGTCCGTCCCGACGGCCAAAATGTACTGCCCATTTTCGATTATATAAAATTTGCCGGTCTGCAGGCAGACGACTTTCGGCATTGAGAAAATCGACTTTAGGCAGCTGATTGTGCCTACTATTTGACCGCATAGGGCGGTTTTTTGTTGGTCCGATACCCATGTCGGGTGGAAGTATAAAATAGCGCTCGCCGGGTCGTCTTCTTCGTTCTGCAACTTGTCCGTGTCGTACACGAAAATTATCGTGGTTTCCTTGGCCATTTTAAGTTACGCCCCTTCAAAAAACGGCCCTATTCATTTTGGGGACTCTAAAACATCGATAATGTTTACAGATCACATAACCTCAATTTAAACGGGCGTGAAGTTCGAAACAAAAGAATGACATTTCTT

Protein RF -1: -2515->-284 (743AA)

Comparison with *Tribolium* PREDICTED: similar to CG4966 CG4966-PB (908AA)

Range1: E:=0.0; bits=632

Query 1 MAKETTIIFVYDTDKLQNEEDDPASAILYFHPTWVSDQQKTALCGQIVGTISCLKSIFSM 60

MAKE II VYD+ LQ EEDDPASAILYFHPTWVSDQQKTALCGQ++GT+ C+KSIFS

Sbjct 1 MAKEMMIILVYDSQMLQKEEDDPASAILYFHPTWVSDQQKTALCGQLMGTVHCVKSIFSA 60

Query 61 PKVVCLQTGKFYIIENGQYILAVGTDRNIADWLLEHRGTLLYSLVTLFNKDFDMLGRIYK 120

PK+V LQ+GKF+I E G+Y++AVGTDRNIADWLLEHR + SL++ F++D +++ ++Y

Sbjct 61 PKIVSLQSGKFFIKEYGRYLMAVGTDRNIADWLLEHRANTMSSLISFFHQDIEIMSKLYD 120

Query 121 DEA-LKAKLYHLFETYLRMLFYGGSIFSHVPVLCLPKSASAVFTEAVTFLEACREYSHVL 179

+ A L AKLY LFETYL+ +F GG+IFS+ P L LPKSAS VF EA+ L+ C+E ++V+

Sbjct 121 NSAKLSAKLYQLFETYLKYMFLGGNIFSYTPSLKLPKSASNVFLEAIQILQCCQELNYVM 180

Query 180 GGTLLYHNKVVATQLPTDITKRIVIADPYRIKSPAEPAETAFKLPLGVQLLQVYITNKDF 239

GGTLLYHNKVVATQL +DITKRIV+ DPYRIK PAE F+LPLGVQLLQVYI++K++

Sbjct 181 GGTLLYHNKVVATQLSSDITKRIVLTDPYRIKCPAETPSVNFELPLGVQLLQVYISSKEY 240

Query 240 CDLLETSMRNKCIFQYLNNKSIKRAHSKSSSEAPIVSAMKRDQSLLFTAVPEEG--PDNS 297

L E + R++ IFQYL++KSIK+ +S E P++SAMKRDQS++FTAVPEE P S

Sbjct 241 YKLHEEATRSRSIFQYLSSKSIKKGKPVASKE-PVISAMKRDQSIIFTAVPEEDSEPQIS 299

Query 298 EITFVTSQKKQRPKFLNLKSVSVESNESKATPMTPFCGQTSVASTPMTELKRFVHQNPLT 357

++ S + RPKFLNLK + + + A P TPF GQTSV STPMT+L + +H PL+

Sbjct 300 KVEKPISS-QNRPKFLNLKHKTTDEKKPVA-PSTPFHGQTSVCSTPMTDLSKVLHSKPLS 357

Query 358 L-INKPETYEDDTESPDKDKSPSKPKGRSNSA------LHLSEILNDPGRYPS---VKAK 407

+ IN+ E ++P+K +K NS L ++ L+D ++ S V+ K

Sbjct 358 ICINETIPCE---KTPEKTSIFAKNGTDLNSVIARVPYLTVTSNLHDCKKFSSVFDVREK 414

Query 408 L-----GLSPRKHR----------------HSRRSKSIHDPSFPLLKLDGIAISKYYYED 446

L G++ + + SR K+I DP++P+ + DG +S +Y+D

Sbjct 415 LKSLDRGITMKYYNSEFREKYKISHDVTPDESRVFKTITDPNYPIFRSDGTVVSHPFYQD 474

Query 447 LVE----------KDSRALVSYSTPNKQLAIEARECQIDTKGANDISKVAEDNKLP---K 493

+ K+ + +S+ + + + Q K + + + E + LP K

Sbjct 475 YLTSQMELITSEIKEEKPDLSHHSFDDFDSNLGDFVQSVKKIPDKVVESIEFSALPRPSK 534

Query 494 ERRRSLILPLKPIS---GTEGSKNERRLSLGVQLTPLMSKLSVLAFEERTIKDSTPLGAT 550

E R+SL LPLK +S G E RR S V LTPLMSKLS +D+TP+ T

Sbjct 535 EHRKSLTLPLKSLSVDGGGEEVSPMRRHSSSVLLTPLMSKLSSFESSGFCSRDTTPI-FT 593

Query 551 PTQPTFPFTYKTKEIKNLRLEDGGALRKCLLFVCGQQDVVTLLLMKEECCKDEETIVRIW 610

PTQP FPF +K K L L + +LRKC+LFVCGQQD+V LL+++E C E + ++W

Sbjct 594 PTQPNFPFAFKRP--KKL-LPETDSLRKCVLFVCGQQDMVVTLLLQDEACASLELLTKLW 650

Query 611 ETCTEHLGKLEKQLNFCLESQSSGTATQESEPYSFLCLDAKWDTVKRGGPWGTGDLSSLV 670

E CTE+LGKLEKQL+ CLE+ G A ++EPYS+L LD+ WDT+ RGGPWG+ +L +L

Sbjct 651 EICTENLGKLEKQLHHCLETYPGGGAPGDTEPYSYLYLDSDWDTIHRGGPWGSAELGALT 710

Query 671 SLHRDFTDTPDITEILL 687

HRDF ++ + EIL+

Sbjct 711 YFHRDFQESSSLIEILM 727

Range 2: E=1e-14; bits=77,8

Query 670 VSLHRDFTDTPDITEILLRNTDSAIYGYHCGPSEVFYHEGAGAASGLPLPSDPVGLIQTK 729

+ L R FT+ +I D IYGY CG SEVFYH+ A +GLP P+DP+ + K

Sbjct 836 ILLFRRFTEVAQ-KKIEESCCDGVIYGYQCGKSEVFYHQAANPNAGLPTPADPMSNVPLK 894

Query 730 AKRRLERDHAVVLL 743

A+RRLERDH+++LL

Sbjct 895 ARRRLERDHSIILL 908

Graphical representation

NO PUTATIVE CONSERVED DOMAINS HAVE BEEN DETECTED
