# Supplementary material for: Transcriptome Analysis and Systemic RNAi Response in the African Sweetpotato Weevil (Cylas puncticollis, Coleoptera, Brentidae)
Source: PLoS One. 2015 Jan 15;10(1):e0115336. doi: 10.1371/journal.pone.0115336 (PMC4295849; doi:10.1371/journal.pone.0115336)

**Supporting Information S4:** Sequences of *C. puncticollis* proteins involved in antiviral RNAi.

**Ars2**

>Cp.comp36546_c1_seq2 len=3219

AAATAACAAATTAGAGATGGTTTTTTTTTAACGAAAATATTCGAAAAAATGAACTTTTAAACAATTTTTTTTGCTTCATTTATTCTTGGTTGAGGCTAAAAACCAAAGAAAAAAACAGTTATTGCTTTTTTTCTATAAGGAAGTTTAAAACGATTTAAGGGGGAAAATCCGAACTGAAACAACATTTTACTAAGCCGAGTATATAATTCCTTTTTATAAAACAAATGGATAGACACAATAGCTAAAATGTTACGATTTGTATTTCAAACGTACCACAAAAGAAATCTTCACTTTTCTAAGTAAATATATATGAAACAAATCATACAAACTCTTCCGGTTCCCTCGGCGCGTCCAGATCCCTGTAATGGATGATCGGCCTTCCAAAATCTCCCGGACCGCCCCTTGCCCTCGGTGCGAATCCTCGCGATCTCGGGGCGAATCCCTGATTATAATAGGGCTGCGGTCTCCCGAAACCACCCCCGTAACCTCCATATCCCGGGTGATTGTACGATGGCGGTTCTTCCCTTTTAGGCTGCGGCGCTTCGGCCAGCATTGGTCTTTTAGTATCTTTCAAGTAATTGTTAAAAAACTCCACCTCTTTTTTGACTTCCTCGATTTTTTCGGCGTGTTTATTGAAAATGTGCTTTCTGACGAAATCCGGACCTTTGAATTTCTTGCCGGAAAGTGGACACAACCATTTATCCTTGGCCAATTCCCTGGTGTTTGCCTGTACGAACTTGTCGACTTCCGTATCGACGTCTTTGAGCGCCATACTAATCAGTTTCGTTTCGTTCTTGTCCTTCTCCTCTTTGGGCTTTTCCGGAAGGAAATTAGCCATTTTGGCCTCGACCGGGGGCCCGATAAATTGCGTCATATCGGTCTTGGACGTGGGCGGCGGACCTCTGGCGTGCAAAATGCCGCACCTATTCGGCATCTCGTCTTCGTTCGGATATTCGCAATGGTTGTAATAATCGACCGAGTGTACCACGCGTAGATACAAAATGATCCTGTCCAGTACTCTGATCAGGTTTTCGTCGCGATCGACTGTCGAGACCGCCTCCTGGTTCTCCGTCGACGGTTCCAGACCCAAAAGTTCTTCTTCCTCGGCGCTCGCCTCTTCTATTAAGTAGTCGGTGATGTTGTGCAGTACGGGATTGTTCGAAACAAGCCCGAAATTCTGTTGCGGTTTCTCCTTCTTTTCCCCATCGTCGGGCCATAAGCCGGCCTTGGCGTCCAAATGTAACGTCACCCTGGCGGCGATCCTGATGTCCGACCTCACTACCTGCTTGTGCGCCATTATACCGTTAACGGGCCTGATCCTCCTGCTCAAGTCCCTATTGACGATGGCTCCGAGTTCGCACTCCCGCAACCTTATGTTGTTCAAATTCCAGCAAATCTCTTTGATATTCGCCTCTCGTTTAAAAGTGACCCAGCCGCGGCGCAACCAGCGTCGTTCCGGTTGAGGATCGGCCAAAGCGACCCGCAAGAAACCGTCGTAACGGGAACAGACCGCCTCCACCTCTTGTTTGGTTATGGTGGGCGCCAGATTTCGCAAAAATATCGACGTCGTCTTATGCAATGCTTTGGGCTTTTCCGGTTTGCCTTCGTCCGAAATGGAATGCACTTCCTCGTCCTTTTCTTTGTCCTCTTTGTCTTTCTCTTCTCCGTCTTTATTTTCTGGTTTGTTTTTGCCTTTTTTCTCATCGTCGTCTTCGCTGTCGCTGCTGGAACTCGACGACGAGCTACTTCTGCTACTACCCGAATAAGATCTTTTCCGTTTCTTCCCGTTTTCTTCTTTATTTTCGACGTTTTCCGCTTCTTTGTTCTGTTCTTCGGGTTCTTTCATTTCGACGTCGCCATTGGCGGCGTCCTCTTTAGACTTGTCATCGGTTTGTTCCAAATCTACCACAGCTTGTGTCTCCGAGTTTTCTTCGATTTCTTTATCCTTTTGCGGTTCTTCTTTGTTTTCTTCTTTCGAATCGTCGCCTTCGATTTCACCGTCATTTTCTGCCGGCTCTTCGGTCCCTTCGGTTTTTTTACATTGTTCATCTTCCTTTTCGACCTTTATCTTAGTCTGGGCGTTGTCAATTTCGCCCTCTTCCTTTTGACTGGCGGTTTCCGCCATTCTGGCTTCGCGTGCCCTTTCGGCAGCCGCCTCCTTCTCTTTTTGTTCGCGATATTTCTGCTCCATTTCTTCGTATTCTTGTTCCAAAGCGGCTAGGTCTTCGTCGGTGCCGCCTTCCAGTTTGATGACGACCGTGTCGAGCAATTTAAGGAGGTCGGTGGTGCGCGCGCAATCTACCGTCGTACTTTCGATCTTGCCCGAATTGAGGAGGTCGATAAACACCTCGAGACGCTTTTTGAGGGCGGCCGCCTGCTCCTGCTTCCTTCTGACGGAATCTTCGGGATGGTACTTTAAACGGAACCATTCGTCATCCTTGTGAGCAACGAAAAATTCGTTCAATTGCTGCCTCTGAAACTCCAGCTTGTAATCATTGTACTTTTCTATGGCTTCGCTATCGGAAATGTTGTCGTCTTGAGTAGCGAGGAACGCCTTGAACGACATCATCGGAGGTTGCTGACCTTCCATTTGAGACATGGTTTGTTCTCTGTGAGGAGGTTGATGGTAGGGCGTATAATGGTTGTAGTAACCTTCTCCGTAATAATCATGCCTCATTCGTTTAGCCGTAGGTTCCATACTAGGAGAGTAGCCTCGATCCCGCGGACGATACTCCCTAGGTCTAGGCCGAGACCAAGACTCGCGTTCGACCCATTCGTCTCTGGGCCTCACTACGCGTTCGGCGCCCCTGTAACTGGCTTCGGCACGTTCTCCTCTAAACTTGTCTCTTCGTTTACGATCGTATTCATCATCGCTGTCCCCCATTATGACAGCCTCCTTAGGAATTCGTTCTTCAATTTTTCCAAATTTTTTCCTTCGATGCAGCAAATTCTCGTTTAATTAACTTTAATTACATCTTTTTCGATAAAGACACGTCTAAAATAGGCCAAACTGTGGTCAAGGGACGCATACCATAGATAAAACTGCAGAGACAAGTATCGGTTTGGCTATAACGCGTTACGTAAACCGTACACAATTTTTTGACGTCCTTACTTCTGTTCTAACACCACACTGGAACAGTTCTAGGATAATCCATTCCAGTCATTCTACTGATCTGTCTATCGGATAAAGTATTCGTCGGTTAAAAACATAGTTGGGTG

Protein RF -3: -2884->-2222 and -1624->-320

Comparison with *Tribolium* hypothetical protein TcasGA2_TC003562 (819AA)

Range 1: E= 4e-91; bits= 312

Query 2884 MGDSDDEYDRKRRDKFRGERAEASYRGAERVVRPRDEWVereswsrprpreyr--prdRG 2711

M DSDDEYDRKRRDKFRGERAEA+YRGAERV RPRD+W ER+SWSRPRPREYR RDRG

Sbjct 1 MADSDDEYDRKRRDKFRGERAEATYRGAERVARPRDDWAERDSWSRPRPREYRPGVRDRG 60

Query 2710 YSPSMEPTAKRMRHDYYGEGYYNHYTPYHQPPHREQT-MSQMEGQQPPMMSFKAFLATQD 2534

YSPS++P KR+RHDYY + YYNHY+PYHQ HRE SQ EGQQPPMMSFKAFLATQD

Sbjct 61 YSPSLDPAPKRLRHDYYADSYYNHYSPYHQSSHREPPPSSQSEGQQPPMMSFKAFLATQD 120

Query 2533 DNISDSEAIEKYNDYKLEFQRQQLNEFFVAHKDDEWFRLKYHPEDSVRRKQEQAAALKKR 2354

DNISDSEAIEKYNDYKLEFQRQQLNEFFVAHKDDEWFRLKYHPEDSV+RK+EQ AALKKR

Sbjct 121 DNISDSEAIEKYNDYKLEFQRQQLNEFFVAHKDDEWFRLKYHPEDSVKRKEEQMAALKKR 180

Query 2353 LEVFIDLLNSGKIESTTVDCARTtdllklldtvvikLEGGTDED 2222

+EVF+DL+N+GK+ + +VDC++T DLLKLLDTVVIKLEGGT+ED

Sbjct 181 VEVFLDLMNNGKL-AVSVDCSKTNDLLKLLDTVVIKLEGGTEED 223

Range 2: E=0.0; bits= 625

Query 1624 VHSISDEGKPEKPKALHKTTSIFLRNLAPTITKQEVEAVCSRYDGFLRVALADPQPERRW 1445

+ ++ D+ KPEKPK+LHKTTSIFLRNLAPTITKQEVEAVC RY+GFLRVALADPQPERRW

Sbjct 390 IETVEDD-KPEKPKSLHKTTSIFLRNLAPTITKQEVEAVCGRYEGFLRVALADPQPERRW 448

Query 1444 LRRGWVTFKREANIKEICWNLNNIRLRECELGAIVNRDLSRRIRPVNGIMAHKQVVRSDI 1265

LRRGWVTFKR+ANIKEICWNLNNIRLR+CELGAIVNRDLSRRIRPVNGI AHKQVVRSDI

Sbjct 449 LRRGWVTFKRDANIKEICWNLNNIRLRDCELGAIVNRDLSRRIRPVNGITAHKQVVRSDI 508

Query 1264 RIAARVTLHLDAKAGLWPDDGEKKEKPQQNFGLVSNNPVLHNITDYlieeasaeeeellg 1085

RI+A+V LHLD K GLW DD EKK+KPQQ FGLVSNNPVLHNITDYLIEEASAEEEELLG

Sbjct 509 RISAKVALHLDNKVGLWLDD-EKKDKPQQTFGLVSNNPVLHNITDYLIEEASAEEEELLG 567

Query 1084 lEPSTENQEAVSTVDRDENLIRVLDRIILYLRVVHSVDYYNHCEYPNEDEMPNRCGILHA 905

LEP+ E Q++ +TV+RDE LI VLDRIILYLRVVHSVDYYNHCEYPNEDEMPNRCGILHA

Sbjct 568 LEPTAETQDSATTVERDEQLISVLDRIILYLRVVHSVDYYNHCEYPNEDEMPNRCGILHA 627

Query 904 RGPPPTSKTDMTQFIGPPVEAKMANFLPekpkeekdknetKLISMALKDVDTEVDKFVQA 725

RGPPPT+KTDMTQFIG PVEAKM +FLP + +++KNETKLI+++LKDVDTE+DKFVQA

Sbjct 628 RGPPPTTKTDMTQFIGAPVEAKMTSFLP-EKPRDENKNETKLINLSLKDVDTEIDKFVQA 686

Query 724 NTRELAKDKWLCPLSGKKFKGPDFVRKHIFNKHAekieevkkevEFFNNYLKDTKRPMLA 545

NTRELAKDKWLCPLSGKKFKGPDFVRKHIFNKHAEKIEEVKKEVEFFNNYL+D KRPMLA

Sbjct 687 NTRELAKDKWLCPLSGKKFKGPDFVRKHIFNKHAEKIEEVKKEVEFFNNYLRDPKRPMLA 746

Query 544 EAPQPKREEppsynhpgyggygggfgrpqpyYNQGfaprsrgfaprarggpGDFGRPIIH 365

EAPQPKREEPPSYNHP YGG GG+GR PYYNQG++ RSRG+ PR+RGGPGD+ RP+IH

Sbjct 747 EAPQPKREEPPSYNHPSYGGSYGGYGRLPPYYNQGYSQRSRGYTPRSRGGPGDY-RPVIH 805

Query 364 YRDLDAPREPEEFV 323

YRDLDAPREPEEF+

Sbjct 806 YRDLDAPREPEEFI 819

Graphical representation


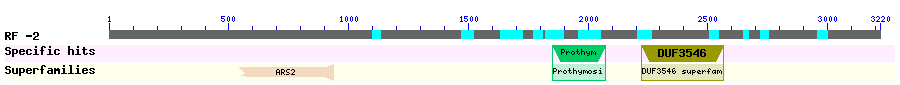


**CG4572**

>Cp.comp36338_c0_seq1 len=1725

TTTTTTTGTATTCAATATCATTTAATATTCTACAAAACAGAGAGCAAATATCAGATTCTCAGTAAAATAGTTACAACTAACAATACCAATGTAACTTCAAAGATATCACACAATATGGAATTTGAGTATTTATTATCATCACTAAATTTGTGGATATGCTAATGCCTCGAATGGAATGGTTTATTTCTGGTAAACCTCGATATCAGATCAAATGCCCATTGAGGTTGATCCGCAGGTACCATATGTCCGGCGTTTCGCACTAAAATTTCTGTCAAGTTGCCCGCTTGTTTCACATAACCCGCCAAGTCGCTGCCTACAAACCACTGAAATCGCGGTGCGATTTTGTACTGGTCAGCACCGTTGAACTTCAAATTTTGTAGAAAATTAACTGTTAACGGATAAGCGACAATAATATCCAATTGGCCATTGTAAATTAACACTCTGTAATTGTTTAAGAGTTCAGAAATCCACGGAGCCACACTCTGCATAACATCTGTCAATAAATTCAATTCGACATCTTGTGCGATCCCGTTAAAAGATGTATTGCCGACGTGAATCGCCGCTCGTATATCGTTACGTTGCACATAGGCCCCCATGAATGTTATTTCGGCATTGTTGGGATCAACAGGGTACAAGAAGTTAAAATAATTATCAAAACCAGTGACGTTTTTAAACAATGATGAATGATTGTTCATATCTCCATTAAGCAATGAGTCGAAAACTTCAAAAGCTTTAGCAAAGTCTTTGTTTTGGATATATTTGATTCCTTCTTGTTCATAATTGTGTATCACTTGTTCAGTATTCTGATCGATCAGTCCAATTTGATACAAATAATCTCCGTACTTGAGTTGGTGCTCAGGATCGCATAAACCATTTCCGATACTCAATCCTTGTAGATTTATTTTAAGTTTGGCCGATGGATTCTTTTGATGTATCGTATACGAAATTGCAGGAACATACTTTCCTCCATATGACTCACCGGCAACGAAAAAGTCATTTTTTTGTATTTCCGGAAACAATGTGAAAAACTGAACTAGAGCAGAATATAAATCTTCTCCAACTTTGGTTTCGTTTTGTGCGTATCCTTTGTTAGTAAAACTGTAACCAGTTCCTACCGGATTGTCAATATAGATCACCGAATGGCTTTTGGTCCAGGAAAAAGGCCTCACTTTTAATCCGTGTTTGGCTTTGACTTTAAAAGGGCCATTCTCGGCAAACAGGCCAATTAAACTGGACGCTCCTGGTCCGCCCTGCAGCCATAAAACCACCGGCGCATTCGCATAGTCAGTCTGAGATGGGAAAAACCAGAAAAACATATTGGAGTCGAACTGTTTGTCCACTGTAAGGTAACCGGCGTAACTCTTTGTTTTCTTAAAACCATTAAAAAACACTTGGGCAGCACTCTGAGCCTCTTTTATTTTGTTCTGTTCAATAAGAGGTGTTAGAAAAAGTGGGACCCCCGGGTCATCATTTAGTTTTTCCGGCTTAATTCTAGGATACACATTGGGAAAAGATGAATTTGCAACAATAATCAATGTACAAAAAACTAAACAAAAATTTAAATATTCCATCACTGCAATTACTTCGCACAACGGAGTTATCACCGTTAACTATATTAATGACGAGTATTTCTTTAAAATTAAATGCCCTTCCGGAAGGTTATCTTCATAGATAAACAACAAATCGGATATCTTTTTGTTTACGTTTAGTTTTTATCACAGACAG

Protein RF -2: -1571->-159 (470AA)

Comparison with *Tribolium* PREDICTED: similar to salivary/fat body serine carboxypeptidase (468AA)

Query 1 MEYLNF--CLVFCTLIIVANSSFPNVYPRIKPEKLNDDPGVPLFLTPLIEQNKIKEAQSA 58

M Y+ F L+ + +++ +FPNVY IK ++ +++PG+PL LTPLIEQ +IK+A +A

Sbjct 1 MHYVVFPAVLLLTFSLNLSSGAFPNVYGPIK-QQPSENPGLPLILTPLIEQGRIKDALTA 59

Query 59 AQVFFNGFKKTKSYAGYLTVDKQFDSNMFFWFFPSQTDYANAPVVLWLQGGPGASSLIGL 118

++V+FNGFK +SY+GY TV+K ++SN+FFWFFPSQTDYANAPVVLWLQGGPGA+SLIGL

Sbjct 60 SRVYFNGFKTIESYSGYFTVNKAYNSNLFFWFFPSQTDYANAPVVLWLQGGPGATSLIGL 119

Query 119 FAENGPFKVKAKHGLKVRPFSWTKSHSVIYIDNPVGTGYSFTNKGYAQNETKVGEDLYSA 178

FAENGPF V +HGLK+R +SW K+HSVIYIDNP GTGYSFTN G+ QNET+VG DLY+A

Sbjct 120 FAENGPFAVMRQHGLKLRKYSWVKTHSVIYIDNPAGTGYSFTNNGFCQNETQVGLDLYNA 179

Query 179 LVQFFTLFPEIQKNDFFVAGESYGGKYVPAISYTIHQKNPSAKLKINLQGLSIGNGLCDP 238

L QFF LFP +QKNDFFV+GESYGGKY PAI+YTIH KNP+AKLKINL+G+SIGNGL DP

Sbjct 180 LQQFFLLFPALQKNDFFVSGESYGGKYTPAIAYTIHTKNPTAKLKINLKGVSIGNGLTDP 239

Query 239 EHQLKYGDYLYQIGLIDQNTEQVIHNYEQEGIKYIQNKDFAKAFEVFDSLLNGDMNNHSS 298

HQL Y DYLYQIGLID N + Y+ +GIKYIQ+KD+ KAF++FD+LLNGD+NNH+S

Sbjct 240 VHQLDYADYLYQIGLIDSNVRSTVKQYQDQGIKYIQSKDWVKAFQLFDNLLNGDLNNHTS 299

Query 299 LFKNVTGFDNYFNFLYPVDPNNAEITFMGAYVQRNDIRAAIHVGNTSFNGIAQDVELNLL 358

LFKNVTGFDNYFNFLYP+DP+N E+ +MG Y+QR+D+RAAIHVGN +F+G +Q+VELNL+

Sbjct 300 LFKNVTGFDNYFNFLYPIDPSN-ELIYMGEYIQRDDVRAAIHVGNATFHGESQEVELNLM 358

Query 359 TDVMQSVAPWISELLNNYRVLIYNGQLDIIVAYPLTVNFLQNLKFNGADQYKIAPRFQWF 418

TDVMQSVAPW++ELL++YRVLIYNGQLDIIVAYPLTVN+LQNL F+ AD+YK A R++W+

Sbjct 359 TDVMQSVAPWVAELLSHYRVLIYNGQLDIIVAYPLTVNYLQNLNFSAADEYKKAQRYKWY 418

Query 419 VGSDLAGYVKQAGNLTEILVRNAGHMVPADQPQWAFDLISRFTRNKPFH 467

V DLAGYVKQAGNLTE+LVRNAGHMVPADQP+WAFDLISRFTRNKPFH

Sbjct 419 VDEDLAGYVKQAGNLTEVLVRNAGHMVPADQPKWAFDLISRFTRNKPFH 467

Graphical representation


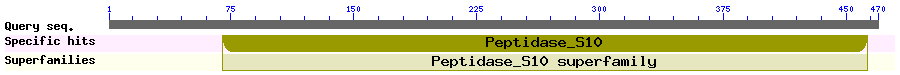


**Egghead**

>Cp.comp38318_c0_seq1 len=2326

ATTATTAAAAGACCTATCTCAATTTAGAATATTTTATTATATTCAAGGCTAAATTATTAACCAACTTAATCACACACACAATTGATAAACTAACCAAAAGTATAATATAAGACATGAGAGGTATGAATTTATGGAAAAGTCTTGGAACTGATAATTGGGAATAAAAATTGCATATTAAATACTACTAGTTCAATAACAATAATAATTAATTGTTTATGAGAACAAGATACGTTTTTAAACGATTCGTCACAGTGAGAAATTTTGTTATTGTAAGAAAAACTTTTATATCCGTTTTAACCTTGAATATTAATTTTAAGCAATATTGCAAACGAGGTTTAATTCCAGAACGTACCTATACAAAATGCCTTTTTAACCTAGTTTTTGATTTTAGGGTTATTTAAAGGTCAAAAAGGTTCCTACTGTGAAGAGGCATAGATAACCTAATTTTTTGAAACTTTTTCCTAAAGTTCGTAATTTTTTTAAATAGCAAAAAATTTAATAATCATTGAAAATATGCTTCCGACTCAAAACAAATTCGACTAAAAAAATCAATAACAACATGAATAAATACTTTGGAACAACGTATAAGTACTTTTATACGGTCACCTCGTGGCCTAAATTTTTATTGACCACGTAGAATTTGTGCTTCTTTCCGAAAATGCCCCACAATACAGCGATATTTTCTATAATCAAATTAAACGGCATAATAACGACCGCACCGAAAATACACAGAAAAAAACCTCGCCAAACCGAATCGATACACATTAAATGACTTTATTACCCCGAATATAAACATATAAATATTAACGGCCCCAATAAACGCTACCAAAAAATCGACTAACGCCGGACAGGGTATCGGAAATTTAGTGGCTAGAAATATATTCGATGTGGACAGAGGTAGAGTAACCCACGAGTAACAGGAACATGCTAAGAGTAATTTGTTTCTAATTGGAATCGATCTCGAGTGTACGACCAGCAAAATACCTTGTATCCAACGTTTACGTTGCTGGATAAAATCCCATAACGTAAAAGGAGATTTTTCCCACATATCGCCCTCGATAAAATTAAAACTATATCCCATACTGTAAGCTTTCATCGCGAAAAAACAGTCTTCCGCTATAGATCCGTCCAAACCGTTATCAAACGAAACATCTTTTTCAGCGGCAACCTGTGTTACGACAAACGATCCTTTCCAACTAAACAAAGGCTTGTGGAACCACAAAAACTGAAGCTTTAATTTGCCCATATCGTCAGTTACTCTAAAACTATCTGCCAGGGTGGTTATCCAATTGACGACTTCCTCGTTCGCGTATGTAATTAAACCTTGTCCAAAATGGTGTTTGCCGTCCATGACAAAGTTTAGGATGCCCCTGATGGAGTTTTCGGTCAGAACGGTCTCTTCGTCCAAATGAACTACCCAGTCGTTATCACTTAAAATGTTAATTTTTTCTTCTAAACAATACTGTAACGCTCTGGCTTTAAACAGAGCTCCCGTAGGTGTCCGGTAATCGGAAGGTACCACCAGTTCACGAACTCTTCGGTGCTGCTCTAATCCAATCGGTTTATCGGACACTACTTCGATTAAGAAGTTCTCTAGTCCTGTATCTAGACACTTATTCATATTTCTATTTACATTATTTTTCACTAATTGGGGAAAGTCACCTCGGGTAACCACCCTAATACAAATAAAGGGCGCCAACAAAGGGGAACCTTTTAGGGACACTTTTTCTGGAAAAGCGTTATAAAAAATAAGCCCGACGAAGTTAAAAATCACCTGCGGCAAGGGCAAAAACGTAACCAAACGTAAAGCGTACAATACACACGCCCCAAAAAAACCATACTCCACCCATGGGTTTATTTCAGATGCCGGTACGAATACACCACCGTTAAACACTCTCAAACCGCCCGAAAATATTTCGAAAAATAATATCACAGTTAGAAACAGGGTGCAGTGCAGGTAATGTTTCGTTTTGCCACTGAGCATTGTGAAAAATCACCCCCAAATTCACAAAACACACCCCTCAAATAACTTTCTTTATAGATCCAGCCAGCTTTGGATTTCGTTACGTCTTAAAATGTACTAAAAATTGTTACAATGTTCCGCACCATCGACGACATAACACCTTCGGAAAAAAATAATAATTTCGCCTGGGCTTTTCGGAGAAAAATACCGACCGATGCGCTCGTGACAAACTATGCGAATGCCGATGAATGCCGAGTCTGACAGGAAAACAAAACTATCGGTTTCGAGGATGAAGACAAGCCGGACGAAGGAATAGGTATCCCAAAAGCTACCCTTAACATGGATGGATGGATCAAAAAGTTC

Protein RF -1: -1984->-695 (429AA)

Comparison with *Tribolium* PREDICTED: similar to conserved hypothetical protein [Tribolium castaneum] (464AA)

Query 1 MLSGKTKHYLHCTLFLTVILFFEIFSGGLRVFNGGVFVPASEINPWVEYGFFGACVLYAL 60

ML+ K KHYLHC LF+ VI FEIF+GG+++ +G FVPA +INPWV YG+ GA VLY L

Sbjct 3 MLTSKAKHYLHCCLFIYVIFMFEIFTGGIKLLDGAFFVPAEDINPWVHYGYLGALVLYLL 62

Query 61 RLVTFLPLPQVIFNFVGLIFYNAFPEKVSLKGSPLLAPFICIRVVTRGDFPQLVKNNVNR 120

RLVTFLPLPQV+FNF+GL +YNAFP+KV LK SP+LAPFICIRVVTRGDFPQLVKNNVNR

Sbjct 63 RLVTFLPLPQVLFNFIGLTYYNAFPDKVVLKASPILAPFICIRVVTRGDFPQLVKNNVNR 122

Query 121 NMNKCLDTGLENFLIEVVSDKPIGLEQHRRVRELVVPSDYRTPTGALFKARALQYCLEEK 180

NMNKCLD GLENFLIEVV+DK +G+E+HR+VRE+VVP DYRT +GALFKARALQYCLE+

Sbjct 123 NMNKCLDAGLENFLIEVVTDKKLGMEKHRKVREIVVPQDYRTKSGALFKARALQYCLEDD 182

Query 181 INILSDNDWVVHLDEETVLTENSIRGILNFVMDGKHHFGQGLITYANEEVVNWITTLADS 240

+N+LS NDW+VHLDEET+LTENS+RGILNFV DGKH FGQGLITYANEEVVNWITTLADS

Sbjct 183 VNVLSPNDWIVHLDEETLLTENSVRGILNFVGDGKHQFGQGLITYANEEVVNWITTLADS 242

Query 241 FRVTDDMGKLKLQFLWFHKPLFSWKGSFVVTQVAAEKDVSFDNGLDGSIAEDCFFAMKAY 300

FRVTDDMGKLKLQF FHKPLFSWKGSFVVTQV AE++VSFDNGLDGSIAEDCFFAMKA+

Sbjct 243 FRVTDDMGKLKLQFRMFHKPLFSWKGSFVVTQVGAEREVSFDNGLDGSIAEDCFFAMKAF 302

Query 301 SMGYSFNFIEGDMWEKSPFTLWDFIQQRKRWIQGILLVVHSRSIPIRNKLLLACSCYSWV 360

S GYSFNFIEG+MWEKSPFT WDFIQQRKRWIQGILLVVHS+ IP+RNKLLLACSCYSW+

Sbjct 303 SKGYSFNFIEGEMWEKSPFTFWDFIQQRKRWIQGILLVVHSKDIPLRNKLLLACSCYSWL 362

Query 361 TLPLSTSNIFLATKFPIPCPALVDFLVAFIGAVNIYMFIFGVIKSFNVYRFGLARFFSVY 420

TLPLS SN+ LA+KFPIPCP ++DF+ AFIGAVNIYMF+FGVIKSF VYRFGL RF V

Sbjct 363 TLPLSVSNLVLASKFPIPCPPIIDFICAFIGAVNIYMFVFGVIKSFTVYRFGLGRF--VL 420

Query 421 FRCG 424

CG

Sbjct 421 CLCG 424

Graphical representation


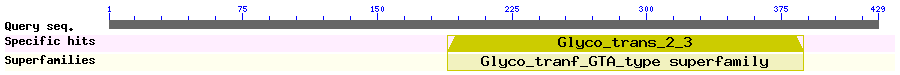


**ninaC**

>Cp.comp38683_c0_seq2 len=4543

CTTTGCAATTCTTATTTATTAATAAGTATCCAGTAAAATATATTTGAGTGCTTTATTATTAAGCGTGCCAAATAATCAAATTTATAAATCAACTTCAATGCCCTGAATAATTAATCCGGGAATTAACTCCATAGTCACCGGTCTGCTGGAAATGATCACCGCCTCGTCAGCATCGCCGTTGAACAGCGCTTCTTCTTTATCCTTAGTAAGGGAAAACTTCCTGACAGCTTCGAAAGTCCTCTTAATCGACTCCCTTTTGATGGAATCCCTTTTGAAATTCGTCTTCCTCAACATTCCTTGGAAATTGAACGGCGGATCGTCGTCGGATGCGGCGTTGGTCTTCGCTCGACTGCGCAACTCCGCTATAGGATCGGCGTTCTTCGGCTTAGATGTCACAGAGCTAGCTTTGAAGTATTCGAAGTCTTTGTTAGCTGGAGCGGAGTTTATCGAGTTTTCTGACTGTGGTTGCGTTTTTTTCAACTGAATAGACGTGAACGATTCGGTTGATATCTTGTCTAGAGATTTAGTATCTTTGTTGATTTTTTCTTTCACTTCTTTAGCTTCATCTGCTTCGACAGTTGGGCTAGGTATTATCGGTAGTGGTTCGTTGGGATCCCTTTTGTAGGGGATATTTGCTAACTCATTTGAAGTTTGACTGGTCGCTTTTTTGGGCAAGTTAGCGTCGTTTTCTCTCCAACGATATGGACTGTCCCAAGGCTCGCCTCTTTCTTGTAAGCTCCCCACAGTAGTTAAAGAGTCACACAGAGGAGAGGTATCGTAAAACGGAATTTCGTCCAGTCTGAAATGTATTTTAAGCACTGCCGGTTTTATAGCGCCCAACCAATTGTTTACTTGGGCTCTGGAGTCGATGTATTTCAAGTCGACACCTTTTTCAACATTTTGGGTCTGGAAGAAGGCGTTTTGATTGTAAAGATGGACCTGTTGGGAAAAATTGAAAAAATCGTGCAGCCTGCTCGCTCTGTATTGAACCAGGACGTGGAATATCGAAGGGGATTTCCATTTTTTCGCGTACGGCATAATGAATTTGCAATCTTCTTCGGTGAGATGATCGAAGGCGCTCTTGGAATCTTTCGCCATCGATCTTCTGTAGGCTTTTTGTATTATTTCTGCTGCTTCTTCTGGCGTCATGCTGCTTCTCCTTCTACCCTTACATTTCTGAACGCATTGATTCTCCTCGTTTTTCGCTTTCTTATTTAACCGACACTTTGCCAGAAAGCCTCTCATAATACTCTGAATTTTGACGATTTTTTTCACTTGAGTCTCGTACAGTCTAGCGAGATACTCTTCGTTGTAGTATTTCAGGAAAACCTTGGATTTTCCTATAGCCCATCCTTCCATGTTCAATCTTATAAACAACAATCGGCAATTTTCCTTGGTGATTTCCACGTTCTCGTCAAAATCGAACGCCAAAAATTTGTACCGGCGAAGAAATTCGGAGAAAGTTATCCTCTGGGGGTAGCCTTTTTGTCGAATTTTTGCCGTCTCTGTTACCGCCAATGCGCGCAACTGTTGCTTTACCAGTTCCCTATGAAAGTGCCGAGGTCTACCCTTAAGGTCCGACCTAACGCACCTGATGAAATGGGTCCCCCCCGAGCTACTACCCACCGACAGTTCTTTCAACAGATCGAGACAAAACGCACGGTATATGGTTGCAGATGTGCGCATTCGTTTCACTTGAGAAAACTGATCGGCACTACCGATCTTTTTCTCCCTTCGCTGTCGATCGAAATGGATATTTAAGTTCCCTTTTCTGTCCAATTTGTTCGTAAACAGTAACTTTATTATAGGATTCTCTGAATCTCTTAGAGTTTCTATCAATTCCGGCGGCAAAAAATCACGGTTTTTGTCAGGCATTTCGCCGACGCAATAGGGAACGATTCCCGTGTAATGCGCGACTGCGAACTCAGACGAGCCTGTCACCATAACTCTGCTGGTCTCTTGCTTCTGGACATTCACCATGACGTATCGGGCGTCCAAATTTTTCCTGGACGCTTCATCGATGATGCACAAAACTCCGTCAGGTTTGCTGAGCAACTGGTTCAACGTCTTTCTATTGTCGACATAATCAAAGTGCTCGACTTCTAACTCCTCGGTTTGAAGATCCTGAAGCTCCCAGGCAAATATCCGCTGCACGTAATGGTACTGAAGTTGTTCGTTAAGAGCGTTGACAAAAAACTGAGCCAAATGATTCTTCTTGAAGCATTCGAACCCGAAGTAGTCGAGCAACTTTATGGTATACCTTTCTCCGAATATTTGCTTTCCGATTGCCAATTTATCGTTAATTACACCAACAACGTAATCCACAAGTCTGCAATACAGGTTATTGGCGAGTACATCTCTAGCGTCTCTAGCCTCATCGGCGGTACTCTTTTTTCTGATAACGTTGCCGTGTTTCACCAAACAATAATTCGTCAGAGCCCAACTAAACTTCTTGTCGTCGATCTCCAGGAGATGGGCGACGTTCTCAACGAACTCTTTGTTTTGGATTGTTGCTGACCCGTCGTCGTCATTTGCCTTAAACCTCACTTCGCCCAAGTTCAAAATGGCGGCTATCGCGCTAAAGAACGTGGTGGTCTGCTCCTCGTCGAACTCGTACTCCTCCAAATAGCTGAAGATTTTCTTGTACTTGATTATGTTTTGTTCTACGTTGTCTCGAGGCCGTTTAGAATTAGATATATCCTCCACCACTCGCAGATATCGGTAGTTTCTCTCGGAATTCAGCTTGTATTTTTCAATGGCTCCGTTATTGACCATTCCGTCGTACAGATAGTAGAATATGTGAAAATTGCTTTGATCCATGTCCACAGAAGATACTCTCCATTTGTCCAGACAAGTCACCTTAAAATCGGCAGCAGTGAGCTTCCCCGTCCGACCCAAAGTAACGATCGTATTCAACACGCATCTGGTGGAGTAATCGTTGGAAGGAGTGGAAGCGTGTGTGAGCGAATGGATCAATTTGATACCATTCTTAATCCTATCGGAACTTACGGGATGATTTGACGCTAAATAGAACAAATGGTCTACCAAATGCAAATAGTTATTGGTTTTTCCCGACCCGGATTCGCCGGTCAACACGATTTGCTGCGGCATTTGGTGATGCAAGACGTTCTGAAGAGCCGTGTCGGCGATTGCGTAAATGTGCGGCGCGTTGTCTGATCTCGATTTGCAGACGTATTTGTTATGAAACTCAGATCCGTATATATTAAGCTTCTCATTTGGATTGATTGCCAAGAGAATATCCCCAATGAAGCTGTAGAATTCGCCTTGCTTGGCTTTAGTCTCCAACAAATCTAGAATTTTGTTTTCGGTAATTTGCTCGATATCAGTCAAATCTTCCTCGACCATTTGCTCCATTTTTCCGTCAATGCGTCGTTTCAGATATCGGCCTTTGACGGCGACTTCCGGACATCGTTCTGGAATCCCAGATGCGCCGATATCTTCGAGCAGGGATTTAATTTCTAGAGTTAAGTGGTAATTATTCTCTGGAATTTGCCCCAGGAACGGATGGTCGATTACTTCCATTATGTACGGTCTGTGCTCATAATTCTTTACCAGACTCTCGTTAATAAAGTCTCTAAAATTCTCCGACCAGTTACTGATCTTTTGCAATGTGGGCGGTGGATTTTTCACAATCTGAAATAGAGCCCTGGTCGGATGCATGTTTTGGAAAGGAGCTACGCCTTCGGCTATTTCAAGAGCGGTAATGCCAAGAGACCAAACATCCACCCTGTTGTCGTAAAAACCAGTTTCGTTATTAGGGTTGGCCGTTACGACTTCAGGCGCCATCCAACACGGAGACCCTACACATTCAGACACCGTATCATCGCGACTCTTCAGCCTCTTTGACATGCCAAAGTCGCACAACTTTATCTCCCCTTCTTTCGTTAAGAGTATGTTGCTAGCCTTTATGTCTCTGTGGATGACGTTGTGCTCGTGGAGATAAATTATGGCCTTGACAAGTTCTTTCAGAACGTACGCGATATGGTCTTCGGACATTCGTCTATGTTTCAGCAACAATCCCTTTACCAGATCTATTACTGTGTTGCCATCACATTGTTCTAAAACAAACCATATTTCGTTATCTCGTTTAAATACTCCGTAAAAATCCACCAGGTAAATGATGTGGCTGAAGTCTTTAAGGATTTCGTATTCTTCTTGGATAAATTCCTTGTTTTCTTGGTCCAACTTTTGCGCCTTGATGGCGACCGCTTTTTGGCTTGCTTGGTTGTCGAACGCCCTGTATACTTTACCAAACACACCTGTTCCCAAAATATCACCGAAGGTGTACCTCTCGCCCGGATCTGGCGAACTGTCCAATCCAGTTTTCATTTTTACGAATTTGAATTTGAGAATGTCGGAATGACTCTTTTTAAGGGAACTTTTAACGTTGTTTTTTTAATCACGTACCAATCTATGCGACTAAAGCATTTGTCGGTTAATTTTTTCCTTGGCTCATTAAATTTCATGGGTTATATTACGGTCCCGGTTAATTTCGTAGCGCGCACTTAATTCTCGGAAGTTA

Protein RF -2: -4350->-82 (1422AA)

Comparison with *Tribolium* PREDICTED: similar to myosin IIIA (1109AA)

Range 1: E= 0.0; bits=1112

Query 4338 LDSSPDPGERYTFGDILGTGVFGKVYRAFDNQASQKAVAIKAQKLDQENKEFIQEEYEIL 4159

L P PGERY D LG G FG V+ A D QA K VAIK QK ++ +++IQ EY++L

Sbjct 3 LKELPSPGERYLVEDCLGVGAFGSVHSARDTQADNKQVAIKVQKHTKKFEKYIQHEYKVL 62

Query 4158 KDFSHIIYLVDFYGVFKRDNEIWFVLEQCDGNTVIDLVKGLLLKHRRMSEDHIAYVLKEL 3979

KD S LVDFYG+F++++++WFVLE C V+DLV+ LL K+RRM E+HIAY+LKE+

Sbjct 63 KDLSWHGNLVDFYGIFRKEDDVWFVLEICSSCCVMDLVQNLLDKNRRMREEHIAYILKEV 122

Query 3978 VKAIIYLHEHNVIHRDIKASNILLTKEGEIKLCDFGMSKRLKSRDDTVSECVGSPCWMAP 3799

VKA I+LHE+ IHRDI+ SNILLT G++KL DFG S L ++CVGSPCWMAP

Sbjct 123 VKAAIFLHENCCIHRDIRGSNILLTNNGDVKLGDFGFSCFLNDVLGGTNDCVGSPCWMAP 182

Query 3798 EVVTANPNNETGFYDNRVDVWSLGITALEIAEGVAPFQNMHPTRALFQIVKNPPPTLQKI 3619

EVVT Y NRVDVWSLGITA+E+ +G AP+Q+M P+R LFQIV NPPPTL +

Sbjct 183 EVVTCKRTKRN--YGNRVDVWSLGITAIELGDGTAPYQSMPPSRILFQIVTNPPPTLYRK 240

Query 3618 SNWSENFRDFINESLVKNYEHRPYIMEVIDHPFLGQIPENNYHLTLEIKSLLEDIGASGI 3439

NWSEN+ DFINE LVKN EHRPY++EVI+HPFL Q+PENNYH L

Sbjct 241 FNWSENYIDFINECLVKNAEHRPYMVEVINHPFLQQVPENNYHCGL-------------- 286

Query 3438 PERCPEVAVKGRYLKRRIDGKMEQMVEEDLTDIEQITENKILDLLETKAKQGEFYSFIGD 3259

DG +E+++ EDL ++ + E ++ LLE + K G+F +FIG+

Sbjct 287 ------------------DGGLEKILAEDLASLDSLQEEDVMKLLEARFKSGQFQTFIGE 328

Query 3258 ILLAINPNEKLNIYGSEFHNKYVCKSRSDNAPHIYAIADTALQNVLHHQMPQQIVLTGES 3079

ILL +NPNEK +IYG EFH KY KSRSDN PHI+AIAD+A QN LHH + Q+IVL+GES

Sbjct 329 ILLILNPNEKKDIYGDEFHRKYQMKSRSDNEPHIFAIADSAYQNALHHHISQKIVLSGES 388

Query 3078 GSGKTNNYLHLVDHLFYLASNHPVSSDRIKNGIKLIHSLTHASTPSNDYSTRCVLNTIVT 2899

GSGKT N+ HL++HL YL N ++ RI N +KLIHSLTHA TP N+YSTRCV +

Sbjct 389 GSGKTTNFFHLLNHLIYLGQNDNINLQRIVNAVKLIHSLTHALTPINNYSTRCVFKVDIK 448

Query 2898 LGRTGKLTAADFKVTCLDKWRVSSVDMDQSNFHIFYYLYDGMVNNGAIEKYKLNSERNYR 2719

G TGK++ A F V +EK++++S +

Sbjct 449 FGNTGKVSGAIFNVF-------------------------------QLEKWRVSSTDRW- 476

Query 2718 YLRVVEDISNSKRPRDNVEQNIIKYKKIFSYLEEYEFDEEQTTTFFSAIAAILNLGEVRF 2539

+ + E++EF++ + T S +AIL LGE+ F

Sbjct 477 ---------------------------MLKFFEDFEFNDSEIDTILSITSAILILGEMSF 509

Query 2538 KANDDDGSATIQNKEFVENVAHLLEIDDKKFSWALTNYCLVKHGNVIRKKSTADEARDAR 2359

D + +KE VE +A LL+ID KF WAL NYCL+K +IRKK+T DEA+ R

Sbjct 510 VEADLESG----DKECVEKIAQLLQIDPCKFHWALANYCLIKKDVIIRKKNTEDEAKSVR 565

Query 2358 DVLANNLYCRLVDYVVGVINDKLAIGKQIFGERYTIKLLDYFGFECFKKNHLAQFFVNAL 2179

D LANNLY RLVDY+V IN++L+ G++IFGE Y++++LDYFGFECFK+N+L+Q FVN

Sbjct 566 DALANNLYLRLVDYIVNTINNRLSAGRKIFGETYSVQILDYFGFECFKENYLSQLFVNCF 625

Query 2178 NEQLQYHYVQRIFAWELQDLQTEELEVE-HFDYVDNRKTLNQLLSKPDGVLCIIDEASRK 2002

NEQ+ Y+Y QR F WE D++ E+L +Y DN+ L++LL KP+GV IIDE S+

Sbjct 626 NEQMHYYYTQRNFYWEYLDMKDEDLNFNTSSNYYDNKSCLDELLGKPEGVFSIIDEVSKM 685

Query 2001 NLDARYVMVNVQKQETSRVMVTGSSEFAVAHYTGIVPYCVGEMPDKNRDFLPPELIETLR 1822

N + ++V+ ++ + V V G+ +FAVAHYTG V Y ++ +KNRDFLP E+IETLR

Sbjct 686 NQNEKHVINFIENSDLKFVKVVGNLDFAVAHYTGTVTYKGCDICEKNRDFLPAEVIETLR 745

Query 1821 DSENPIIKLLFTNKLDRKGNLNIHF-DRQRRE---KKIGSADQFSQVKRMRTSATIYRAF 1654

S+NP +KLLFTNKL+R GNL + DR + + KK+ + +Q+SQ+K + TS ++A

Sbjct 746 LSDNPTVKLLFTNKLNRTGNLIVDAPDRSKYKFTSKKL-THNQYSQIKHLTTSLRKFKAL 804

Query 1653 CLDLLKELSVGSSSGGTHFIRCVRSDLKGRPRHFHRELVKQQLRALAVTETAKIRQKGYP 1474

++LLK+LS G THF+RCVR+DL P++F LVKQQ+RAL V ETAK+RQ GY

Sbjct 805 GVELLKDLS----KGCTHFVRCVRTDLHQVPKNFDCGLVKQQIRALEVVETAKLRQNGYS 860

Query 1473 QRITFSEFLRRYKFLAFDFDENVEITKENCRLLFIRLNMEGWAIGKSKVFLKYYNEEYLA 1294

R +F EFLRRYKFLAFDF+ENVE TKENCRLL IRL +EGW +GKSKVFLKYY EEYL

Sbjct 861 YRTSFHEFLRRYKFLAFDFNENVETTKENCRLLLIRLGVEGWDVGKSKVFLKYYVEEYLT 920

Query 1293 RLYETQVKKIVKIQSIMRGFLAKCRLNKKAKNEENQCVQKCKGRRRSSMTPEEAAEIIQK 1114

RL+ETQVKKI+KIQSI+R FLAKC + K +N+ +C+ + MT EEAA IIQK

Sbjct 921 RLFETQVKKIIKIQSILRRFLAKCLVTKHLQNKGEKCILGHVQAEKYRMTEEEAAIIIQK 980

Query 1113 AYRRSMAKDSKSAFDH----LTEEDCKFIMPYAKKWKSPSIFHVLVQYRASRLHDFFNFS 946

AYR S K S F L EE C FI +A KWK+ ++F +L Y++ R D FNFS

Sbjct 981 AYRESTVKKSYQDFAEDYKILDEETCGFIRKFALKWKNNTVFRILFLYKSVRHQDCFNFS 1040

Query 945 QQV 937

QQ+

Sbjct 1041 QQL 1043

Range 2: E= 6e-10; bits=63,9

Query 432 ANKDFEYFKASSVTSKPKNADPIAELRSRAKTNAASDDDPPFNFQGMLRKTNFKRD 265

AN+DF+Y K S +T K K DP+ EL+ + AS+++PPFNFQGMLRKTNFK++

Sbjct 1044 ANEDFDYIK-SPLTKKLKRIDPVTELKQIGQ-KTASENEPPFNFQGMLRKTNFKKE 1097

Graphical representation


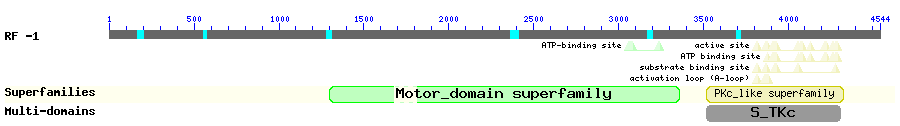

Supplement: S4 Supporting Information — (DOCX) [file pone.0115336.s004.docx]
