# Supplementary material for: Transcriptome Analysis and Systemic RNAi Response in the African Sweetpotato Weevil (Cylas puncticollis, Coleoptera, Brentidae)
Source: PLoS One. 2015 Jan 15;10(1):e0115336. doi: 10.1371/journal.pone.0115336 (PMC4295849; doi:10.1371/journal.pone.0115336)

**Supporting information S5:** Sequences of *C. puncticollis* nucleases.

**Snipper = Eri1**

>Cp.comp37539_c0_seq1 len=2626

ACAAATTTTATTGAACGTTTATAATCCTAAATAATAATAGTACATTATTAAAATCATGTCAAATACGACCCAAAACTTGCCAGTGTCACGCATTCTCAAAACAAAAAGTGAGCTAGATGACGAACCAAAAAAGAAATCCAGAGAAGACTGGCGTAAGGCGAAGGAGTTAGAGGAAGCTAGAAAAGCTGGTACCGCGCCAGCAGCTGTTGATGAAGAAGGTAAAGATATCAATCCTCACATTCCTCAGTACATATCTAACGCACCATGGTATTATGGTACAAGTGGACCTACCTTAAAACATCAAAGGCCCCAAGAAGAAAAACAGAAACAGTATTCTGGTATCGATGAATGGTATAAAAGAGGTTTGGATACGACAAAAATTGTAACAAAGTATCGGAAGGGGGCATGTGAAAATTGTGGTGCTTTAACCCACAAGAAAAAAGATTGCATGGATCGACCTAGAAAAGTCGGTGCTAAGTATTCTGGTACAGATATATTACCAGATGAATTTATACAAAAAAATCTAGCTTTAAGTTTTGACGGCAAAAGAGACAGATGGAGTGGTTACGATCCAGGGGAACATAAATCGGTTTTAGAAGAATTTCAAAAAGTGGAGGAAGCTAAAAGACAATTGAAAGCAGAAAAGCTTGATGCAAATCAGTCAGATGAAGATGAAGATCAGGATGAAGATAAATATGTGGATGAGGTGGACATGCCTGGTACCAAAGTGGATAGCAAGCAAAGAATTACAGTGAGAAATTTGCGCATCAGAGAAGATACTGCCAAATATCTGAGAAATTTGGATCCAAATTCAGCTTACTATGACCCTAAAACTAGGTCGATGAGAGAAAATCCCAATCCTGGGGTTGATGACACGCATCAAGGAGAGAATTTCATTCGATTTACTGGTGATACCAAAGGACATGCCAAAGCACAGTTATTTGCTTGGGAGGCACACGAAAGAGGTGTGGACGTGCATCTGCTTGCAGAACCTACAAAGCTGGAACTTCTTCAGAAGGAGTATGAAAAAAAGAAAGAACAGTTCAAAACCAAAGTGCAGTCAAGCATTTTGGACAAATATGGTGGAGAGGAACACTTAAAAGCTCCACCAAAAACATTACTTCTTGCACAAACTGAAGATTACGTGGAATATTCTCGATCAGGAAAAATAATTAAAGGCCAAGAGAAAGAAGAAATTAAATCAAAATATGAGGAAGATGTTTATATTAATAATCACGCAAGTGTATGGGGATCATATTGGAAAGGTGGCCAATGGGGGTACAGATGTTGCCATTCATTTGTAAAGAATTCTTACTGTTTGGGTGAAGCGGGAAAAATATCAGAAAACACACTTGCGACGATGACTGACGAACAAAAGGAACCCACACATTCAGAATTAAATCTCAAGGACAAAGAATGTAGTGAAAGTTCAAGTTCTGAGGAGGGAGACGATATTAGAACGAAAACAGAAAAAAAGAAAAAGAAAAAACATAAGGACAAGAAACGTAAAGTCAAAGATAAACTTAAAGAAGCTCTCGAGGCTGAAGAATTGCATCAACAAAAGGCTGAAGCCATATTAAGGTTAGATGAAAGAAAAAGACCATACAATAGTATGTTTGAAGTCAAGAAACCTACCGAAGAGGAAATGGAAGCTTACTATCTCAAGAGAAAAAGAGAAGAAGATCCTATGAACCAGTTTCTATAAATTATTACAAATAATTTATAAAAAAATAGATTTACTATTATCAAATACAATTACATGAACGATGTACAAACAATATTAATTTTAATAATTATTTAATTAAAAAAAAACATTATTTTGGTTTTGATATATCTTTTGTTATCCTTAATTTTGCACCATCACTTATCATTTTGCTCACTAATTTAGCAGTATTCCGAGCATCGTGTAAGCCACAGTGCGGAGTACCTTCAAATGTTAGCCCAACTTCACCTAAGGCTCCAAGAAGACCGTTGGGCTTTATATTATAATGTTCCACATACAAAGTACGTATATCAATCCATCTGTCAAACATATCGTGTCGAACAATGTTTTTTCTTCCACATTCCTTTCTTAGGCAATTTCCTAAATCCCAGTCGGACCATGTTGCAAACACACAACTTTTGCAACATGATGTGACTGGATATTTCAGTGATATATTGTATTTAAGAATTTGTTCGTTCAGCCATCTGTGGAATAGCACAAGACCTGTTTTTAAAGGAACACCCTCGTCAACTTGTACTTGTTGAATACCAGTTAGTTCTTTACAAAAATTGCTTAATTTGGGCCTTTCGGTAGGCATGATATATTGCTGAAATTCCGAGATCATTTTATCATTCCTCACATCATACAACACGCACGGAAATTCTATTATTTCTGATCGTCCTTTATTATAATCGGTTTTACTCCAACAAGTTGCTTCAAAATCCAATACCAACAAATAGTCGAAGCACTGCTTGTTGCGTTTTGAACTTTCGTTCTCCACGAATATTGTTTCAATGCAGTTTAGCCTTCTTGCAAGTTCCAACGTTGTTACTCCAACTTTATCCATAATACATTATAATTTAAAAAACATTTATCCAGTATATTTTTAATGGTCTAAAATATTGTCTTCATCTTTTATCTATG

Protein RF -3: -2549->-1815 (244AA)

Comparison with *Tribolium* Snipper [Tribolium castaneum](232AA)

Query 6 VTTLELARRLNCIETIFVENESSKRNKQCFDYLLVLDFEATCWSKTDYNKGRSEIIEFPC 65

++T ELAR+L +E I+ +++ Q FDYLLVLDFEATCWS D K +EIIEFP

Sbjct 1 MSTRELARKLGALEVIYSTAKAT--TPQPFDYLLVLDFEATCWSNGDPRKNPAEIIEFPV 58

Query 66 VLYDVRNDKMISEFQQYIMPTERPKLSNFCKELTGIQQVQVDEGVPLKTGLVLFHRWLNE 125

VLYDV+N K+I+EFQQY+MP E PKLS+FC ELTGIQQ QVD GVPL+ L+LF RW+ E

Sbjct 59 VLYDVKNAKIIAEFQQYVMPVENPKLSDFCTELTGIQQHQVDNGVPLQACLLLFSRWVAE 118

Query 126 QILKYNISLKYPVTSCCKSCVFATWSDWDLGNCLRKECGRKNIVRHDMFDRWIDIRTLYV 185

++ Y++ + K+C FATWSDWDLG CLRKEC RKNI M+ +WIDIR L+

Sbjct 119 KMSLYDMDFPNGESQATKTCAFATWSDWDLGTCLRKECIRKNIRIEKMYRKWIDIRALFK 178

Query 186 EHYNIKPNGLLGALGEVGLTFEGTPHCGLHDARNTAKLVSKMISDGAKLRITK 238

+ GL GAL E+GLTFEGT HCGLHDARNTA+LV KM+ G L++T+

Sbjct 179 RYIRRPFIGLAGALAELGLTFEGTEHCGLHDARNTARLVGKMVDKGVVLQLTR 231

Graphical representation


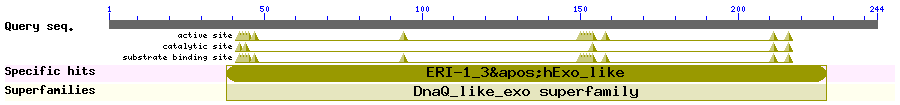


**Nibbler**

>Cp.comp36632_c0_seq4 len=3141

GGTAAATATTGTTTCAAGTTGCGTAAAAGTGTCTAAAAATCGATAATGTTTGAACATATTAAACTGTTTTGCTGTAATAGTTACGCATTAAGTCGACAATGGGCGACGATCTTATGCAATCTGGTCCAACGTTACGTAAAAGAAGAAGAACTGGACTAATCTGTCATAAGAAGAAGAAACTCAGCGGTTGTAGTCGTTTTGGAGGCTTTTTTAACTGTTTTAGTCGTTTTAGGATGTTTTTGATCGTTATATCGTCCGTTTTAAGCGTTATTAGAGTGTTATTCGTGCTTTGCTTAAAGCGGAGTCAAAAAAAGTCCGGATCAAGATTCAAAGTTCCTCAGTTTGAAAGGTGGCGGGTATTCGAAATAAAATCGCCAACTGGAGTTTAAAAAATGTAATAGTCAAATAAAAGTTTACCAAAAATACAAAGGTTAAGTATTTATAAAGATATTTTATTACTGCACGATGCCCTGGAGTCGGCCGTTCAGTACCCGATCGAAATGGCTGCCGTCGTAATAAACTTTGCCGCACTCTTCGCATATATAGAACACTTGGTAATTGTAAATGACCGGCCTGGGTATTTGGTCGGCCTTAATCGTCACGCCGAGCCTCGTCAAGCGAGAGTCGACGTCGATCCTGTCGGTCAACTCCCACTTGCGAGACCCGGAACTGTGACCGGGCATTTCGTCGTCCAAATCTTCGTCGCTCGTAAATCCGGTCGATTCGTCTTCGTAGTACCCCCCGGGGGCGTAATTAACCATCGACGGACCGCCGTTTAACGCCAGCATCGTCGACTGCGGTACTCGAACGAAACTGTTCCCGTTACAAGCCTGACACCGACTAAAAACGTCGTCCTTAGTAACCGTCACTTTGTAATACTCCAAAACTTGTTGCAGTTGTTCGTCGACGTCGTCCGATATGATTTTCAAACAATGCCCGGACGGAACGTAACCGTTCAACATTTTAAACGGTCCTTTACGGGTCAAAATGTACCGTCTCTCGTTCAAATGGTACTTGACGCACTCCTTGTGGTCGTGGTGGTTCTCCAAGATGGCGGTGTCGATGCCGCACCGTCTCAAATTCTTGCCTAAACCTTGGAGCATGGTATCGCATACCACTTTCAATTGATCGACCCGTATCGATTCCGGATGCGGACTGGGCGGTTGCGGTACGTCCTCCTCCTGTTGGTGCCTCTTTTTGTTCGGTTTTTTCTTGACCGACGGTTCGTTGTTGGCCAAACCGTCGCAGATTTCGTAAAACGGACAACCGGCCGTCTCGCAGCACTGTTTCAGCACGTCGTAGACTTCGACGAGGCAGTAAGCGTCGAGGGCGGCGTACCCGATCTGGCTCTGGCGCAACGGCCTGTTTTCCCAATTCGAAAACTGCTCGGATTTGTCCAGAGGACGGCCCAGGCACGCCTGCACGAGCGTACTTAGGCTGGGACCGCCGGTTTGTACTTCGTGTGGTAAGGTGACCTTCGGGTACTTGTCCAGGTGCTTCCAGAGCGTGCACAAGTCCAAGAAACCGACCCGATTGGTGGAAAAGTTAAGCTCGGGAAGTGCTTGTCGTATCATGTGAAAATCGCTGGTCAAACTAAAACCTAATTTTAAAATATCACAATTGTTGAACAATTTCCGGCCGAGCTCCTGCCACAACCGGGGCTCCTTATTCGAAAGGTTCACGATATCGAGCACAAAAACCGTTTCCCTAGTAGAAATTTGCATCAGAGCCAGCTCGTTCGCTTGACTGAGGAAACTGGGCTTCCATTCGCAATCGATCCCGACCATATCCGCGCCCCGAAAGCCAAATTCGAGCATATTCTTAAACCTGGCGGCCGTATCGACCAAAACAATCCGCTCCAACGGCAACGCCAGTTTGTGATAGTCGACACGATTGACCGGTTCGTCGTCCCAATCGTCGGTCGGTGCCGTATCCGACGATCCGTTCTCCTCCAACGCCCGTACGCTGTACGGCCAGTCGTTTTTATCGATATTGTAAAATCGAGCCCATTTTAAAGCTTCGGCGAACTCCCCGTACTGCGCCACCATCTGGACCACCTCCTTTTGTAGGGATTCTTCGTCGCCGACCGCTTCCTGCACCATTTCTTTCCAACTCTCGTCGCTAAAAGTATTGTCGGTGAATCGTTTATGAATCAAAAACTTCAAAGCGCCCTCGTTCCTTTTCCTGTTTAAGTTCGGCGTCAAATCCGGCGACATTTTGAACATTTTAGCGAACCTAGTGATCGTTTTTTTCCACGGTTTCGAGTGTACTTTGTCGTACTTAACGCCCGGTATGTTCCGAGACTCGATGTACGGCAACACGGTCTCGCCGACCGATATCCGGCTTAAAGCCGCGTCCAAAAACTGTACCAAATCGACCTGGTGGCGCGAACTAGAAGCTAAAAATTCGTCGACGCTACAAAGCTTGTCCTGCAAGACTAAAGGCACGAGAAAATCGTCCACGGTGAATTTGTCGTGCAGATTGAGCATCGTCGCGTACTGACACGCTTCTTTGTATTGTTTATTTTCGATCAGGCATCGGACGTTATCCAGAAAAACGTCCGGTTCGCGGGCCATTTCGTAAGCGTCGAACACCAGTTTCGTGAGCGCTTGCGAATTCTGTCTCGTGATCAATTTGAAAGCGTCCAGTTTGATTTGAGGCACCAGAAGGTGTACGATCATGTGTCGGTGTCGTCCGCTCCACTGTTTAAACTCTTCGGCTATAAATATCGGCAGACTTTTGTTTTTGGCGTTGAAAAAGTCCTGGCAGTTGTTCAGGAGGCGGAGAGTTTGCTCGTACGGGTTCGAGCACGTGCCAAAG

TACTGTTCAAGTTTGGCGACGACCGGAGGACTTCGTTTCACCATGTTGTACACGCTCTTCAGTTCGTCGAAAAAAGCCGTATCGCCCTCGCTCAAAGTCAAATTTAAACTAATATTTTTACAGCATTTATTGTCATGACTTGATATGATTTTTCCCGGAGGTTTCGGACTGGAATGAACCGGGCTGTTGAACGGGCGGCCCCTGCCCATGATGTGCGACATTTTTTTACCTAAAATTACCACCGTGATCTGTTTTTTTCGTCATTTTTGCTGCAATTTTGACAATGACTTTGACACTAACCTAAATTAACCAAACAACTGT

Protein RF -2: -3041->-456 (861AA)

Comparison with *Tribolium* hypothetical protein TcasGA2_TC002596 [Tribolium castaneum] (1249AA)

Query 54 LKSVYNMVKRSPPVVAKLEQYFGTCSNPYEQTLRLLNNCQDFFNAKNKSLPIFIAEEFKQ 113

LK + + VK+SPPVV KL QYF C NPYE T+RL+ NCQ+F +AK+KSLP FI EEFK

Sbjct 405 LKCLLSTVKKSPPVVNKLHQYFHLCENPYEHTIRLMYNCQEFNSAKSKSLPFFIIEEFKI 464

Query 114 WSGRHRHMIVHLLVPQIKLDAFKLITRQNSQALTKLVFDAYEMAREPDVFLDNVRCLIEN 173

W HR+ +VHLL P++K+D FK+I++QN+Q LTKLV D YEMA++ ++FLD ++C+IE

Sbjct 465 WLSVHRNKVVHLLTPKLKIDVFKIISKQNAQNLTKLVVDVYEMAQDGEIFLDIIKCMIER 524

Query 174 KQYKEACQYATMLNLHDKFTVDDFLVPLVLQDKLCSVDEFLASSSRHQVDLVQFLDAALS 233

K+YKEACQ A + NL DKF+V+DFL+PL+LQDKL +D+FL S RHQV+LV LD+ L

Sbjct 525 KRYKEACQSAVLFNLQDKFSVEDFLLPLILQDKLYGIDDFLTVSPRHQVELVTLLDSTLG 584

Query 234 RISVGETVLPYIESRNIPGVKYDKVHSKPWKKTITRFAKMFKMSPDLTPNLNRKRNEGAL 293

R SV + + Y+ + ++P +K+DK+H+KP KK ITR KMFK+ ++TPNLN++RNEGAL

Sbjct 585 RTSVRDALASYVFNLDVPDIKWDKLHAKPLKKLITRLVKMFKLPTNITPNLNKRRNEGAL 644

Query 294 KFLIHKRFTDNTFSDESWKEMVQEAVGDEESLQKEVVQMVAQYGEFAEALKWARFYNIDK 353

+FL+HKRF +N+F DESWKEMVQEA+G++E LQ+E+V V+ YG AEAL WA FYN+DK

Sbjct 645 QFLLHKRFVENSFGDESWKEMVQEAIGEDEELQRELVAQVSTYGAVAEALWWAHFYNVDK 704

Query 354 NDWPYSVRALEEN------GSSDTAPTDD-WDDEPVNR---VDYHKLALPLERIVLVDTA 403

WPY+VR LEEN + P ++ W + V V+YHK LP I L+D+

Sbjct 705 QHWPYNVRMLEENPDEERLHQRNILPEEESWGYDEVQNTEPVEYHKFPLPFSSIHLIDSE 764

Query 404 ARFKNMLEFGFRGADMVGIDCEWKPSFLSQANELALMQISTRETVFVLDIVNLSNKEPRL 463

F+ L+ G + ++VGIDCEWKP+F SQ NELALMQI++R+ VF+LDI+++ K P L

Sbjct 765 ESFERFLDGGLQDVEVVGIDCEWKPNFGSQKNELALMQIASRKNVFILDIISIGTKVPHL 824

Query 464 WQELGRKLFNNCDILKLGFSLTSDFHMIRQALPELNFSTNRVGFLDLCTLWKHLDKYPKV 523

WQELG+ LFNNCDILKLGF TSD MI+ +LPELNF+ +VGFLDL +LWK L+KYPKV

Sbjct 825 WQELGKFLFNNCDILKLGFGFTSDILMIKHSLPELNFTPKQVGFLDLLSLWKLLEKYPKV 884

Query 524 TLPHEVQTGGPSLSTLVQACLGRPLDKSEQFSNWENRPLRQSQIGYAALDAYCLVEVYDV 583

LP+EVQ GPSL TLV CLGRPLDKS+QFSNWE RPLR SQ+ YAALDAYCL+EVYDV

Sbjct 885 VLPYEVQGSGPSLGTLVNQCLGRPLDKSDQFSNWEKRPLRNSQLVYAALDAYCLIEVYDV 944

Query 584 LKQCCETAGCPFYEICDGLANNEPSVKKKPNKKRH------QQEEDVPQPPSPHPESIRV 637

+K CCE A PF E C L NE + KKK K Q +E++ QPPSPH +

Sbjct 945 IKGCCEKAEFPFDETCYNLMTNEKAPKKKAKKPVQKKPKPLQADEEIAQPPSPHSSQVPA 1004

Query 638 DQLKVVCDTMLQGLGKNLRRCGIDTAILENHHDHKECVKYHLNERRYILTRKGPFKMLNG 697

+KVVCDTMLQGLGKNLRRCGIDTAILEN+ DH ECV+Y +E+RYILT+ F L G

Sbjct 1005 ASIKVVCDTMLQGLGKNLRRCGIDTAILENYMDHMECVRYAQDEQRYILTKGNVFNKLYG 1064

Query 698 YVPSGHCLKIISDDVDEQLQQVLEYYKVTVTKDDVFSRCQACNGNSFVRVPQSTMLALNG 757

YVP GHCL++ SD+VDEQL++ ++YYKV VT +DVFS CQ+CNG SF++V +STMLAL

Sbjct 1065 YVPLGHCLRVNSDNVDEQLKEFVDYYKVNVTVNDVFSVCQSCNGRSFIKVSRSTMLALTQ 1124

Query 758 GPSMVNYAPGGYYE--DESTGFTSDEDLDDEMPGHSSGSRKWELT--------------- 800

+ + Y P Y DE+TGF+SD+D D E +RKW+L

Sbjct 1125 SQNSLQYVPPDYDNDIDEATGFSSDDDFDFEPGPPVQTTRKWDLCMHFTYNYYFSLTFII 1184

Query 801 ----DRIDVDSRLTRLGVTIKADQIPRPVIYNYQVFYICEECGKVYYDGSHFDRVLNGRL 856

+++DV TRLG I+ IP V+ ++FY+CE CGK+++DGSH +RVL GRL

Sbjct 1185 LDSDEKLDVGLCQTRLGAKIQVATIPDGVLEKTELFYVCEHCGKIFWDGSHLERVLTGRL 1244

Query 857 QGIVQ 861

QGIVQ

Sbjct 1245 QGIVQ 1249

Graphical representation


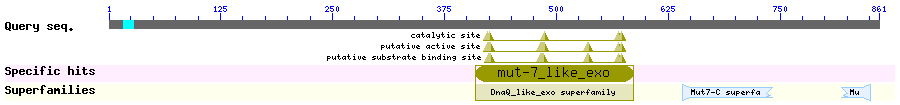


**Sdn1-like (small RNA-degrading nuclease 1)**

>Cp.comp36451_c0_seq1 len=3501

AGTGCAAATAGTATTATCTCTCTTGTTCCATGCATTCGTCAGCCACATGTTCGATAACGAGGTCAAAATCCATTCTATTTTTAGAACGGATAATAATAACCGAATCGGTTTCCTGTTAGACGTTTTGTGCGTTACGTTGTGCGGGTTTTTATGTGTCGCCATTTGGCCAAACGACCCGAATTGTAGCGGCCGACGATGCGACGCGACGGTTTTATACAGTGCCAGTGTCAGCGTTGACAGTTTACTTTAAGGTTAAACAAAGGTTTTCAATTATTGTTCTGGCTCGAAATTTAAGCGTTGAAATGCTAAAGTAGGACGAAACTATGCTGCCTACGAAGGGGTATTTCCAAGATATCGAGTGCCCTTTCAACGATACGTCGTGCGGCCGGCCCTATTGTCATTTCAGGCACAGGAAAAGGGCAATCGAGAACTTGGAAGAAACCGTTCCGGAATCCTCCAAATCGCAAGTACCGACGTACAAACCGACCCCGAAAAGTGAATTGGAGAATATTCAGAACAAGACGCACATACCTATAAGTTACGTGCCCGATTTGGCGTTTAGAAACGATCGGCCTTTAAGACCCAAGTTCGATAAACCTACGTATAAGCCTACACCTTTGAGCTTACTGTCGTCTGCTAATAATAAAAGCTCATTATCGGGCCACGACGAGAAACAACAAGAAACTATTAAAGACATTCAACAAAACATCGCCAACAATGAATACGACCCGTTAAAGTCGGAAATCAACTTTGAAGATTTATCGAACGAGTTCGATTTAATCGACGAGATTATTAATGACGACCAATCGGATCAGGAGGTGTTAATATCGGAAAATTTGAGCAAAATAAACAGCGATATTAAAAAGGAGCAAAATCGTTTGAATTTGTTGTTGAAAAACGACTTAAACGCCGATGATGAAACCGGCAAAGCTAACGACAATGTCAAAAAACACGAAATTGTTTTAAAACAACATATTAAAAAGGAAAAAGATGATTTGTTCGGAGAAAAACCGATCAAAAAAGAGAAATCTGACGATAAAAAAATCAAAGAGGAAGACAAAAGCCGGAGTAAAAGTAAAACTGGCTCAGATGATAAAGTACAAATTAAAAAAGAGAAAAATCGTCATAGAAAAGACAGTCGGAGCGATCGAAAGGAGGAACATAAGAGCCGAAAGAAAGATGACAATAGCAACAGGGAAAAACACAACAAAAGTCGAGAGAGGAGTAGAAGCTCAGATCGACACAAGCACAAAAAGCGTAGCCGGAGCAGGAAACGGAGCAAAACGAAAGAACGAGACAGAACGAGTCACAAAAGTAGCAAAGAAAAGAGACGGAAATCGTCGTGCAGTTCGGGCAGTGATAGTGAACGTTTAAGGCATAAAAAGAGCAATAATAGTCGCGGTGAAAAAGAATCAGAGTCGAAAAAGCAGAAAGAAAAGACTGGTAAAATCAAAGACAAAGAAAAAAATAAAAAAACGACGTTCGGTGATAGTGATAATGATAATGACGTAACCCCGATGGAAACTGACGAATTAGGACTGTCCGACGTCGATTTAGACTTGGATGATGAAGATGAAACAATGAGAGAATGTTACAGGATATTTAACGAGTATAAACCTAAAACGGAAAGGTTACCGTCTCCTAAACTCGTAGATTTGGCTAAAAAGGATTTATCTGAAGAGCAATACAATCCCGTGTCGATGAAAAAACGAGTGGCGCACGGCGGCGCCGAAACCTCCAAACCCGTCTCGACGCCGGTCCCGGTAAAACCGAAATCGGTCGCCACCCCCGGTCAAATCCTAGCAAATCGTTACAAGGCTGCCAAATTGGCGCAGGCCAACAACGAACAAGAAAACATAATGAACGAAGTGAGACAAATTACCGCGATCCGTTCGGCCCCGTCGTTACTGGAAGCGGCCCGGTTGCACAAGCTGCGCAGACTCGAACGCCAAAAACAATTGGAAAAAGCATCGGCGCAGAAACCCTCCACCAATATCGTCGACGATATATTAAACGGTGTTCAAAAACCGACCGTTTCGAAAATTAAACTGCCGGTTAAAAAAATAGCGGCCGTACCGAACGTGGCGCTTATAGAAAAGGCCAAAGAACGTATTTCGTTGATAAAACAGCGCAGAATGGACGTTCCGAAGACGGTCGCGCAGACTCAGAAAACGGGCCGGGTCGCGCACGTGCCGGAAGTGTCGTTACCGGACGTTCCGGACGTTTTGAACGCGGACAAGTCGAAACTTCCGGTCAACGTTCGGACGCGATTTTTAACGATGATCGTCGAAGAGTGTTGCAAGTTGTACGCGTCGAAACAGGACGCTTACGATAGAGCTTTGAACGAGGAATATTCGTGTTACGAAAAGTGCAAAGTGATCGCTACTTATAGGAACTCGGCTATGCTCGCGGTCAACAGGTTACGGAAAGAGATTCAGGATAGGGAAAATCGAAATTTGGGACCGTTGCTGGCCGGAGAAACCCCTTGTTCGGATAAAGAATCCGATTTTCGGGGGAAAAGATTTTACGAGCACGTGAAGAAGTGGATACTTTCGGAGGAGGAGCTCGATTTGCACGGTTATCCTAGGGAGAGTAGCGAAAAGGGCCTGGCGATTATTAATAATCAACGGGATGTCGATTATTCGATCGTCGACGAGAATCAGAGGAAATGTAGCCGATGTTCCAAGATCTACCAAGTGGACGACGACGGTTGGCCCTTGTTCGAAGAAGAGTGCATGTACCACCCCCTAAAAAAGCGCACCATCAGGGGCGAACAGGTGTTCCTGTGTTGTAAAAGTACCGACGACACCGGCTGCGTAA

CCTCCGACACGCACGTTTTCGACGGTCTCAAAAGTCACCAGCTCGAAGGCTACCAAACCACCCTACCGCCCGAAAGAGACGACGACCCCAGAAGTTACGCGGTCTACGCATTGGATTGCGAAATGTGTTACACGACCAAAGGCCTGGAACTGACCAGAGTGACGATAGTAGATCCCGATTGCAAAACCGTGTACGAAAGTCTAGTGAAGCCGTTAAATCCGATCGTCGATTACAACACCAGGTTTTCGGGCATCACCAAAGAACAAATGGACCGGACTAGTACGAGCATATTGCAAGTTCAGGCTAATATATTGCACCTTTGCAATTCGGCTACGATTTTAGCCGGTCACAGTCTGGAATCGGACATGAAAGCGTTAAAAATCGTGCACGGTTCCGTCATCGACACGTCCGTCTTGTTCCCTCATAAAATGGGACTGCCCCATAAACGGGCGTTACGGGCCTTGGCCAGTGAATATTTAAAAAAGATCATTCAGAACGACGTTAGCGGACACGACAGCGCCGAGGACGCTATCGCTTGTATGGAACTGATCAAGTGGAAATTGAAAGAGGAACGCAAAGTGCGAACGAAATAATTTGTAAATACGTTAGTATTATTTAAGTATCTCAATTGTTCGATTGTAAATTGTATTTATCCGTTAAATAAATTATTTTCAATTTTTT

Protein RF 3: 324->3413 (1029AA)

Comparison with *Tribolium* hypothetical protein TcasGA2_TC002980 [Tribolium castaneum] (927AA)

Query 1 MLPTKGYFQDIECPFNDTSCGRPYCHFRHRKRAIENLEETVPESSKS-QVPTYKPTPKSE 59

MLPTKGYFQDIECP+ D++C RPYCHFRHRK+ E +EE E+ K +VPTYKPTPKSE

Sbjct 1 MLPTKGYFQDIECPYFDSTCNRPYCHFRHRKKTQETIEEVANETPKEVEVPTYKPTPKSE 60

Query 60 LENIQNKTHIPISYVPDLAFRNDRPLRP----KFDKPTYKPTPLSLLSSANNKSSLSGHD 115

L NI K+HIPISYVPDLAFR+DR +RP F+KPTYKPTPLS+LSSA+ + ++ D

Sbjct 61 LANI--KSHIPISYVPDLAFRSDRTIRPLPKFTFEKPTYKPTPLSILSSASKRENVLEDD 118

Query 116 EKQQ---ETIKDIQQNIANNEYDPLKSEINFEDLSNEFDLIDEIINDDQSDQEVLISENL 172

E++ E I++++QNIAN+EY+P E L D+I +D S + +I + +

Sbjct 119 ERETSDIEAIREVKQNIANDEYNP------------EISLQDDINFEDLSAEFDMIDDLI 166

Query 173 SKINSDIKKEQNRLNLLLKNDLNADDETGKANDNVKKHEIVLKQHIKKEKDDLFGEKPIK 232

+ + KE N +ET + ND K+ K D +K

Sbjct 167 EEPKNKESKENNV----------GKEETHQENDKDKE----------KPASDKTEKKHSS 206

Query 233 KEKSDDKKIKEEDKSRSKSKTGSDDKVQIKKEKNRHRKDSRSDRKEEHKSRKKDDNSNRE 292

K + ++ KSK + +K +K+R KD RSD K +HKS ++ +

Sbjct 207 SSSKSHKSKYHKKDTKDKSKDKHVKEKDVKSDKSRE-KDRRSDHKSKHKSSRE--KEKSK 263

Query 293 KHNKSRERSRSSDRHKHKKRSRSRKRSKTKERDRTSHKSSKEKRRKSSCSSGSDSERLRH 352

+K + RSRS R +HK++ + K + + D +KRRKSS SSG DS L +

Sbjct 264 SKHKDKRRSRSRSRERHKEKEKRAKEKQKEAEDN-------KKRRKSSESSGEDSSELLY 316

Query 353 KKSNNSRGEKESESKKQKEKTGKIKDKEKNKKTTFGDSDNDNDVTPMETDELGLSDVDLD 412

SNN DE+ D D

Sbjct 317 --SNN-------------------------------------------FDEIPALDFD-- 329

Query 413 LDDEDETMRECYRIFNEYKPKTERLPSPKLVDLAKKDLSEEQYNPVSMKKRVAHGGAETS 472

DDE++T+ ECY+IF EY+P + P A+ ++ +E+ N + KKR+AH A S

Sbjct 330 -DDEEDTLSECYKIFKEYEPPKVEVKEPP---PAEPEVIKEETN--ASKKRIAHSSANPS 383

Query 473 KPVSTPVPVKPKSVATPGQILANRYKAAKLAQANNEQENIMNEVRQITAIRSAPSLLEAA 532

+ S VKPK A P Q +ANR+K AKLAQANNEQ+N+MNEV+Q T R APSLLEAA

Sbjct 384 EGGSKINYVKPKIQANPAQAMANRFKLAKLAQANNEQKNLMNEVKQ-TVKRPAPSLLEAA 442

Query 533 RLHKLRRLERQKQLEKASAQKPSTNIVDDILNGVQ-KPTVSKIKLPVKKIAAVPNVALIE 591

R +KL+RL + K E A N++D ILN + KP KKIA V NV I+

Sbjct 443 RNYKLQRLAKPKPSENA-------NVIDSILNSAKNKP---------KKIAPVQNVNSIQ 486

Query 592 KAKERI-SLIKQRRMD-VPKTVAQTQKTGRVAHVPEVSLPDVPDVLNADKSKLPVNVRTR 649

+AK RI L KQ+ + + KT AQT K R+AHVP++SL D+PDVLNADKSKLP+NVRTR

Sbjct 487 RAKARIEELAKQKATETLNKTPAQTVKGKRIAHVPDISLSDIPDVLNADKSKLPINVRTR 546

Query 650 FLTMIVEECCKLYASKQDAYDRALNEEYSCYEKCKVIATYRNSAMLAVNRLRKEIQDREN 709

FLTMI +EC KLY K+DAY RALNEE+ CYEKCKV+ATY+NSAMLAVNRLRKE+Q+R+

Sbjct 547 FLTMIADECVKLYLIKEDAYTRALNEEFVCYEKCKVLATYKNSAMLAVNRLRKELQERDR 606

Query 710 RNLGPLLAGETPCSDKESDFRGKRFYEHVKKWILSEEELDLHGYPRESSEKGLAIINNQR 769

LGP+ GE P +D S+++G +FY H+K + L+ EELD+HGYPRES+ G A I N++

Sbjct 607 LGLGPIGEGEAPANDTASNYKGAKFYNHIKGYALTNEELDIHGYPRESATPGRATIKNRK 666

Query 770 DVDYSIVDENQRKCSRCSKIYQVDDDGWPLFEEECMYHPLKKRTIRGEQVFLCCKSTDDT 829

+S + ENQRKCSRC KIY VD+DG+ + EEC+YHPLKKRT+RGEQ +LCCKS DD

Sbjct 667 TTAWSSLRENQRKCSRCGKIYLVDEDGFVQYPEECIYHPLKKRTLRGEQTYLCCKSNDDV 726

Query 830 GCVTSDTHVFDGLKSHQLEGYQTTLPPERDDDPRSYAVYALDCEMCYTTKGLELTRVTIV 889

GC TS+THV + +LEG+QTT+ PE ++DPRS AVYALDCEMCYT KGLELTRVTIV

Sbjct 727 GCATSNTHVSEACGDAELEGFQTTMEPESEEDPRSQAVYALDCEMCYTIKGLELTRVTIV 786

Query 890 DPDCKTVYESLVKPLNPIVDYNTRFSGITKEQMDRTSTSILQVQANILHLCNSATILAGH 949

D +CKTVYE+LVKPLNPI+DYNT FSGITKEQM+RTSTSILQVQANILHLCNS TIL GH

Sbjct 787 DSECKTVYETLVKPLNPIIDYNTTFSGITKEQMERTSTSILQVQANILHLCNSKTILIGH 846

Query 950 SLESDMKALKIVHGSVIDTSVLFPHKMGLPHKRALRALASEYLKKIIQNDVSGHDSAEDA 1009

SLESDMKALKI+HG+VIDTSVLFPHKMGLPHKRAL+ALAS++LKKIIQN VSGHDSAEDA

Sbjct 847 SLESDMKALKIIHGTVIDTSVLFPHKMGLPHKRALKALASDFLKKIIQNSVSGHDSAEDA 906

Query 1010 IACMELIKWKLKEERKVR 1027

I CMEL+KWKL+EE KVR

Sbjct 907 ITCMELVKWKLREELKVR 924

Graphical representation


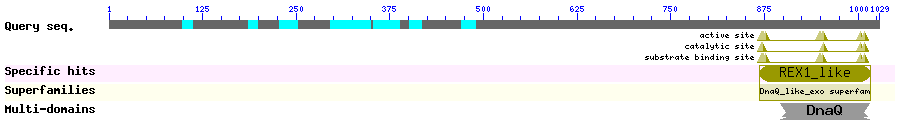


**dsRNAse (Bombyx-Drosophila)**

>Cp.comp35928_c0_seq3 len=1589

ATTAACTAAGATGCAAGGCAGTTATTCGATTTTAGATCTCAGTAGGACCGGGCGTCATATAAGTAGGTATTTATTCCTACGTACGGAAGTTAGCTTGCAAATTTTTACCAATAACTAAGATGCAAGGCAGTTCTTTGATTTTAGATCTCAGTAGGCTTTTGCTTATTTCCCACACCATAACGTTGGTGCTTGCAAATGATGATTGTGAAATTTATCCTTTCGAAGTCGATCCGTCGCCTTTGGTGGTGTTATCTGGAACCAGTGATTTTATATATCCCAAGCCGTTTGAAAAAACCTTAAAATTCAGCTCCGGTCAATCTCTGGATTTCGTATGCCCGGAACGGACTTTGATTTTAGGAGCTACGAAAACGAACGATTCAGTGTTACAAGGTACATGTGTAAAAGATGATACGTTCAAGATAAACGGCGGAGAAGTACTTTGGAATCAAATATCGTGCAGCAATTATCCATATAGGGTTGCCAAACGTACCGGTCTATCGTGCGAAAATGGAGGCACAGACATAGAGATAGGTTTTGAGGTTGGAGATGGTCGTTTTTTGAGATCCCTGAGTGTGTGTTTTAACGAATCAAGCCAGATTGCTTTATACTCTTTTCACAATATGACTACTGCTATTAATCAGAGAGTTCTAGCCACTCCTAGGCCTTCTTGGCTGCAAGGCTCGGGGCTCTATACAATCAGTACCATAACCAATTATTACGTACGGAACAAACAGAGGAGTACTATTAACACTTTGTTAGGATTGGATATCAATTCCACGAAATACATCGAGGACAATTCCAACTACTATTTGGCTCGTGGGCATCTAAGTGCTAGAAGCGATCATTATTACGCGGCTCAACAAAACGCCACATTTTTTTTGATAAACGTTGCTCCTCAGTGGCAAACGTTCAACAGTCTCAATTGGAACCAGGTCGAGATAGACGTAAGAGATTACGCCGAAGAACGCGGAATAAATCTTCAAGTTTGGACCGGCATTTACGGAGTCACTATATTGCCCCACGAGCAAACTGGTCGTCCGGTAGAGTTATATTTATACGTCGATGACAATAATAACAAAGCATTACCTGTACCGGAAATATACTGGAAAGTTGCGTACGACCCTATCACTGAGAAAGGACTCGCCATGATCGGTGTCAACAATCCGTATCTTGCAAATTATACGGAGATCTGTAATGACGTTTCCGACAGAATAACATGGTTGCATTGGAAGAAAGACGATCAGGCGCGGGGTTATTCCTACGCGTGCGCTGTTGACGTATTTCGAAGGGTCGTCACATCACTACCCGATATTCCTGTCAGAGGACTGTTATTATAACATTAACATTGTTTAATTTTATGCAATAATATAATACACTAAATGCGACAAAGATGGCTAAGCGTGTGTTTTTGATGGTATAAGAAACCGGCAATAATTAGTGGTATCTTTTCTTTTATATAGATTACAACTCACTGTGAGTGACTGCAGTAATAATTTATTTAGTTTGATATGTAACCTCGGGATAGTATTTATCTATAAACTTCTCGGTTATATGTATAACCTGTAAGTGTGATTATACTTTTTCAGGCA

Protein RF 3: 120->1337 (405AA)

Comparison with *Tribolium* PREDICTED: similar to CG6839 CG6839-PA (400AA)

Query 22 LVLANDDCEIYPFEVDPSPLVVLSGTSDFIYPKPFEKTLKFSSGQSLDFVCPERTLILGA 81

+L DC I +DP P+VV GT F+Y P ++ SG+++ CP + +G+

Sbjct 18 FILRAPDCNIQISNLDPEPIVV-DGTYTFLYAAPDASSVLVKSGETIIISCPGGEITVGS 76

Query 82 TKTNDSVLQGTCVKDDTFKINGGEVLWNQISCSNYPYRVAKRTGLSCENGGTDIEIGFEV 141

T N S + TCV + F + + +NQI CS P+ A+ TG CE G +IE+GF +

Sbjct 77 TSFN-STVSATCVSNSDFSVGSATINFNQIVCSWNPFHTARYTGKLCEKQGKEIEVGFVI 135

Query 142 GDGRFLRSLSVCFNESSQIALYSFHNMTTAINQRVLATPRPSWLQGSGLYTISTITNYYV 201

+ F R +++CF+ ++ LYS + +T +I RP +++ + + YV

Sbjct 136 NE-NFAREITICFDNANLNTLYSSYEITKSIGHHESGVSRPFFIEDDFYNLDVKVNSLYV 194

Query 202 RNKQRSTINTLLGLDINSTKYIEDNSNYYLARGHLSARSDHYYAAQQNATFFLINVAPQW 261

R QR+TIN+LLGL STKYI+D +++YLARGH +A++D YA QQ ATF +NVAPQW

Sbjct 195 RGGQRTTINSLLGLPAGSTKYIQDGNDFYLARGHFAAKADFVYAPQQTATFHYVNVAPQW 254

Query 262 QTFNSLNWNQVEIDVRDYAEERGINLQVWTGIYGVTILPHEQTGRPVELYLYVDDNNNKA 321

Q+FN NWNQVE DVRDYAE+ GI+L+++TG YGVT LPHE+TG LYLY+ N +

Sbjct 255 QSFNGYNWNQVESDVRDYAEKNGIDLKMYTGTYGVTTLPHEETGEETPLYLYIGSNGIQG 314

Query 322 LPVPEIYWKVAYDPITEKGLAMIGVNNPYLA--NYTEICNDVSDRITWLHWKKDDQARGY 379

+ VPE+YWKVAY+P T+ G+A++G+NNPY N + IC DVS +I WLHW D GY

Sbjct 315 IAVPELYWKVAYNPETQLGVALLGINNPYQKDINKSIICEDVSAKINWLHWNASDTKAGY 374

Query 380 SYACAVDVFRRVVTSLPDIPVRGLLL 405

SYAC VD FR+ VT LPD V+GLLL

Sbjct 375 SYACEVDAFRKRVTYLPDFVVKGLLL 400

Graphical representation


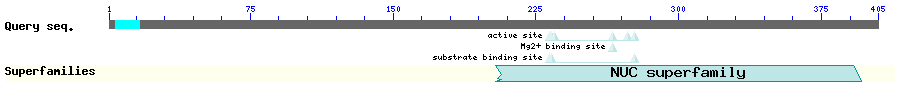


**Exosome**

>Cp.comp37176_c0_seq1 len=3045

AATGGAATTATGAATATATACAGATTTGTAGTGACGCCATTACAACTGTCGATCGAACTTTGGTGCAAGTAATGTTAACCTCAGTATTCTATGGTGTAATGAAGAAGAAGAAGAGGTATTCTATGGTATACTCACACATTTTTGTCAAAGGTTAAGTAAAAAACAATAATTTTTAAAATGATTTCAAAAAATCAACCACCGTCTTGTGAAAACGAAAAAAATCAACATGATAGTGCTGGAAATAAATCTATAGAAGAGTTCCTTAAGGACGGATACCAAGTCGTGAAGGAAACGTTAAAATTATCGAACTCTCTACCAAGTGGCAAAAAAATTCAAGATTTATACGGTGGATTCCTATAAAACAACCTTACAAGAATGTAGCGATAATATTCTTAGTTCCATGAACTCGATATTAAGAAAAAATGAAGTAAAAGGCAACATGCGCAACAATGTCTTGGAGAAGAAAACTGAATTGCTTATTGAAGCCTGCGACGTTATACTTGAAAGAGTCACAAATAATATTGATGAAGTAAATGGGATAAGGAAAAATGATGTCGAACCCATGGTCTTTCAAACCGTTAGTGTACAATTGCCAGTCAATGGATCTTGGAATCGTGTCAGTAAACCCGTGATGTCTGCTACTTCGGCAATTACTTCTTCACAAACTACAGCATCTGGCAGTTCCACTCAGACTATCCGCTTGATTACAGCTGAGAATATTGTTCGACCTCAGAAATTCTTTAAAGATAAAATAGATAACAGCAGACAGAGGCCTTGGGAGCCTCGAATAAAAGAGAAACCAAATTCAATTAAACCGCTTGCCATATTTTTGGAAGAAACAGAAAATGGACAGGAATACTCGCATCCTTATGAATGTGAACTTGATAAATTTTCACCCCCAGAGGATCAGTTAAGGAAAAGCGATGTAGTGAAACCTAAACTGTTAAAAGATACACCTCTGGTAGAAATAACTCAAGAGGAACAATTGAATGATTTAGTGGAAGATCTTCGAAGTGCTAAGGAAATCGGGATAGATCTTGAACATCATTCGTACAGATCTTTCATGGGTATTACTTGCCTTCTTCAGATTTCTACATTAGAAAAAGATTATTTGATCGATACTTTGGTATTGAGAGATAAATTATATATTTTGAATGAAATATTTACTGATCCGAAAATAATTAAAATTTTTCATGGTGCCAAACATGACATACAGTGGTTACAGAGAGACTTATCGCTCTATGTAGTCAATATGTTTGATACACATTTTGCAGCTAAACAGCTTGAATATCCAGGTCTGTCACTGGCTTATTTAATGCAAAAGTTTTGCAATTTCGCACCAAATAAGCAGTTTCAATTGGCTGATTGGAGAATTCGTCCACTACCGCATGAACTAAAAACTTACGCGAGAGAAGATACCCATTACCTGATTTACATATATCAAAATATGAAGAACGAGCTGATCGATAAGTATAACGGTCAACCTAACCTATTAAAATCAGTGATACAAATGAGTACCGATTTATGCAAAACTCGTTATGTAAAACCAGTTTTTACTAGCGATAGTTATTTGAACCTGTACCGACGTTGCAGCAGAATGTTCCACAATAGACAGTTGTTTGCACTCCAGGAACTATATAGATGGAGAGATGAGATATCTCGTGAAGAAGATGAAAGTACTGGTTACGTTTTACCAAATAATATGTTATTGGAAATATCAGAACGACTGCCCAGAGAAATGCAAGGAATTCTTGCCTGTTGCAATCCTACTCCACCGCTGGTCAAGGCCAATCTACTTGATTTGCATAAAATAATGTTAAAAGCTCTGGAACAACCATTTGAGTCACCAATTTTGAAAGAAGATACTCGATCGAGGGGCATTACTACTAAAATATCGAAGATTGATGTCAACAACCCTTTGCATTGTCCTCACGACCTAACCAAAAGGGAGGAAGTCTGCGATAATTTGCCTACAATTTTAAGCGGGGATTCAATTAATCTTGACAGTATTTTCACCCATTATATTGAGGAAGACTCTTCCGTTTCTTCTATATTTGACTTTCCAGTAAACGCAGCCAATGAGACAAGACTACCAAACAAATTAGAAGGAGTACTAAAGTTTTTAAGCCCTTATGCAAGATATAAATTAATTAAACCGTTCATGCAGGCTAGAGAACAGAAAATTGTGGAAGAGAGAGAAAAGAAAATTGCGGAGGAAAATGCCAAAAATGAGAAAGTAGTTGCTAATGATGATCAAACAGAAACGAAGACCGATGATAATAGAACAGATGAAGACAGAATTGAAGGTATAAGAAAACATTTTCTGAAACTCTCAAACCAGGCTGTTGCTACAACCTCATCAAAAGAAAATGACAAAAAGAGGAAACGAGATAACTCTGTTGATTTTAATTCAGACTTTACAGAGATACCAGGATCTGAGGAAGTCCTACCAGATTCAGGTACACTACAAGAAAGACTTTCAGAAAATGCAGATTCGAATGCCAGGGAATGGAGAAAGTTTGATACAAATGGTCATAAGGGAAAGGGTAATAAAAAGAAGAATAAAATGAAGCGCAAACAAATGTCAGATAGGGAGCAAACGCAAGACGGTGAAACTAGTGTAAATAGTTGTAGTTCATTAACAGCTGCACAAAAAAACAATAATCAGAGAGACAAATTAAGAAAGGCACAAAAAAACAAACAGTTCTGGCAAAATCGCAAAAAGGGACAAAAAAAGAATATTAGTCAGCCAAGTAATGACAACAGTTCCAACCAAAATCAACAACAATTTAATGCCTATGATTATTCAATGGTTGATTT

TAGACAATTTCAAGGCGGTGCAAGGGGAGTTGACAAGCCTAAAGCGTTTAAAAGCAAATTTAAATCGAAAGGAAAGAAAAGAAATATAGGAAATAGGAATCCAAGGAAGACAATTTAATATTATCATATAAAACTTTACACCTTTTTTTTAATATTTGACTATTTATGTACAATTTTTTCAAAATACTTGTGTAAAAATATTGTTAATAAAAATCTTTATCGATG

Protein RF 2: 350->2938 (862AA); RF1 178->360 (60AA)

Comparison with *Tribolium* PREDICTED: similar to Rrp6 CG7292-PB [Tribolium castaneum] (947AA)

Range 1: E=0.018; bits=38,9

Query 238 GNKSIEEFLKDGYQVVKETLKLSNSLPSGK 327

G S+E+F KDG++V+ E +K SN+LPSG+

Sbjct 21 GYSSMEQFTKDGFKVLMEAIKHSNALPSGR 50

Range 2: E=0.0; bits=711

Query 350 DSYKTTLQECSDNILSSMNSILRKNEVKGNMRNNVLEKKTELLIEACDVILERVTNNIDE 529

DS+K ++ +++L MN ++R N++ N+RN VL++K EL+IEA D+ILE+V NNIDE

Sbjct 59 DSFKEIMKVEGNHVLRLMNQVMRCNDLDSNLRNRVLDEKIELVIEANDIILEKVANNIDE 118

Query 530 VNGIRKNDVEPMVFQTVSVQLPVNGSWNRVSKPVMsatsaitssqttasgsstqtiRLIT 709

+NGIRK V P+V QTVS QLPVNGSWNR + ++ +S + S +LIT

Sbjct 119 MNGIRKTVVAPVVLQTVSAQLPVNGSWNRQTAATVTVSSVVPESSGQNCI------KLIT 172

Query 710 AENIVRPQKFFKDKIDNSRQRPWEPRIKEKPNSIKPLAIFLEETENGQEYSHPYECELDK 889

A+NI+RPQKFFKD+IDN + PW PRI EKPNS+KPLAIFLEE E+ QEYSHPYE ELD+

Sbjct 173 AKNIIRPQKFFKDQIDNRNKTPWSPRITEKPNSLKPLAIFLEEYEDRQEYSHPYEFELDR 232

Query 890 FSPPEDQLRKSDVVKPKLLKDTPLVEITQEEQLNDLVEDLRSAKEIGIDLEHHSYRSFMG 1069

F P QL V PK L DTPL+EI + EQL++LVE LR KE +D+EHHSYRSFMG

Sbjct 233 FQPTPSQLIDEKSVPPKSLSDTPLIEIDKAEQLDELVETLRHCKEFSVDVEHHSYRSFMG 292

Query 1070 ITCLLQISTLEKDYLIDTLVLRDKLYILNEIFTDPKIIKIFHGAKHDIQWLQRDLSLYVV 1249

ITCL+QIST +KDYLID L LRDKL ILNE+FT I+KIFHGA DI+WLQRDLSLYVV

Sbjct 293 ITCLIQISTEDKDYLIDALALRDKLSILNEVFTKNTIVKIFHGADKDIEWLQRDLSLYVV 352

Query 1250 NMFDTHFAAKQLEYPGLSLAYLMQKFCNFAPNKQFQLADWRIRPLPHELKTYAREDTHYL 1429

NMFDTH AAK L+YP LSLA+LM+KFCN PNKQFQLADWRIRPLP ELK+YAREDTHYL

Sbjct 353 NMFDTHQAAKALQYPALSLAFLMKKFCNVTPNKQFQLADWRIRPLPDELKSYAREDTHYL 412

Query 1430 IYIYQNMKNELIDKYNGQPNLLKSVIQMSTDLCKTRYVKPVFTSDSYLNLYRRCSRMFHN 1609

IYIY+ MK EL+ K N LL+SVI+ ST++CK RY KP+ DS+L LYR+C +MF N

Sbjct 413 IYIYKMMKRELLHKTNKCDKLLRSVIERSTEVCKKRYFKPILHEDSHLELYRKCKKMFDN 472

Query 1610 RQLFALQE---LYRWRDEISREEDESTGYVLPNNMLLEISERLPREMQGILACCNPTPPL 1780

RQ++AL+E S YVLPN+MLL+ISE LPREMQGILACCNP PPL

Sbjct 473 RQMYALKEXXXXXXXXXXXXXXXXXSCSYVLPNHMLLQISELLPREMQGILACCNPIPPL 532

Query 1781 VKANLLDLHKIMLKALEQPFESPILKEDTRSRGITTKISKIDVNNPLHCPHDLTKREEVC 1960

V+++LL+LH+I+LKA EQP E ILKE T RG+ ++SK+++++ LHCPHDLTK E

Sbjct 533 VRSHLLELHQIILKAREQPLEKAILKE-TSGRGVLKEMSKVNMDSVLHCPHDLTKTNEFR 591

Query 1961 DNLPTILSGDSINLDSIFTHYIEEDSSVSSIFDFPVNAANETRLPNKLEG---------- 2110

D+LPT+L + Y E+ V+ + D + + + N +G

Sbjct 592 DDLPTLLQNE---------QYKAENKRVAQV-DLAIEKPSSYSIFNSDQGFKPGEAFKKL 641

Query 2111 -VLKFLSPYARYKLIKPFMQAREQkiveerekkiaeenaknekvVANDDQTET-KTDDNR 2284

+LSPY RYKL+KPF+ VA D+ + K D +

Sbjct 642 HTSAYLSPYERYKLVKPFV-------------------------VAEDEAAKAQKEKDEK 676

Query 2285 TDEDRIEGIRKHFLKLS 2335

TD++RI IR HF++LS

Sbjct 677 TDDERITSIRDHFVQLS 693

Graphical representation


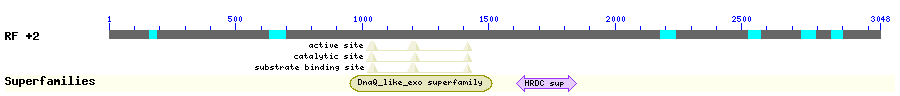


**Poly(A) polymerase (Pla1 homolog Schizosaccharomyces)**

>Cp.comp37990_c0_seq4 len=2705

TTTTTTTTGTAGGGGAAAATATTTTTTATTTATTGTACTTAATCACTTACAGGGAAATTTTTACAACGATGAGAAATTGCTTTAAATAGACTAACTTAAAATACCTATATGAAATAATAAAATAAACTTCTACTTGGTACTCTGGTCAAAATGAATTTTAAACATTATTTTCCCCAATTTTTAAATAAATACAATAATACAATGAATCGGAAAGGCTACATAATAAATTGACAAAAAAATGGTATCGAAATTGATACGAAAAGAAAGTTAATATCAAAAATAAAAAGAGATGAGCTCTATATAAGCATTGCTTAAAATTCCTTCTGAATCTCGATACTAAATCATTGTTTAAATAAAATGAACTATGTACAACTATAGTGTAAAATATTACCGCTTCAGAAATGTTGTTTTTATTTATTAAAATTATGATTTATACATCTAAAAAAAATAACGTAATAAATAAACACACTCATACTCGCACAAAACTACACCAGACGTTACATTTATTAAAGAAATTGCACTTACGCGATAATATTTTACATTTTCCTATAAATCAAAAATACCTGCGTAAATGTCAAGGTGCCGCTATATCCTTCGTCCTCTCCAATGGTGACACTTAAATCCTAAACGACGATCGGCAGGATTCACGAACACACGGTTTCTTGCTTGTTTGCCGTCGGTACCGACGGTAGCTTAACCGGACTCGCCGTCGACGTCGTAGACGCGGCAGATGTATCGGCAAAACTACTCGCGGAATCGTCATTGTTCAATGAATTGTTTGAGCTATCGTCGAAATTAGTTCCGTTCATTTCGTTCGACAACCGGGTCTTTTTAACGGTAGTTTCGCTATCGCTCGATTCCGATTGTCTTTTCCTGCCCTCTTTTTGTTCCTTGCATTCGGTGAACGTAGCCAAATATTTCTTTTCGCGTTTGAGGACGCCCGACGGTAAATAGTTGGGTAACATTTTCCGTTTAACGTGCCTGGCTTCGAGTTTCATAGTATCTTTGAACATTTGTATGTGCATGGCGTGTTTGTTAACCGTTTCGGTAAATTGTTGAATGTCTTGAGTGAGATCAACGTTTAAGCCCTCCATTTTTCGAAATTCCAAACCAATAAACCACATTGAATATTGAAATTTCGGTTTGCGTAACGGTTCCGGTAACATGAAATTTTCTGGGTTTATATGGGCTAACGTTATCGCTCCGTTCCTTTCTAACGTCGAAATCAACAATCGTATCTTGCTCTCCACCAGCCCGCTCCACTCTAAGAGATCTTTTGACGTTTCCGCGCTAATGATTAACACTATAAAATGGCGGTACTTCATGAAAAACGGAGGCTGTACGAACAATCGTTCCCACGTGGATTTGTTTAACATAATTTGATCTGTAATTTTTAGACCCCTTTCGAATTCTTCCATGATGATGTTTCTGGTCGAGTTGGACACGTTAAACGTAGAATTTTGTTGGGGGTACGCTGGCGTAATGATCGGCATCAGGTGGTAACGATCTTGAATGTTTACCCTCGGATCCCAAACAGTAAACCCCAAACTGGCATTGGTAGGTTGCTTAAGAAGTACCGGCTGAGGCCACTGCCACTTTGAAAATATCAAAAAAAACTTATGAACTAAGGTAGCGGGGGCCGCATTCGGGTACAATTGACATGTGCGAGCTACGAGCATTGCCCACGAGACGCCACCTAAATATCCTAACACGTTGCTATAAATACCGTGTCTTTTGGCCCAAAGCTTAATAGCTCTAAGCGCTAATCGAAATGTTTCGATGTTGGGTACGAGCCTCAAAATTTCATCAGTTACTCTGCAGCCGTTAAGGCTTCTCACACACTTTTGGTCGAGATTTTTTAACAAGTTGTCATCGCGAAGATCCATTGAATCGGGAATTTCTTTTTGAAGTAATCGGGCAAACAACAAATCGATTTCAATACCGTCAAAACTCATTTTAATTACCGGCACAAAAGCTTCCTCGACTGCCCTCAACTCTGTCACCTCCGGTTGTTTCTTTAATAATTCGTAAAATGACGTGAAAAAATCACTTCTGCATATGTGCTTTGGTGCAACACATAAAGCATCTATATCGGCGCCTTTGTTATGAACACCAAGCCTATAACTACCGAACGTATACACTTTTCCTCCAACATTTTCCGCAACACTTTCAGGCATGTTACACTTAATCGAGAGTTCTTTTATCCATTGCTTGACTAAGGAATACAATTTGCCTAAAATGAGCATGCGATGATTAAGTTCCTGTTCTGTTTCAAAAACATCAAATGGTATCAAAGTCTGCTCTAACTCCTTGGTTTTAACCAGGTCAATTGGCTTAGGTGGTGCGGCAGAAATAGCGGAAGTCATCCCCAGGGTCACCAAGTTGTCTTCCGATTGATTTTGGTTTTCTTTATTGTTTAAAGTAGAGTTTGACTGTGAAGACCACATTTTTAATGATTTAACACAAAACAAAATACTAGAAATCCAGAGCCCGCCGTTTTAAATGCGTACAAAGCGGGTATCGTCAGTCCTACGACTTCAATTGATTTGTCCTTTTAATATAAATTGCCCAAAAATTATTATTAACTAAAAACATTTCAAAAATCACCAAAAAAAACTATTTATTTTGACAATTTTTGACTGATTATTATTAGGAACAAAAACGTCCAAATCAGCCATTTGTCAGCACTCGGAAGCGATAACGGC

Protein RF -1: -2447->-645 (600AA)

Comparison with *Tribolium* hypothetical protein TcasGA2_TC003818(565AA)

Query 1 MWSSQ--SNSTLNNKENQNQSEDNLV-TLGMTSAISAAPPKPIDLVKTKELEQTLIPFDV 57

MWSSQ +N T NNKEN Q D + TLGMTSAIS APPKP DL+KT+ELE+ L PF V

Sbjct 1 MWSSQPVNNGTQNNKENVTQKNDTKIPTLGMTSAISTAPPKPSDLLKTQELEEALKPFGV 60

Query 58 FETEQELNHRMLILGKLYSLVKQWIKELSIKCNMPESVAENVGGKVYTFGSYRLGVHNKG 117

FE+EQELNHRM+ILGKLYSLVKQWIK++SI NMPESVAENVGGK+YTFGSYRLGVHN+G

Sbjct 61 FESEQELNHRMVILGKLYSLVKQWIKDVSISKNMPESVAENVGGKIYTFGSYRLGVHNRG 120

Query 118 ADIDALCVAPKHICRSDFFTSFYELLKKQPEVTELRAVEEAFVPVIKMSFDGIEIDLLFA 177

ADIDALCVAP+HI R+DFF SFYELLKKQPEVT+LRAVEEAFVPVIKM+FDGIEID+LFA

Sbjct 121 ADIDALCVAPRHISRNDFFGSFYELLKKQPEVTDLRAVEEAFVPVIKMNFDGIEIDMLFA 180

Query 178 RLLQKEIPDSMDLRDDNLLKNLDQKCVRSLNGCRVTDEILRLVPNIETFRLALRAIKLWA 237

RLL KEIPDSMDLRDD LLKNLDQKCVRSLNGCRVTDEILRLVPN++ FRLALRAIKLWA

Sbjct 181 RLLLKEIPDSMDLRDDLLLKNLDQKCVRSLNGCRVTDEILRLVPNVDNFRLALRAIKLWA 240

Query 238 KRHGIYSNVLGYLGGVSWAMLVARTCQLYPNAAPATLVHKFFLIFSKWQWPQPVLLKQPT 297

KRHGIYSN LGYLGGVSWAMLVARTCQLYPNAAPATLVHKFFL+FS+W+WPQPVLLKQP+

Sbjct 241 KRHGIYSNALGYLGGVSWAMLVARTCQLYPNAAPATLVHKFFLVFSQWKWPQPVLLKQPS 300

Query 298 NASLGFTVWDPRVNIQDRYHLMPIITPAYPQQNSTFNVSNSTRNIIMEEFERGLKITDQI 357

N +LGF VWDPRVNIQDRYHLMPIITPAYPQQNSTFNVS STR IIMEEF+ GL++TD I

Sbjct 301 NVNLGFAVWDPRVNIQDRYHLMPIITPAYPQQNSTFNVSGSTRQIIMEEFKLGLQLTDDI 360

Query 358 MLNKSTWERLFVQPPFFMKYRHFIVLIISAETSKDLLEWSGLVESKIRLLISTLERNGAI 417

ML+K TW++LF P FFMKY+HFIVL++SAE+ +D LEW GLVESK RLLI TLERN I

Sbjct 361 MLSKQTWDKLFEPPLFFMKYKHFIVLLVSAESPEDHLEWCGLVESKFRLLIGTLERNQHI 420

Query 418 TLAHINPENFMLPEPLRKPKFQYSMWFIGLEFRKMEGLNVDLTQDIQQFTETVNKHAMHI 477

TLAHINPE+F L E R+ SMWFIGLEF K E LNV+LT DIQQFTETV HA++I

Sbjct 421 TLAHINPESFSLLESQRESNTHCSMWFIGLEFAKSENLNVNLTFDIQQFTETVQNHALNI 480

Query 478 QMFKDTMKLEARHVKRKMLPNYLPSGVLKREKKYLATFTECKEQKEGRKRQSE--SSDSE 535

M K+ MKLEARHVKRK L YL +LKRE+K T + +KR S+ +SDS+

Sbjct 481 SMLKEGMKLEARHVKRKQLYQYLSPSLLKRERKTSITVKSQSNGTDSKKRLSDPGNSDSD 540

Query 536 TTVKKTRLSNEMNGT 550

KK RLS EM+ T

Sbjct 541 NPNKKIRLSEEMHST 555

Graphical representation


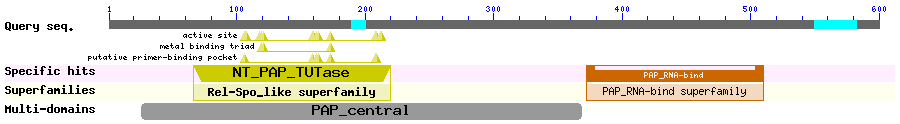

Supplement: S5 Supporting Information — (DOCX) [file pone.0115336.s005.docx]
